# Remote Desaturation of Unprotected Amines and C─H Amination of Alkanes by Polarity‐Matched N‐Radical Cations

**DOI:** 10.1002/anie.4933839

**Published:** 2026-08-05

**Authors:** Pavitra Laohapaisan, Ross M. Dare, Meredith L. Rudich, Jacob J. A. Garwood, Siyuan Gong, R. Thomas Simons, David A. Nagib

**Affiliations:** ^1^ Department of Chemistry and Biochemistry The Ohio State University Columbus Ohio USA

**Keywords:** C─H amination, C─H functionalization, heterocycles, N‐radical cations, protecting‐group free

## Abstract

A CuCl‐catalyzed strategy enables practical, selective, and efficient δ desaturation of unprotected amines. These bifunctional molecules allow rapid conversion of simple amines to privileged heterocycles in medicine. The key radical relay mechanism is an interrupted Hofmann–Löffler–Freytag (HLF) reaction, which may now be carried out without protecting groups. Based on ‘radical polarity matching’ analysis, we show here that N‐radical cations are ideal intermediates for facilitating efficient and selective 1,5‐H‐atom transfer (HAT) of unprotected amines. Mechanistic probes illustrate the scope, rate, selectivity, and utility of these distal amino alkenes. Computational studies compare the importance of kinetic (radical polarity) over thermodynamic (bond strength) factors in predicting HAT reactivity. Extension to an intermolecular variant allows the direct, desaturative C─H amination of alkanes in a net triple C─H functionalization.

## Graphical abstract

Highly electrophilic N‐radical cations enable efficient and selective H‐atom transfer (HAT) of unprotected amines. In this exceedingly practical strategy, simple CuCl catalyzes an interrupted Hofmann–Löffler–Freytag (HLF) reaction to afford δ desaturated amines, aza‐heterocycles, and C─H aminated alkanes without protecting groups.

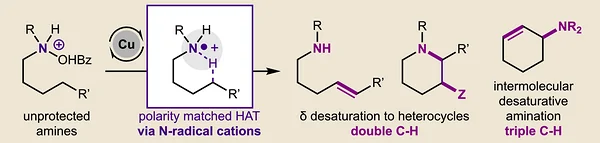

## 1 Introduction

Nitrogen‐containing heterocycles (e.g. pyrrolidines, piperidines) are ubiquitous in medicines [1, 2]. Their typical syntheses entail cyclization of bifunctional molecules containing both a nucleophile and electrophile [3, 4]. Alternatively, remote C─H amination permits use of a simpler (mono‐functional) amine precursor to access heterocycles more rapidly (Figure 1). To this end, we have sought to modernize the radical‐mediated Hofmann–Löffler–Freytag (HLF) reaction [5, 6, 7, 8], which generates pyrrolidines by a selective 1,5‐H‐atom transfer (HAT) mechanism [9]. To complement this δ C─H amination, we also introduced a Cu‐catalyzed strategy [10] to interrupt the HLF reaction and promote C─C bond formation by δ C─H functionalization (e.g., arylation, cyanation) [11, 12]. This approach now allows remote addition of many nucleophiles [13, 14, 15, 16, 17, 18] and electrophiles [19, 20, 21, 22, 23, 24, 25] to amides or sulfonamides by copper, iron, or nickel catalysis. Given its wide adaptation and utility [26, 27, 28], we sought to develop the first *protecting‐group‐free*, interrupted HLF reaction, so the products could be used directly without the need for further sulfonamide deprotection.

**FIGURE 1:**
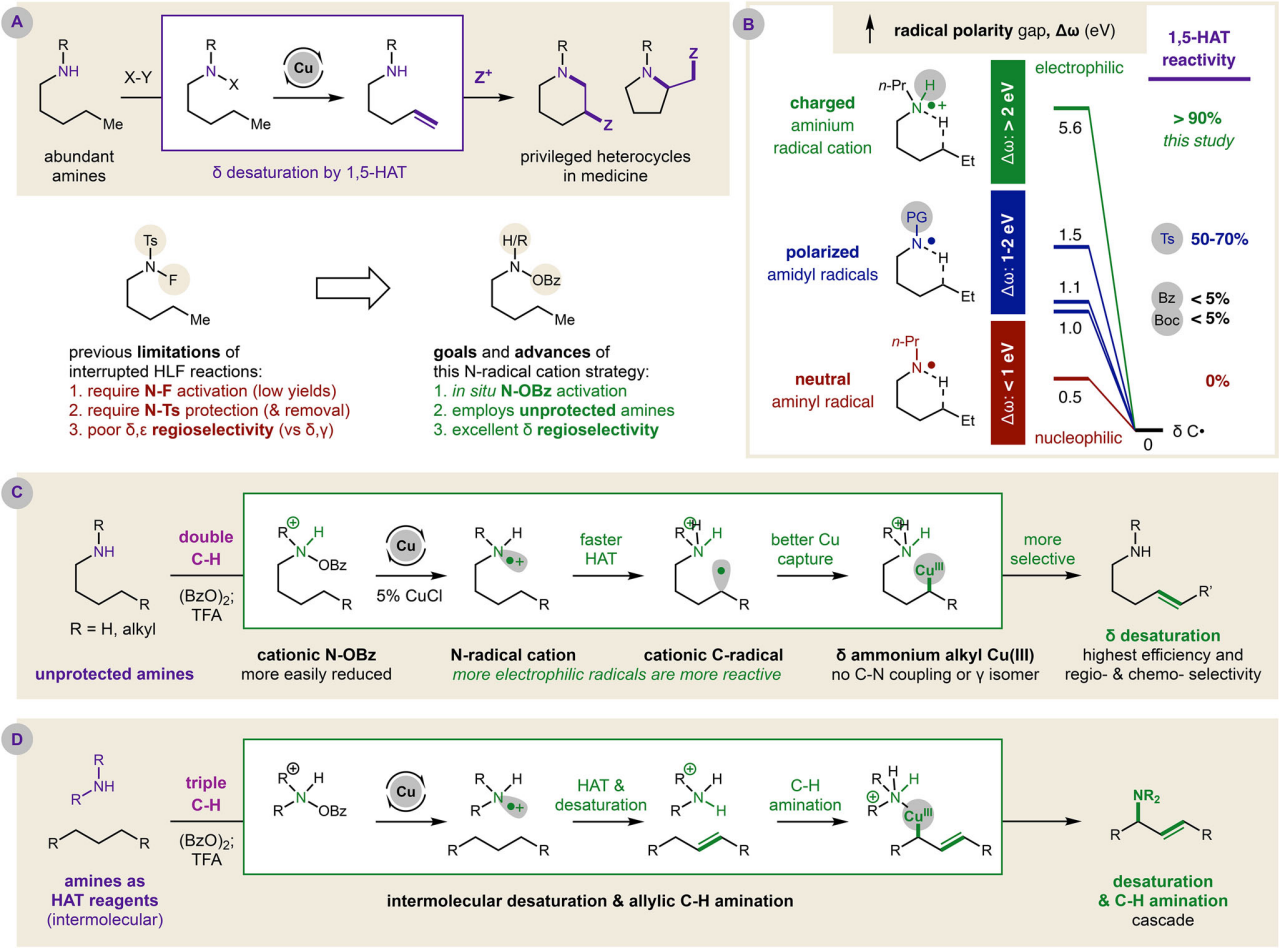
Cu‐Catalyzed remote desaturation of amines by 1,5‐H‐atom transfer (1,5‐HAT) provides rapid access to medicinally relevant heterocycles. (A) Key challenges for this interrupted Hofmann–Löffler–Freytag (HLF) reaction, entailing the need for N─F and N─Ts activation, as well as poor regioselectivity, are each addressed by this N‐radical cation strategy. (B) Insight: Increasing gaps in radical polarity, Δω, such as by aminium radical cations, improve HAT reactivity. (C, D) Strategy: N‐radical cation enables more selective and efficient HAT desaturation, enabling: (C) double C─H functionalizations of unprotected amines by remote desaturation, and (D) triple C─H functionalizations by desaturation/C─H amination cascades that employ unprotected amines as HAT reagents.

### 1.1 Mechanism

A synthetically valuable application of the interrupted HLF reaction is the δ desaturation of amines (Figure 1A) [29]. This *double*, remote C─H functionalization converts protected amines to versatile bifunctional molecules [30, 31, 32, 33, 34], which are easily cyclized to privileged heterocycles in medicines (pyrrolidines, piperidines). In the key mechanistic steps of this strategy, an N─F bond (typically TsN‐F) is reduced by a Cu(I) catalyst (either with a suitably donating ligand, or with a co‐photocatalyst) [29]. An ensuing N‐to‐C radical relay (via 1,5‐HAT) is then trapped by the resulting Cu(II) to afford an alkyl Cu(III). In the Cu‐catalyzed HLF reaction [7, 29], C─N reductive elimination affords a pyrrolidine. However, under acidic conditions and with a sterically hindered ligand, *regioselective* desaturation provides the alkene and regenerates the Cu(I) catalyst [29]. Regioselectivity of the desaturation is critical, since other alkene isomers cannot be bifurcated to access both five‐ and six‐membered heterocycles.

### 1.2 Challenge

A critical element of this radical relay is the need for a sulfonamide protecting group. While not easily removed from the products (requiring harsh reductants or tailored reagents), they are essential for the 1,5‐HAT mechanism. For instance, whereas sulfonamides are effective HAT reagents, even simple amides or carbamates are often not electrophilic enough to facilitate HAT [35]. Given our interest in quantifying *radical polarity* effects [36], we noted that HAT of relatively electron‐rich, *nucleophilic* C─H bonds are best mediated by *electrophilic* radicals [37, 38], such as highly electron‐deficient amidyl [39, 40, 41], sulfonamidyl [42, 43, 44], iminyl [45, 46, 47, 48, 49], and imidate [50, 51, 52] radicals. In pursuit of using unprotected amines as synthetically more practical and efficient HAT reagents, we turned to a radical polarity analysis of N‐mediated HAT.

## 2 Results and Discussion

### 2.1 Polarity Quantification

To determine the kinetic impact of radical polarity on HAT reactivity, we calculated the difference (Δ) in global electrophilicity (ω) [53] between a secondary C‐radical and various candidate N‐radicals for promoting 1,5‐HAT (Figure 1B; see Supporting Information for details). In this study, a clear trend emerged, wherein reactivity highly correlates with a difference in radical polarity between the N‐ and C‐radicals. Specifically, the *neutral* aminyl radical, which is nearly as nucleophilic as the δ C• (Δω = 0.5 eV), does not promote HAT reactivity (0%). Similarly, the amidyl and carbamate radicals, which are only *slightly polarized* versus their δ C• (Δω ∼1 eV), are also ineffective (< 5%). Conversely, the uniquely effective sulfonamide (Ts: 50%–70%) is polarized enough (Δω = 1.5 eV) to accelerate HAT. To extend this observation, we investigated *charged* aminium radical cations, which are *highly electrophilic* (Δω >5 eV vs C•). Here, we show N‐radical cations are *the most rapid and efficient* mediators of 1,5‐HAT (>90%)—far superior to any protecting group we have investigated over the past decade.

### 2.2 Protecting‐Group Free Strategy

The highly polarized, charged aminium benefits *every* key step of the interrupted HLF mechanism (Figure 1C) [54]. First, the radical precursor is more easily and efficiently prepared by directly combining an unprotected amine with benzoyl peroxide [55]. After acid addition, this cationic N‐OBz salt is then more easily reduced [56, 57, 58, 59]—perhaps even by a simple Cu(I) salt—due to its electron‐deficiency. Importantly, the *highly electrophilic* N‐radical cation, which efficiently adds to π bonds [60, 61], kinetically favors 1,5‐HAT by accelerating an early transition state. The resulting δ C‐radical, which is also more reactive due to the inductively withdrawing ammonium substituent, then more rapidly captures Cu(II) over byproduct formation by intermolecular HAT (typically ∼20% with N─Ts). Last, but not least, we anticipated the δ ammonium would improve both chemo‐ and regio‐ selectivity—precluding C─N coupling by negated N‐nucleophilicity and inductively influencing the reductive elimination away from the salt. Notably, employing unprotected amines as HAT reagents in an *intermolecular* fashion (Figure 1D) would have similar reactivity and selectivity benefits, enabling triple C─H functionalization by a radical, desaturation/C─H amination cascade.

### 2.3 Discovery

To test our design (Figure 2), the N‐OBz of bis‐*n*‐hexyl amine was prepared by combining a 1:1 mixture of the amine, benzoyl peroxide, and base. The cationic salt was then generated in situ by adding acid (1 equiv) and catalyst (5% CuCl). The key factors for obtaining δ desaturated **1** in the best yield and selectivity are highlighted below. Namely, *solvent* choice critically impacts reactivity [62]. For example, THF and MeCN promote full starting material (SM; **S1**) consumption, but little product formation (<2%), with reduction as the major byproduct. Alternatively, perfluoro alcohols provide increased reactivity and selectivity—with HFIP (66% yield, >10:1 rr) outperforming TFE (15% yield, 7:1 rr). Importantly, *acid* choice impacts regioselectivity most strongly—by promoting the hypothesized ammonium. For example, the stronger acid, TFA, affords **1** more regioselectively (>10:1 rr; δ desaturation over γ or ε) than DCA (Cl_2_AcOH: 7:1 rr) or AcOH (1:2 rr). The outlier, MsOH, is too strong and prevents N‐OBz reduction.

**FIGURE 2:**
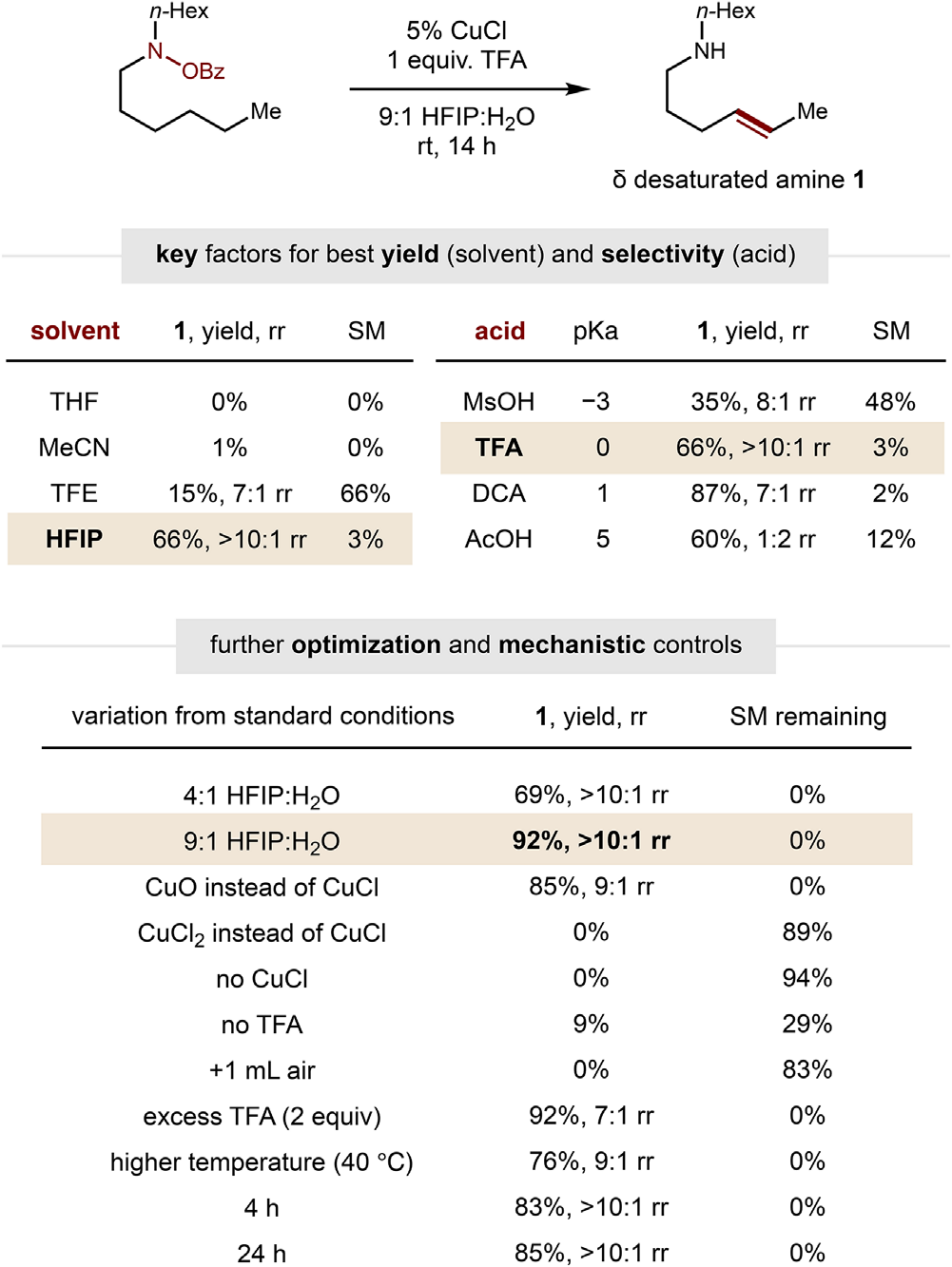
Development of protecting‐group‐free, remote desaturation. *Conditions*: R_2_N‐OBz (1 equiv, 0.2 mmol), TFA (1 equiv), CuCl (5 mol%), 9:1 HFIP:H_2_O (1 mL), 23°C, 14 h. Yield and rr (^1^H NMR) of HCl salt.

Other important optimization parameters and mechanistic controls include the addition of 10 vol% H_2_O, which improves efficiency (9:1 HFIP: H_2_O = 92% yield, >10:1 rr) versus no H_2_O addition (HFIP only: 66% yield, >10:1 rr) or greater H_2_O concentration (4:1 HFIP: H_2_O = 69% yield, >10:1 rr). As expected, CuCl and TFA are both critical for promoting efficient reactivity, and air must be excluded from this radical relay. Other variations, which are only slightly deleterious, include use of excess TFA (2 equiv; 92%, 7:1 rr), higher temperature (40°C; 76%, 9:1 rr), and varying reaction times (4–24 h: 83%–85%, >10:1 rr).

### 2.4 Synthetic Scope

To quantify the synthetic utility of this remote radical desaturation, we designed several amine probes and subjected them to this Cu‐catalyzed radical relay (Figure 3). We noted the other alkyl group (R) of the secondary amine had minimal effect on desaturation. Thus, truncated R groups, lacking a δ C─H (e.g. *n*‐propyl **2**, *t*‐butyl **3**) were also tolerated in this δ desaturation. Next, we systematically placed a methyl group at varying positions along the alkyl chain of the amine. In these cases, we were pleased to find high δ selectivity is retained despite the presence of α or β substituents (**4**‐**5**: >10:1 rr), while the γ methyl probe provides more thermodynamic competition (**6**: 3:1 rr). Cyclic alkenes are also generated by this desaturation, as in the case of the five‐ and six‐membered rings (**7**‐**8**). Additionally, common alkaloid motifs, such as cyclic amines are tolerated (e.g., coniine analog **9**).

**FIGURE 3:**
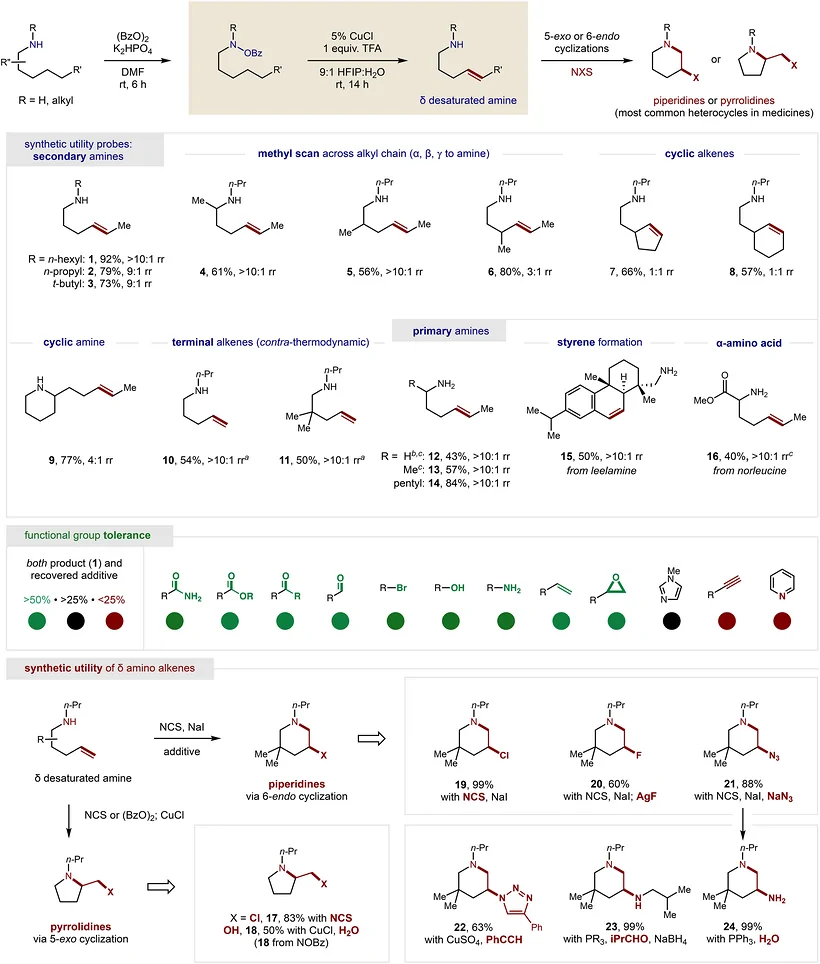
Remote desaturation: reaction scope, chemoselectivity, and synthetic utility of distal amino alkenes. *Conditions*: R_2_N‐OBz (0.2 mmol, 1 equiv), CuCl (5 mol%), TFA (1 equiv), 9:1 HFIP:H_2_O (1 mL), 23°C, 14 h. Yield and regioselectivity ratio (rr) by ^1^H NMR of HCl salt. Functional group tolerance investigated by performing desaturation in the presence of an additive and measuring ensuing product formation and additive recovery. See Supporting Information for full details. *^a.^* HFIP only. *^b.^* 10% CuO. *^c.^* 40°C.

If a shorter *n*‐pentyl amine is desaturated, then terminal alkenes are accessed *contra*‐thermodynamically (**10**‐**11**)—in preference to their internal olefin isomers. Simple primary amines are also amenable to this strategy, such as *n*‐hexyl amine (**12**) and its α substituted analogs (**13**‐**14**). To our surprise, and in contrast with our previous N─Ts variant, styrenes may also be formed (e.g. leelamine analog **15**)—a testament to the mildness of this protecting‐group‐free version.

Most notably, α‐amino acids may be desaturated, affording valuable δ desaturated analogs of norleucine (**16**). This key substrate class has not been accessible by any protecting‐group‐based method, as the acidic α‐C─H of an N─Ts (or any protected amine) is prone to elimination to form imines.

### 2.5 Chemoselectivity and Robustness

Given the expected high utility of this desaturation, we next investigated functional group tolerance in a similarly systematic manner. By adding various reactive groups—common in organic synthesis and medicinal chemistry—to the reaction, we probed the tolerance to this motif (reaction efficiency) as well as its likely amenability (recovered additive). With a threshold of >50% for both yield and additive recovery (green circle), we were pleased to find that various carbonyls are tolerated, including amides, acids, esters, ketones, and aldehydes. Moreover, nucleophiles (alcohols, amines, alkenes) and electrophiles (epoxides, halides) are also suitable. In contrast, highly nucleophilic N‐Me‐imidazole is only modestly withstood (black), but alkynes and pyridines are not tolerated (red). Based on these studies, additional examples (with functional groups embedded in the amine starting materials) are included in the Supporting Information.

### 2.6 Synthetic Utility

These bifunctional products are valuable in streamlining access to novel heterocycles. To this end, we were pleased to find that such unprotected, δ desaturated amines are easily converted to pyrrolidines (via 5‐*exo* cyclization) and piperidines (via 6‐*endo* cyclization). In the former case, we accessed 2‐Cl‐methyl pyrrolidine **17** (with NCS) as well as 2‐OH‐methyl pyrrolidine **18** (with 5% CuCl, H_2_O, from the N‐OBz starting material). In contrast to the simple pyrrolidines obtained in the HLF reaction, these products contain an additional alcohol or halide handle for further synthetic manipulation.

Similarly, we modified a procedure to enable direct access to *unprotected* piperidines by net 6‐*endo* cyclization [63, 64]. In this way, we synthesized 3‐Cl‐piperidine **19** (with NCS, NaI), 3‐F‐piperidine **20** (with NCS, AgF), and 3‐azido‐piperidine **21** (with NCS, NaN_3_). Although *gem*‐di‐methyl substitution is not essential, it ensures excellent 6‐*endo* (over 5‐*exo*) selectivity in the cyclization [63]. To illustrate the value of these 3‐substituted piperidines, we subjected azido piperidine **21** to a Cu‐catalyzed click reaction (**22**), as well as Ph_3_P‐mediated reduction (with or without reductive alkylation) to access the alkylated (**23**) or free amine (**24**).

### 2.7 Biomolecule Applicability

To further probe the utility of this remote desaturation with more complex molecules, we expanded our investigations to include medicines, metabolites, fragrances, and natural products (Figure 4). In an enhanced evaluation of functional group tolerance, we investigated natural products, cinchonidine and estrone, as well as medicines, febuxostat and gemfibrozil. To our delight, in each of these cases, the desaturation of **1** also occurred with excellent regioselectivity, illustrating that these mild conditions are tolerated by complex, valuable molecules. Moreover, in three of these four cases, the biomolecule was recovered in >70% yield.

**FIGURE 4:**
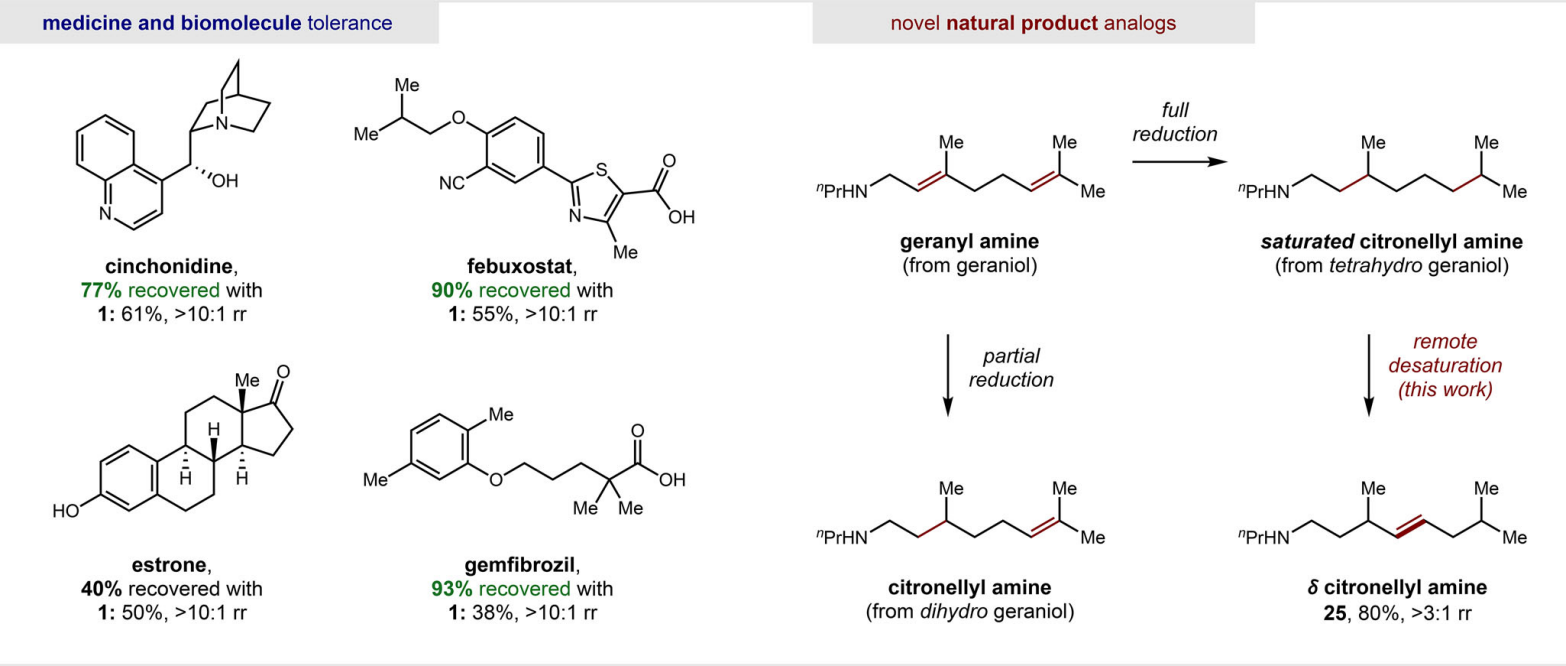
Biomolecule applicability of remote desaturation: Expanded chemoselectivity and functional group tolerance investigation includes medicines, metabolites, fragrances, and biomolecules, as well as synthetic access to unnatural isomers of natural products.

Next, we considered the rapid synthesis of unnatural analogs of readily available natural products by this strategy. For example, propyl geranyl amine, easily obtained from geraniol, can be partially reduced to the fragrant molecule citronellyl amine (or citronellol) by selective activation of the alkene closer to the amine (or alcohol) functional group. In contrast, the fully saturated, *tetrahdro* geraniol analog, is even easier to access. By applying this regioselective desaturation technology—without the need for a protecting group—δ alkene **25** is instead obtained both efficienctly with respect to this reaction (80%) and the overall synthetic sequence.

### 2.8 Kinetics Versus Thermodynamics

To elucidate the importance of radical polarity in governing HAT reactivity, we completed DFT analysis of the 1,5‐HAT reaction energies for each of the three classes of N‐radical (Figure 5; see Supporting Information for full details and coordinates). Specifically, we quantified *thermodynamic* (ΔG_rxn_) and *kinetic* (ΔG_ts_) differences for HAT mediated by the: (i) **neutral** aminyl radical, (ii) **polarized** amidyl radical, or (iii) **charged** N‐radical cation. In comparing the three scenarios, we noted the **neutral** case is *disfavored* for *both* thermodynamic and kinetic reasons (unfilled red boxes). Both the HAT endothermicity (ΔG_rxn_: +2.3 kcal/mol) and its high transition state barrier (ΔG_ts_: 18.9 kcal/mol) explain why *no 1,5‐HAT reactivity* has been observed for such **neutral** N‐radicals (red box, right). In comparison, HAT by **polarized** amidyl radicals is significantly more exothermic (ΔG_rxn_: −6.3 kcal/mol) with only half the transition state barrier (ΔG_ts_: 9.9 kcal/mol) (see unfilled boxes). These effects explain why *all previous* 1,5‐HAT (interrupted HLF) reactivity has required such **polarized** amidyl radicals (grey box, right). Surprisingly, however, the superior reactivity observed herein for **charged** N‐radical cations (green box, right) is neither explained by the relatively thermoneutral character (ΔG_rxn_: +0.4 kcal/mol) nor modest kinetic barrier (ΔG_ts_: 11.2 kcal/mol).

**FIGURE 5:**
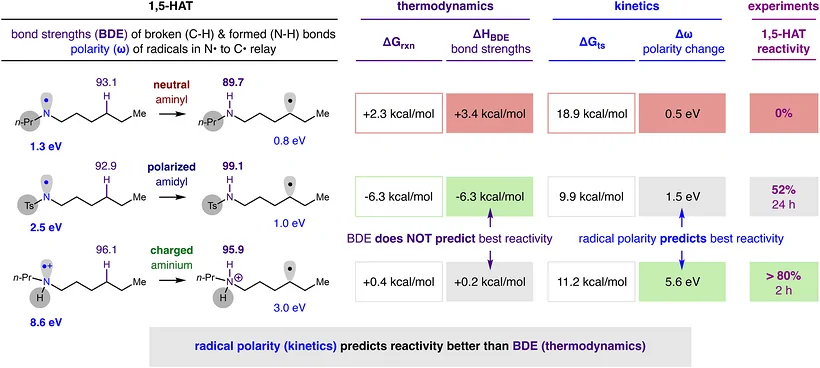
Kinetics versus thermodynamics: DFT Analysis of 1,5‐HAT for each class of N‐radical (aminyl, amidyl, and aminium radical cation) shows that kinetics, specifically the difference in radical polarity (Δω) between the key N‐ and C‐radicals best predicts observed experimental reactivity.

Seeking a better explanation, we then probed the *key components* of these thermodynamic and kinetic factors. In these simplified calculations, a difference in **bond strengths** (ΔH_BDE_) of the broken C─H and formed N─H bonds was computed for each class of N‐radical. This provides a much faster method to access the same trend showing that **polarized** amidyl radicals (filled green box) should be the most favored, while **neutral** aminyl radicals (filled red box) should be least favored, and **charged** N‐radical cations (filled gray box) should be in the middle. However, since this simplification of the *thermodynamic* landscape still does not explain observed 1,5‐HAT reactivity, we then turned to a key *kinetic* factor: **radical polarity**.

To our delight, the very simple approximation of *kinetics* by measuring radical polarity, or global electrophilicity (ω), is the only one of these four factors that correctly predicts observed reactivity. Specifically, the **polarity change** (Δω) column in Figure 5 *matches* the experimental 1,5‐HAT reactivity, where the ranking of best (green box), middle (grey box), and worst (red box) reactivity are the same in both columns for the first time. Since **neutral** aminyl radicals are similar in polarity to the remote C‐radical (Δω = 0.5 eV), the 1,5‐HAT reactivity is *disfavored*, as observed (0%). On the other hand, **polarized** amidyl radicals are much more electrophilic than C‐radicals (Δω = 1.5 eV)—explaining the increased 1,5‐HAT reactivity (52%, 24 h). Yet, this analysis clearly shows **charged** N‐radical cations, which are *highly electrophilic* and have the largest polarity change from the forming nucleophilic C‐radical (Δω = >5 eV), are *most favored*. Importantly, this prediction matches observed experimental reactivity—with this N‐radical cation‐enabled strategy providing much higher yields in shorter reaction times (>80%, 2 h). Thus, we have developed the **mnemonic** shown in Figure 1, whereby 1,5‐HAT with a polarity change (Δω) greater than 2 eV is *highly favored*, whereas a smaller gap of 1–2 eV is only *modestly favored*, and a polarity gap less than 1 eV is *disfavored*.

An explanation for this effect is that polarity differences (Δω) between radicals involved in HAT reactivity can predict transition‐state stability. Polar effects are known to arise from greater stabilization of polarized transition states [38]. Indeed, our DFT analysis shows the N‐radical cation, polarized transition state exhibits an activation barrier ∼8 kcal/mol *lower*—and a reaction free energy ∼2 kcal/mol *more favorable*—than those of the corresponding neutral aminyl radical. Quantification of the accessibility of byproduct pathways (e.g., β‐scission) will likely further explain the advantage of the N‐radical cation pathway over the polarized amidyl mechanism.

### 2.9 Mechanism

Given our experimental observations, we propose the following mechanism for this Cu‐catalyzed remote desaturation (Figure 6A). A Cu(I) precatalyst (e.g., CuCl) is sufficiently reducing (–0.3 V; all redox potentials vs SCE) to engage cationic N‐OBz **A** (> +1 V) [56] in a single‐electron transfer (SET) mechanism. Since this cation is *more easily reduced* than the neutral N─O species, a photocatalyst is no longer needed to initiate the radical relay [29]. The resulting N‐radical cation **B** then mediates a radical relay (via 1,5‐HAT) to form δ C‐radical **C**. This key step, as we have shown in Figure 5 is *most polarity matched*, wherein the *highly electrophilic* N‐radical cation (ω > 8 eV) is best suited to generate a more nucleophilic C‐radical, given the large polarity change (Δω > 5 eV). The remote radical **C** is then captured by Cu(II) to generate alkyl Cu(III) **D**. Importantly, the δ ammonium substituent is protected from deprotonation by loss of a highly acidic HX (e.g., TFA)—preventing formation of the undesired metallocycle **E**. Thus, the acid is also key to promoting *chemoselectivity* by inhibiting the HLF reaction to form a pyrrolidine by C─H amination. The highly electrophilic alkyl Cu(III) **D** is then poised to undergo a formal β hydride elimination [65] to the desaturated product—regenerating the Cu(I) catalyst. Importantly, the acid also promotes *regioselectivity* of this elimination, by polarizing the alkyl chain so that an H is eliminated from the more hydridic *distal* C─H bond (further from the inductively withdrawing ammonium). Thus, we note **four key roles** for the activation of the N‐radical by an acid—improving each of the following: (1) N─O bond reduction, (2) radical relay kinetics, (3) chemoselectivity, and (4) regioselectivity.

**FIGURE 6:**
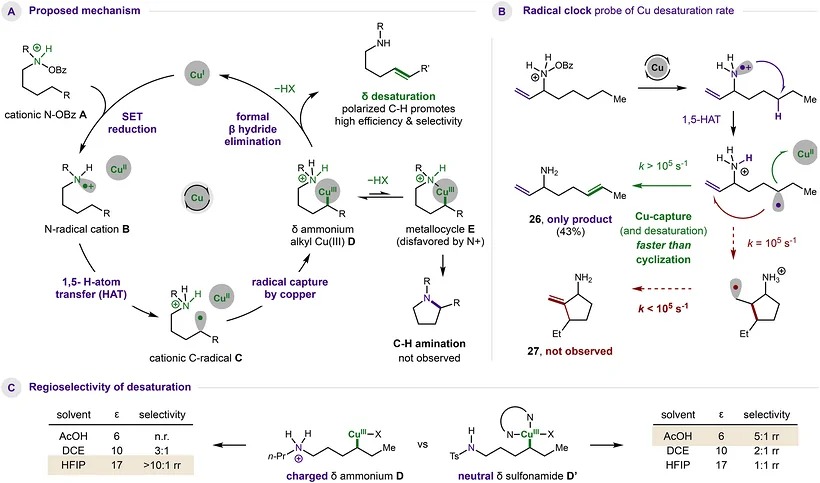
(A) Proposed mechanism for Cu‐catalyzed radical relay desaturation. (B) Radical clock probe. (C) Solvent effects on regioselectivity in N‐radical cation vs neutral amidyl radical mechanisms.

Additional mechanistic studies were focused on elucidating the rate of the Cu‐catalyzed desaturation, as well as comparing the impact of solvent effects in the charged versus polarized N‐radical relays. In the former, a radical probe was designed wherein the relayed C‐radical is poised for alkene capture by 5‐*exo*‐trig cyclization (Figure 6B). This rapid cyclization (*k* = 10^5^ s^−1^) [66] would result in a terminal alkyl radical that is desaturated to allyl amine **27**–if desaturation is slower than 10^5^ s^−1^. However, this product was not observed. Rather, direct desaturation of the distal radical provides the only observed product—1,6‐diene **26**—with the allyl group remaining intact. Thus, we conclude that radical capture by Cu(II) and ensuing desaturation is *quite rapid* (*k* > 10^5^ s^−1^).

Lastly, we noted significant solvent effects on *regioselectivity*—and a major difference between the charged and polarized N‐radical relays (Figure 6C). For example, in this TFA‐mediated, charged case (**D**), increasing selectivity is observed for *more polar* (and solubilizing) solvents [62, 67], where HFIP (ε = 17; >10:1 rr) is more selective than DCE (ε = 10; 3:1 rr) and AcOH (ε = 6; no reaction). In contrast, in the polarized N─Ts case (**D’**), the *least polar* solvent, AcOH is best (ε = 6; 5:1 rr), while more polar solvents decrease regioselectivity (DCE, ε = 10, 2:1 rr; HFIP, ε = 17, 1:1 rr). We had previously found that an *N,N*‐ligand is critical to boost regioselectivity in the *polarized* case (with more polar solvents likely inhibiting complexation) [29]. However, in this ligandless, *charged* environment, the more polar solvents likely benefit regioselectivity by stabilizing the δ ammonium—allowing it to direct distal desaturation (away from the ammonium) and without a ligand. Thus, this N‐radical cation strategy affects higher selectivity (>10:1 rr) via inductive *electronic* effects—versus the ligand‐based *steric* effects that we had developed previously (5:1 rr).

### 2.10 Intermolecular Desaturative C─H Amination Cascade

In a major extension of this N‐radical‐cation‐mediated strategy, we also developed an intermolecular variant to promote direct desaturative C─H amination of alkanes (Figure 7). The mechanism of this net triple C─H functionalization, shown in Figure 7A, entails two consecutive Cu catalytic cycles. In the first cycle, SET reduction of protonated N‐OBz **A** by Cu(I)Cl generates an *electrophilic* N‐radical cation **B** and Cu(II). However, in the absence of a δ C─H bond, this key intermediate now enables *polarity matched*, intermolecular HAT of a simple alkane. Capture of the resultant, *nucleophilic* alkyl radical **C** by Cu(II) leads to formation of alkyl Cu(III) **D**. Desaturation of this key intermediate then affords alkene **I** and regenerates the Cu(I)Cl catalyst. Notably, this Cu‐catalyzed intermolecular desaturation nicely complements a recently developed, photocatalytic version employing cobalt and decatungstate catalysts [34].

**FIGURE 7:**
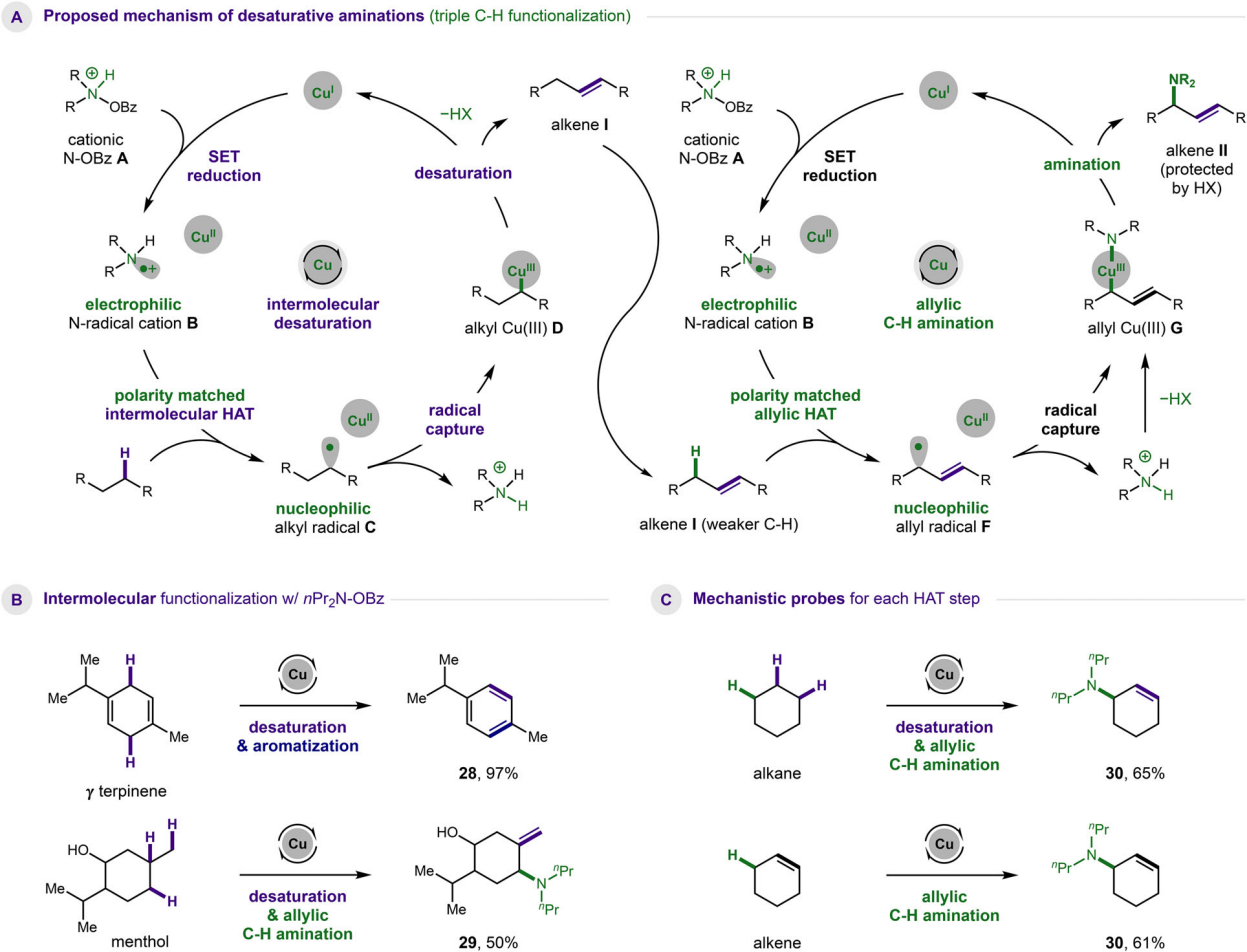
Triple C─H functionalization by desaturative C─H amination. (A) Proposed mechanism. (B) Synthetic applications. (C) Mechanistic probes.

In a second catalytic cycle, parallel activation of N‐OBz·HX **A** by CuCl generates another N‐radical cation **B** and Cu(II). However, in this case, polarity matched HAT is selective for the weaker, allylic C─H of alkene **I**. The resultant allyl radical **F** is captured by Cu(II) to form allyl Cu(III) **G**, which entails a coordinated amine byproduct (upon reversible loss of acid). In this case, C─N reductive elimination of complex **G** regenerates Cu(I)Cl catalyst along with aminated alkene **II**. Notably, upon re‐protonation, this allyl amine product is protected from further reaction with **B** thanks to polarity matching. Specifically, the inductively withdrawing ammonium (1) renders the alkene too electron‐deficient for π‐addition reactions, and (2) ensures the polarity mismatched C─H is less electron‐rich than those of the starting material (alkyl C─H) or intermediate (allyl C─H), which will preferably undergo HAT instead. Thus, product stability is ensured in this dual catalytic cycle.

To our delight, an intermolecular version is indeed promoted by employing *^n^* Pr_2_N‐OBz as the N‐radical cation precursor (Figure 7B). In a first test, the diene, γ‐terpinene (1 equiv), was desaturated to form *p*‐cymene **28**, nearly quantitatively with 5% CuCl, TFA and *^n^* Pr_2_N‐OBz (3 equiv each). Given the driving force of aromatization in this reaction, we then tested another natural product with a less activated C─H bond. In this case, we were pleased to find that the alcohol‐containing menthol is also desaturated under the same conditions—and ensuing allylic C─H amination affords amine **29**–validating our proposed cascade sequence.

To elucidate the mechanism of this triple C─H functionalization, we then subjected a pair of probes to this Cu‐catalyzed cascade (Figure 7C). Firstly, cyclohexane (1 equiv) cleanly reacts with *^n^* Pr_2_N‐OBz (3 equiv) as expected to provide allyl amine **30**. Next, the proposed alkene intermediate was validated by subjecting cyclohexene to the same procedure, which affords allyl amine **30** in a similar yield. Thus, we have shown that this mild, simple, CuCl‐catalyzed procedure to access N‐radical cations may also promote intermolecular HAT cascades of alkanes.

## 3 Conclusion

In summary, a simple, practical, and highly selective, *remote, radical desaturation* has been developed for the synthesis of bifunctional alkenyl amines, which enable rapid access to diverse, substituted heterocycles. This Cu‐catalyzed approach represents the first, interrupted HLF reaction of *unprotected* amines. Our analysis of the kinetic and thermodynamic aspects of this HAT reaction suggests the large difference in radical polarity (Δ ω)—provided by *highly electrophilic*, N‐radical cations—is key to accessing both efficiency and selectivity in this radical relay. We anticipate many other valuable methods that employ the 1,5‐HAT mechanism may now provide access to remote C─H functionalization of unprotected amines.

## Author Contributions

**Pavitra Laohapaisan**: methodology, writing – review and editing. **Ross M. Dare**: conceptualization, methodology, writing – review and editing. **Meredith L. Rudich**: methodology, writing – review and editing. **Jacob J. A. Garwood**: writing – review and editing, formal analysis. **Siyuan Gong**: investigation. **R. Thomas Simons**: investigation. **David A. Nagib**: supervision, writing – review and editing.

## Conflicts of Interest

The authors declare no conflicts of interest.

## Supporting information



The authors have cited additional references within the Supporting Information [68, 69, 70, 71, 72, 73, 74].**Supporting File 1**: anie73944‐sup‐0001‐SuppMat.pdf.

## Data Availability

The data that supports the findings of this study are available in the supplementary material of this article.
