# Supplementary material for: Remote Desaturation of Unprotected Amines and C─H Amination of Alkanes by Polarity‐Matched N‐Radical Cations
Source: Angew Chem Int Ed Engl. 2026 Aug 5;65(40):e4933839. doi: 10.1002/anie.4933839 (PMC13618280; doi:10.1002/anie.4933839)
Supplement: Supplementary file 1 [file ANIE-65-e4933839-s001.pdf]

Supporting Information for:

Remote Desaturation of Unprotected Amines and C-H Amination of Alkanes by Polarity-Matched N-Radical Cations

Pavitra Laohapaisan,† Ross M. Dare,† Meredith L. Rudich,†
Jacob J. A. Garwood,† Siyuan Gong, R. Thomas Simons, and David A. Nagib*

Department of Chemistry and Biochemistry, The Ohio State University,
151 W. Woodruff Ave., Columbus, OH, 43210, United States

Corresponding Author
*E-mail: nagib.1@osu.edu

Table of Contents

Section		Page
I.	General Information	S3
II.	General Procedures	S4
III.	Additional Substrates and Functional Group Tolerance	S8
IV.	Development of δ Desaturation	S11
V.	Synthesis and Characterization of Starting Materials	S13
VI.	δ Desaturated Amine Product Characterization	S24
VII.	Post-Functionalization: Synthetic Utility of Amino Alkenes	S30
VIII.	Applications to Medicine and Biological Molecules	S34
IX.	Mechanistic Studies, Radical Clock, & Regioselectivity Assignment	S35
X.	Intermolecular Desaturation	S40
XI.	Characterization of the Additional Substrates	S41
XII.	Computational Details	S45
XIII.	References	S72
XIV.	NMR Spectra	S73

I. General Information

All **reagents** were purchased from Sigma-Aldrich, Alfa Aesar, Acros, TCI, or ChemImpex.

$(BzO)_2$ used was a 75% wt solid remainder weight water.

CuCl was stored and weighed out in a glovebox for best reaction efficiency.

Solvents: CH_2Cl_2, THF, DMF were dried and degassed with N_2 using an Innovative Technology solvent system. HFIP and H_2O were degassed by bubbling N_2 through the solvents using a Schlenk line prior to use.

Silica gel: Silicycle F60 (230-400 mesh) was used or a CombiFlash® Automated Flash Chromatograph for flash column chromatography.

Thin layer chromatography (TLC) analyses were performed using Merck silica gel 60 F254 plates and visualized under UV (254 nm), $KMnO_4$, or iodine stain.

^{1}H, ^{19}F, ^{13}C NMR spectra were recorded using a Bruker AVIII 400 or AVIII 600 MHz NMR spectrometer. ^{1}H NMR and ^{13}C NMR chemical shifts are reported in parts per million and referenced with respect to $CDCl_3$ (^{1}H: residual $CHCl_3$ at δ 7.26, ^{13}C: $CDCl_3$ triplet at δ 77.16). ^{1}H NMR data are reported as chemical shifts (δ ppm), multiplicity (s = singlet, bs = broad singlet, d = doublet, t = triplet, q = quartet, quint = quintet, sext = sextet, m = multiplet, app t = apparent triplet, app q = apparent quartet, app qd = apparent quartet of doublets), coupling constant (Hz), relative integral. ^{19}F NMR data are reported as chemical shifts (δ ppm).

High resolution mass spectra were obtained using Bruker MicrOTOF (ESI) or a Thermo Scientific Orbitrap Exploris MX with Vanquish UHPLC system (LCMS).

Gas Chromatography experiments were performed on Agilent Technologies 7820 GC System.

Infrared (IR) spectra were recorded using a Thermo Fisher Nicolet iS10 FT-IR and are reported in terms of frequency of absorption (cm^{-1}).

II. General Procedures

δ Desaturation of Amines: General Procedure 1 (**GP1**)

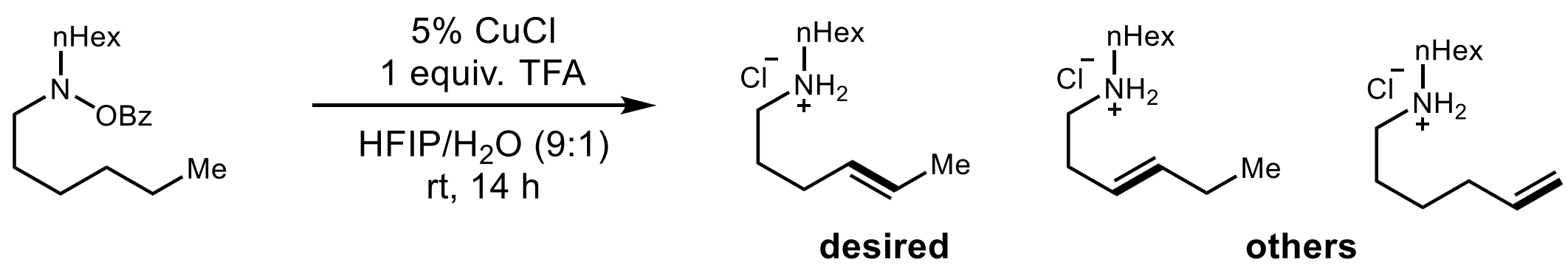

CuCl (1.0 mg, 5mol%) and a stir bar were added to an 8 mL vial inside a glovebox before sealing and removing from the glovebox. In a separate vial, O-Benzoylhydroxylamine (R_2N-OBz; 0.2 mmol, 1 equiv) and a stir bar were added before evacuating and refilling with N_2 (3x). Next, hexafluoroisopropanol (HFIP; 900 uL) and water (H_2O; 100 uL) – both degassed (by sparging with N_2 for 1 h) – and TFA (15.3 uL, 1.0 equiv) were added. This protonated R_2N-OBz solution was then added to the CuCl-containing vial while under positive N_2 pressure, before being sealed with parafilm and electrical tape. The reaction was allowed to stir at 25ºC until reaction completion (4-14 h). Upon completion, the solvent was removed under vacuum and the reaction was diluted in DCM and quenched by adding 2 mL of 1M NaOH, 1 mL of saturated EDTA (tetrasodium ethylenediaminetetraacetic acid) solution, and stirring for 30 minutes. After 30 minutes, the aqueous layer was removed and extracted twice with 1 mL of DCM for 5 minutes. The organic layers were combined, and 0.5 mL of 2M HCl in Et_2O (5 equiv) was added. Then solvent was removed under vacuum to afford the protonated alkene product.

N-OBz Hydroxylamine Synthesis: General Procedure 2 (**GP2**)

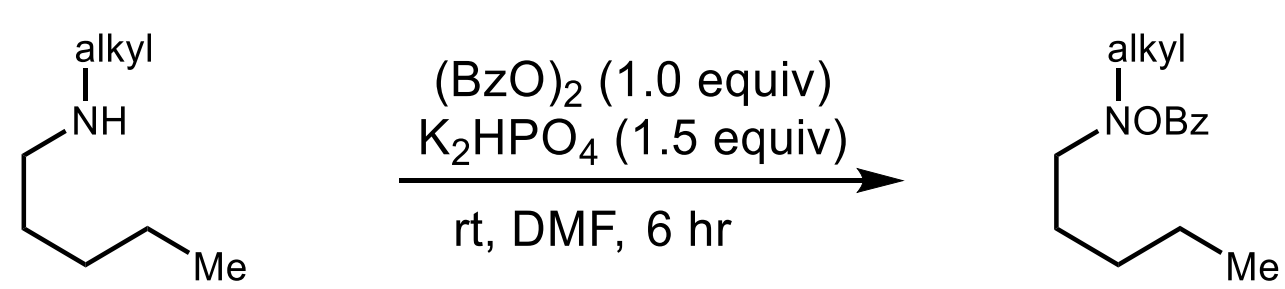

Synthesized according to a reported procedure[55]: A round-bottom flask was charged with a stir bar, K_2HPO_4 (7.8 g, 45 mmol, 1.5 equiv) and DMF (120 ml, 0.25M). $(BzO)_2$ (75%wt, 9.7 g, 1 equiv) was added to the stirred suspension followed by dropwise addition of alkylamine (synthesized according to GP3 and GP4) (36 mmol, 1.2 equiv). The reaction was stirred for 6 h at 25°C monitored by TLC. Upon completion the reaction was quenched by addition of 120 mL H_2O and further stirring for 30 min. The reaction mixture was extracted with Et_2O (3 x 100 ml) and the combined ethereal layers washed with water (4 x 50 ml) and brine (1 x 20 ml). The crude mixture was dried over Na_2SO_4 and concentrated. The residue was purified by column chromatography on silica gel with a gradient of 5% EtOAc/Hex.

Amine Synthesis by Reductive Amination: General Procedure 3 (**GP3**)

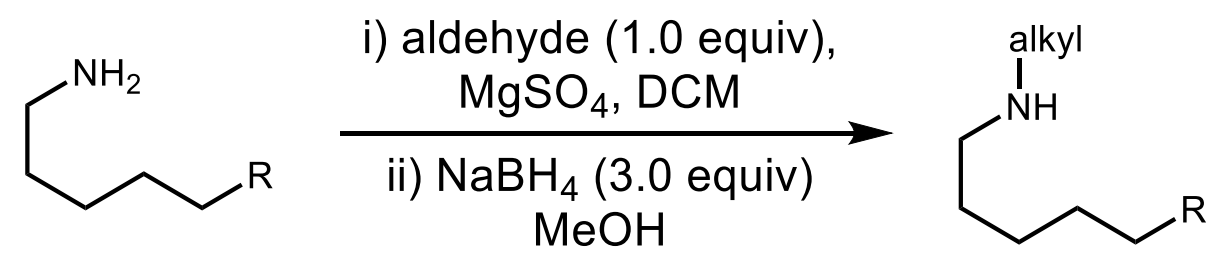

To a clean, dry round bottom flask was added a magnetic stir bar and primary amine (1 equiv) at room temperature. The substrate was dissolved in DCM (0.25 M), followed by addition of aliphatic aldehyde (1 equiv), and $MgSO_4$. The reaction was allowed to stir at room temperature for 3 h (monitored by GC for imine formation). Upon completion the reaction mixture was filtered and concentrated. The residue was redissolved in MeOH (0.2M) and cooled to 0°C, followed by addition of $NaBH_4$ (3 equiv). The reaction mixture was allowed to warm to room temperature and stir for 2 h. H_2O was added to quench the reaction and stirred for an additional 20 min followed by extraction of the aqueous layer with DCM (3 x 100 mL). The combined organic layers were washed with brine, dried over Na_2SO_4, and concentrated *in vacuo*. The crude material was used directly or salted out using HCl in MeOH.

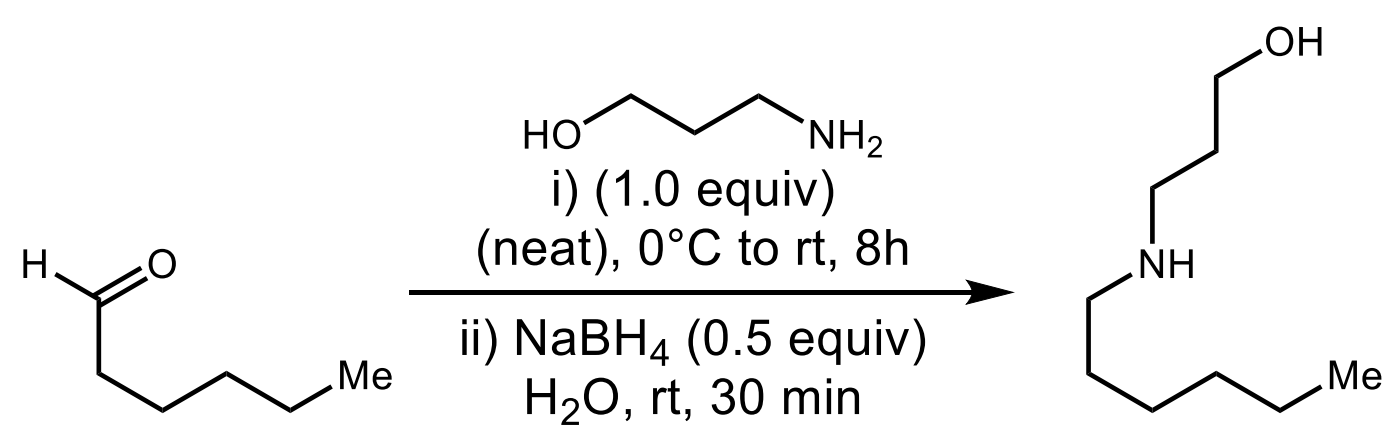

To a clean, dry round-bottom flask equipped with a magnetic stir bar was added hexanal (1.0 equiv), and the reaction mixture was cooled to 0 °C. 3-Aminopropan-1-ol (1.0 equiv) was added dropwise under neat conditions. After complete addition, the cooling bath was removed, and the reaction mixture was allowed to warm to room temperature and stirred for 8 h. Subsequently, an aqueous solution of $NaBH_4$ (0.5 equiv, 1.0 M in H_2O) was added dropwise. The reaction mixture was stirred for 30 min, or until gas evolution had ceased. The mixture was then diluted with H_2O and extracted with DCM (3 × 100 mL). The combined organic layers were dried over Na_2SO_4, filtered, and concentrated in vacuo to afford 3-(hexylamino)propan-1-ol in quantitative yield. The crude product was used directly in the subsequent reaction without further purification.

Amine Synthesis by Amide Reduction: General Procedure 4 (**GP4**)

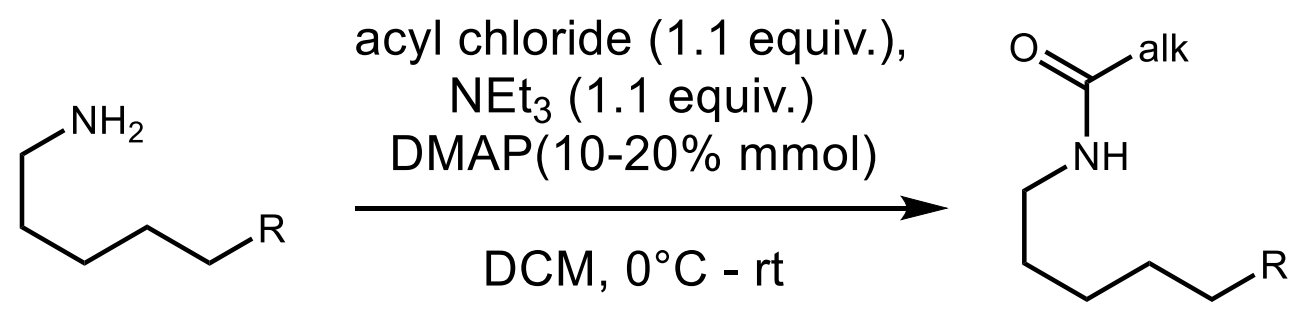

To a clean, dry round bottom flask was added a stir bar followed by a primary amine (1 equiv) at room temperature. The substrate was dissolved in DCM (0.5 M), followed by addition of NEt_3 (1.1 equiv) and DMAP (10-20%). The reaction was allowed to stir while being cooled to 0°C. After cooling, acyl chloride (1.1 equiv) was added dropwise under N_2. The reaction mixture was allowed to warm to room temperature until starting material is completely consumed (monitored by TLC analysis, usually >6 h). Upon reaction completion, the organic layer was extracted with 1M HCl (1 x 100 mL), followed by H_2O (1 x 100 mL) and brine (1 x 100 mL). The organic layers were combined and dried with $MgSO_4$ before being concentrated *in vacuo*. The crude material was purified via column chromatography (EtOAC/hexanes) prior to the LAH reduction.

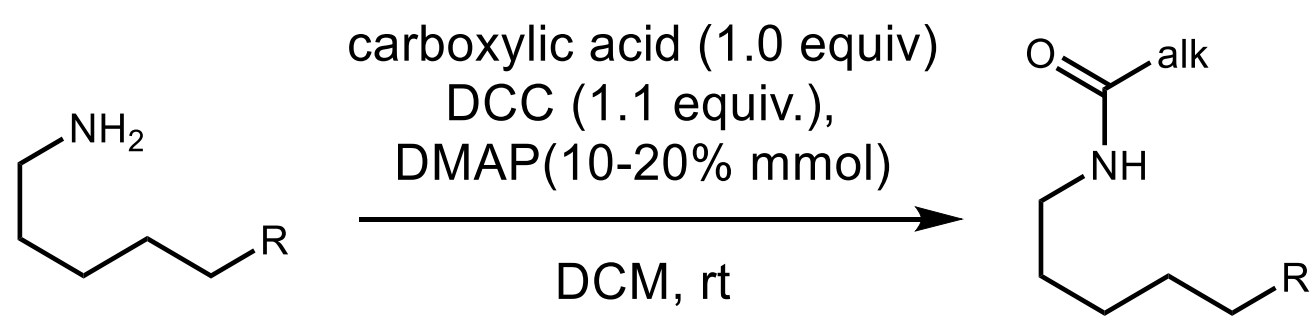

To a clean, dry round bottom flask was added a stir bar followed by a primary amine (1.1 equiv) at room temperature. The substrate was dissolved in DCM (0.5 M), followed by addition of DCC (1.1 equiv) and DMAP (10-20%). The reaction was allowed to stir briefly before carboxylic acid (1 equiv) was added. The reaction mixture was stirred at room temperature until carboxylic acid is completely consumed (monitored by TLC analysis). Upon reaction completion, the solids were filtered out from the reaction mixture. The organic layer was extracted with 1M HCl (1 x 100 mL), followed by H_2O (1 x 100 mL) and brine (1 x 100 mL). The organic layers were combined and dried with $MgSO_4$ before being concentrated *in vacuo*. The crude material was purified via column chromatography (EtOAC/hexanes) prior to the LAH reduction.

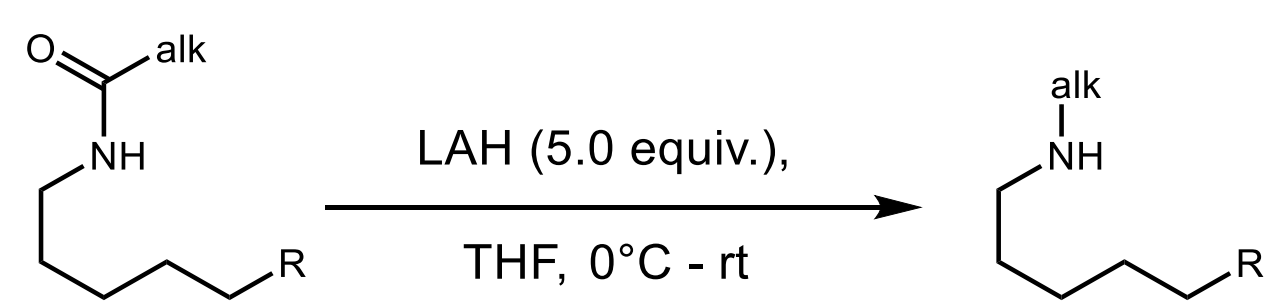

To a clean, flame-dried round bottom flask was added LAH (5 equiv) followed by a backfill of N_2 before being placed over ice and being cooled to 0°C. Anhydrous THF (roughly two-thirds of total solvent volume; 0.5 M total) was added to the LAH powder under N_2 and allowed to stir until H_2 evolution ceases. Secondary amide (1 equiv) was added dropwise as a solution in the remaining anhydrous THF under N_2. The reaction was placed on a reflux apparatus once H_2 evolution ceased and set to reflux under inert atmosphere for 12 h. Upon reaction completion, the mixture was cooled to 0°C and diluted with Et_2O. The reaction was quenched following the Fieser workup method or alternatively with sodium sulfate decahydrate (Glauber's salt) until no H_2 gas evolves.

The solids were filtered from the reaction mixture and the organic layer dried with $MgSO_4$ before being concentrated *in vacuo*. For volatile amine products, HCl (in MeOH or Et_2O) was added to form the salt prior to concentration *in vacuo*. The crude products were used directly in the benzoylation procedure (GP2).

N-OBz Hydroxylamine O-Acylation: General Procedure 5 (**GP5**)

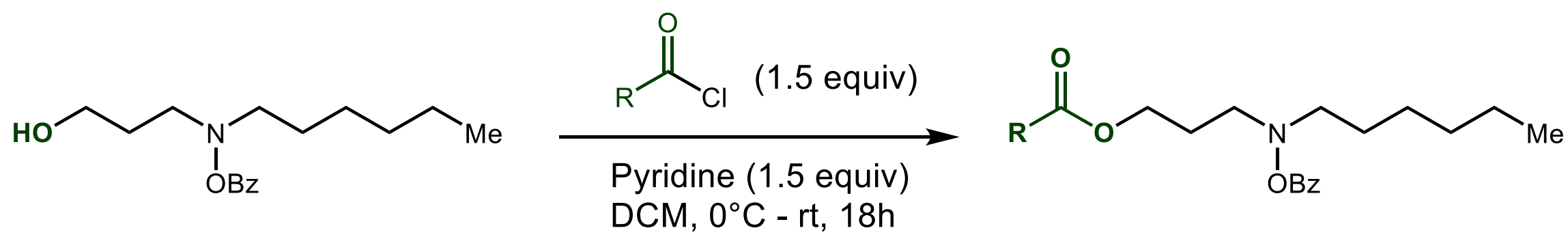

To a clean, dry round-bottom flask equipped with a magnetic stir bar was added the corresponding N-OBz hydroxylamine (1.0 equiv) and DCM (0.2 M). Pyridine (1.5 equiv) was added, and the reaction mixture was cooled to 0 °C. The corresponding acid chloride (1.5 equiv) was added dropwise, and the reaction mixture was allowed to warm to room temperature and stirred for 18h. The reaction was quenched with H_2O, and the aqueous layer was extracted with DCM (3 × 100 mL). The combined organic layers were washed with brine, dried over Na_2SO_4, filtered, and concentrated in vacuo. The crude product was purified by column chromatography on silica gel with a gradient of 5 - 15% EtOAc/Hex.

Intermolecular Desaturation of Amines: General Procedure 6 (**GP6**)

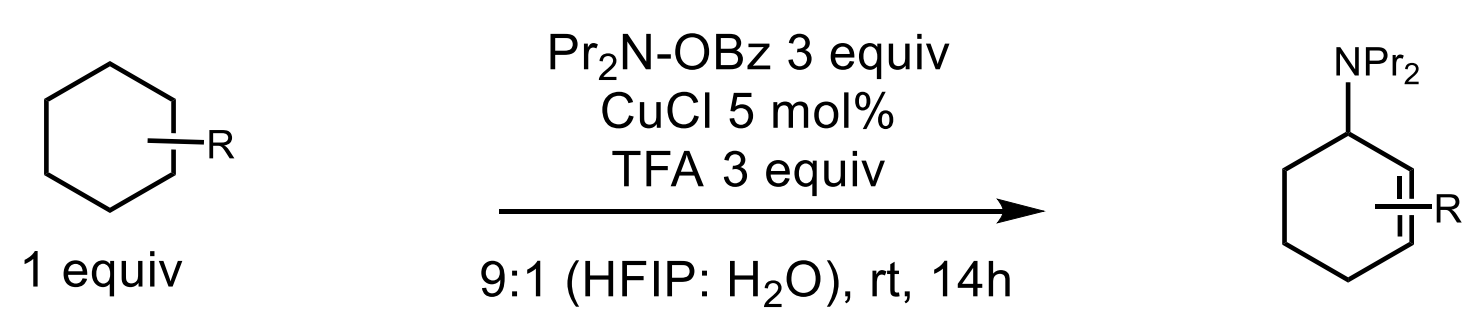

CuCl (1.0 mg, 5mol%) and a stir bar were added to an 8 mL vial inside a glovebox before sealing and removing from the glovebox. This CuCl vial was added cyclohexane (21.6 uL, 1 equiv) In a separate vial, O-Benzoyl-*N,N*-dipropylhydroxylamine (Pr_2N-OBz; 0.6 mmol, 3 equiv) and a stir bar were added before evacuating and refilling with N_2 (3x). Next, hexafluoroisopropanol (HFIP; 900 uL) and water (H_2O; 100 uL) – both Pr_2N-OBz solution was then added to the CuCl mixture-containing vial while under positive N_2 pressure, before being sealed with parafilm and electrical tape. The reaction was allowed to stir at 25ºC until reaction completion (14 h). Upon completion, the reaction was diluted in DCM (2 mL) and quenched by adding 0.5 mL of saturated EDTA (tetrasodium ethylenediaminetetraacetic acid) solution, 3M NaOH (120 mg) and stirring for 30 minutes. After 30 minutes, the aqueous layer was removed and extracted twice with 1 mL of DCM for 5 minutes. The organic layers were combined. Then the solvent was removed under vacuum to afford the alkene product.

III. Additional Substrates and Functional Group Tolerance

Table 1: Additional Substrates for δ Desaturation

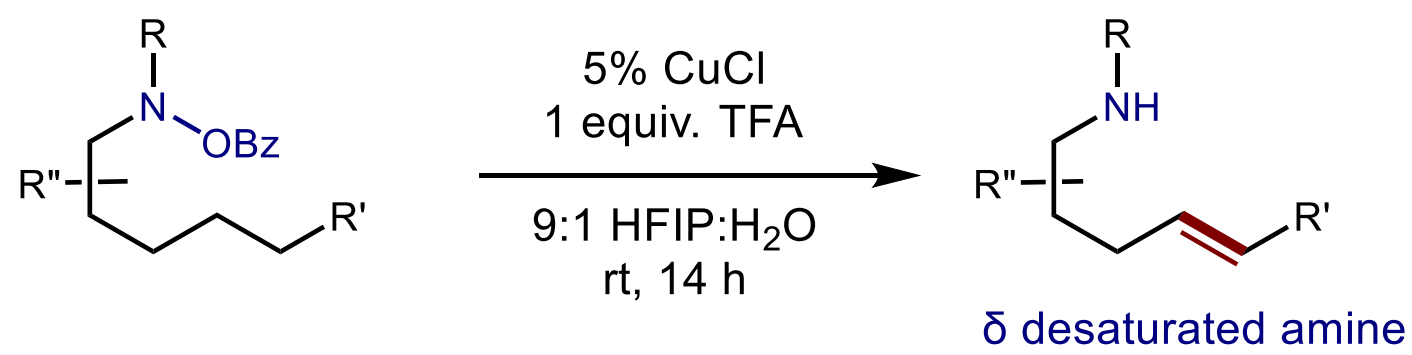

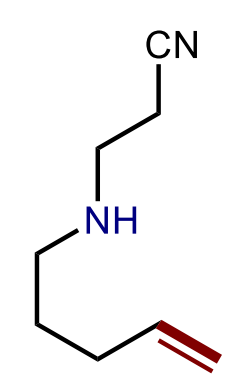

32, 52%, 4:1 rr*

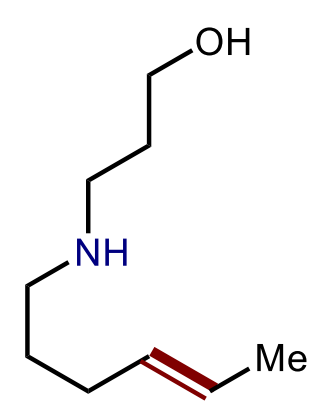

33, 22%, >10:1 rr

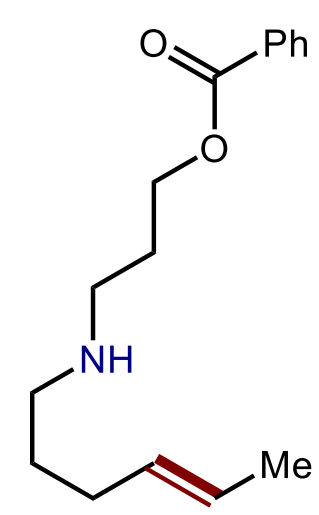

34, 66%, >10:1 rr*

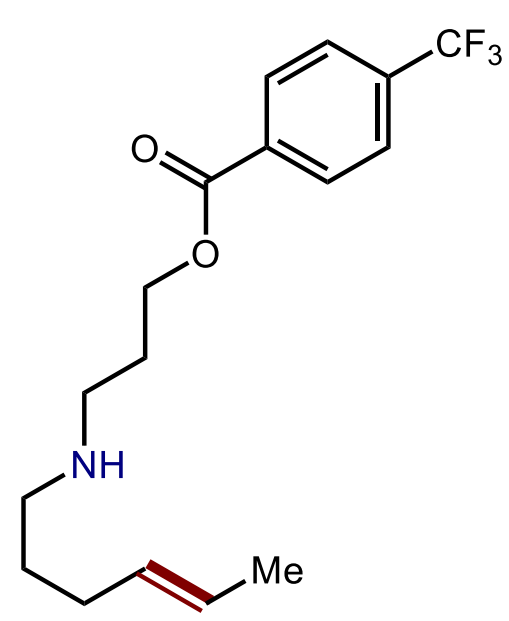

35, 73%, >10:1 rr*

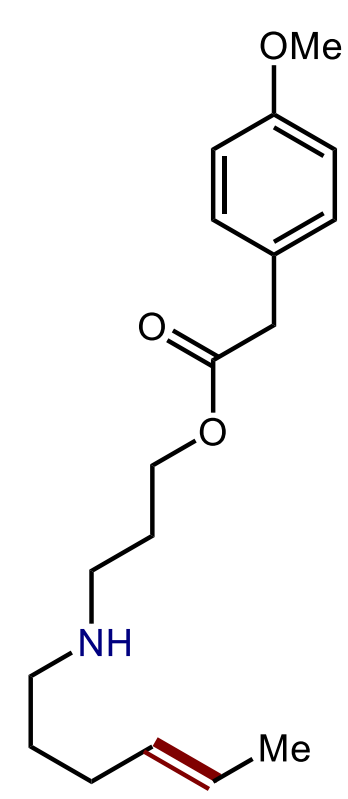

36, 56%, >10:1 rr*

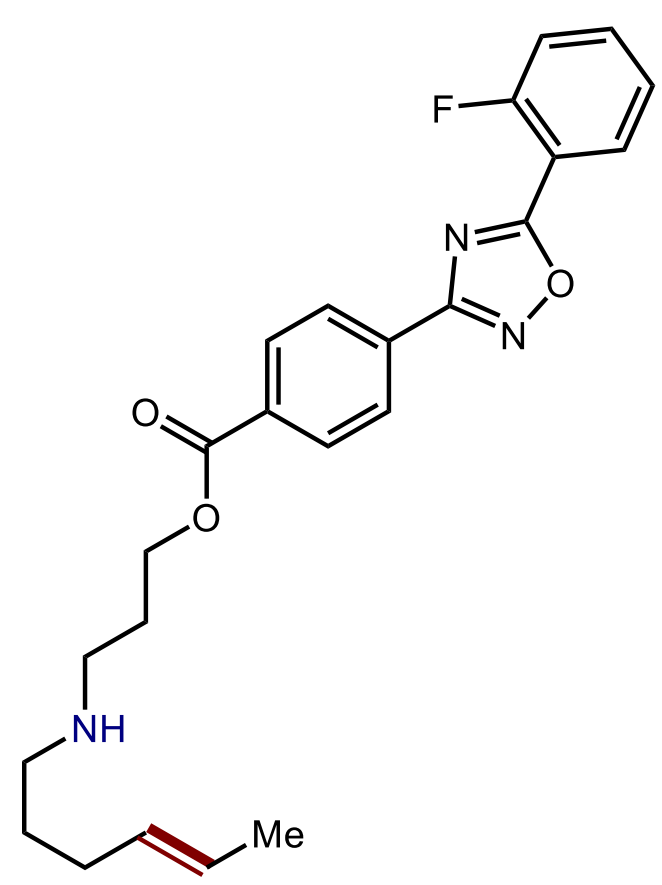

from ataluren
37, 57%, >10:1 rr*

*40°C

Functional Group Tolerance

O-Benzoylhydroxylamine **S1** (0.2 mmol) was subjected to **GP1** with the following modifications:

An **additive** (0.2 mmol, **1 equiv**) was included at the start of the reaction. After 14 h, the amount of product and recovered additive were each determined by NMR with isopropyl acetate as internal standard or GC with *n*-decane as internal standard.

The following additives were subjected to standard reaction conditions to determine the effects of various functional groups on the remote desaturation.

Additives were selected based on expected synthetic utility and potential for further synthetic applications.

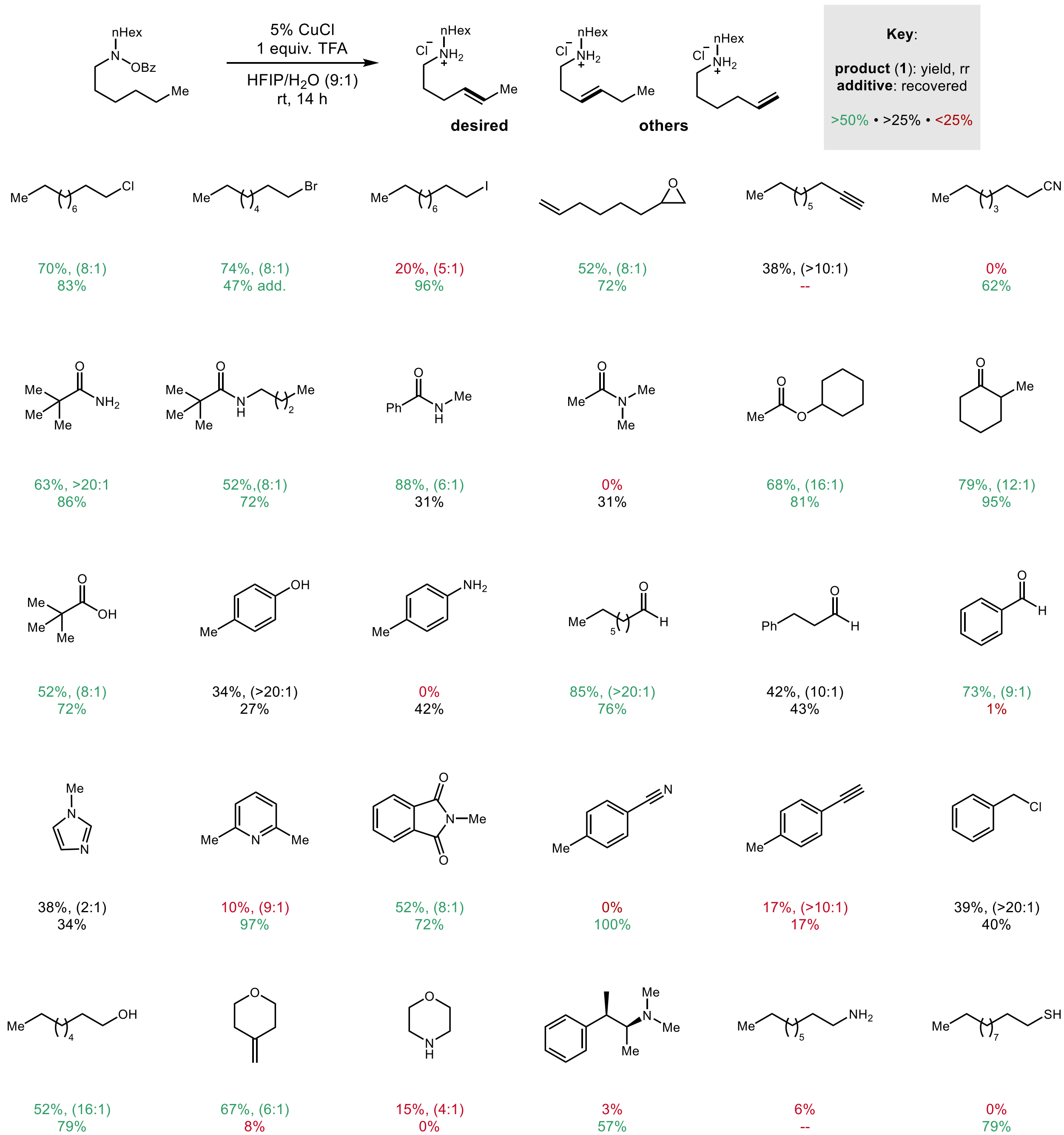

Figure S1: Functional Group Tolerance

IV: Development of δ Desaturation

O-Benzoylhydroxylamine **S1** (0.2 mmol) was subjected to **GP1** with the modifications described in the Tables below. After 14 h, the product yield and regioselectivity ratio (rr) was determined by ^{1}H NMR of the HCl salt.

Table 2: Effect of Acid on δ Desaturation

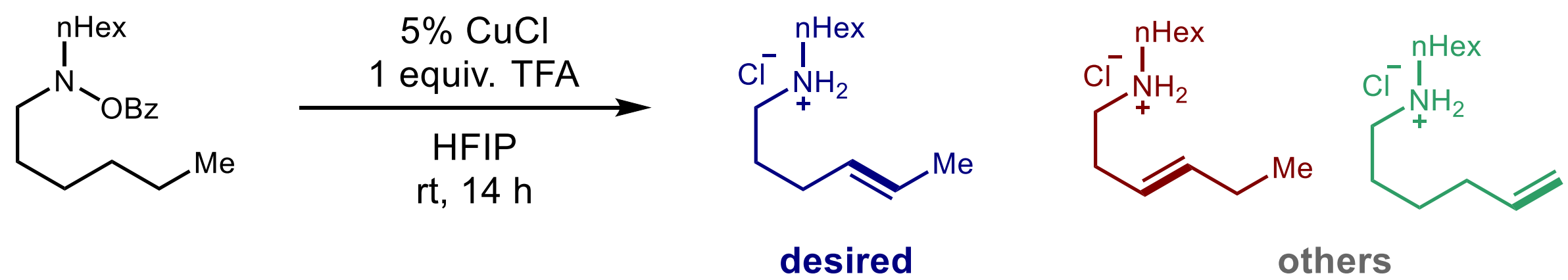

entry	variation from above	% yield (total)	rr (desired:others)	% SM
1	HCl	0	-	92
2	pTsOH	7	>10:1	7
3	AcOH	27	>10:1	2
4	BzOH	46	>10:1	5
5	TFA (0.5 equiv)	30	>10:1	4
6	TFA (1.5 equiv.)	75	>10:1	1
7	none	66	>10:1	3

Table 3: Mechanistic Probes and Reaction Controls

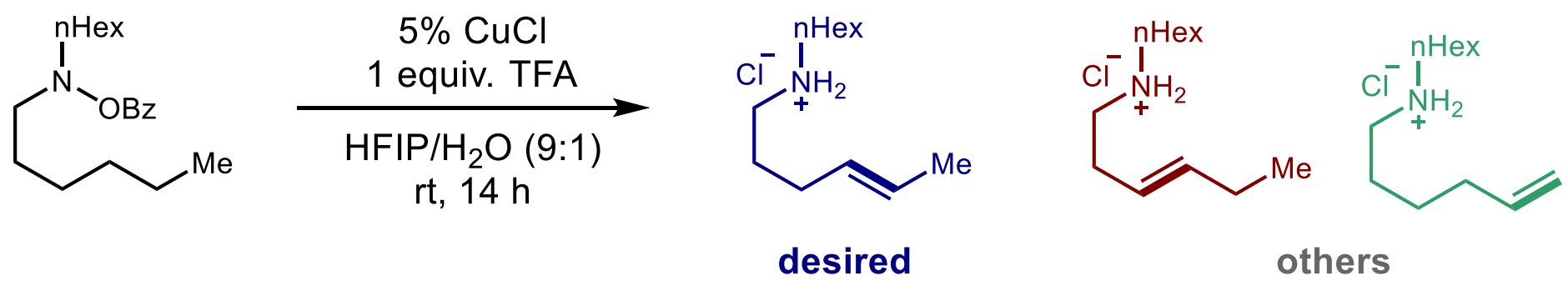

entry	variation from above	% yield (total)	rr (desired:others)	% SM
1	none	92	>10:1	-
2	no copper	-	-	94
3	no TFA	9	-	29
4	+ 1 mL air	-	-	83
5	CuOTf Benzene complex	86	7:1	10
6	CuO	85	9:1	-
7	$CuCl_2$	-	-	89
8	MsOH	35	8:1	49
9	dichloroacetic acid	87	7:1	2
10	AcOH	60	1:2	12
11	2 equiv. TFA	92	7:1	-
12	40°C	76	9:1	-
13	TFE	15	7:1	66
14	THF	-	-	-
15	MeCN	1	-	-
16	4 h	83	>10:1	-
17	24 h	85	10:1	-
18	HFIP only	66	>10:1	3
19	$HFIP/H_2O$ (8:2)	69	>10:1	-

V. Synthesis and Characterization of Starting Materials

O-benzoyl-N,N-dihexylhydroxylamine (S1)

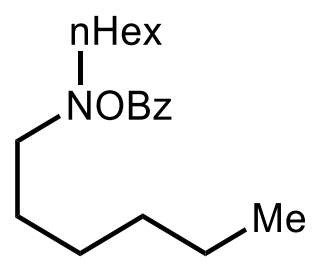

Prepared following **GP2** using dihexylamine (7.0 mL, 30 mmol). Upon completion, the crude mixture was purified on silica gel (5 % EtOAc:Hex) yielding **(S1)** as a colorless oil (5.6 g, 74%). R_f: 0.33 (10% EtOAc:Hex). ^{1}H NMR (400 MHz, $CDCl_3$) δ 8.03 – 8.01 (m, 2H), 7.56 (tt, J = 7.2, 1.2 Hz, 1H), 7.46 – 7.41 (m, 2H), 2.96 – 2.94 (m, 4H), 1.62 – 1.55 (m, 4H), 1.37 – 1.23 (m, 12H), 0.85 (t, J = 6.8 Hz, 6H). ^{13}C NMR (100 MHz, $CDCl_3$) δ 165.6, 133.0, 129.5, 129.4, 128.3, 59.9, 31.6, 26.9, 26.8, 22.5, 14.0. IR (film) cm^{-1}: 1742, 1601, 1450, 1243, 1176, 1082, 706. HRMS (ESI-Orbitrap): *m/z* calculated for $C_{19}H_{32}NO_2$ $[M+H]^+$: 306.2428, found 306.2426.

O-benzoyl-N-hexyl-N-propylhydroxylamine (S2)

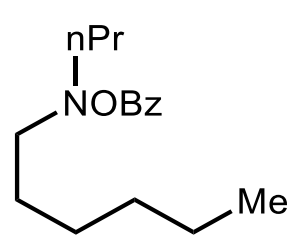

Prepared following **GP2** using N-propylhexan-1-amine (860 mg, 6 mmol). Upon completion, the crude mixture was purified on silica gel (5 % EtOAc:Hex) yielding **(S2)** as a colorless oil (78%). R_f: 0.36 (10% EtOAc:Hex). ^{1}H NMR (400 MHz, $CDCl_3$) δ 8.05 – 7.99 (m, 2H), 7.59 – 7.53 (m, 1H), 7.47 – 7.40 (m, 2H), 3.00 – 2.89 (m, 4H), 1.67 – 1.54 (m, 4H), 1.39 – 1.20 (m, 6H), 0.94 (t, J = 7.4 Hz, 3H), 0.85 (t, J = 6.9 Hz, 3H). ^{13}C NMR (100 MHz, $CDCl_3$) δ 165.8, 133.1, 129.6, 129.5, 128.5, 61.8, 60.0, 31.8, 27.1, 26.9, 22.7, 20.3, 14.1, 11.9. IR (film) cm^{-1}: 1742, 1450, 1246, 1176, 705. HRMS (ESI-Orbitrap): *m/z* calculated for $C_{16}H_{26}NO_2$ $[M+H]^+$: 264.1958, found 264.1957.

O-benzoyl-N-(tert-butyl)-N-hexylhydroxylamine (S3)

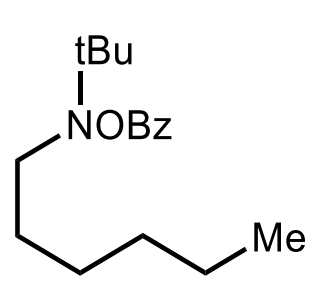

Prepared following **GP2** using N-(tert-butyl)hexan-1-amine (1.99 g, 12.7 mmol). Upon completion, the crude mixture was purified on silica gel (5 % EtOAc:Hex) yielding **(S3)** as a colorless oil (74%). R_f: 0.25 (10% EtOAc:Hex). ^{1}H NMR (400 MHz, $CDCl_3$) δ 8.05 – 8.03 (m, 2H), 7.58 – 7.54 (m, 1H), 7.46 – 7.42 (m, 2H), 2.91 (t, J = 7.6 Hz, 2H), 1.51 – 1.48 (m, 2H), 1.37 – 1.23 (m, 6H), 1.21 (s, 9H), 0.84 (t, J = 7.2 Hz, 3H). ^{13}C NMR (100 MHz, $CDCl_3$) δ 166.2, 133.0, 129.7, 129.0, 128.5, 60.4, 52.0, 31.8, 28.0, 27.2, 25.8, 22.7, 14.1. IR (film) cm^{-1}: 1743, 1450, 1237, 1080, 704. HRMS (ESI-Orbitrap): *m/z* calculated for $C_{17}H_{28}NO_2$ $[M+H]^+$: 278.2115, found 278.2113.

O-benzoyl-N-(heptan-2-yl)-N-propylhydroxylamine (S4)

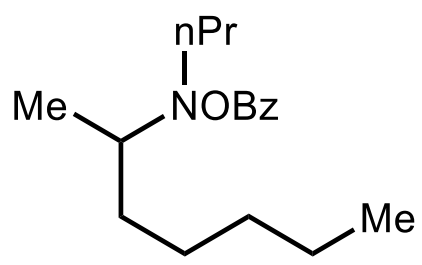

Prepared following **GP2** using N-propylheptan-2-amine (1 g, 7 mmol). Upon completion, the crude mixture was purified on silica gel (5 % EtOAc:Hex) yielding **(S4)** as a colorless oil (48%). R_f: 0.46 (10% EtOAc:Hex). ^{1}H NMR (400 MHz, $CDCl_3$) δ 8.04 – 7.99 (m, 2H), 7.58 – 7.52 (m, 1H), 7.47 – 7.41 (m, 2H), 3.14 – 3.02 (m, 1H), 3.02 – 2.81 (m, 2H), 1.78 – 1.64 (m, 1H), 1.62 – 1.52 (m, 2H), 1.42 – 1.25 (m, 7H), 1.17 (d, J = 6.4 Hz, 3H), 0.94 (t, J = 7.6 Hz, 3H), 0.88 (t, J = 7.1 Hz, 3H). ^{13}C NMR (100 MHz, $CDCl_3$) δ 165.9, 133.0, 129.7, 129.6, 128.5, 62.1, 56.9, 33.2, 28.9, 22.9, 20.7, 14.8, 14.2, 11.9. IR (film) cm^{-1}: 1742, 1601, 1239, 1175, 705. HRMS (ESI-Orbitrap): *m/z* calculated for $C_{17}H_{28}NO_2$ $[M+H]^+$: 278.2115, found 278.2113.

O-benzoyl-N-(2-methylhexyl)-N-propylhydroxylamine (S5)

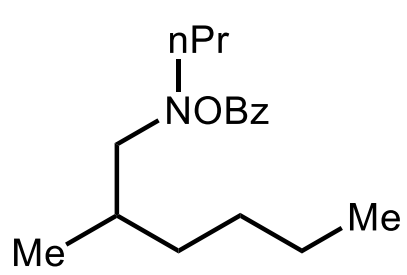

Prepared following **GP2** using 2-methyl-N-propylhexan-1-amine (432 mg, 3 mmol) with the following changes: 1.5 equiv of amine and 2 equiv of K_2HPO_4. Upon completion, the crude mixture was purified on silica gel (5 % EtOAc:Hex) yielding **(S5)** as a colorless oil (42%). R_f: 0.49 (10% EtOAc:Hex). ^{1}H NMR (400 MHz, $CDCl_3$) δ 8.04 – 8.01 (m, 2H), 7.59 – 7.55 (m, 1H), 7.47 – 7.43 (m, 2H), 3.03 – 2.93 (m, 4H), 1.65 – 1.60 (m, 3H), 1.53 – 1.39 (m, 2H), 1.28 – 1.25 (m, 3H), 1.14 – 1.11 (m, 1H), 0.94 (t, J = 7.6 Hz, 3H), 0.88 – 0.83 (m, 6H). ^{13}C NMR (100 MHz, $CDCl_3$) δ 165.5, 133.0, 129.7, 129.6, 128.5, 66.4, 62.2, 37.4, 31.1, 20.2, 20.1, 18.5, 14.4, 11.9. IR (film) cm^{-1}: 1742, 1465, 1239, 1176, 705. HRMS (ESI-Orbitrap): *m/z* calculated for $C_{17}H_{28}NO_2$ $[M+H]^+$: 278.2115, found 278.2113.

O-benzoyl-N-(3-methylhexyl)-N-propylhydroxylamine (S6)

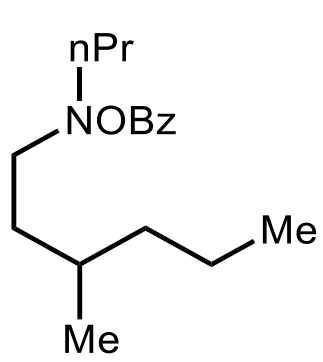

Prepared following **GP2** using 3-methyl-N-propylhexan-1-amine (430 mg, 3 mmol) with the following changes: 1.5 equiv of amine and 2 equiv of K_2HPO_4. Upon completion, the crude mixture was purified on silica gel (5 % EtOAc:Hex) yielding **(S6)** as a colorless oil (55%). R_f: 0.46 (10% EtOAc:Hex). ^{1}H NMR (400 MHz, $CDCl_3$) δ 8.03 – 8.01 (m, 2H), 7.58 – 7.54 (m, 1H), 7.46 – 7.42 (m, 2H), 3.02 – 2.92 (m, 4H), 1.65 – 1.59 (m, 3H), 1.53 – 1.37 (m, 2H), 1.30 – 1.20 (m, 3H), 1.14 – 1.07 (m, 1H), 0.94 (t, J = 7.6 Hz, 3H), 0.89 – 0.86 (m, 6H). ^{13}C NMR (100 MHz, $CDCl_3$) δ 165.8, 133.0, 129.6, 129.5, 128.5, 61.8, 58.1, 33.4, 32.8, 29.6, 20.3, 19.4, 11.9, 11.4. IR

(film) cm^{-1}: 1742, 1601, 1450, 1239, 1175, 1080, 705. HRMS (ESI-Orbitrap): *m/z* calculated for $C_{17}H_{28}NO_2$ $[M+H]^+$: 278.2115, found 278.2113.

O-benzoyl-N-(2-cyclopentylethyl)-N-propylhydroxylamine (S7)

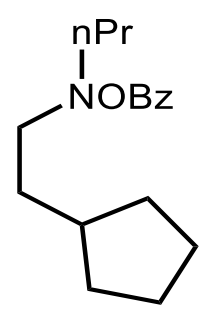

Prepared following **GP2** using N-(2-cyclopentylethyl)propan-1-amine (287 mg, 1.9 mmol). Upon completion, the crude mixture was purified on silica gel (5 % EtOAc:Hex) yielding (**S7**) as a light yellow oil (30%). Rf: 0.29 (5% EtOAc:Hex).).[1] ^{1}H NMR (400 MHz, $CDCl_3$) δ 8.04 – 8.00 (m, 2H), 7.56 (t, *J* = 7.6 Hz, 1H), 7.47 – 7.41 (m, 2H), 3.04 – 2.89 (m, 4H), 1.85 – 1.43 (m, 11H), 1.13-1.02 (m, 2H), 0.94 (t, *J* = 7.4 Hz, 3H). ^{13}C NMR (100 MHz, CDCl3) δ 165.8, 133.1, 129.6, 129.5, 128.5, 61.8, 59.4, 38.2, 33.2, 32.8, 25.8, 20.3, 11.9. IR (film) cm^{-1}: 1742, 1450, 1176, 814. HRMS (ESI-Orbitrap): *m/z* calculated for $C_{17}H_{26}NO_2$ $[M+H]^+$: 276.1958, found 276.1957.

O-benzoyl-N-(2-cyclohexylethyl)-N-propylhydroxylamine (S8)

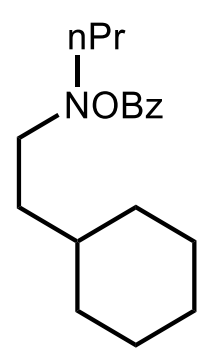

Prepared following **GP2** using N-(2-cyclohexylethyl)propan-1-amine (1.0 g, 6.0 mmol). Upon completion, the crude mixture was purified on silica gel (5 % EtOAc:Hex) yielding (**S8**) as a light yellow oil (42%). Rf: 0.29 (5% EtOAc:Hex). ^{1}H NMR (400 MHz, $CDCl_3$) δ 8.04 – 8.00 (m, 2H), 7.56 (t, *J* = 7.6 Hz, 1H), 7.47 – 7.41 (m, 2H), 3.03 – 2.90 (m, 4H), 1.71 – 1.12 (m, 14H), 0.97- 0.86 (m, 4H). ^{13}C NMR (100 MHz, $CDCl_3$) δ 165.8, 133.1, 129.6, 129.5, 128.5, 61.8, 57.9, 36.1, 34.3, 33.5, 26.6, 26.4, 20.3, 11.9. IR (film) cm^{-1}: 1745, 1449, 1240, 1176, 1118, 707. HRMS (ESI-Orbitrap): *m/z* calculated for $C_{18}H_{28}NO_2$ $[M+H]^+$: 290.2115, found 290.2113.

2-pentylpiperidin-1-yl benzoate (S9)

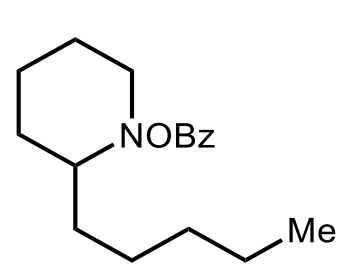

Prepared following **GP2** using 2-pentylpiperidine (379 mg, 2,44 mmol). Upon completion, the crude mixture was purified on silica gel (5 % EtOAc:Hex) yielding (**S9**) as a light yellow oil (29%). Rf: 0.12 (10% EtOAc:Hex). ^{1}H NMR (400 MHz, $CDCl_3$) δ 8.02 (d, *J* = 7.2 Hz, 2H), 7.57 – 7.53 (m, 1H), 7.43 (t, *J* = 7.6 Hz, 2H), 3.56 (d, *J* = 8.8 Hz, 1H), 3.09 – 2.68 (m, 2H), 1.91 – 1.17 (m, 14H), 0.82 (t, *J* = 6.8 Hz, 3H). ^{13}C NMR (100 MHz, $CDCl_3$) δ 165.0, 132.8, 129.6, 129.3, 128.3, 66.9, 58.1, 33.0, 32.0, 30.6, 25.4, 24.9, 23.7, 22.5, 13.9. IR (film) cm^{-1}: 1745, 1240, 1174, 1025, 707. HRMS (ESI-Orbitrap): *m/z* calculated for $C_{17}H_{26}NO_2$ $[M+H]^+$: 276.1958, found 276.1957.

O-benzoyl-N-pentyl-N-propylhydroxylamine (S10)

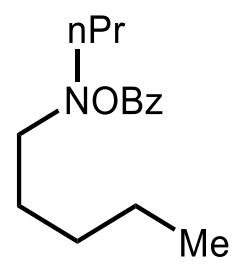

Prepared following **GP2** using N-propylpentan-1-amine (2.58 g, 20 mmol). Upon completion, the crude mixture was purified on silica gel (5 % EtOAc:Hex) yielding **(S10)** as a colorless oil (34%). R_f: 0.40 (10% EtOAc:Hex). ^{1}H NMR (400 MHz, $CDCl_3$) δ 8.05 – 8.00 (m, 2H), 7.58 – 7.53 (m, 1H), 7.48 – 7.40 (m, 2H), 3.01 – 2.89 (m, 4H), 1.68 – 1.54 (m, 4H), 1.37 – 1.24 (m, 4H), 0.94 (t, *J* = 7.6 Hz, 3H), 0.87 (t, *J* = 7.2 Hz, 3H). ^{13}C NMR (100 MHz, $CDCl_3$) δ 165.8, 133.1, 129.6, 129.5, 128.5, 61.7, 60.0, 29.6, 26.6, 22.6, 20.3, 14.1, 11.6. IR (film) cm^{-1}: 1741, 1584, 1239, 1175, 1080, 705. HRMS (ESI-Orbitrap): *m/z* calculated for $C_{15}H_{24}NO_2$ $[M+H]^+$: 250.1802, found 250.1800.

O-benzoyl-N-(2,2-dimethylpentyl)-N-propylhydroxylamine (S11)

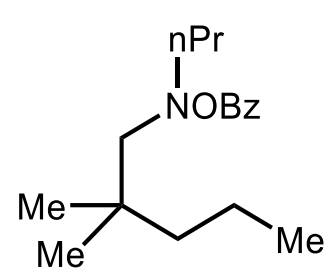

Prepared following **GP2** using 2,2-dimethyl-N-propylpentan-1-amine (1.9 g, 12 mmol). Upon completion, the crude mixture was purified on silica gel (5 % EtOAc:Hex) yielding **(S11)** as a colorless oil (33%). R_f: 0.60 (10% EtOAc:Hex). ^{1}H NMR (400 MHz, $CDCl_3$) δ 8.02 – 7.97 (m, 2H), 7.57 – 7.51 (m, 1H), 7.46 – 7.39 (m, 2H), 2.94 (t, *J* = 7.5 Hz, 2H), 2.78 (s, 2H), 1.57 (sext, *J* = 7.6 Hz, 2H), 1.31 – 1.20 (m, 4H), 0.96 (t, *J* = 7.6 Hz, 3H), 0.90 (s, 6H), 0.83 – 0.77 (m, 3H). ^{13}C NMR (100 MHz, $CDCl_3$) δ 165.3, 132.9, 130.0, 129.5, 128.5, 68.8, 63.6, 43.4, 34.5, 26.2, 20.0, 17.2, 15.0, 11.8. IR (film) cm^{-1}: 1714, 1466, 1255, 1024, 705. HRMS (ESI-Orbitrap): *m/z* calculated for $C_{17}H_{28}NO_2$ $[M+H]^+$: 278.2115, found 278.2113.

***O*-benzoyl-*N*-hexylhydroxylamine (S12)**

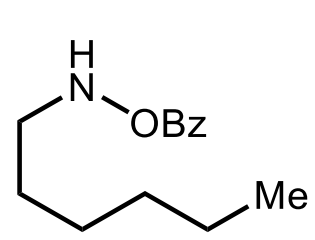

Prepared following **GP2** using hexylamine (2.0 mL, 15.0 mmol). Upon completion, the crude mixture was purified on silica gel (7 % EtOAc:Hex) yielding **(S12)** as a colorless oil (45%). R_f: 0.54 (10% EtOAc:Hex). ^{1}H NMR (400 MHz, $CDCl_3$) δ 8.04 – 7.98 (m, 2H), 7.58 – 7.53 (m, 1H), 7.46 – 7.40 (m, 2H), 3.12 (t, *J* = 7.2 Hz, 2H), 1.60 (pent, *J* = 7.2 Hz, 2H), 1.43 – 1.25 (m, 7H), 0.88 (t, *J* = 6.8 Hz, 3H). ^{13}C NMR (100 MHz, $CDCl_3$) δ 167.0, 133.3, 129.4, 128.6, 128.6, 52.7, 31.7, 27.2, 26.8, 22.6, 14.1. IR (film) cm^{-1}: 3232, 1717, 1584, 1268, 1143, 796. HRMS (ESI-Orbitrap): *m/z* calculated for $C_{13}H_{20}NO_2$ $[M+H]^+$: 222.1489, found 222.1488.

***O*-benzoyl-*N*-(heptan-2-yl)hydroxylamine (S13)**

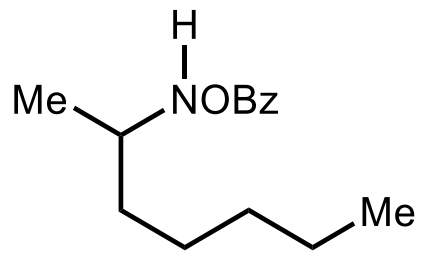

Prepared following **GP2** using heptan-2-amine (0.75 mL, 5.0 mmol). Upon completion, the crude mixture was purified on silica gel (5 % EtOAc:Hex) yielding (**S13**) as a light yellow oil (64%). Rf: 0.25 (10% EtOAc:Hex). ^{1}H NMR (400 MHz, $CDCl_3$) δ 8.03 – 8.00 (m, 2H), 7.77 (br s, 1H), 7.59 – 7.55 (m, 1H), 7.47 – 7.43 (m, 2H), 3.23 – 3.16 (m, 1H), 1.65 – 1.55 (m, 1H), 1.41 – 1.29 (m, 7H), 1.24 (d, *J* = 4.4 Hz, 3H), 0.89 (t, *J* = 6.8 Hz, 3H). ^{13}C NMR (100 MHz, $CDCl_3$) δ167.1, 133.4, 129.4, 128.7, 128.6, 56.9, 34.1, 32.0, 25.7, 22.7, 18.2, 14.1. IR (film) cm^{-1}: 3380, 1714, 1584, 1266, 1177, 1144, 794, 704. HRMS (ESI-Orbitrap): *m/z* calculated for $C_{14}H_{22}NO_2$ $[M+H]^+$: 236.1645, found 236.1644.

***O*-benzoyl-*N*-(undecan-6-yl)hydroxylamine (S14)**

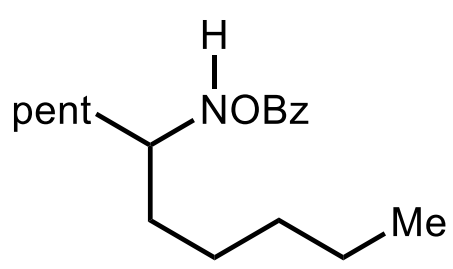

Prepared following **GP2** using undecan-6-amine (1.07 mL, 5.0 mmol). Upon completion, the crude mixture was purified on silica gel (5 % EtOAc:Hex) yielding (**S14**) as a light yellow oil (67%). Rf: 0.38 (10% EtOAc:Hex). ^{1}H NMR (400 MHz, CDCl3) δ 8.03 – 8.0 (m, 2H), 7.86 (br s, 1H), 7.60 – 7.56 (m, 1H), 7.46 (t, *J* = 8.0 Hz, 2H), 3.04 (quint, *J* = 5.6 Hz, 1H), 1.59 – 1.37 (m, 8H), 1.36 – 1.29 (m, 8H), 0.89 (t, *J* = 6.8 Hz, 6H). ^{13}C NMR (100 MHz, CDCl3) δ 167.0, 133.3, 129.4, 128.7, 128.6, 61.4, 32.2, 32.1, 25.6, 22.7, 14.1. IR (film) cm^{-1}: 3325, 1717, 1584, 1267, 1177, 1090, 705. HRMS (ESI-Orbitrap): *m/z* calculated for $C_{18}H_{30}NO_2$ $[M+H]^+$: 292.2271, found 292.2270.

***O*-benzoyl-*N*-(((1*S*,4a*R*,10a*S*)-7-isopropyl-1,4a-dimethyl-1,2,3,4,4a,9,10,10a-octahydrophenanthren-1-yl)methyl)hydroxylamine (S15)**

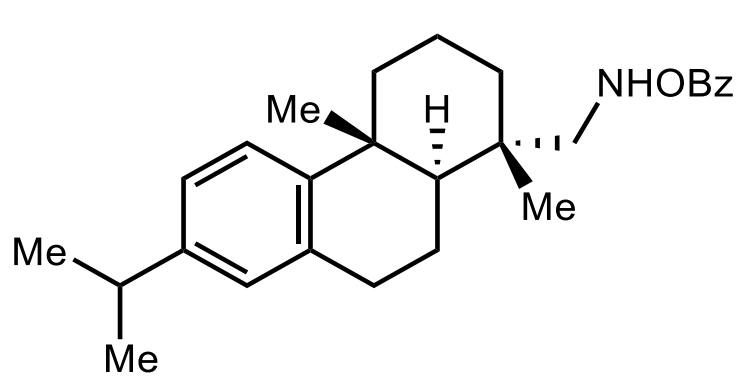

Prepared following **GP2** using ((1S,4aR,10aS)-7-isopropyl-1,4a-dimethyl-1,2,3,4,4a,9,10,10a-octahydrophenanthren-1-yl)methanamine (1.4 g, 5.0 mmol). Upon completion, the crude mixture was purified on silica gel (5 % EtOAc:Hex) yielding (**S15**) as a light yellow oil (75%). Rf: 0.15 (10% EtOAc:Hex). ^{1}H NMR (400 MHz, $CDCl_3$) δ 8.03 – 8.01 (m, 2H), 7.62 – 7.57 (m, 1H), 7.49 – 7.44 (m, 2H), 7.20 (d, *J* = 8.4 Hz, 1H), 7.01 (dd, *J* = 8.0, 1.6 Hz, 1H), 6.91 – 6.90 (m, 1H), 6.56 (br s, 1H), 3.01 (q, *J* = 12.0 Hz, 2H), 2.96 – 2.92 (m, 2H), 2.89 – 2.79 (m, 1H), 2.32 (dt, *J* = 12.8,

3.2 Hz, 1H), 1.90 – 1.69 (m, 4H), 1.65 – 1.62 (m, 3H), 1.46 (td, J = 12.8, 3.6 Hz, 1H), 1.25 (s, 3H), 1.24 (d, J = 6.8 Hz, 6H), 1.07 (s, 3H). ^{13}C NMR (100 MHz, $CDCl_3$)167.1, 147.1, 145.5, 134.6, 133.3, 129.2, 128.4, 128.3, 126.7, 124.1, 123.8, 63.3, 45.9, 38.2, 37.4, 37.2, 36.5, 33.4, 30.0, 25.3, 23.93, 23.90, 19.0, 18.7. IR (film) cm^{-1}: 3248, 1718, 1584, 1266, 1243, 795, 706. HRMS (ESI-Orbitrap): *m/z* calculated for $C_{27}H_{36}NO_2$ $[M+H]^+$: 406.2741, found 406.2739.

Methyl 2-((benzoyloxy)amino)heptanoate (S16)

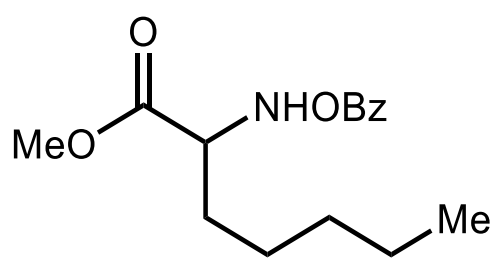

Prepared following **GP2** using methyl 2-aminoheptanoate (318 mg, 2.0 mmol). Upon completion, the crude mixture was purified on silica gel (5 % EtOAc:Hex) yielding (**S16**) as a light yellow oil (35%). Rf: 0.10 (10% EtOAc:Hex). ^{1}H NMR (400 MHz, $CDCl_3$) δ 8.06 (br s, 1H),7.97 – 7.95 (m, 2H), 7.59 – 7.54 (m, 1H), 7.43 (t, J = 7.6 Hz, 2H), 3.94 (t, J = 6.8 Hz, 1H), 3.73 (s, 3H), 1.78 – 1.72 (m, 2H), 1.56 – 1.40 (m, 2H), 1.35 – 1.30 (m, 4H), 0.89 (t, J = 6.8 Hz, 3H). ^{13}C NMR (100 MHz, $CDCl_3$) 173.1, 165.7, 133.3, 129.3, 128.4, 128.1, 63.3, 52.2, 31.4, 29.4, 25.5, 22.3, 13.9. IR (film) cm^{-1}: 3391, 1727, 1583, 1266, 1204, 1091, 935, 735, 705. HRMS (ESI-Orbitrap): *m/z* calculated for $C_{15}H_{22}NO_4$ $[M+H]^+$: 280.1543, found 280.1542.

O-benzoyl-N-(pent-4-en-1-yl)-N-propylhydroxylamine (S17)

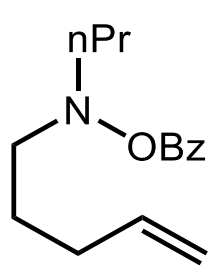

Prepared following **GP2** using N-propylpent-4-en-1-amine (550 mg, 4.32 mmol). Upon completion, the crude mixture was purified on silica gel (5 % EtOAc:Hex) yielding (**S17**) as a light yellow oil (74%). Rf: 0.10 (10% EtOAc:Hex). ^{1}H NMR (400 MHz, CDCl3) δ 8.03 – 8.01 (m, 2H), 7.58 – 7.54 (m, 1H), 7.46 – 7.42 (m, 2H), 5.83 – 5.73 (m, 1H), 5.03 – 4.93 (m, 2H), 3.00 – 2.92 (m, 4H), 2.16 – 2.10 (m, 2H), 1.74 – 1.66 (m, 2H), 1.64 – 1.57 (m, 2H), 0.94 (t, J = 7.2 Hz, 3H).^{13}C NMR (100 MHz, $CDCl_3$) 165.7, 138.2, 133.1, 129.6, 129.4, 128.5, 115.1, 61.8, 59.2, 31.4, 26.1, 20.3, 11.8. IR (film) cm^{-1}: 2934, 1742, 1450, 1240, 1176, 993, 813, 706. HRMS (ESI-Orbitrap): *m/z* calculated for $C_{15}H_{22}NO_2$ $[M+H]^+$: 248.1651, found 248.1644.

O-benzoyl-N-(3,7-dimethyloctyl)-N-propylhydroxylamine (S18)

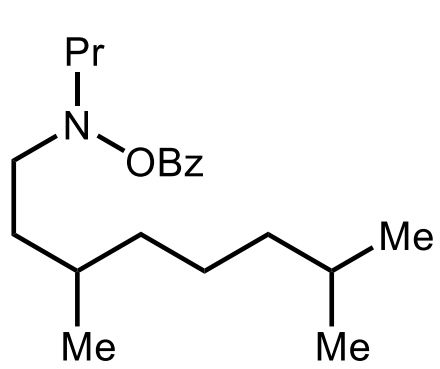

Prepared following **GP2** using 3,7-dimethyl-N-propyloctan-1-amine (369 mg, 1.85 mmol). Upon completion, the crude mixture was purified on silica gel (5 % EtOAc:Hex) yielding (**S18**) as a light

yellow oil (31%). Rf: 0.57 (10% EtOAc:Hex). ^{1}H NMR (400 MHz, $CDCl_3$) δ 8.03 – 8.00 (m, 2H), 7.57 – 7.52 (m, 1H), 7.45 – 7.40 (m, 2H), 3.01 – 2.90 (m, 4H), 1.64 – 1.58 (m, 3H), 1.50 – 1.41 (m, 3H), 1.26 – 1.21 (m, 3H), 1.11 – 1.07 (m, 3H), 0.93 (t, *J* = 7.2 Hz, 3H), 0.86 (d, *J* = 6.4 Hz, 3H), 0.81 (d, *J* = 6.4 Hz, 6H).^{13}C NMR (100 MHz, $CDCl_3$) 165.8, 133.1, 129.6, 128.5, 61.8, 58.1, 39.3, 37.3, 33.8, 31.2, 28.0, 24.7, 22.8, 22.7, 20.3, 19.8, 11.8. IR (film) cm^{-1}: 1743, 1602, 1451, 1240, 1176, 1080, 686. HRMS (ESI-Orbitrap): *m/z* calculated for $C_{20}H_{34}NO_2$ $[M+H]^+$: 320.2584, found 320.2583.

O-benzoyl-N-(oct-1-en-3-yl)hydroxylamine (S19)

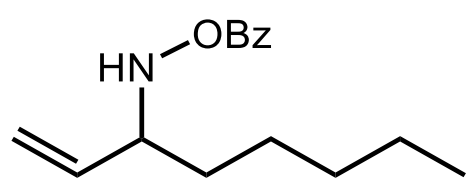

Prepared following **GP2** using oct-1-en-3-amine (322 mg, 2.53 mmol). Upon completion, the crude mixture was purified on silica gel (5 % EtOAc:Hex) yielding (**S19**) as a light yellow oil (45%). Rf: 0.10 (10% EtOAc:Hex). 1H NMR (400 MHz, CDCl3) δ 8.01 – 7.99 (m, 2H), 7.77 (br s, 1H), 7.60 – 7.56 (m, 1H), 7.47 – 7.43 (m, 2H), 5.79 – 5.70 (m, 1H), 5.25 – 5.17 (m, 2H), 3.59 (q, *J* = 7.2 Hz, 1H), 1.73 – 1.64 (m, 1H), 1.56 – 1.48 (m, 2H), 1.42 – 1.29 (m, 6H), 0.88 (t, *J* = 5.1 Hz, 3H).^{13}C NMR (100 MHz, $CDCl_3$) 167.1, 137.7, 133.5, 129.5, 128.7, 128.6, 118.4, 65.0, 31.9, 25.6, 22.7, 14.2. IR (film) cm^{-1}: 2942, 1631, 1441, 1375, 1038, 918, 748. HRMS (ESI-Orbitrap): *m/z* calculated for $C_{15}H_{22}NO_2$ $[M+H]^+$: 248.1651, found 248.1646.

O-Benzoyl-N,N-dipropylhydroxylamine (S20)

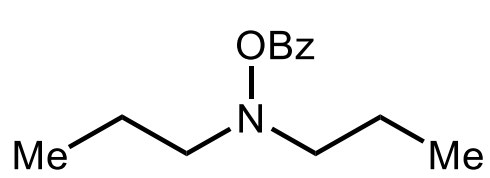

Prepared following **GP2** using dipropylamine (3.3 mL, 20 mmol). Upon completion, the crude mixture was purified on silica gel (5 % EtOAc:Hex) yielding as a colorless oil (3.2 g, 73%). ^{1}H NMR (400 MHz, $CDCl_3$) δ 8.01 – 7.99 (m, 2H), 7.53 (tt, *J* = 5.4, 0.9 Hz, 1H), 7.43 – 7.38 (m, 2H), 2.93 – 2.89 (m, 4H), 1.60 (quint, *J* = 5.6 Hz, 4H), 0.92 (t, *J* = 5.2 Hz, 6H). ^{13}C NMR (100 MHz, $CDCl_3$) δ 165.7, 133.0, 129.5, 129.4, 128.4, 61.7, 20.2, 11.7. IR (film) cm^{-1}: 1741, 1450, 1238, 1059, 706. HRMS (ESI-Orbitrap): *m/z* calculated for $C_{13}H_{20}NO_2$ $[M+H]^+$: 222.1489, found 222.1488.

O-benzoyl-N-benzyl-N-hexylhydroxylamine (S21)

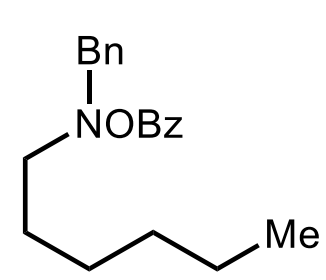

Prepared following **GP2** using N-benzylhexan-1-amine (693 mg, 3.6 mmol). Upon completion, the crude mixture was purified on silica gel (5 % EtOAc:Hex) yielding (**S21**) as a light yellow oil (83%). Rf: 0.43 (10% EtOAc:Hex). ^{1}H NMR (400 MHz, $CDCl_3$) δ 7.94 – 7.91 (m, 2H), 7.54 – 7.50 (m, 1H), 7.42 – 7.37 (m, 4H), 7.31 – 7.22 (m, 3H), 4.17 (s, 2H), 3.02 – 2.98 (m, 2H), 1.65 – 1.58 (m, 2H), 1.38 – 1.21 (m, 6H), 0.85 (t, *J* = 6.8 Hz, 3H).^{13}C NMR (100 MHz, $CDCl_3$) 165.3, 135.9, 132.9, 129.7, 129.5, 129.4, 128.41, 128.35, 127.7, 63.6, 58.6, 31.7, 26.9, 22.6, 14.1. IR

(film) cm^{-1}: 2929, 1740, 1601, 1451, 1241, 1176, 1081, 697. HRMS (ESI-Orbitrap): *m/z* calculated for $C_{20}H_{26}NO_2$ $[M+H]^+$: 312.1958, found 312.1957.

3-((benzoyloxy)(pentyl)amino)propanenitrile (S22)

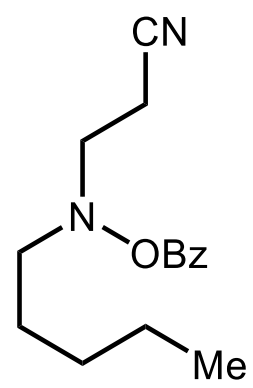

Prepared following **GP2** using 3-(pentylamino)propanenitrile (463 μL, 2.5 mmol). Upon completion, the crude mixture was purified on silica gel (5 % EtOAc:Hex) yielding (**S22**) as a light yellow oil (59 %). Rf: 0.10 (20% EtOAc:Hex). ^{1}H NMR (400 MHz, $CDCl_3$) δ 8.02 – 8.00 (m, 2H), 7.60 – 7.56 (m, 1H), 7.47 – 7.43 (m, 2H), 3.30 (t, *J* = 7.2 Hz, 2H), 3.02 (dd, *J* = 7.2, 9.2 Hz, 2H), 2.67 (t, *J* = 7.2 Hz, 2H), 1.69 – 1.55 (m, 2H), 1.37 – 1.26 (m, 4H), 0.86 (t, *J* = 6.8 Hz, 3H).^{13}C NMR (100 MHz, $CDCl_3$) 165.5, 133.6, 129.7, 128.6, 128.5, 118.1, 59.7, 54.5, 29.2, 26.3, 22.4, 16.0, 14.0. IR (film) cm^{-1}: 2955, 2250, 1741, 1601, 1450, 1240, 1177, 1081, 706. HRMS (ESI-Orbitrap): *m/z* calculated for $C_{15}H_{21}N_2O_2$ $[M+H]^+$: 261.1598, found 261.1597.

3-((benzoyloxy)(hexyl)amino)propan-1-ol (S23)

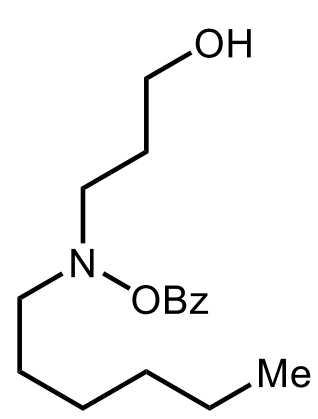

Prepared following **GP2** using 3-(hexylamino)propan-1-ol (795 mg, 5 mmol). Upon completion, the crude mixture was purified on silica gel (30 % EtOAc:Hex) yielding (**S21**) as a yellow oil (38%). Rf: 0.18 (40% EtOAc:Hex). ^{1}H NMR (400 MHz, $CDCl_3$) δ 8.02 – 8.00 (m, 2H), 7.59 – 7.55 (m, 1H), 7.46 – 7.42 (m, 2H), 3.81 (t, *J* = 5.6 Hz, 2H), 3.31 (br s, 1H), 3.18 – 3.15 (m, 2H), 3.00 – 2.96 (m, 2H), 1.79 – 1.76 (m, 2H), 1.61 – 1.53 (m, 2H), 1.37 – 1.33 (m, 2H), 1.26 – 1.25 (m, 4H), 0.85 (t, *J* = 6.8 Hz, 3H).^{13}C NMR (100 MHz, $CDCl_3$) 166.0, 133.3, 129.6, 129.0, 128.6, 62.3, 59.4, 58.4, 31.7, 29.3, 26.90, 26.89, 22.6, 14.1. IR (film) cm^{-1}: 3305, 2928, 1722, 1596, 1247, 672. HRMS (ESI-Orbitrap): *m/z* calculated for $C_{16}H_{26}NO_3$ $[M+H]^+$: 280.1907, found 280.1908.

3-((benzoyloxy)(hexyl)amino)propyl benzoate (S24)

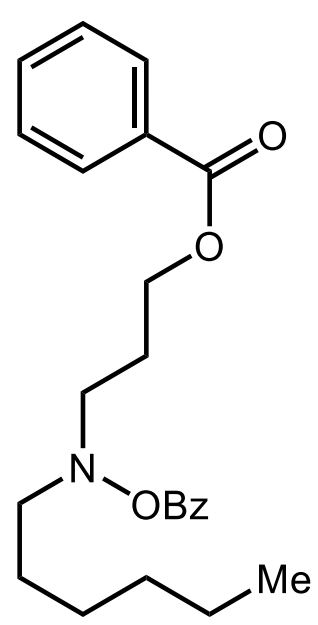

Prepared following **GP5** using benzoyl chloride (222 μL, 1.5 mmol). Upon completion, the crude mixture was purified on silica gel (15 % EtOAc:Hex) yielding (**S23**) as a light yellow oil (38%). Rf: 0.41 (20% EtOAc:Hex). ^{1}H NMR (400 MHz, $CDCl_3$) δ 8.04 – 8.01 (m, 4H), 7.59 – 7.53 (m, 2H), 7.47 – 7.41 (m, 4H), 4.43 (t, J = 6.4 Hz, 2H), 3.17 – 3.14 (m, 2H), 3.03 – 2.99 (m, 2H), 2.09 – 2.06 (m, 2H), 1.62 – 1.56 (m, 2H), 1.39 – 1.32 (m, 2H), 1.28 – 1.25 (m, 4H), 0.85 (t, J = 7.2 Hz, 3H).^{13}C NMR (100 MHz, $CDCl_3$)166.6, 165.7, 133.2, 133.0, 130.4, 129.7, 129.6, 129.2, 128.6, 128.5, 63.0, 60.2, 56.5, 31.7, 27.0, 26.9, 26.7, 22.6, 14.1. IR (film) cm^{-1}: 2929, 1717, 1602, 1451, 1243, 1175, 1082, 686. HRMS (ESI-Orbitrap): *m/z* calculated for $C_{23}H_{30}NO_4$ [M+H]$^+$: 384.2169, found 384.2169.

3-((benzoyloxy)(hexyl)amino)propyl 4-(trifluoromethyl)benzoate (S25)

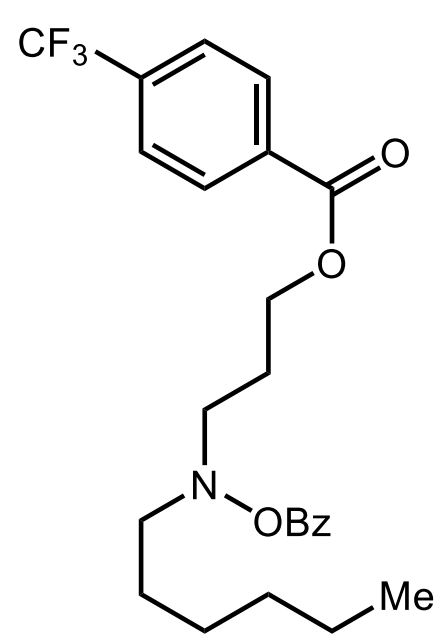

Prepared following **GP5** using 4-(trifluoromethyl)benzoyl chloride (222 μL, 1.5 mmol). Upon completion, the crude mixture was purified on silica gel (15 % EtOAc:Hex) yielding (**S24**) as a light yellow oil (41%). Rf: 0.41 (20% EtOAc:Hex). ^{1}H NMR (400 MHz, $CDCl_3$) δ 8.14 – 8.11 (m, 2H), 8.04 – 8.01 (m, 2H), 7.70 – 7.68 (m, 2H), 7.60 – 7.56 (m, 1H), 7.47 – 7.43 (m, 2H), 4.48 (t, J = 6.4 Hz, 2H), 3.17 – 3.13 (m, 2H), 3.03 – 2.99 (m, 2H), 2.11 – 2.05 (m, 2H), 1.63 – 1.56 (m, 2H), 1.37 – 1.33 (m, 2H), 1.27 – 1.25 (m, 4H), 0.85 (t, J = 7.2 Hz, 3H).^{13}C NMR (100 MHz, $CDCl_3$) 165.7, 165.4, 134.4 (q, J = 33 Hz), 133.6, 133.3, 130.1, 129.7, 128.6, 125.5 (q, J = 4 Hz), 123.8 (q, J = 271 Hz), 63.6, 60.2, 56.3, 31.7, 27.0, 26.9, 26.6, 22.6, 14.1.^{19}F (377 MHz, $CDCl_3$) δ -63.1. IR (film) cm^{-1}: 2931, 1723, 1585, 1274, 704, 669. HRMS (ESI-Orbitrap): *m/z* calculated for $C_{24}H_{29}F_3NO_4$ [M+H]$^+$: 452.2043, found 452.2044.

3-((benzoyloxy)(hexyl)amino)propyl 2-(4-methoxyphenyl)acetate (S27)

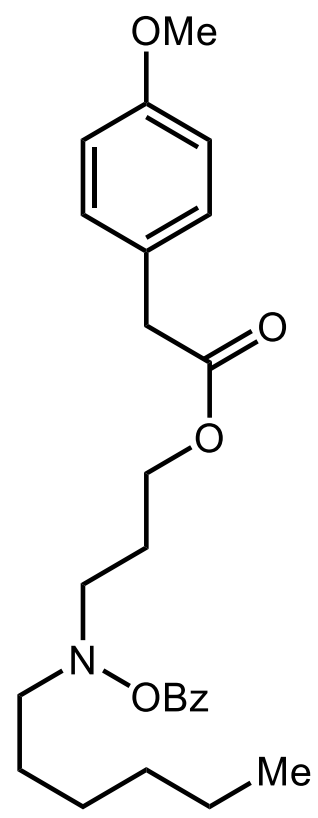

Prepared following **GP5** using 2-(4-methoxyphenyl)acetyl chloride (229 µL, 1.5 mmol). Upon completion, the crude mixture was purified on silica gel (20 % EtOAc:Hex) yielding (**S25**) as a light yellow oil (43%). Rf: 0.14 (20% EtOAc:Hex). ^{1}H NMR (400 MHz, $CDCl_3$) δ 8.02 – 7.99 (m, 2H), 7.59 – 7.55 (m, 1H), 7.45 – 7.43 (m, 2H), 7.17 (d, *J* = 8.8 Hz, 2H), 6.83 (d, *J* = 8.8 Hz, 2H), 4.19 (t, *J* = 6.0 Hz, 2H), 3.78 (s, 3H), 3.53 (s, 2H), 3.00 – 2.96 (m, 2H), 2.96 – 2.92 (m, 2H), 1.94 – 1.87 (m, 2H), 1.59 – 1.52 (m, 2H), 1.35 – 1.33 (m, 2H), 1.28 – 1.25 (m, 4H), 0.86 (t, *J* = 6.8 Hz, 3H).^{13}C NMR (100 MHz, $CDCl_3$) 172.0, 165.7, 158.8, 133.2, 130.4, 129.6, 129.2, 128.6, 126.3, 114.1, 62.9, 60.1, 56.3, 55.4, 40.6, 31.7, 27.0, 26.8, 26.4, 22.6, 14.1. IR (film) cm^{-1}: 2931, 1732, 1511, 1243, 1082, 709, 687. HRMS (ESI-Orbitrap): *m/z* calculated for $C_{25}H_{34}NO_5$ $[M+H]^+$: 428.2431, found 428.2432.

3-((benzoyloxy)(hexyl)amino)propyl 4-(5-(2-fluorophenyl)-1,2,4-oxadiazol-3-yl)benzoate (S28)

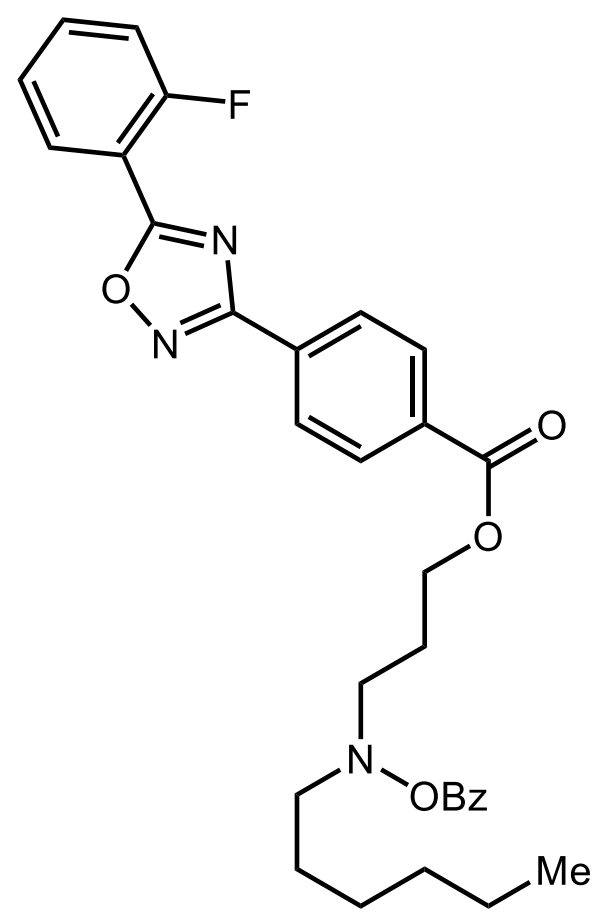

Prepared following **GP5** using 4-(5-(2-fluorophenyl)-1,2,4-oxadiazol-3-yl)benzoyl chloride (453 mg, 1.5 mmol). Upon completion, the crude mixture was purified on silica gel (30 % EtOAc:Hex) yielding (**S26**) as a light yellow oil (23%). Rf: 0.55 (40% EtOAc:Hex). ^{1}H NMR (400 MHz, $CDCl_3$) δ 8.82– 8.81 (m, 1H), 8.38 – 8.36 (m, 1H), 8.27 – 8.23 (m, 1H), 8.19 – 8.17 (m, 1H), 8.05 – 8.03 (m, 2H), 7.61 – 7.57 (m, 3H), 7.46 – 7.42 (m, 2H), 7.38 – 7.32 (m, 1H), 7.32 – 7.27 (m, 1H), 4.50 (t, *J* = 6.4 Hz, 2H), 3.21 – 3.17 (m, 2H), 3.05 – 3.01 (m, 2H), 2.15 – 2.08 (m, 2H), 1.65 – 1.57 (m, 2H), 1.39 – 1.31 (m, 2H), 1.27 – 1.23 (m, 4H), 0.84 (t, *J* = 6.8 Hz, 3H).^{13}C NMR (100 MHz, $CDCl_3$) δ 173.17, 168.18, 165.90, 165.76, 160.93 (d, *J* = 259 Hz), 134.86 (d, *J* = 8.6 Hz), 133.49, 133.24, 132.35, 131.87, 131.21 (d, *J* = 10.8 Hz), 129.66, 129.18, 128.55, 127.34, 124.88 (d, *J* = 3 Hz), 117.30 (d, *J* = 20.7 Hz), 112.82 (d, *J* = 11 Hz), 63.44, 60.22, 56.41, 31.71, 27.01, 26.89, 26.68, 22.62, 14.09. ^{19}F (377 MHz, $CDCl_3$) δ -108.2. IR (film) cm^{-1}: 2929, 1723, 1556, 1368, 1255, 1063, 750, 709. HRMS (ESI-Orbitrap): *m/z* calculated for $C_{31}H_{33}FN_3O_5$ $[M+H]^+$: 546.2399, found 546.2397.

VI. δ Desaturated Amine Product Characterization

(E)-N-hexylhex-4-en-1-aminium (1)

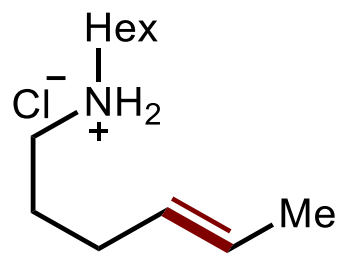

Prepared following **GP1** with **S1**. The crude product (92%, >10:1 r.r., E:Z 1.3:1) was recrystallized with EtOAc to afford a white solid. ^{1}H NMR (400 MHz, $CDCl_3$) 9.47 (br s, 2H), 5.49 – 5.44 (m, 1H), 5.35 – 5.29 (m, 1H), 2.89 – 2.88 (m, 4H), 2.15 – 1.84 (m, 6H), 1.62 – 1.57 (m, 3H), 1.36 – 1.26 (m, 6H), 0.86 (t, *J* = 6.8 Hz, 3H). ^{13}C NMR (100 MHz, $CDCl_3$) δ: 128.9, 127.0, 48.0, 47.4, 31.3, 29.8, 26.6, 25.9, 25.6, 22.6, 17.9, 14.0. IR (film) cm^{-1}: 3390, 1590, 1245, 1144, 968, 895, 702. HRMS (ESI-Orbitrap): *m/z* calculated for $C_{12}H_{26}N$ $[M]^+$: 184.2060, found 184.2060.

(E)-N-propylhex-4-en-1-aminium (2)

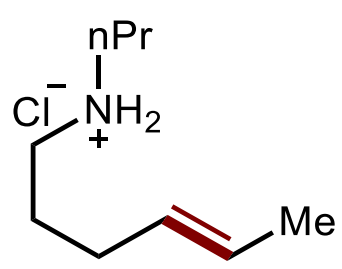

Prepared following **GP1** with **S2**. The crude product (79%, 9:1 r.r., E:Z 1.4:1) was recrystallized with EtOAc to afford a white solid. ^{1}H NMR (400 MHz, $CDCl_3$) δ 9.41 (br s, 2H), 5.49 – 5.37 (m, 1H), 5.32 – 5.23 (m, 1H), 2.85 – 2.80 (m, 4H), 2.11 – 1.81 (m, 6H), 1.57 – 1.52 (m, 3H), 0.95 – 0.92 (m, 3H). ^{13}C NMR (100 MHz, $CDCl_3$) δ: 128.9, 127.0, 49.5, 47.4, 29.7, 25.6, 19.5, 17.9, 11.4. IR (film) cm^{-1}: 3395, 1591, 1265, 1144, 969, 896, 731, 700. HRMS (ESI-Orbitrap): *m/z* calculated for $C_9H_{20}N$ $[M]^+$: 142.1590, found 142.1590.

(E)-N-(tert-butyl)hex-4-en-1-aminium (3)

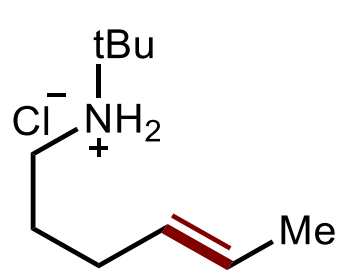

Prepared following **GP1** with **S3**. The crude product (73%, 9:1 r.r., E:Z 1.9:1) was recrystallized with EtOAc to afford a white solid. ^{1}H NMR (400 MHz, $CDCl_3$) δ 9.17 (br s, 2H), 5.45 – 5.36 (m, 1H), 5.32 – 5.25 (m, 1H), 2.78 (br s, 2H), 2.07 – 1.93 (m, 4H), 1.57 – 1.52 (m, 3H), 1.41 (br s, 9H). ^{13}C NMR (100 MHz, $CDCl_3$) δ: 129.1, 126.8, 57.1, 41.6, 30.1, 26.4, 26.1, 18.0. IR (film) cm^{-1}: 3360, 1591, 1265, 1205, 968, 731, 700. HRMS (ESI-Orbitrap): *m/z* calculated for $C_{10}H_{22}N$ $[M]^+$: 156.1747, found 156.1747.

(E)-N-propylhept-5-en-2-aminium (4)

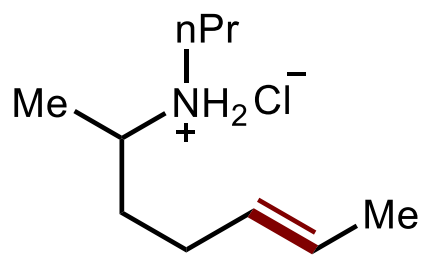

Prepared following **GP1** with **S4**. The crude product (61%, >10:1 r.r., E:Z 4:1) was recrystallized with EtOAc to afford a white solid. ^{1}H NMR (400 MHz, $CDCl_3$) δ 9.38 (s, 2H), 5.51 – 5.43 (m, 1H), 5.38 – 5.30 (m, 1H), 3.13 – 3.12 (m, 1H), 3.09 – 3.07 (m, 2H), 2.90 – 2.81 (m, 2H), 2.18 – 1.91 (m, 3H), 1.78 – 1.70 (m, 1H), 1.63 – 1.59 (m, 3H), 1.44 – 1.40 (m, 3H), 0.98 (td, J = 7.2, 0.8 Hz, 3H). ^{13}C NMR (100 MHz, $CDCl_3$) δ: 129.0, 126.8, 53.8, 46.3, 32.4, 28.7, 19.7, 18.0, 15.9, 11.5. IR (film) cm^{-1}: 3396, 1590, 1265, 1079, 966, 732, 701. HRMS (ESI-Orbitrap): *m/z* calculated for $C_{10}H_{22}N$ $[M]^+$: 156.1747, found 156.1747.

(E)-2-methyl-N-propylhex-4-en-1-aminium (5)

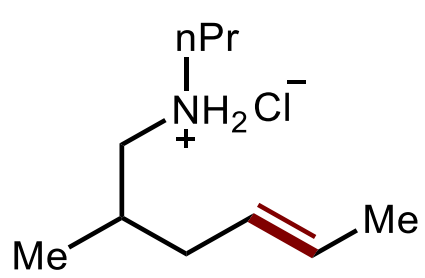

Prepared following **GP1** with **S5**. The crude product (56%, >10:1 r.r., E:Z 1:1) was recrystallized with EtOAc to afford a white solid. ^{1}H NMR (400 MHz, $CDCl_3$) δ 9.47 (br s, 2H), 5.50 – 5.41 (m, 1H), 5.25 – 5.19 (m, 1H), 2.89 (br s, 4H), 2.21 – 2.14 (m, 1H), 1.96 – 1.92 (m, 2H), 1.79 – 1.75 (m, 2H), 1.65 – 1.59 (m, 3H), 1.02 – 0.99 (m, 6H). ^{13}C NMR (100 MHz, $CDCl_3$) δ: 135.2, 125.4, 49.6, 46.6, 39.0, 35.3, 32.6, 21.2, 19.6, 11.5. IR (film) cm^{-1}: 3396, 1592, 1147, 970, 733, 701. HRMS (ESI-Orbitrap): *m/z* calculated for $C_{10}H_{22}N$ $[M]^+$: 156.1747, found 156.1747.

(E)-3-methyl-N-propylhex-4-en-1-aminium (6)

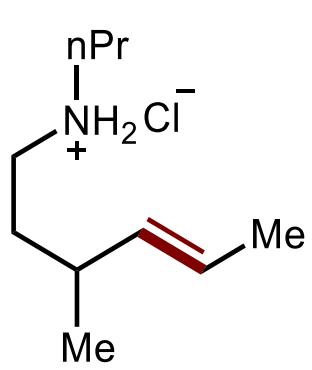

Prepared following **GP1** with **S6**. The crude product (80%, 3:1 r.r., E:Z 6:1) was recrystallized with EtOAc to afford a white solid. ^{1}H NMR (400 MHz, $CDCl_3$) δ 9.43 (br s, 2H), 5.48 – 5.39 (m, 1H), 5.24 – 5.17 (m, 1H), 2.87 – 2.86 (m, 4H), 2.20 – 2.12 (m, 1H), 1.95 – 1.89 (m, 4H), 1.60 (dd, J = 8.8, 1.6 Hz, 3H), 1.00 – 0.97 (m, 6H). ^{13}C NMR (100 MHz, $CDCl_3$) δ: 135.2, 125.3, 49.5, 46.5, 35.2, 32.5, 21.1, 19.5, 17.9, 11.4. IR (film) cm^{-1}: 3400, 1592, 1266, 1049, 970, 732, 701. HRMS (ESI-Orbitrap): *m/z* calculated for $C_{10}H_{22}N$ $[M]^+$: 156.1747, found 156.1747.

N-(2-(cyclopent-2-en-1-yl)ethyl)propan-1-aminium (7)

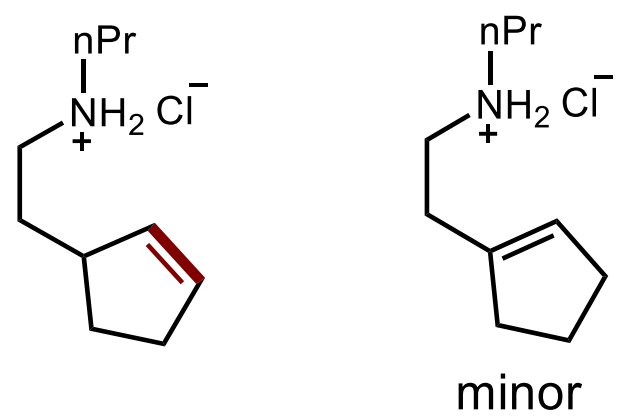

Prepared following **GP1** with **S7**. The crude product (66%, 1.2:1 r.r.) was recrystallized with EtOAc to afford a white solid. ^{1}H NMR (400 MHz, $CDCl_3$) δ 9.49 (br s, 2H), 5.76 – 5.74 (m, 1H), 5.60 – 5.58 (m, 1H), 2.92 – 2.87 (m, 4H), 2.75 – 2.66 (m, 2H), 2.32 – 2.27 (m, 2H), 2.08 – 2.00 (m, 2H), 1.84 – 1.83 (m, 2H), 1.45 – 1.40 (m, 1H), 1.02 – 0.98 (m, 3H). ^{13}C NMR (100 MHz, $CDCl_3$) δ: 133.0, 132.0, 49.4, 46.4, 43.1, 32.1, 31.7, 29.3, 19.6, 11.4. IR (film) cm^{-1}: 3388, 1593, 1266, 1140, 1019, 911, 732, 701. HRMS (ESI-Orbitrap): *m/z* calculated for $C_{10}H_{20}N$ $[M]^+$: 154.1590, found 154.1590.

N-(2-(cyclohex-2-en-1-yl)ethyl)propan-1-aminium (8)

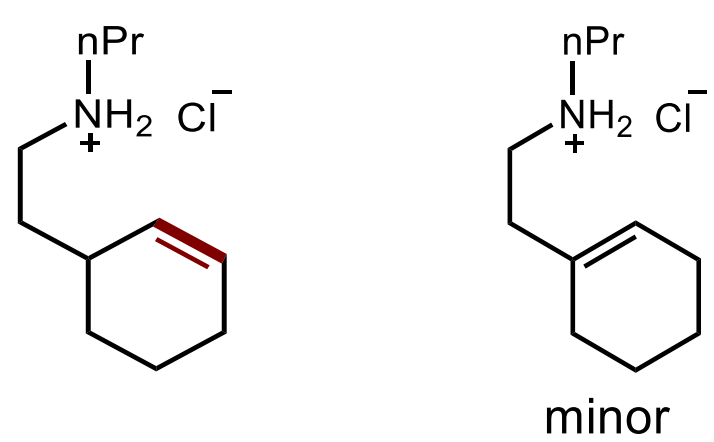

Prepared following **GP1** with **S8**. The crude product (57%, 1.2:1 r.r.) was recrystallized with EtOAc to afford a white solid. ^{1}H NMR (400 MHz, $CDCl_3$) δ 9.49 (br s, 2H), 5.73 – 5.68 (m, 1H), 5.51 – 5.48 (m, 1H), 3.01 – 2.94 (m, 2H), 2.92 – 2.85 (m, 2H), 2.23 – 2.17 (m, 1H), 1.96 – 1.90 (m, 5H), 1.79 – 1.75 (m, 3H), 1.72 – 1.67 (m, 2H), 1.00 (t, J = 7.6 Hz, 3H). ^{13}C NMR (100 MHz, $CDCl_3$) δ: 129.8, 128.7, 49.5, 45.9, 33.1, 31.9, 28.5, 25.1, 21.2, 19.6, 11.4. IR (film) cm^{-1}: 3017, 1264, 1067, 917, 731, 702. HRMS (ESI-Orbitrap): *m/z* calculated for $C_{11}H_{22}N$ $[M]^+$: 168.1747, found 168.1746.

(E)-2-(pent-3-en-1-yl)piperidin-1-ium (9)

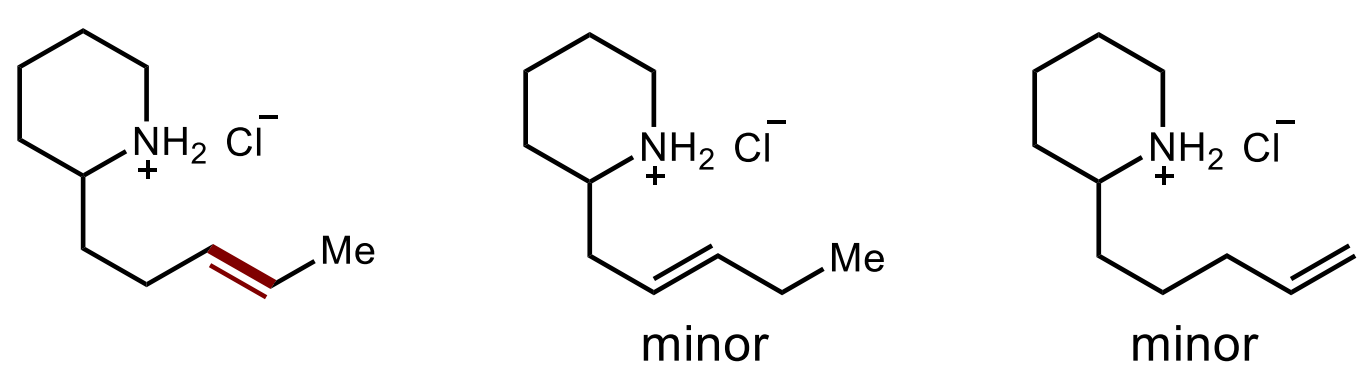

Prepared following **GP1** with **S9**. The crude product (77%, 4:1 r.r., E:Z 1.3:1) was recrystallized with EtOAc to afford a white solid. ^{1}H NMR (400 MHz, $CDCl_3$) δ 9.58 – 9.24 (m, 2H), 5.54 – 5.46 (m, 1H), 5.39 – 5.29 (m, 1H), 2.93 – 2.92 (m, 1H), 2.85 – 2.75 (m, 2H), 2.13 – 1.84 (m, 7H), 1.62 (td, J = 6.4, 1.2 Hz, 3H), 1.47 – 1.24 (m, 3H). ^{13}C NMR (100 MHz, $CDCl_3$) δ:129.1, 126.8, 56.8, 44.9, 33.0, 28.2, 28.1, 22.5, 22.45, 18.0. IR (film) cm^{-1}: 2933, 1455, 1028, 997, 732, 700. HRMS (ESI-Orbitrap): *m/z* calculated for $C_{10}H_{20}N$ $[M]^+$: 154.1590, found 154.1590.

N-propylpent-4-en-1-aminium (10)

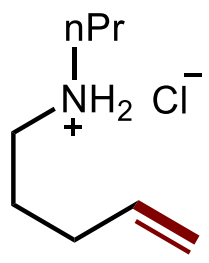

Prepared following **GP1** with **S10**. The crude product (54%, >10:1 r.r.) was recrystallized with EtOAc to afford a white solid. ^{1}H NMR (400 MHz, $CDCl_3$) δ 9.54 (br s, 2H), 5.80 – 5.70 (m, 1H), 5.10 – 5.01 (m, 2H), 2.90 (br s, 4H), 2.17 – 2.14 (m, 2H), 2.05 – 1.91 (m, 2H), 1.67 – 1.65 (m, 1H), 1.35 – 1.34 (m, 1H), 1.01 (t, *J* = 6.4 Hz, 3H). ^{13}C NMR (100 MHz, $CDCl_3$) δ: 136.4, 116.6, 49.5, 47.3, 30.9, 25.0, 19.6, 11.5. IR (film) cm^{-1}: 3390, 1641, 1128, 1061, 992, 971, 719. HRMS (ESI-Orbitrap): *m/z* calculated for $C_8H_{18}N$ $[M]^+$: 128.1434, found 128.1433.

2,2-dimethyl-N-propylpent-4-en-1-aminium (11)

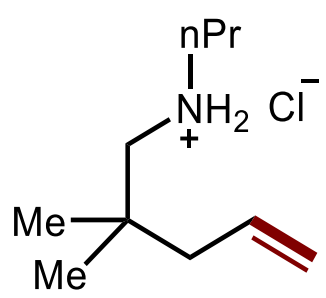

Prepared following **GP1** with **S11**. The crude product (50%, >10:1 r.r.) was recrystallized with EtOAc to afford a white solid. ^{1}H NMR (400 MHz, $CDCl_3$) δ 9.15 (br s, 2H), 5.87 – 5.73 (m, 1H), 5.23 – 5.09 (m, 2H), 3.02 – 2.93 (m, 2H), 2.77 – 2.71 (m, 2H), 2.26 – 2.21 (m, 2H), 2.00 – 1.89 (m, 2H), 1.16 (s, 6H), 1.00 – 0.94 (m, 3H). ^{13}C NMR (100 MHz, $CDCl_3$) δ: 133.4, 119.3, 57.3, 51.1, 44.5, 33.8, 25.5, 18.7, 11.4. IR (film) cm^{-1}: 2967, 1265, 731, 702. HRMS (ESI-Orbitrap): *m/z* calculated for $C_{10}H_{22}N$ $[M]^+$: 156.1747, found 156.1753.

(E)-hex-4-en-1-aminium (12)

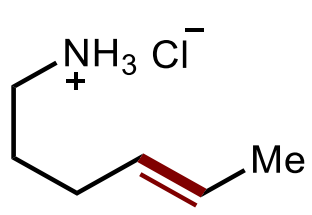

Prepared following **GP1** with **S12**. The crude product (43%, >10:1 r.r., E:Z 1:1) was recrystallized with EtOAc to afford a white solid. ^{1}H NMR (400 MHz, $CDCl_3$) δ 8.24 – 8.08 (br s, 3H), 5.53 – 5.49 (m, 1H), 5.37 – 5.34 (m, 1H), 3.02 (br s, 2H), 2.17 – 2.11 (m, 2H), 1.83 – 1.79 (m, 2H), 1.64 – 1.59 (m, 3H). ^{13}C NMR (100 MHz, $CDCl_3$) δ:128.8, 127.0, 31.2, 22.5, 17.8, 14.0. IR (film) cm^{-1}: 3412, 1608, 1505, 1155, 947, 735, 701. HRMS (ESI-Orbitrap): *m/z* calculated for $C_6H_{14}N$ $[M]^+$: 100.1121, found 100.1120.

(E)-hept-5-en-2-aminium (13)

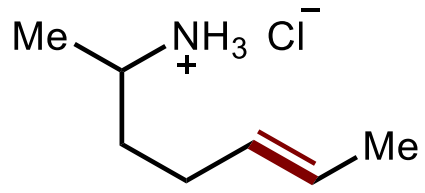

Prepared following **GP1** with **S13**. The crude product (57%, >10:1 r.r., E:Z 1:1) was recrystallized with EtOAc to afford a white solid. ^{1}H NMR (400 MHz, $CDCl_3$) δ 8.34 (br s, 3H), 5.56 – 5.47 (m, 1H), 5.39 – 5.30 (m, 1H), 3.33 – 3.30 (m, 1H), 2.20 – 2.10 (m, 2H), 1.92 – 1.84 (m, 1H), 1.71 – 1.67 (m, 1H), 1.63 (td, *J* = 6.4,1.2 Hz, 3H), 0.90 – 0.86 (m, 3H). ^{13}C NMR (100 MHz, $CDCl_3$) δ: 128.9, 127.0, 48.1, 34.9, 28.6, 23.2, 18.0. IR (film) cm^{-1}: 3378, 1614, 1515, 1193, 947, 704. HRMS (ESI-Orbitrap): *m/z* calculated for $C_7H_{16}N$ $[M]^+$: 114.1277, found 114.1277.

(E)-undec-2-en-6-aminium (14)

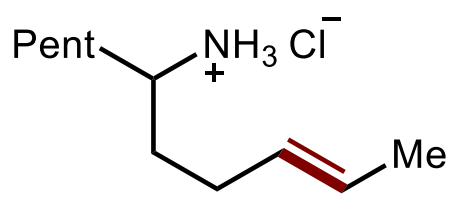

Prepared following **GP1** with **S14**. The crude product (84%, >10:1 r.r., E:Z 1.3:1) was recrystallized with EtOAc to afford a white solid. ^{1}H NMR (400 MHz, $CDCl_3$) δ 9.47 (br s, 3H), 5.50 – 5.44 (m, 1H), 5.35 – 5.30 (m, 1H), 2.88 (br s, 1H), 2.15 – 2.04 (m, 2H), 2.01 – 1.84 (m, 4H), 1.62 – 1.57 (m, 3H), 1.35 – 1.27 (m, 6H), 0.86 (t, *J* = 6.8 Hz, 3H). ^{13}C NMR (100 MHz, $CDCl_3$) δ: 128.9, 127.1, 48.0, 31.3, 29.9, 26.9, 25.7, 24.2, 22.7, 17.9, 14.0. IR (film) cm^{-1}: 3391, 1591, 1265, 1112, 945, 910, 792, 703. HRMS (ESI-Orbitrap): *m/z* calculated for $C_{11}H_{24}N$ $[M]^+$: 170.1903, found 170.1903.

((1S,4aR,10aS)-7-isopropyl-1,4a-dimethyl-1,2,3,4,4a,10a-hexahydrophenanthren-1-yl)methanaminium (15)

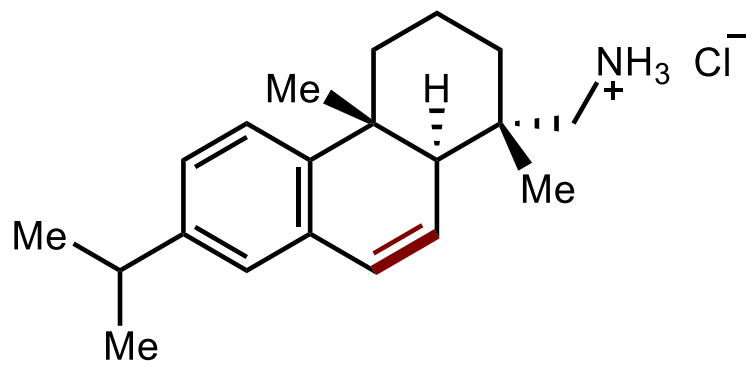

Prepared following **GP1** with **S15**. The crude product (50%, >10:1 r.r.) was recrystallized with EtOAc to afford a white solid. ^{1}H NMR (400 MHz, $CDCl_3$) δ 8.35 (br s, 3H), 7.069 – 7.065 (m, 2H), 6.93 (s, 1H), 6.57 (dd, *J* = 9.6, 3.2 Hz, 1H), 5.80 (dd, *J* = 9.6, 3.2 Hz, 1H), 3.12 (d, *J* = 7.6 Hz, 1H), 2.88 – 2.81 (m, 1H), 2.57 (d, *J* = 8.0 Hz, 1H), 2.21 – 2.15 (m, 2H), 1.88 – 1.70 (m, 3H), 1.65 – 1.57 (m, 1H), 1.36-1.30 (m, 1H), 1.23 – 1.22 (m, 9H), 1.07 (s, 3H). ^{13}C NMR (100 MHz, $CDCl_3$) δ: 146.4, 145.0, 132.1, 129.3, 126.8, 126.0, 124.8, 121.7, 51.4, 48.4, 37.6, 35.7, 35.23, 35.15, 33.46, 23.88, 23.87, 20.6, 18.0, 17.7. IR (film) cm^{-1}: 3390, 1597, 1512, 1144, 1006, 888, 795. HRMS (ESI-Orbitrap): *m/z* calculated for $C_{20}H_{30}N$ $[M]^+$: 284.2373, found 284.2371.

(E)-1-methoxy-1-oxohept-5-en-2-aminium (16)

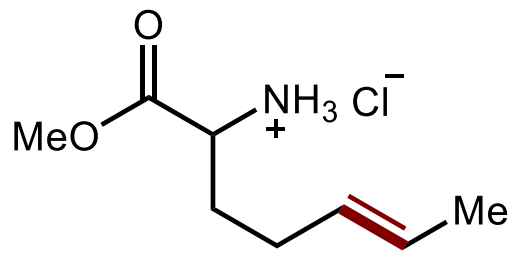

Prepared following **GP1** with **S16**. The crude product (40%, >10:1 r.r., E:Z 1.5:1) was recrystallized with EtOAc to afford a white solid. ^{1}H NMR (400 MHz, $CDCl_3$) δ 8.73 (br s, 3H), 5.59 – 5.46 (m, 1H), 5.39 – 5.34 (m, 1H), 4.11 (br s, 1H), 3.79 (s, 3H), 2.29 – 2.05 (m, 4H), 1.62 – 1.60 (m, 3H). ^{13}C NMR (100 MHz, $CDCl_3$) δ: 170.0, 128.5, 127.4, 53.2, 30.3, 28.0, 22.8, 18.0. IR (film) cm^{-1}: 3415, 1745, 1524, 1149, 1071, 967, 918, 702. HRMS (ESI-Orbitrap): *m/z* calculated for $C_8H_{16}NO_2$ $[M]^+$: 158.1176, found 158.1174.

VII. Post-Functionalizations: Synthetic Utility of δ Amino Alkenes

Pyrrolidines (via 5-*exo*-cyclizations)

2-(chloromethyl)-1-propylpyrrolidin-1-ium (17)

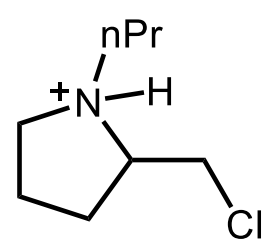

To an 8 ml vial containing a solution of 2,2-dimethyl-N-propylpent-4-en-1-amine (**10**) (63.6 mg, 0.5 mmol) in HFIP (5 ml) was added NCS (66.8 mg, 0.5 mmol) and the reaction was stirred in the dark at room temperature for 30 min. TFA (42 µL, 0.55 mmol) was added and stirred for 1h. The solvent was removed in vacuo. Analysis by crude NMR revealed 83% product (**17**) via internal standard nitromethane. The residual succinimide was removed by diluting the residue with DCM. The organic layer was dried over Na_2SO_4, filtered and dried in vacuo yielding **17** as a white solid. ^{1}H NMR (400 MHz, $CDCl_3$) δ 10.97 (br s, 1H), 4.05 (dd, *J* = 12.0, 6.0 Hz, 1H), 3.99 – 3.95 (m, 1H), 3.79 (dd, *J* = 12.0, 7.2 Hz, 1H), 3.56 – 3.51 (m, 1H), 3.46 – 3.39 (m, 1H), 3.05 – 2.94 (m, 2H), 2.42 – 2.34 (m, 1H), 2.27 – 2.19 (m, 1H), 2.12 – 2.04 (m, 2H), 1.92 – 1.70 (m, 2H), 1.01 (t, *J* = 7.2 Hz, 3H). ^{13}C NMR (100 MHz, CDCl3) δ 69.4, 57.7, 55.0, 41.7, 29.1, 22.2, 18.8, 10.9. IR (film) cm-1: 2969, 2881, 1708, 1644, 1462, 1379, 1342, 1291, 1219, 1128, 1104, 950, 816, 628. HRMS (ESI): m/z calculated for $C_8H_{17}NCl$ [M+H]+: 162.1050, found 162.1044.

2-(hydroxymethyl)-1-propylpyrrolidin-1-ium (18)

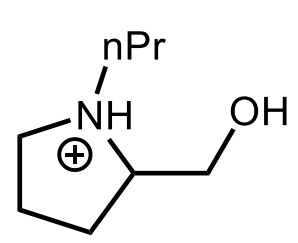

The title compound was synthesized from **10** and was identical by both ^{1}HNMR and HRMS to an authentic material. Analysis by crude NMR revealed 50% of product (**18**) via internal standard nitromethane. ^{1}H NMR (400 MHz, $CDCl_3$) δ 10.8 (br s, 1H), 4.78 (br s, 1H), 3.97 (dd, *J* = 13.2, 2.8 Hz, 1H), 3.86 (dd, *J* = 13.2, 6.4 Hz, 1H), 3.79 – 3.72 (m, 1H), 3.56 – 3.52 (m, 1H), 3.35 – 3.27 (m, 1H), 3.02 – 2.92 (m, 2H), 2.18 – 1.97 (m, 5H), 1.87 – 1.76 (m, 1H), 0.98 (t, *J* = 7.2 Hz, 3H). HRMS (ESI-Orbitrap): *m/z* calculated for $C_8H_{18}NO$ $[M]^+$: 144.1383, found 144.1383.

Piperidines (via 6-*endo*-cyclizations)

5-chloro-3,3-dimethyl-1-propylpiperidine (19)

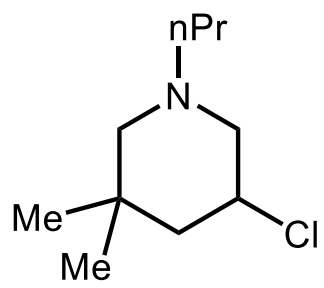

Modified version of procedure reported by Wang and colleagues.[63] To an 8 ml vial containing a solution of 2,2-dimethyl-N-propylpent-4-en-1-amine (**10**) (47 mg, 0.3 mmol) in $CHCl_3$ (3 ml) was added NCS (40 mg, 0.3 mmol) and the reaction was stirred in the dark at room temperature for 1 h. The vial was opened followed by addition of NaI (4.5 mg, 0.03 mmol), resealed and heated to 50°C for 1 h. The reaction was cooled to room temperature and the organics were concentrated. Analysis of the crude NMR revealed 99% of product **19** via internal standard DCE. The residual succinimide was removed by diluting the residue with DCM (15 ml) and washing the organic layer with H_2O (10 ml x3). The organic layer was dried over Na_2SO_4 and dried in vacuo yielding pure **19** as a colorless oil (54 mg, 95%). R_f: 0.51 (10% EtOAc:Hex). ^1H NMR (400 MHz, $CDCl_3$) δ 4.08 (m, 1H), 3.18–3.10 (m, 1H), 2.44 – 2.37 (m, 1H), 2.35–2.21 (m, 2H), 1.96–1.88 (m, 2H), 1.68 (d, *J* = 10.8 Hz, 1H), 1.52–1.39 (m, 2H), 1.32 (t, *J* = 12.4 Hz, 1H), 1.03 (s, 3H), 0.91 (s, 3H), 0.88 (t, *J* = 7.2 Hz, 3H). ^{13}C NMR (100 MHz, $CDCl_3$) δ 64.8, 62.5, 60.0, 54.6, 48.6, 33.4, 29.6, 25.4, 20.2, 11.9. IR (film) cm^{-1}: 2956, 2872, 2762, 1709, 1464, 1209, 1196, 1084, 1008, 908, 808, 747. HRMS (ESI): *m/z* calculated for $C_{10}H_{20}NCl$ $[M+H]^+$: 190.1357, found 190.1357.

5-fluoro-3,3-dimethyl-1-propylpiperidine (20)

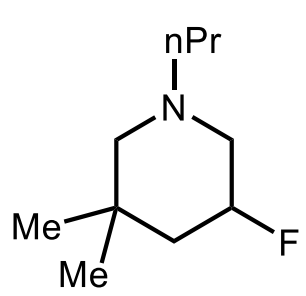

Procedure by Wang and colleagues.[63] To an 8 ml vial containing a solution of 2,2-dimethyl-N-propylpent-4-en-1-amine (**10**) (47 mg, 0.3 mmol) in tBuOH (1.5 ml) was added NCS (40 mg, 0.3 mmol) and the reaction was stirred in the dark at room temperature for 2 h. The vial was opened followed by addition of NaI (4.5 mg, 0.03 mmol), resealed and heated to 70°C for an additional 2 h. The vial was opened and charged with AgF (114 mg, 0.9 mmol) sealed and continued heating at 70°C for 16 h. The reaction was cooled to room temperature and filtered through a short column of celite with DCM and the organics were concentrated. Analysis of the crude NMR revealed 60% of product **20** via internal standard DCE. The residue was purified via column chromatography (10% EtOAc:Hex) yielding pure **20** as a colorless oil (33 mg, 63%). R_f: 0.29 (5% EtOAc:Hex). ^1H NMR (400 MHz, $CDCl_3$) δ 4.71 (dsept, *J* = 12.0, 4.4 Hz, 1H), 2.96 – 2.82 (m, 1H), 2.30 (t, *J* = 7.2 Hz, 2H), 2.25 – 2.18 (m, 1H), 2.10 (q, *J* = 8.8 Hz, 1H), 1.88 – 1.81 (m, 1H), 1.78 – 1.67 (m, 1H), 1.48 (sext, *J* = 7.2 Hz, 2H), 1.37 – 1.23 (m, 2H), 0.98 (s, 6H), 0.89 (t, *J* = 7.2 Hz, 3H). ^{13}C NMR (100 MHz, $CDCl_3$) δ 88.1 (d, *J* = 169 Hz), 65.1, 60.2, 58.6 (d, *J* = 24 Hz), 43.6 (d, *J* = 17 Hz), 32.0 (d, *J* = 9 Hz), 29.1, 26.9, 20.2, 11.9 . ^{19}F (377 MHz, $CDCl_3$) δ -182.0. IR (film) cm^{-1}:

2954, 2804, 1469, 1365, 1214, 1142, 1004, 929, 768. HRMS (ESI): *m/z* calculated for $C_{10}H_{20}NF$ $[M+H]^+$: 174.1653, found 174.1653.

5-azido-3,3-dimethyl-1-propylpiperidine (21)

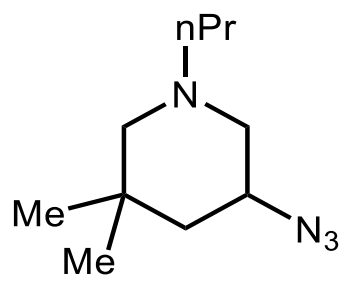

Procedure by Wang and colleagues.[63] To a 20 ml vial containing a solution of 2,2-dimethyl-N-propylpent-4-en-1-amine (**10**) (188 mg, 1.23 mmol) in MeCN (12 ml) was added NCS (164 mg, 1.23 mmol) and the reaction was stirred in the dark at room temperature for 2 h. The vial was opened followed by addition of NaI (9 mg, 0.06 mmol) and NaN_3 (96 mg, 1.44 mmol), resealed and heated to 60°C for an additional 48 h. The reaction was cooled to room temperature and diluted with 0.4M NaOH (30 ml), then extracted with hexanes (40 ml, x4). The combined organic layers were washed with 1.25M NaOH (20 ml), brine (60 ml) then the organic layer was dried with Na_2SO_4, concentrated. Analysis of the crude NMR revealed 88% of product **21** via internal standard DCE. The residue was purified by keugelrohr distillation yielding pure **21** as a colorless oil (181 mg, 75%). R_f: 0.54 (10% EtOAc:Hex). ^{1}H NMR (400 MHz, $CDCl_3$) δ 3.63 – 3.51 (m, 1H), 3.02 – 2.93 (m, 1H), 2.41 – 2.34 (m, 1H), 2.34 – 2.20 (m, 2 H), 1.80 – 1.62 (m, 3H), 1.46 (sext, J = 7.2 Hz, 2 H), 1.15 – 1.05 (m, 1H), 1.02 (s, 3H), 0.93 (s, 3H), 0.88 (t, J = 7.4 Hz, 3H). ^{13}C NMR (100 MHz, $CDCl_3$) δ 65.0, 60.2, 58.6, 55.8, 43.0, 31.8, 29.6, 25.8, 20.2, 11.9. IR (film) cm^{-1}: 2955, 2804, 2766, 2091, 1465, 1249, 1212, 1112, 1010, 912, 702, 681. HRMS (ESI): *m/z* calculated for $C_{10}H_{20}N_4$ $[M+H]^+$: 197.1761, found 197.1762.

3,3-dimethyl-5-(4-phenyl-1H-1,2,3-triazol-1-yl)-1-propylpiperidine (22)

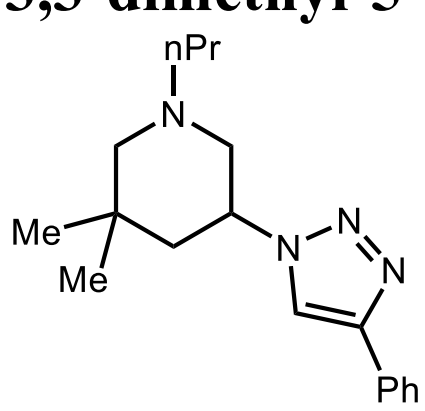

Procedure by Wang and colleagues.[63] To an 8 ml vial containing a solution of 5-azido-3,3-dimethyl-1-propylpiperidine (**21**) (29 mg, 0.15 mmol) in tBuOH : H_2O (0.6 ml, 1:1) was added sodium ascorbate (30 mg, 0.015 mmol), phenyl acetylene (16.5 uL, 0.15 mmol) and $CuSO_4$•5H_2O (0.8 mg, 0.003 mmol) and the reaction was stirred vigorously at 60°C for 12 h. The reaction was allowed to cool to room temperature and water added to induce crystallization. The solid was filtered and washed with excess H_2O and allowed to dry yielding pure **22** as an off-white solid (28 mg, 63%). R_f: 0.29 (20% EtOAc:Hex). ^{1}H NMR (400 MHz, $CDCl_3$) δ 7.84 – 7.80 (m, 2H), 7.76 (s, 1H), 7.45 – 7.39 (m, 2H), 7.35 – 7.29 (m, 1H), 4.86 (ddt, J = 12.4, 10.8, 4.3 Hz, 1H), 3.33 – 3.25 (m, 1H), 2.56 – 2.49 (m, 1H), 2.38 – 2.31 (m, 2H), 2.24 (t, J = 10.6 Hz, 1H), 2.02 – 1.94 (m, 1H), 1.84 (d, J = 11.2 Hz, 1H), 1.71 (t, J = 12.0 Hz, 1H), 1.50 (sext, J = 7.2 Hz, 2H), 1.16 (s, 3H), 1.00 (s, 3H), 0.91 (t, J = 7.2 Hz, 3H). ^{13}C NMR (100 MHz, $CDCl_3$) δ 147.4, 130.9, 129.0, 128.2, 125.8, 118.0, 65.0, 60.1, 59.9, 56.1, 44.2, 31.9, 29.6, 25.5, 20.2, 11.9. IR (film) cm^{-1}: 3117, 2959,

2806, 1482, 1364, 1281, 1079, 975, 766, 692. HRMS (ESI): *m/z* calculated for $C_{18}H_{26}N_4$ $[M+H]^+$: 299.2230, found 299.2235.

***N*-isobutyl-5,5-dimethyl-1-propylpiperidin-3-amine (23)**

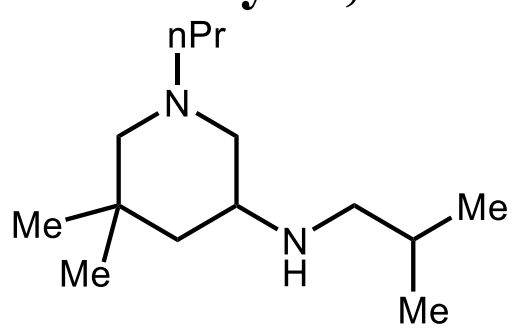

Procedure by Wang and colleagues.[63] To an 8 ml vial containing a solution of 5-azido-3,3-dimethyl-1-propylpiperidine (**21**) (29 mg, 0.15 mmol) in DCM (1.5 ml) was added $P(nBu)_3$ (74uL, 0.3 mmol) and stirred at 25°C for 4 h. Next isobutyraldehyde was added (34uL, 0.375 mmol) and the reaction stirred for an additional 12 h. Next 4 ml of MeOH was added to the reaction mixture followed by addition of $NaBH_4$ (17 mg, 0.45 mmol), and let stir at 25°C for 1 h. The reaction was quenched by addition of water and let to stir for 30 minutes. Extraction of the reaction mixture with DCM (x3 10 ml), and drying over $MgSO_4$ gave the crude reaction mixture. Analysis of the crude NMR revealed 99% of product **23** via internal standard DCE. Upon standing for 3 days to oxidize remaining $P(nBu)_3$, the residue was purified by acid-base extraction from DCM with 1M HCl, and back-extraction and neutralization using 6M NaOH and DCM yielding pure **23** as a colorless oil (9 mg, 27%). R_f: 0.30 (5% MeOH:DCM). ^1H NMR (400 MHz, $CDCl_3$) δ 3.05 – 3.01 (m, 1H), 2.81 – 2.73 (m, 1H), 2.44 – 2.39 (m, 3H), 2.33 – 2.16 (m, 2H), 1.70 – 1.43 (m, 7H), 1.01 (s, 3H), 0.89– 0.88 (m, 13H). ^{13}C NMR (100 MHz, $CDCl_3$) δ 65.7, 61.5, 60.7, 55.5, 52.2, 45.7, 31.5, 30.1, 28.9, 26.1, 20.9, 20.8, 20.3, 12.0. IR (film) cm^{-1}: 3635, 2869, 2761, 1635, 1468, 1385, 1210, 1167, 885, 715, 520. HRMS (ESI): *m/z* calculated for $C_{14}H_{30}N_2$ $[M+H]^+$: 227.2482, found 227.2476.

5,5-dimethyl-1-propylpiperidin-3-amine (24)

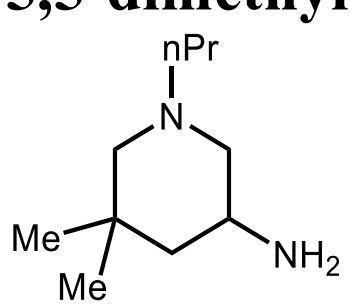

Procedure by Wang and colleagues.[63] To an 8 ml vial containing a solution of 5-azido-3,3-dimethyl-1-propylpiperidine (**21**) (29 mg, 0.15 mmol) in THF (3 ml) was added H_2O (19 uL, 1mmol) then PPh_3 (59 mg, 0.23 mmol) and the reaction mixture was heated to reflux for 5 h. The reaction was cooled to room temperature and the solvent removed in vacuo. Analysis of the crude NMR revealed 99% of product **24** via internal standard DCE. The residue was purified by acid-base extraction from DCM with 1M HCl, and back-extraction and neutralization using 6M NaOH and DCM yielding pure **24** as a colorless oil (19 mg, 74%). R_f: 0.35 (10% MeOH:DCM). ^1H NMR (400 MHz, $CDCl_3$) δ 3.02 – 2.87 (m, 2H), 2.43 (m, 1H), 2.32 – 2.14 (m, 2H), 1.62–1.57 (m, 1H), 1.56 – 1.42 (m, 4H), 1.25 – 1.10 (m, 2H), 1.00 (s, 3H), 0.90 – 0.85 (m, 6H), 0.80–0.75 (m, 1H). ^{13}C NMR (100 MHz, $CDCl_3$) δ 65.4, 64.4, 60.5, 48.5, 45.9, 31.7, 30.0, 26.0, 20.3, 12.0. IR (film) cm^{-1}: 3280, 2951, 1596, 1463, 1304, 1192, 1072, 862, 785. HRMS (ESI): *m/z* calculated for $C_{10}H_{22}N_2$ $[M+H]^+$: 171.1856, found 171.1856.

VIII. Applications to Medicines and Biological Molecules

Drug compound tolerance

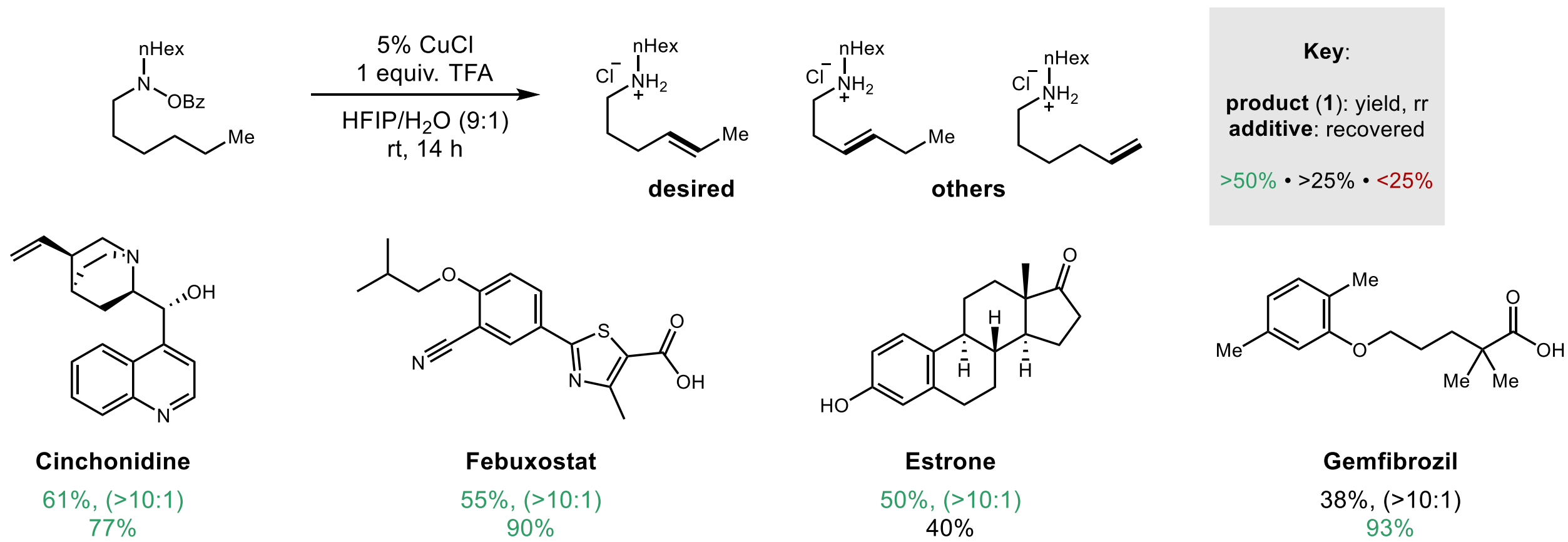

Application to biological molecules

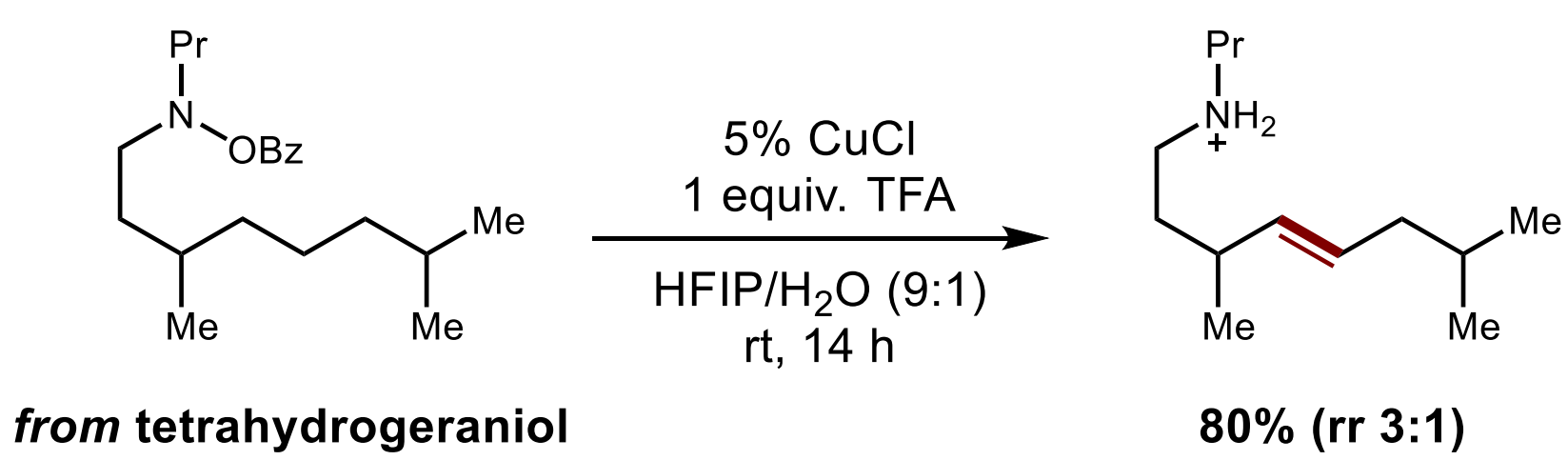

(E)-3,7-dimethyl-N-propyloct-4-en-1-aminium (25)

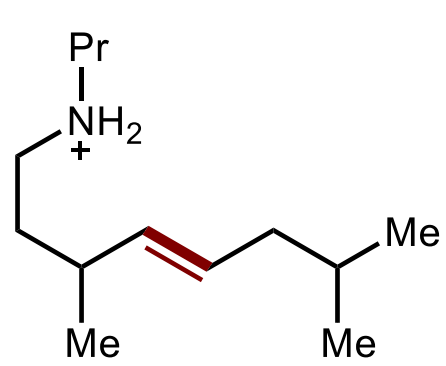

Prepared following **GP1** with **S18**. The crude product (80%, 3:1 r.r.) was recrystallized with EtOAc to afford a white solid. ^{1}H NMR (400 MHz, $CDCl_3$) δ 9.43 (br s, 2H), 5.41 – 5.37 (m, 1H), 5.16 (dd, J = 10.0, 5.6 Hz, 1H), 2.87 – 2.83 (m, 4H), 2.18 – 2.16 (m, 1H), 1.92 – 1.89 (m, 4H), 1.84 – 1.79 (m, 2H), 1.57 – 1.53 (m, 1H), 1.00 – 0.97 (m, 6H), 0.89 – 0.83 (m, 6H). ^{13}C NMR (100 MHz, $CDCl_3$) δ: 135.1, 129.7, 49.6, 46.6, 41.8, 35.3, 32.6, 28.4, 22.4, 22.3, 21.3, 19.5, 11.4. IR (film) cm^{-1}: 2956, 1593, 1271, 1051, 970, 757, 664. HRMS (ESI-Orbitrap): m/z calculated for $C_{13}H_{28}N$ $[M]^+$: 198.2216, found 198.2216.

IX. Mechanistic Studies

Radical Clock Probe of Cu Desaturation

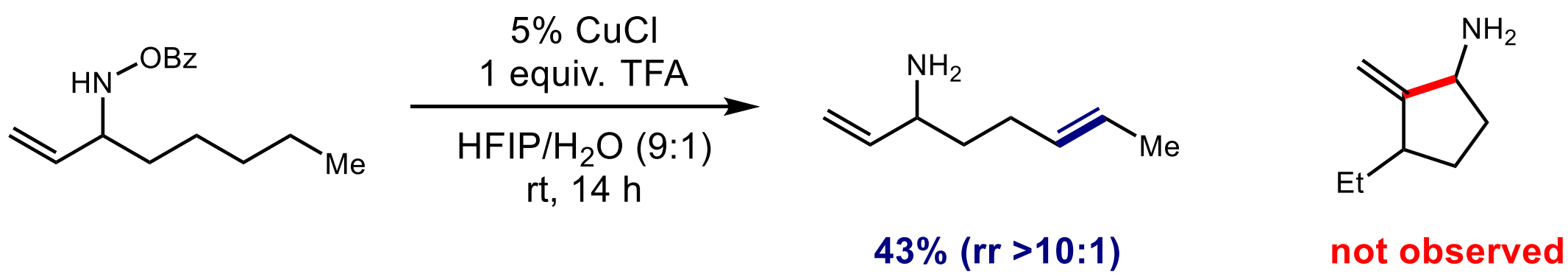

(*E*)-octa-1,6-dien-3-aminium (26)

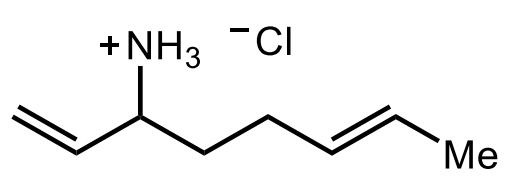

Prepared following **GP1** with **S19**. The crude product (43%, >10:1 r.r., E:Z 2:1) was recrystallized with EtOAc to afford a white solid. ^{1}H NMR (400 MHz, CDCl3) δ 8.55 (br s, 3H), 5.93 – 5.82 (m, 1H), 5.52 – 5.43 (m, 2H), 5.42 – 5.31 (m, 2H), 3.71 (br s, 1H), 2.15 – 2.04 (m, 2H), 2.00 – 1.92 (m, 1H), 1.89 – 1.72 (m, 1H), 1.65 – 1.58 (m, 3H). ^{13}C NMR (100 MHz, $CDCl_3$) δ: 133.4, 128.8, 127.0, 121.0, 54.4, 33.0, 28.3, 18.0. IR (film) cm^{-1}: 3642, 1631, 1443, 1375, 1038, 918, 749. HRMS (ESI-Orbitrap): *m/z* calculated for $C_8H_{16}N$ $[M]^+$: 126.1283, found 126.1276.

Regioselectivity of Desaturation

O-benzoyl-N,N-dihexylhydroxylamine **S1** (0.2 mmol) was subjected to **GP1**. After 14 h, the product yield and regioselectivity ratio (rr) was determined by ^{1}H NMR of the HCl salt. The structures of the major and minor regioisomers were assigned by 2D COSY NMR based on the connectivity between the vinylic proton and the terminal methyl group.

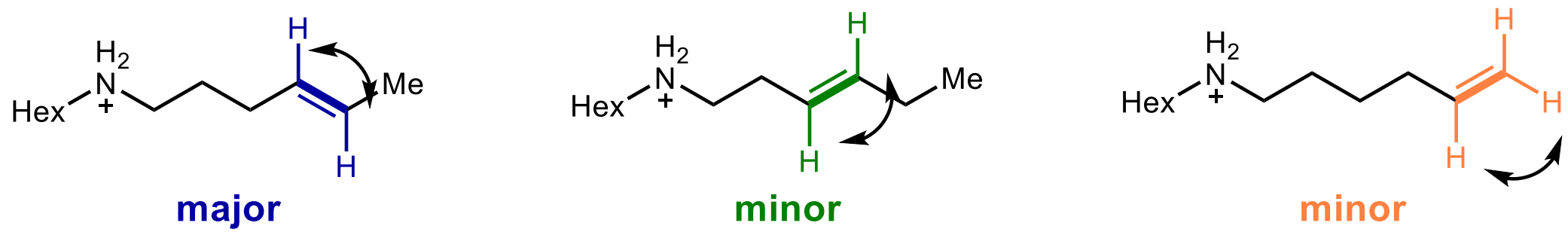

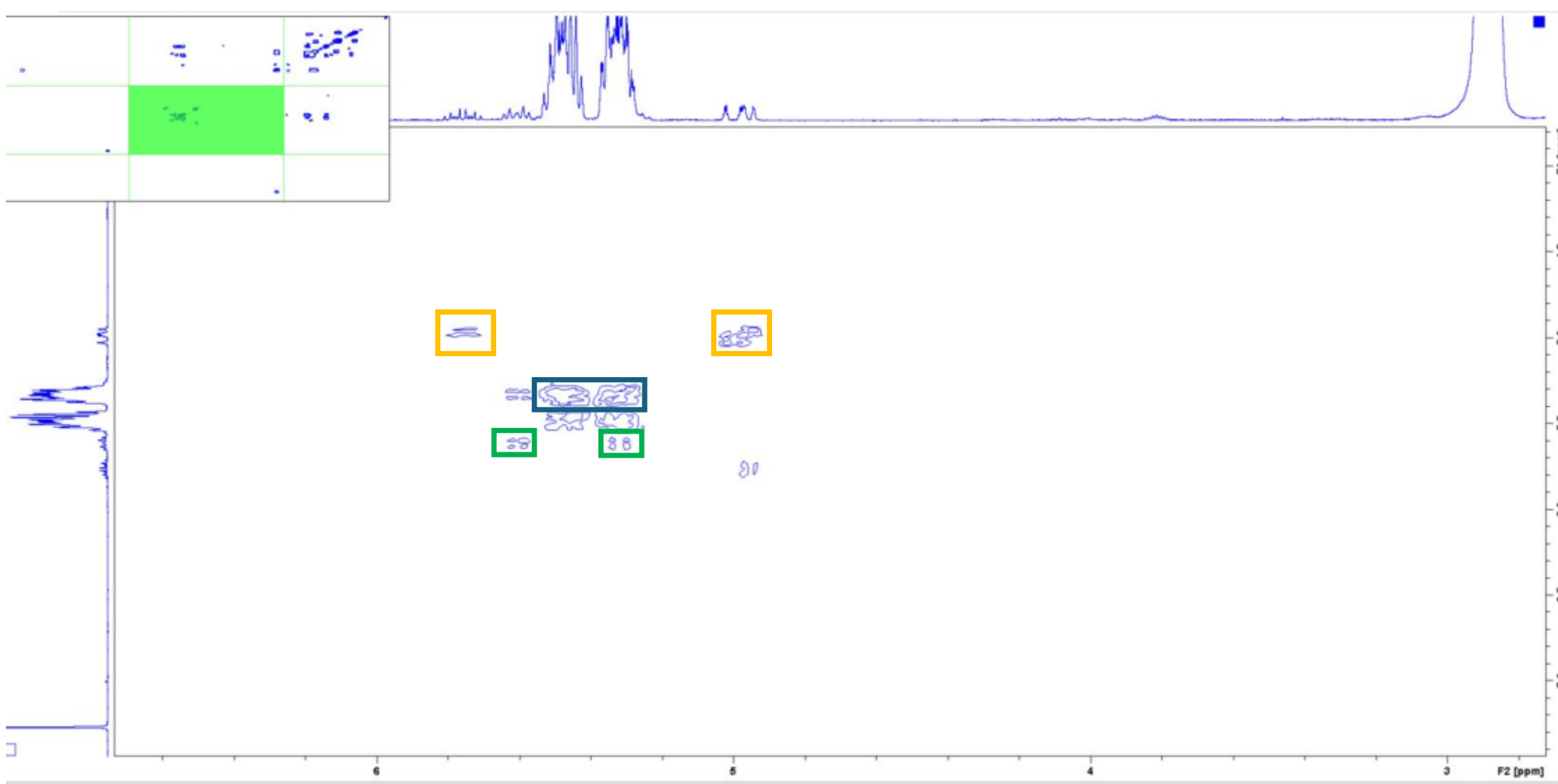

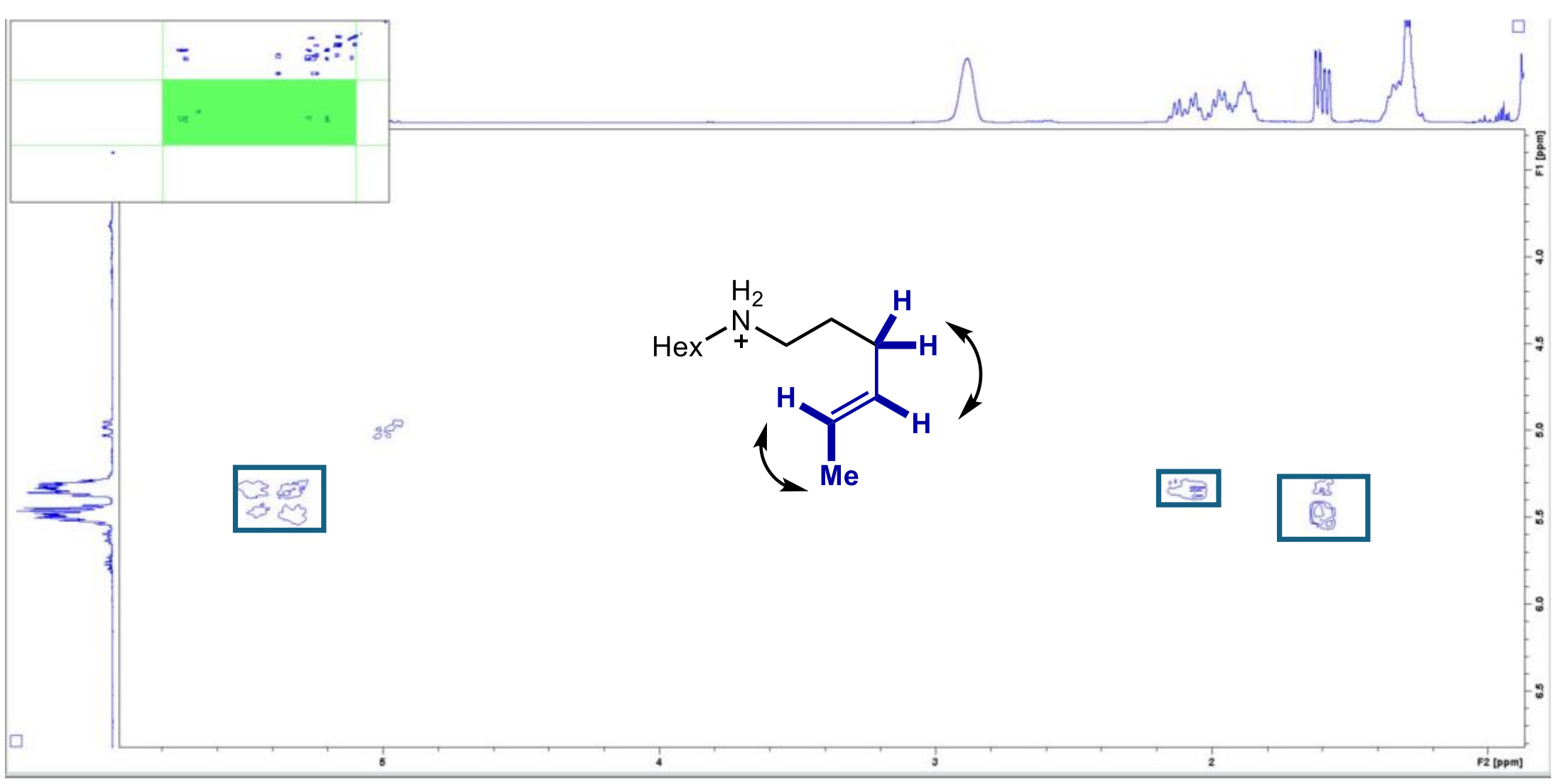

Alternative Synthesis of the Authentic Product

To confirm regioselectivity assignment, the major product was also prepared from commercially available (E)-tetrahydrogeraniol. The ^{1}H NMR spectrum was identical to that of the product obtained from the desaturation reaction. Furthermore, DEPT-135, HSQC, and HMBC analyses supported the assignment of the major product as the δ,ε-desaturated regioisomer. The terminal methyl group adjacent to the alkene exhibited a clear HMBC correlation to the olefinic carbon, with no corresponding correlation observed for any other carbons.

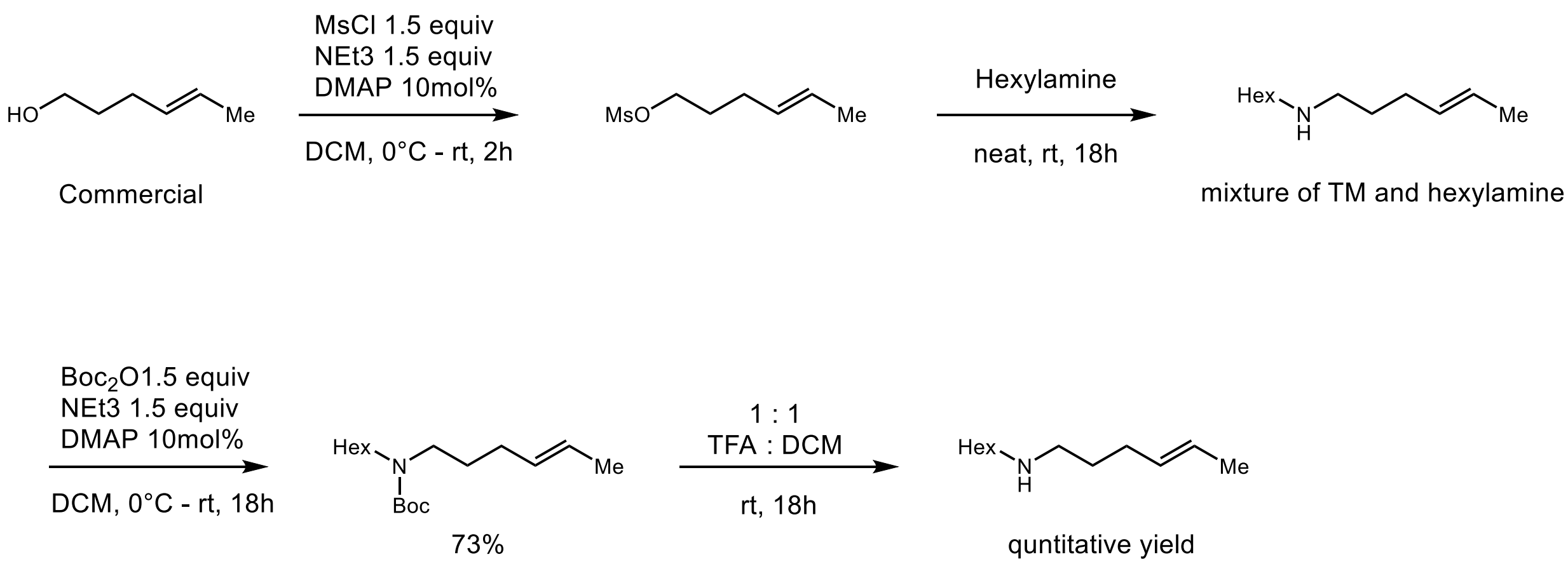

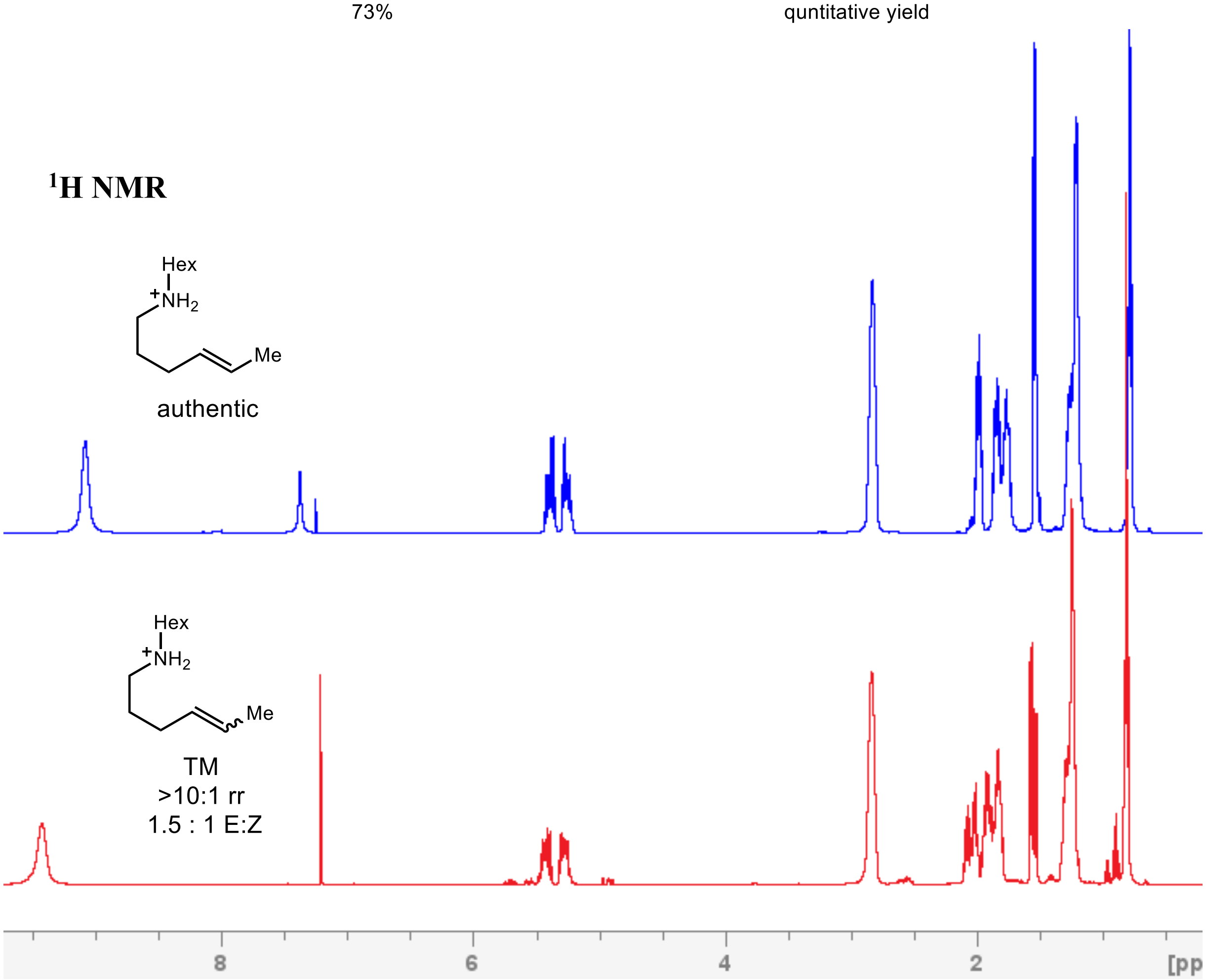

^{13}C NMR

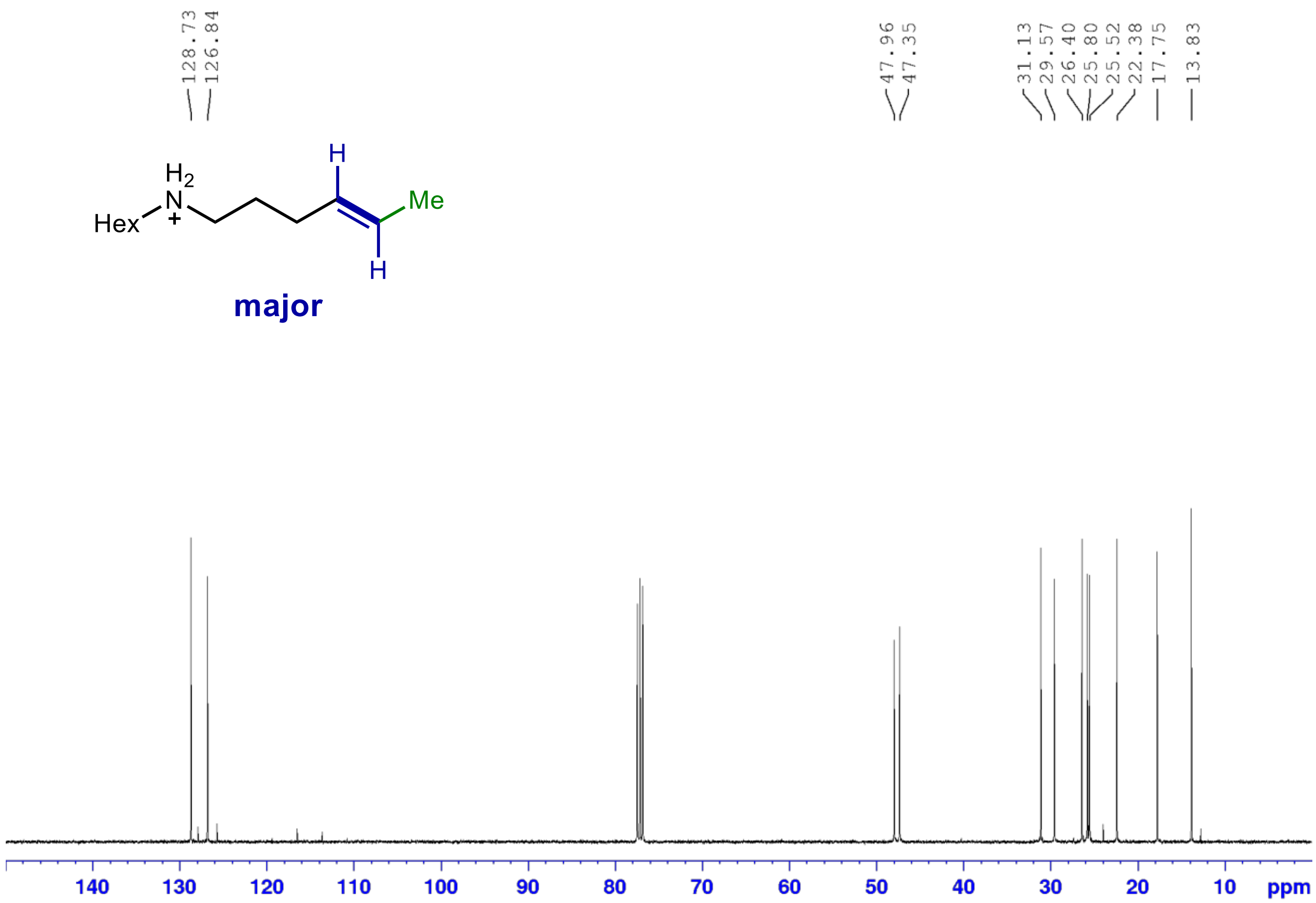

DEPT-135

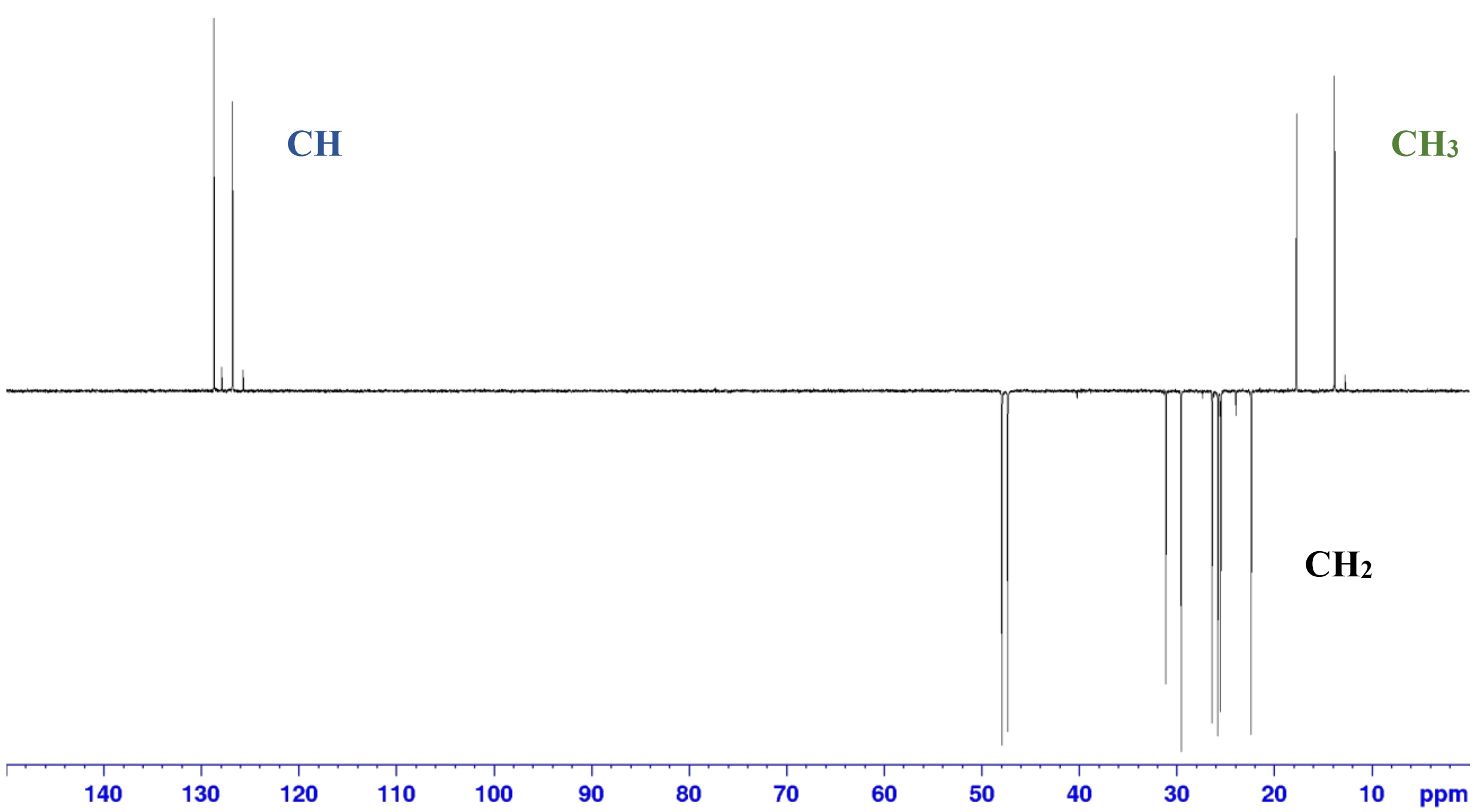

HMQC

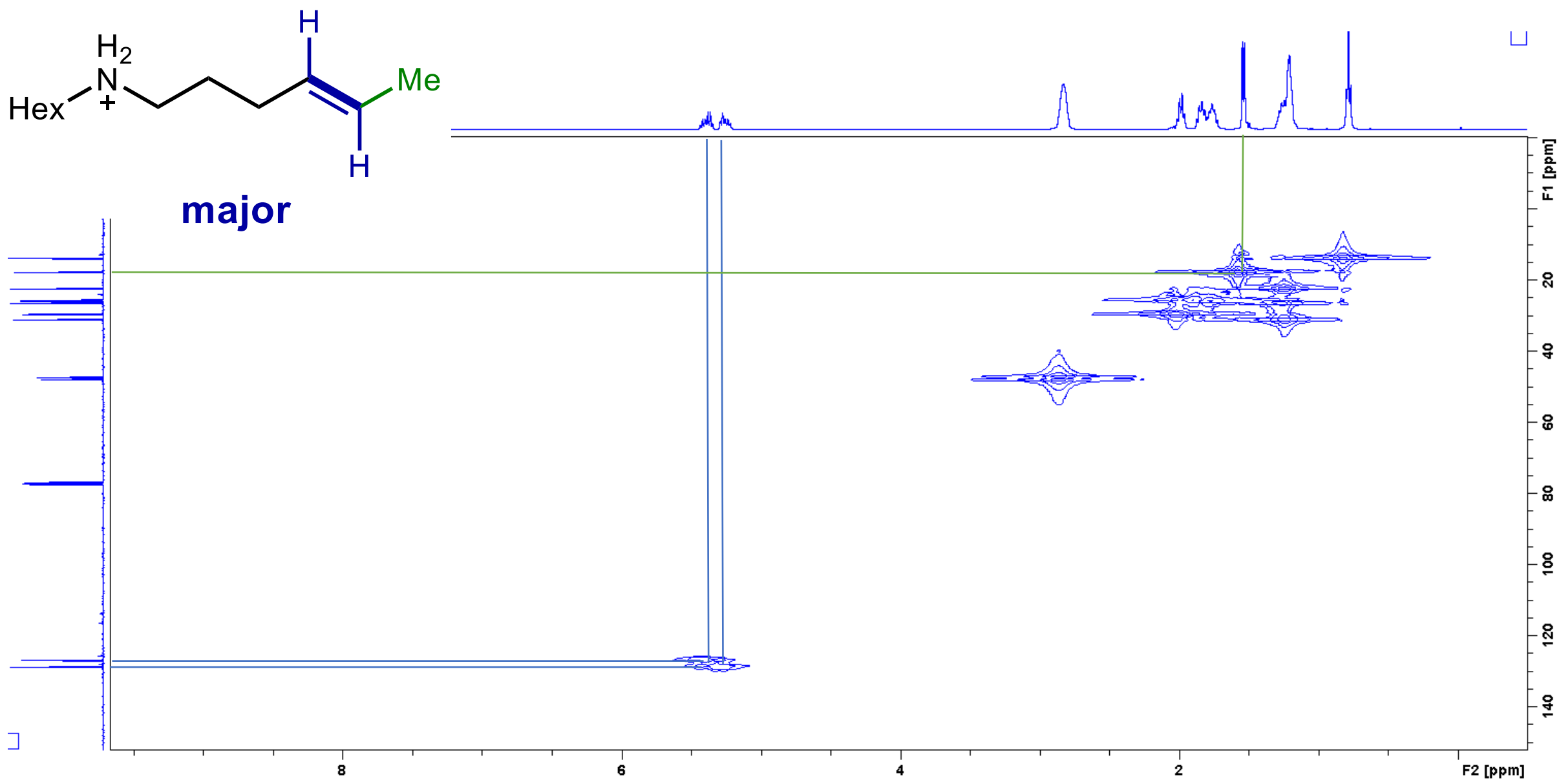

HMBC

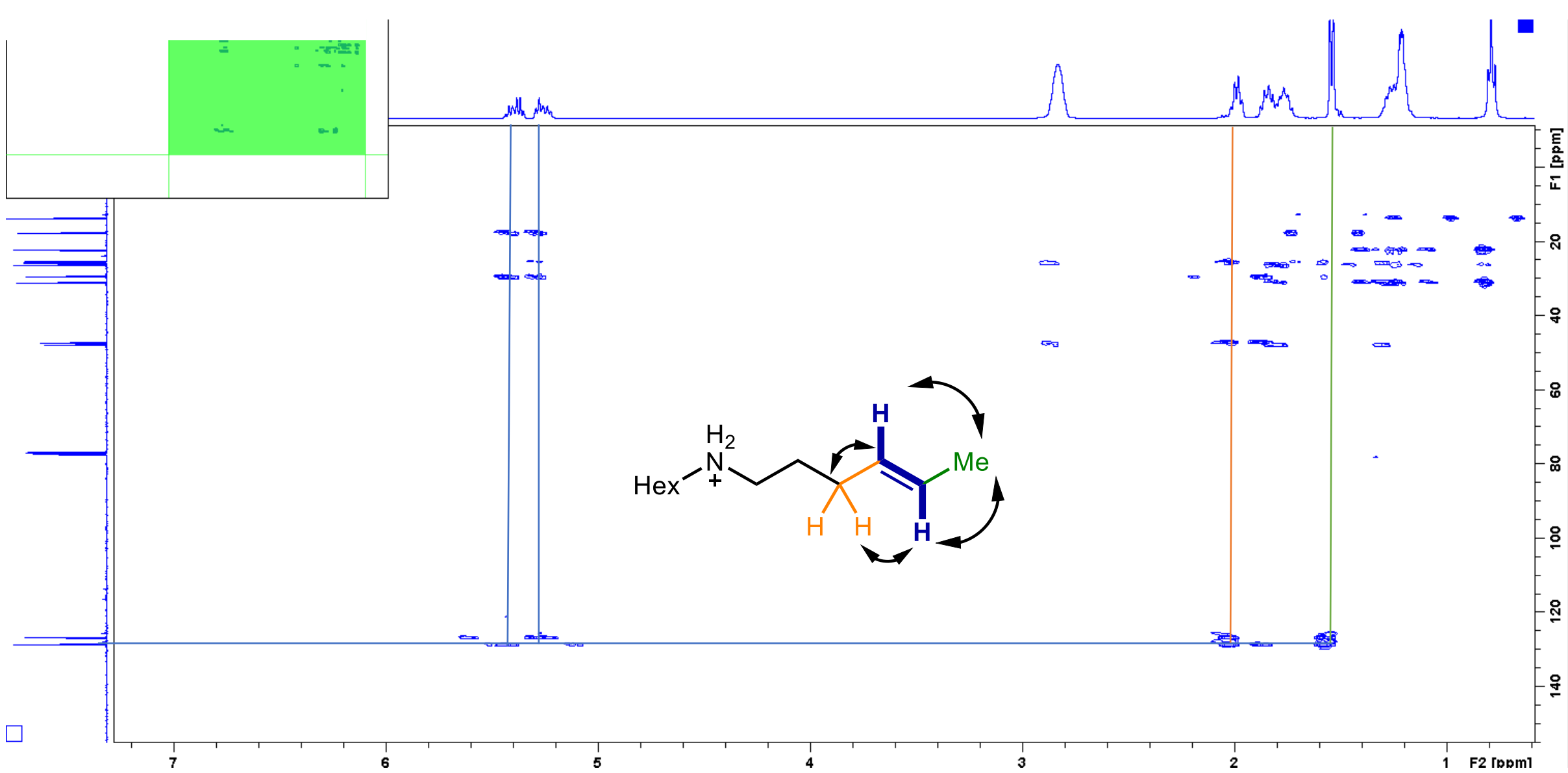

X. Intermolecular Desaturation

***P*-Cymene (28)**

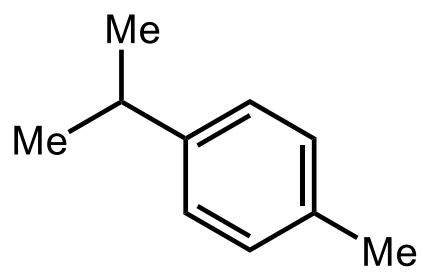

Prepared following **GP6** using gamma-terpene and **S20**. The crude product (97%) was purified on silica plug (100% petroleum ether) to afford a colorless oil. The analytical data is consistent with those reported for the commercially available compound. ^{1}H NMR (400 MHz, $CDCl_3$) δ 7.15 – 7.11 (m, 4H), 2.89 (sept, *J* = 5.2 Hz, 1H), 2.33 (s, 3H), 1.25 (d, *J* = 5.2 Hz, 6H).^{13}C NMR (100 MHz, $CDCl_3$) δ: 146.0, 135.3, 129.1, 126.4, 33.8, 24.2, 21.1.

4-(Dipropylamino)-2-isopropyl-5-methylenecyclohexan-1-ol (29)

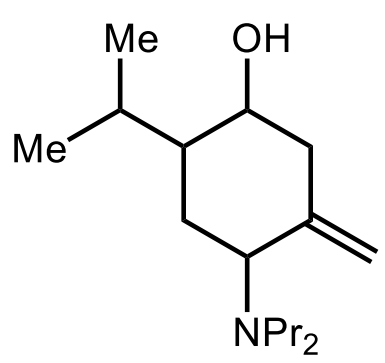

Prepared following **GP6** with menthol and **S20**. The crude product (50%) was purified on silica gel (10% EtOAC/Hexane) to afford a colorless oil. ^{1}H NMR (400 MHz, $CDCl_3$) δ 5.06 (br s, 1H), 4.91 (br s, 1H), 3.26 (td, *J* = 7.6, 3.0 Hz, 1H), 2.91 (dd, *J* = 24.4, 9.2 Hz, 2H), 2.49 – 2.34 (m, 4H), 2.01- 1.97 (m, 1H), 1.87 – 1.80 (m, 1H), 1.64 – 1.60 (m, 2H), 1.53 – 1.36 (m, 6H), 0.92 – 0.84 (m, 12H). ^{13}C NMR (100 MHz, $CDCl_3$) δ: 148.5, 117.0, 73.3, 61.1, 54.8, 51.9, 44.0, 31.5, 29.8, 22.2, 18.9, 11.9. IR (film) cm^{-1}: 2954, 2834, 1450, 1238, 1059, 705. HRMS (ESI-Orbitrap): *m/z* calculated for $C_{16}H_{32}NO$ $[M+H]^+$: 254.2478, found 254.2477.

***N,N*-dipropylcyclohex-2-en-1-amine (30)**

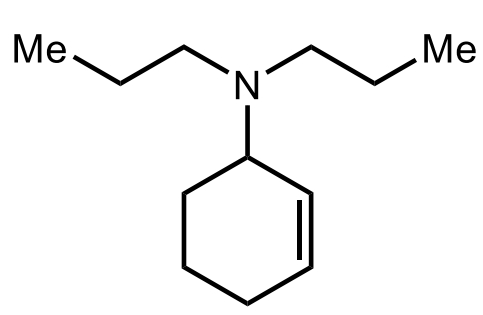

Prepared following **GP6** with cyclohexane and **S20**. The crude product (66%) was purified on silica plug (100% petroleum ether) to afford a colorless oil. ^{1}H NMR (400 MHz, $CDCl_3$) δ 5.79 – 5.74 (m, 1H), 5.64 – 5.59 (m, 1H), 3.39 (m, 1H), 2.45 – 2.29 (m, 4H), 2.00 – 1.92 (m, 2H), 1.83 – 1.76 (m, 2H), 1.49 – 1.38 (m, 6H), 0.86 (t, *J* = 5.6 Hz, 6H).^{13}C NMR (100 MHz, $CDCl_3$) δ: 131.4, 129.4, 57.2, 53.2, 25.5, 24.1, 22.6, 22.2, 12.1. IR (film) cm^{-1}: 1749, 1454, 1061, 1025, 708. HRMS (ESI-Orbitrap): *m/z* calculated for $C_{12}H_{24}N$ $[M+H]^+$: 182.1903, found 182.1903.

XI. Characterization of the Additional Substrates

Benzyl Protected Amines

Benzyl-protected amines were found to be less amenable to the standard reaction conditions. The benzylic position is susceptible to competing oxidation and/or elimination pathways, resulting in diminished yields of the desired products.

(E)-N-benzylhex-4-en-1-aminium (31)

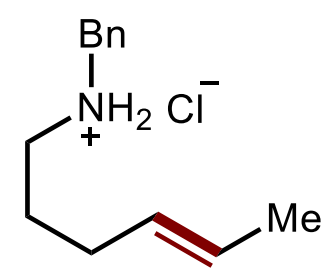

Prepared following **GP1** with **S21**. The crude product (32%, >10:1 r.r., E:Z 1.5:1) was recrystallized with EtOAc to afford a white solid. ^{1}H NMR (400 MHz, $CDCl_3$) 9.87 (br s, 2H), 7.60 – 7.58 (m, 2H), 7.40 – 7.38 (m, 3H), 5.47 – 5.37 (m, 1H), 5.30 – 5.23 (m, 1H), 4.01 (br s, 2H), 2.74 (br s, 2H), 2.07 – 2.00 (m, 2H), 1.91 – 1.89 (m, 2H), 1.57 – 1.53 (m, 3H). ^{13}C NMR (100 MHz, $CDCl_3$) δ: 130.5, 130.2, 129.5, 129.2, 128.8, 127.0, 50.6, 45.5, 29.6, 25.5, 17.9. IR (film) cm^{-1}: 2930, 1583, 1244, 1176, 965, 790, 707. HRMS (ESI-Orbitrap): *m/z* calculated for $C_{13}H_{20}N$ $[M]^+$: 190.1590, found 190.1590.

N-(2-cyanoethyl)pent-4-en-1-aminium (32)

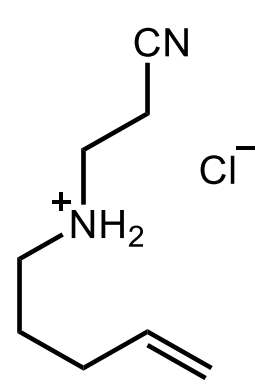

Prepared following **GP1** with **S22**. The crude product (52%, 4:1 r.r.) was recrystallized with EtOAc to afford a white solid. ^{1}H NMR (400 MHz, $CDCl_3$) δ 9.74 (br s, 2H), 5.81 – 5.71 (m, 1H), 5.12 – 5.04 (m, 2H), 3.32 (br s, 2H), 3.23 – 3.21 (m, 2H), 3.02 (br s, 2H), 2.21 – 2.16 (m, 2H), 2.01 – 1.95 (m, 2H). ^{13}C NMR (100 MHz, $CDCl_3$) δ: 136.0, 116.8, 116.6, 47.9, 43.3, 30.6, 25.0, 15.4. IR (film) cm^{-1}: 2934, 2223, 1642, 1467, 1288, 1047, 914, 789. HRMS (ESI-Orbitrap): *m/z* calculated for $C_7H_{13}N_2$ $[M]^+$: 125.1073, found 125.1074.

(E)-N-(3-hydroxypropyl)hex-4-en-1-aminium (33)

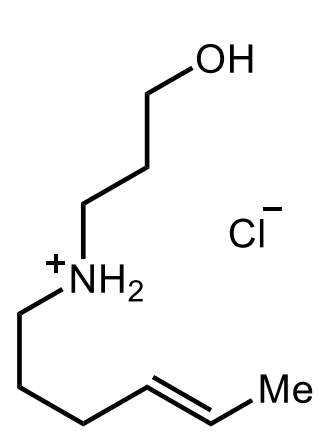

Prepared following **GP1** with **S23**. The crude product (22%, >10:1 r.r., E:Z 1.5:1) was recrystallized with EtOAc to afford a white solid. ^{1}H NMR (400 MHz, $CDCl_3$) δ 9.03 (br s, 2H), 5.43 – 5.42 (m, 1H), 5.36 – 5.27 (m, 1H), 3.79 (t, *J* = 6.0 Hz, 2H), 3.13 – 3.12 (m, 2H), 2.93 – 2.92

(m, 2H), 2.07 – 2.02 (m, 2H), 1.62 – 1.56 (m, 3H), 1.36 – 1.34 (m, 2H), 1.28 – 1.27 (m, 3H). ^{13}C NMR (100 MHz, $CDCl_3$) δ: 128.8, 127.1, 59.6, 47.3, 46.3, 29.6, 25.6, 17.9. IR (film) cm^{-1}: 3372, 1629, 1465, 1274, 1065, 970, 715. HRMS (ESI-Orbitrap): *m/z* calculated for $C_9H_{20}NO$ $[M]^+$: 158.1539, found 158.1539.

(E)-N-(3-(benzoyloxy)propyl)hex-4-en-1-aminium (34)

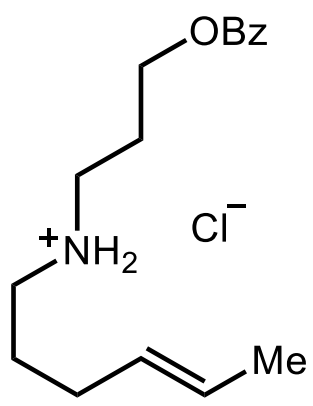

Prepared following **GP1** with **S24**. The crude product (66%, >10:1 r.r., E:Z 1.5:1) was recrystallized with EtOAc to afford a white solid. ^{1}H NMR (400 MHz, $CDCl_3$) δ 9.64 (br s, 2H), 8.00 – 7.97 (m, 2H), 7.55 – 7.51 (m, 1H), 7.42 – 7.38 (m, 2H), 5.51 – 5.40 (m, 1H), 5.34 – 5.25 (m, 1H), 4.40 (t, *J* = 6.0 Hz, 2H), 3.11 (br s, 2H), 2.94 (br s, 2H), 2.45 – 2.39 (m, 2H), 2.14 – 2.04 (m, 2H), 1.99 – 1.93 (m, 2H), 1.59 – 1.54 (m, 3H). ^{13}C NMR (100 MHz, $CDCl_3$) δ: 166.4, 133.3, 129.68, 129.67, 128.7, 128.5, 127.1, 61.9, 47.7, 45.3, 29.7, 25.7, 24.0, 17.9. IR (film) cm^{-1}: 2935, 2522, 1717, 1451, 1271, 1113, 967, 709. HRMS (ESI-Orbitrap): *m/z* calculated for $C_{16}H_{24}NO_2$ $[M]^+$: 262.1802, found 262.1801.

(E)-N-(3-((4-(trifluoromethyl)benzoyl)oxy)propyl)hex-4-en-1-aminium (35)

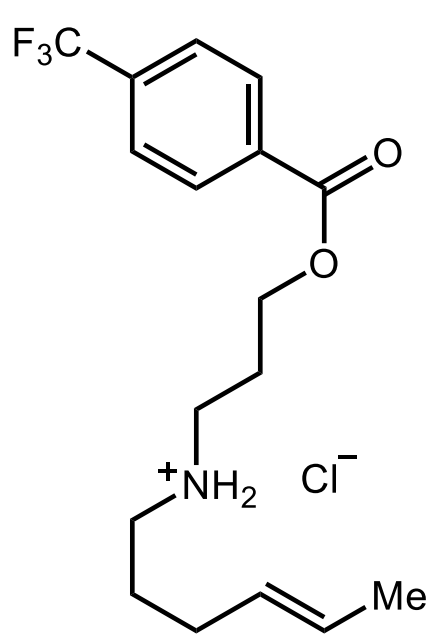

Prepared following **GP1** with **S25**. The crude product (73%, >10:1 r.r., E:Z 1.5:1) was recrystallized with EtOAc to afford a white solid. ^{1}H NMR (400 MHz, $CDCl_3$) δ 9.68 (br s, 2H), 8.10 (d, *J* = 8.0 Hz, 2H), 7.67 (d, *J* = 8.4 Hz, 2H), 5.51 – 5.40 (m, 1H), 5.34 – 5.25 (m, 1H), 4.45 (t, *J* = 6.0 Hz, 2H), 3.11 (br s, 2H), 2.94 (br s, 2H), 2.48 – 2.41 (m, 2H), 2.15 – 2.04 (m, 2H), 2.01 – 1.91 (m, 2H), 1.59 – 1.54 (m, 3H). ^{13}C NMR (100 MHz, $CDCl_3$) δ: 165.3, 137.6, 134.8 (q, *J* = 28 Hz), 132.9, 130.2, 128.7, 127.2, 125.6 (q, *J* = 4 Hz), 123.6 (q, *J* = 270 Hz), 62.4, 47.8, 45.2, 29.7, 24.1, 17.9. ^{19}F (377 MHz, $CDCl_3$) δ -63.2. IR (film) cm^{-1}: 1720, 1411, 1326, 1017, 861, 774, 703. HRMS (ESI-Orbitrap): *m/z* calculated for $C_{17}H_{23}F_3NO_2$ $[M]^+$: 330.1675, found 330.1675.

(E)-N-(3-(2-(4-methoxyphenyl)acetoxy)propyl)hex-4-en-1-aminium (36)

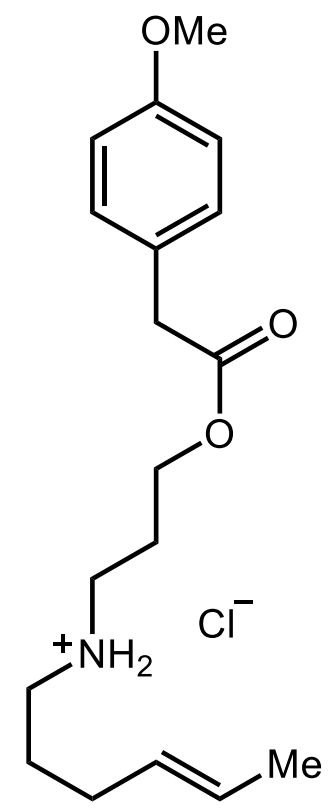

Prepared following **GP1** with **S26**. The crude product (56%, >10:1 r.r., E:Z 1.1:1) was recrystallized with EtOAc to afford a white solid. ^{1}H NMR (400 MHz, $CDCl_3$) δ 9.47 (br s, 2H), 7.17 (d, *J* = 8.4 Hz, 2H), 6.85 (d, *J* = 8.4 Hz, 2H), 5.54 – 5.43 (m, 1H), 5.37 – 5.27 (m, 1H), 4.18 (t, *J* = 5.6 Hz, 2H), 3.78 (s, 3H), 3.55 (s, 2H), 2.48 – 2.41 (m, 2H), 2.91 (br s, 2H), 2.85 (br s, 2H), 2.26 – 2.21 (m, 2H), 2.14 – 2.03 (m, 2H), 1.94 – 1.87 (m, 2H), 1.63 – 1.57 (m, 3H). ^{13}C NMR (100 MHz, $CDCl_3$) δ: 172.0, 158.9, 130.4, 128.8, 127.2, 126.0, 114.2, 61.6, 55.4, 47.6, 45.0, 40.4, 29.7, 25.5, 24.0, 17.9. IR (film) cm^{-1}: 1731, 1511, 1244, 1152, 1030, 819, 722 HRMS (ESI-Orbitrap): *m/z* calculated for $C_{18}H_{28}NO_3$ $[M]^+$: 306.2064, found 306.2065.

(E)-N-(3-((4-(5-(2-fluorophenyl)-1,2,4-oxadiazol-3-yl)benzoyl)oxy)propyl)hex-4-en-1-aminium (37)

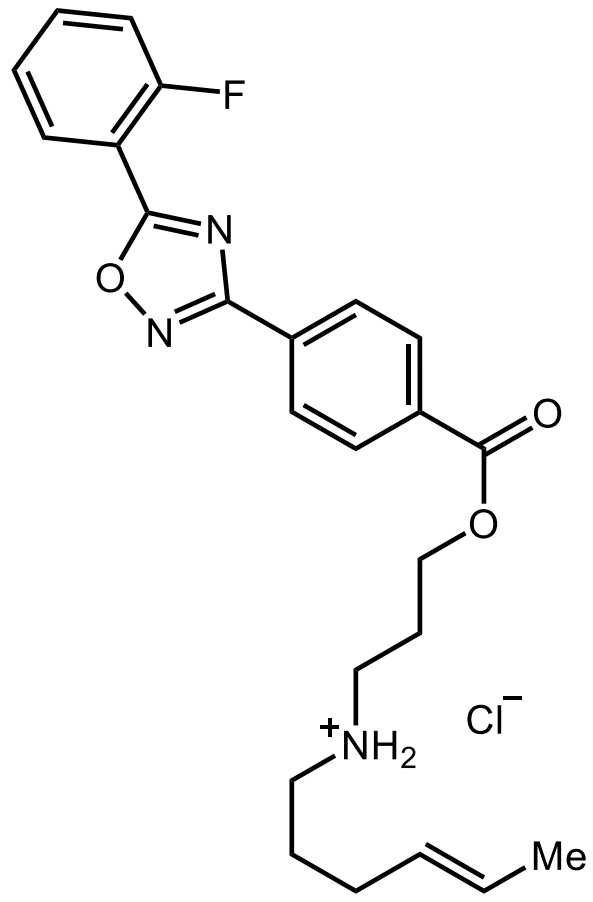

Prepared following **GP1** with **S27**. The crude product (57%, >10:1 r.r., E:Z 1.2:1) was recrystallized with EtOAc to afford a white solid. ^{1}H NMR (400 MHz, $CDCl_3$) δ 9.68 (br s, 2H), 8.76 – 8.75 (m, 1H), 8.35 - 8.32 (m, 1H), 8.23 – 8.19 (m, 1H), 8.16 – 8.14 (m, 1H), 7.62 – 7.55 (m, 2H), 7.35 – 7.31 (m, 1H), 7.29 – 7.24 (m, 1H), 5.51 – 5.44 (m, 1H), 5.37 – 5.28 (m, 1H), 4.51 (t, *J* = 5.2 Hz, 2H), 3.18 (br s, 2H), 2.99 (br s, 2H), 2.51 – 2.48 (m, 2H), 2.18 – 2.07 (m, 2H), 2.03 – 1.95 (m, 2H), 1.60 – 1.55 (m, 3H). ^{13}C NMR (100 MHz, $CDCl_3$) δ: 173.2, 167.9, 165.8, 160.9 (d, *J* = 259 Hz), 134.9 (d, *J* = 9 Hz), 132.1 (d, *J* = 26 Hz), 131.1, 130.6, 129.3, 128.8, 127.8, 127.2, 124.8 (d, *J* = 4 Hz), 117.4 (d, *J* = 21 Hz), 112.7 (d, *J* = 12 Hz), 62.3, 47.8, 45.3, 29.7, 25.8, 17.9. ^{19}F (377 MHz, $CDCl_3$) δ -108.2. IR (film) cm^{-1}: 1721, 1621, 1464, 1259, 1109, 825, 749, 722. HRMS (ESI-Orbitrap): *m/z* calculated for $C_{24}H_{27}FN_3O_3$ $[M]^+$: 424.2031, found 424.2029.

XII. Computational Details

Computational Methods

Initial guess structures were generated in Spartan '18.[68] An initial structure was optimized with MMFF,[69] and a Monte-Carlo (MC) conformational distribution search was conducted at the same level. The structure with the largest Boltzmann weight fraction was used as an initial guess structure, and a geometry optimization was performed at the uωB97M-V/def2-TZVP/SMD(Trifluoroethanol) level of theory[70] using QChem v6.[71] A subsequent frequency calculation was performed to ensure that all optimized intermediate structures had no imaginary frequencies and energies were refined by taking the single point energy with uωB97M-V/def2-QZVPPD/SMD(Trifluoroethanol).

Transition state structures were estimated using the freezing string method in QChem v6, and a subsequent transition state optimization was conducted using the uωB97M-V/def2-TZVP/SMD(Trifluoroethanol) level of theory. A frequency calculation was performed to confirm that transition state structures had exactly one imaginary frequency and energies were refined by taking the single point energy with uωB97M-V/def2-QZVPPD/SMD(Trifluoroethanol).

The gas phase electrophilicity (ω) of radicals was calculated using the uB3LYP-D3/6-311+G** level[72-73] according to the method previously described by Garwood *et al*[36] (equation 1), where *I* and *A* were computed using the ΔSCF method.[74]

$$Eq.1)\ \omega = \frac{\mu^2}{2\eta} = \frac{(I + A)}{8\,(I - A)}$$

Radical Polarity

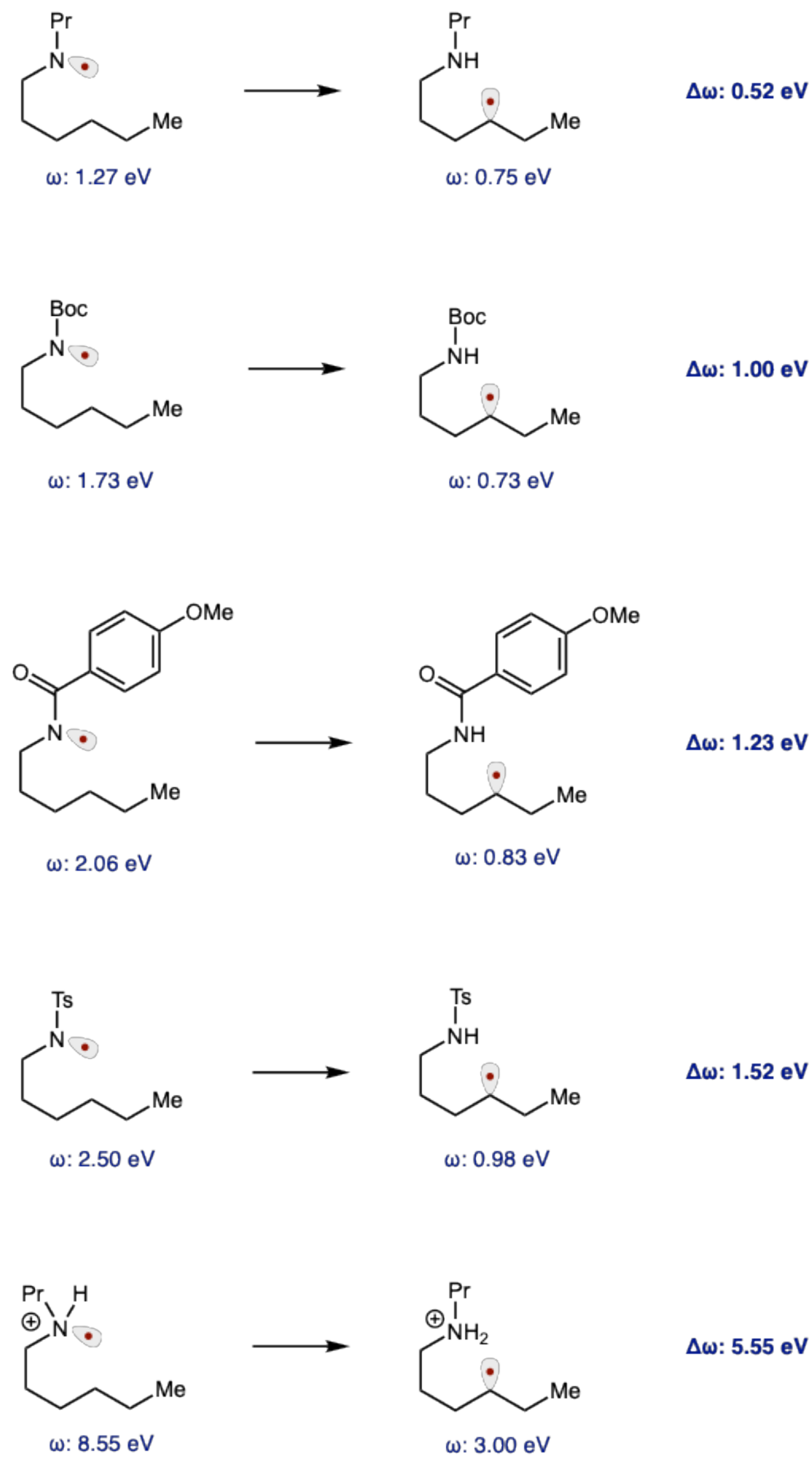

Figure S2: Polarity (ω, eV) of N-centered and δ C-centered radicals, uB3LYP-D3/6-311+G**

Bond Dissociation Enthalpies

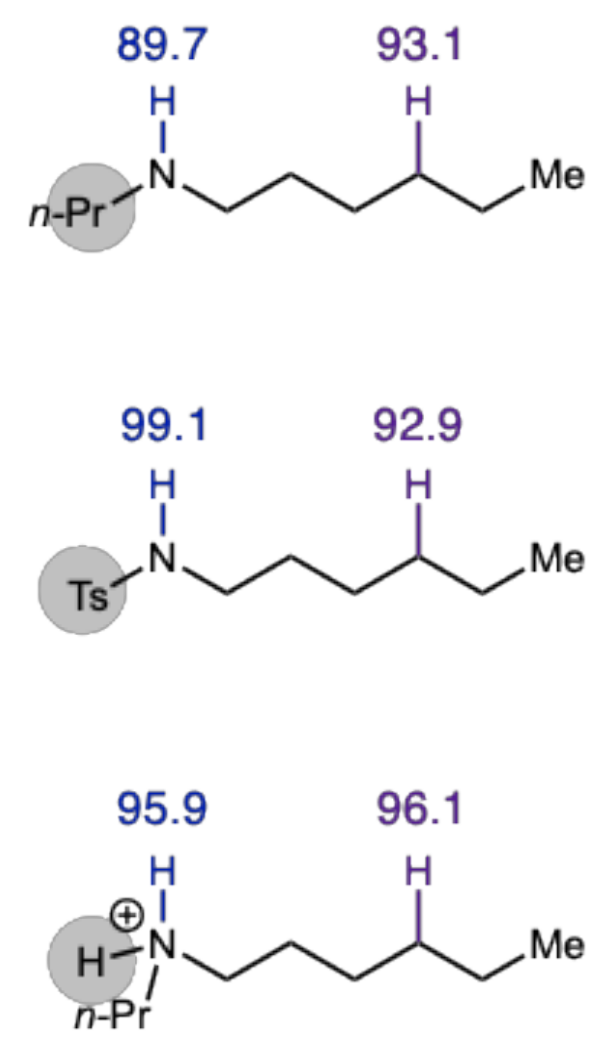

Figure S3: Bond dissociation enthalpies (kcal/mol) of N-H and δ C-H bonds

uωB97M-V/def2-QZVPPD //uωB97M-V/def2-TZVP/SMD(trifluoroethanol)

Reaction Energy Profiles

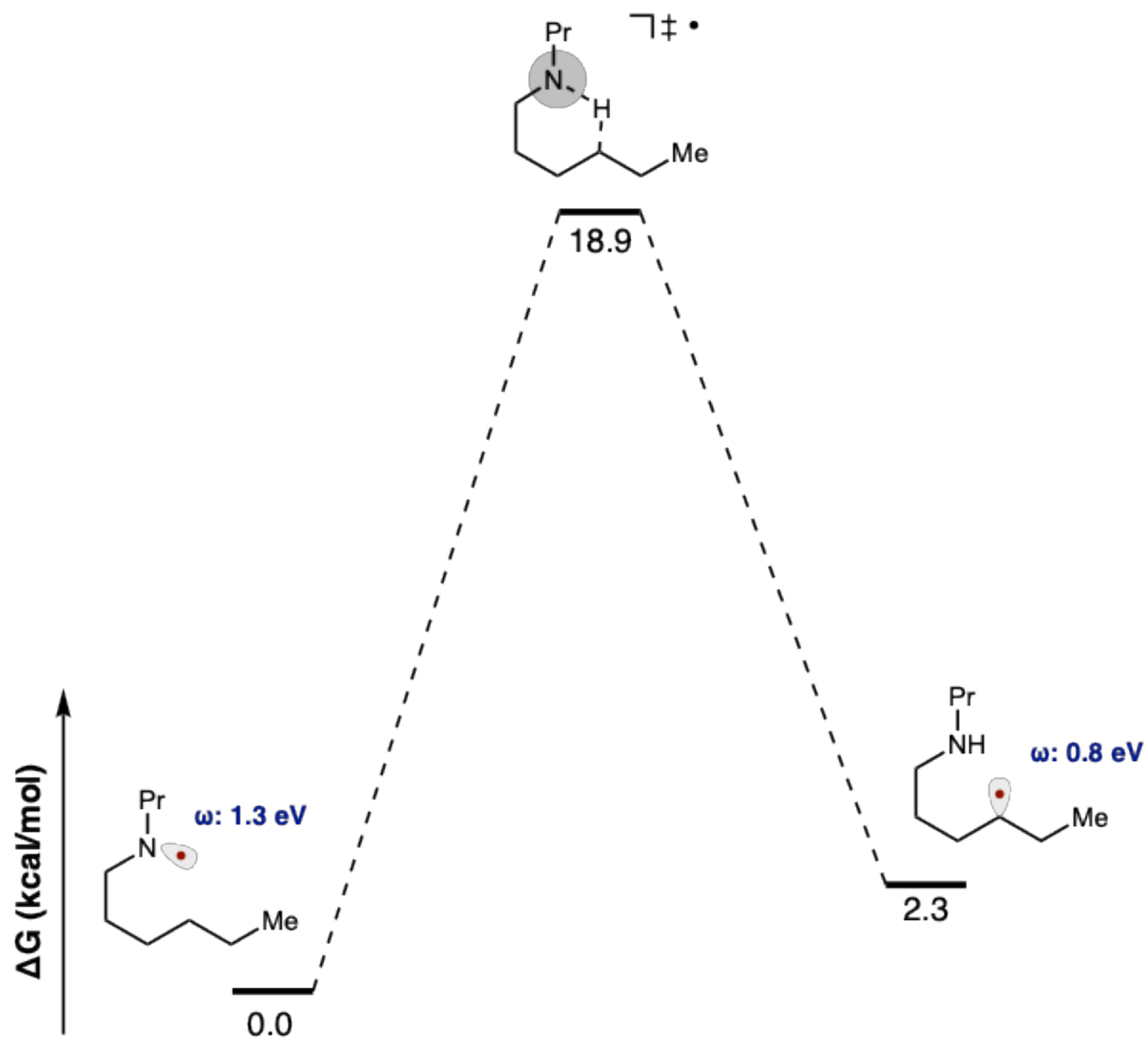

Figure S4: 1,5-HAT via aminyl radical

uωB97M-V/def2-QZVPPD //uωB97M-V/def2-TZVP/SMD(trifluoroethanol)

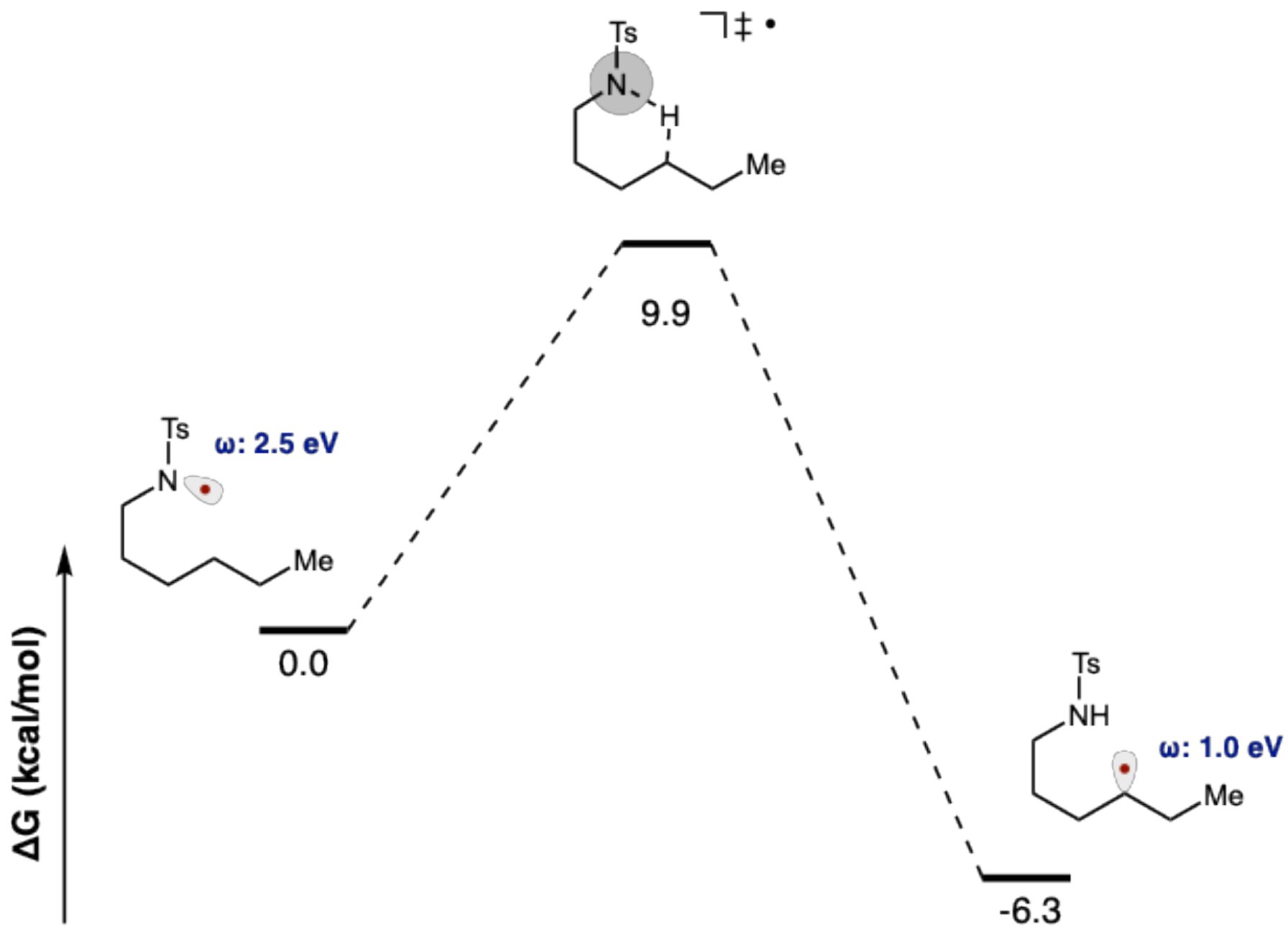

Figure S5: 1,5-HAT via sulfonamido radical

uωB97M-V/def2-QZVPPD //uωB97M-V/def2-TZVP/SMD(trifluoroethanol)

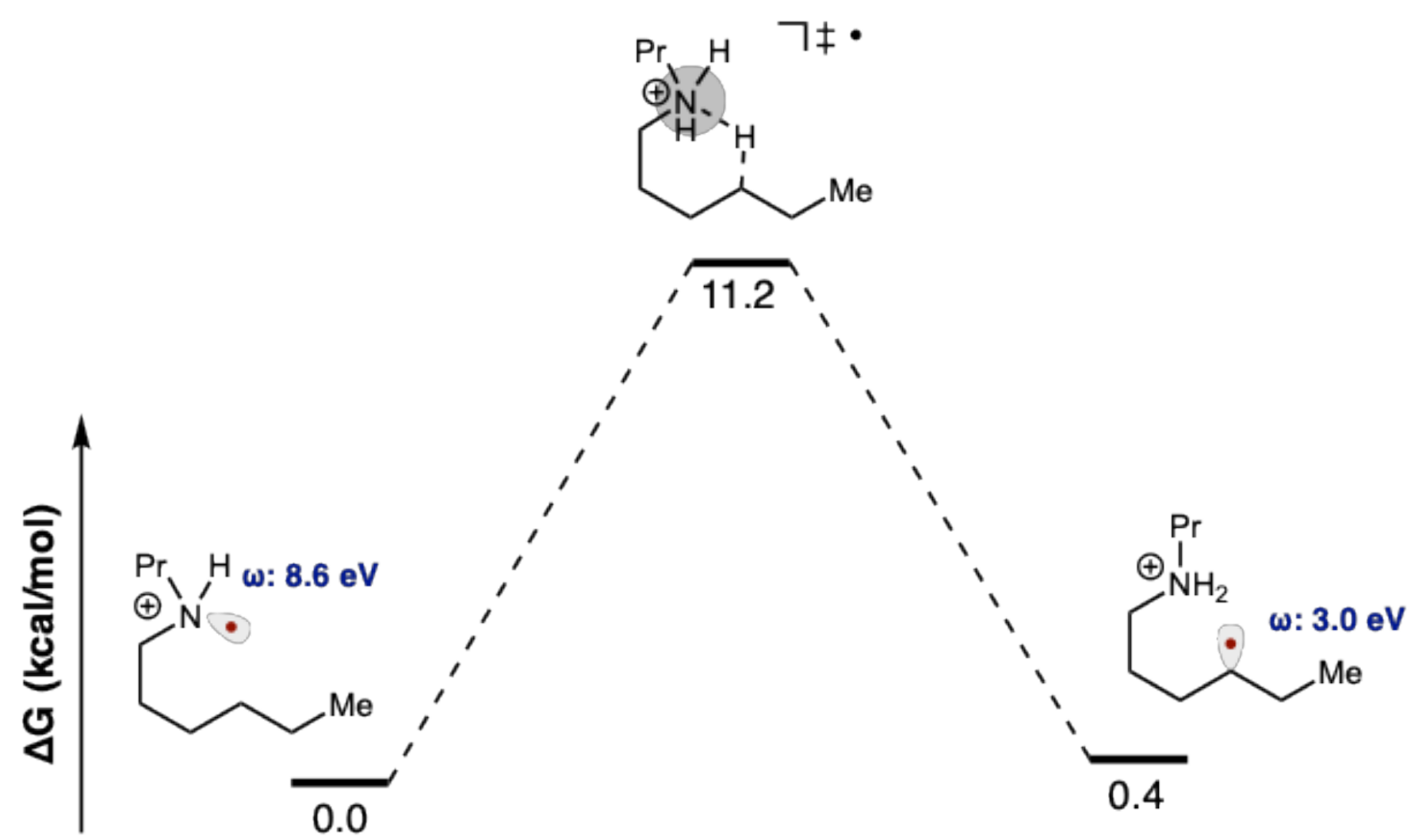

Figure S6: 1,5-HAT via aminium radical cation

uωB97M-V/def2-QZVPPD// uωB97M-V/def2-TZVP/SMD(trifluoroethanol)

Polarity Geometries and Energies

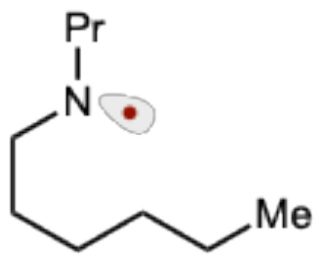

Propylhexylaminyl

EE (radical): -409.84703 hartree
EE (N-1): -409.5326 hartree
EE (N+1): -409.8656 hartree
ω: 1.27 eV

N 1.0857732 0.8428290 0.8870250
C 0.6160560 2.1454318 0.4747263
C -0.4536552 2.1018495 -0.6492109
C -1.7586476 1.3820787 -0.2804725
C -1.6559796 -0.1471946 -0.2283661
C -2.9845421 -0.8326987 0.1120407
H 1.4502407 2.7740043 0.1199135
H 0.1725192 2.6365195 1.3475916
H -0.0246658 1.6488333 -1.5503006
H -0.6732434 3.1438912 -0.9061847
H -2.5251888 1.6597107 -1.0149868
H -2.1167549 1.7574035 0.6869201
H -0.8988806 -0.4335104 0.5091880
H -1.2968974 -0.5165305 -1.1990109
H -3.3372463 -0.4658398 1.0837003
H -3.7471804 -0.5372946 -0.6198309
C 2.0868421 0.2909963 0.0109421
C 2.4206047 -1.1607771 0.3557166
H 2.9979499 0.9143623 0.0819062
H 1.7729061 0.3655960 -1.0449744
H 2.7174414 -1.2083966 1.4083786
C 3.5207406 -1.7421498 -0.5361681
H 1.5073163 -1.7583846 0.2659543
H 4.4529387 -1.1757197 -0.4395625
H 3.2305034 -1.7216003 -1.5918676
H 3.7367409 -2.7808313 -0.2731631
C -2.8739368 -2.3602126 0.1496787
H -2.1448898 -2.6827957 0.8998064
H -2.5468083 -2.7544849 -0.8182024
H -3.8321071 -2.8286764 0.3922286

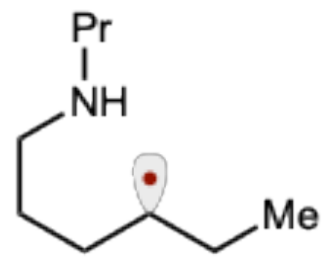

6-(propylamino)hexan-3-yl

EE (radical): -409.83984 hartree
EE (N-1): -409.57359 hartree
EE (N+1): -409.82387 hartree
ω: 0.75 eV

N 1.8316983 -0.3741708 -0.0571554
C 3.0976204 0.3446880 0.0638383
H 3.1754132 0.9062541 1.0143646
H 3.1331989 1.0932991 -0.7375031
C 4.2946437 -0.5964192 -0.0674004
H 4.2476684 -1.3531809 0.7269026
H 4.2100229 -1.1379984 -1.0150102
C 5.6371639 0.1370358 0.0076283
H 5.7426502 0.6820040 0.9511843
H 6.4750434 -0.5611692 -0.0669992
H 5.7366739 0.8621830 -0.8062182
C 0.6505047 0.4760522 0.0661156
H 0.6378428 1.0434385 1.0160388
H 0.6913749 1.2228003 -0.7364576
C -0.6383381 -0.3345190 -0.0609939
H -0.6119161 -0.8882001 -1.0052408
H -0.6776833 -1.0844255 0.7402090
C -1.9014773 0.5319930 0.0005928
H -1.8578036 1.2908506 -0.8016879
H -1.9026916 1.1199600 0.9330213
C -3.1761945 -0.2373440 -0.1047471
H -3.1354030 -1.2946907 -0.3535149
C -4.4995877 0.4523307 -0.1077636
H -4.4933999 1.2632614 0.6352356
H -4.6493712 0.9644626 -1.0759260
C -5.6901564 -0.4794693 0.1514173
H -6.6373115 0.0652690 0.1108746
H -5.6121681 -0.9495187 1.1359006
H -5.7322768 -1.2775725 -0.5962577
H 1.7931771 -1.1139189 0.6390482

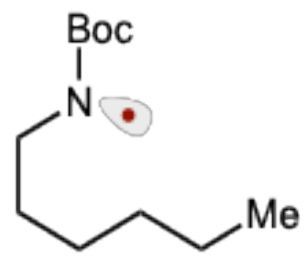

[(tert-butoxy)carbonyl]hexylaminyl

EE (radical): -637.81299 hartree
EE (N-1): -637.46881 hartree
EE (N+1): -637.8587 hartree
ω: 1.73 eV

N 1.0072741 -2.0458196 -0.8993829
C 0.0119266 -2.7894765 -0.1550402
H 0.5583135 -3.4007117 0.5809106
H -0.4797036 -3.4754156 -0.8494562
C -1.0197536 -1.9167783 0.5847221
H -0.4957749 -1.2857915 1.3126645
H -1.6688991 -2.5810207 1.1664768
C -1.8663556 -1.0442524 -0.3506641
H -2.4311973 -1.6914724 -1.0339829
H -1.2047431 -0.4382404 -0.9777467
C -2.8306806 -0.1261790 0.4032300
H -3.4893419 -0.7268956 1.0444838
H -2.2547757 0.5175341 1.0821818
C -3.6846201 0.7531150 -0.5189788
H -3.0234851 1.3452865 -1.1636472
H -4.2664795 0.1108202 -1.1916337
C -4.6312414 1.6837556 0.2440650
H -4.0749232 2.3705856 0.8904660
H -5.2378601 2.2866150 -0.4373086
H -5.3142823 1.1131674 0.8817614
C 1.6909601 -1.0767261 -0.1663606
O 2.4963394 -1.3572855 0.6964527
O 1.3733737 0.1457690 -0.6047865
C 1.9665972 1.3697209 -0.0123001
C 3.4767255 1.3777685 -0.2542553
H 3.6907020 1.2459335 -1.3177371
H 3.8880075 2.3418540 0.0577691
H 3.9742401 0.5877109 0.3064408
C 1.2748849 2.4807102 -0.8010842
H 1.4927698 2.3890831 -1.8671274
H 1.6248445 3.4565471 -0.4555488
H 0.1922896 2.4316171 -0.6630466
C 1.6063668 1.4495874 1.4728349
H 1.9224408 2.4182789 1.8687168
H 0.5245467 1.3668144 1.6051364
H 2.0958293 0.6630971 2.0455654

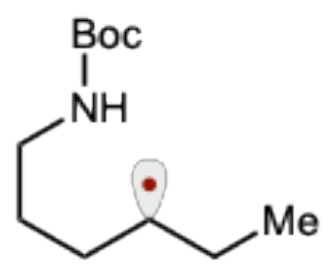

6-{[(tert-butoxy)carbonyl]amino}hexan-3-yl

EE (radical): -637.82554 hartree
EE (N-1): -637.5708 hartree
EE (N+1): -637.81149 hartree
ω: 0.73 eV

N -0.2760879 -1.8491840 0.4199995
C -1.4898119 -2.4111782 -0.1520784
H -1.5152126 -3.4813903 0.0784445
H -1.3988627 -2.3116467 -1.2350461
C -2.7895896 -1.7522259 0.3288310
H -2.8684755 -1.8438058 1.4201066
H -3.6219536 -2.3324218 -0.0863958
C -2.9435174 -0.2766233 -0.0794452
H -4.0042838 0.0008032 0.0553558
H -2.7538690 -0.1762995 -1.1565596
C -2.0744505 0.6786451 0.6707089
H -1.9082393 0.5018872 1.7297194
H -0.1141037 -1.9317024 1.4116786
C -1.6445424 1.9733858 0.0642384
H -0.9180685 2.4667341 0.7169952
H -1.1265275 1.7694176 -0.8829015
C -2.8111356 2.9494205 -0.2184231
H -3.3358327 3.2135377 0.7044744
H -2.4433160 3.8720916 -0.6773972
H -3.5398266 2.5040205 -0.9013732
C 0.5044182 -0.9505864 -0.2451189
O 0.3725883 -0.6417684 -1.4136547
O 1.4582536 -0.4786949 0.5973609
C 2.5839519 0.3319264 0.1024359
C 3.3800992 -0.4632950 -0.9365410
H 2.7929506 -0.6374931 -1.8375013
H 4.2813392 0.0929246 -1.2088651
H 3.6891324 -1.4266695 -0.5222032
C 2.0837401 1.6677658 -0.4522625
H 1.4767417 1.5223451 -1.3438386
H 2.9412932 2.2971086 -0.7061330
H 1.4875218 2.1909230 0.2983969
C 3.4160121 0.5515565 1.3666375
H 4.2981231 1.1520054 1.1314612
H 2.8312702 1.0762754 2.1256811
H 3.7450354 -0.4033990 1.7823582

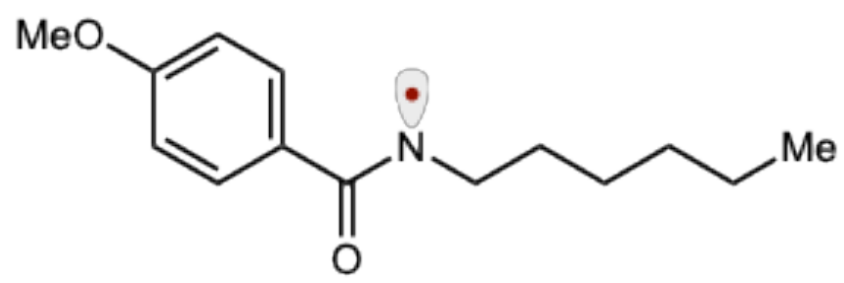

N-hexyl(4-methoxyphenyl)amidyl
EE (radical): -750.92849 hartree
EE (N-1): -750.6087 hartree
EE (N+1): -750.99812 hartree
ω: 2.06 eV

N -0.8869338 -2.2673180 -0.8935556
C -2.2312081 -2.5119444 -0.4333650
C -3.5565059 2.4472342 -0.2915967
C -2.8564356 -0.0162991 -0.3563222
C -3.4818329 1.1158114 0.4641875
C -2.8011769 -1.3454553 0.4035138
C -4.1859929 3.5735725 0.5337599
H -2.8640912 -2.7055127 -1.3030450
H -4.1318991 2.3047294 -1.2148535
H -2.5473546 2.7409698 -0.6054740
H -3.4257150 -0.1532932 -1.2850881
H -1.8437598 0.2732449 -0.6600501
H -4.4915822 0.8215760 0.7800916
H -2.9043745 1.2560543 1.3876738
H -2.1990787 -1.2347822 1.3122024
H -3.8078991 -1.6321308 0.7279339
H -4.2339345 4.5075798 -0.0330290
H -3.6091341 3.7672810 1.4440737
H -2.2114638 -3.4137447 0.1951745
H -5.2055834 3.3164825 0.8385592
C 0.1092638 -2.2534570 0.0798787
O 0.1474685 -3.1056455 0.9629139
C 1.1402100 -1.1990664 -0.0792300
C 3.0944771 0.7991167 -0.2774187
C 1.2031123 -0.3732122 -1.2150906
C 2.0665666 -1.0046454 0.9495129
C 3.0397293 -0.0161608 0.8620726
C 2.1694471 0.6124607 -1.3156060
H 0.5004874 -0.5291623 -2.0240650
H 2.0148591 -1.6438491 1.8227055
H 3.7409099 0.1123067 1.6752400
H 2.2386180 1.2479700 -2.1900476
O 4.0033565 1.7894602 -0.4686237
C 4.9935040 2.0190736 0.5291343
H 5.6168883 1.1319903 0.6817734
H 5.6087252 2.8332952 0.1510729
H 4.5383699 2.3175329 1.4791353

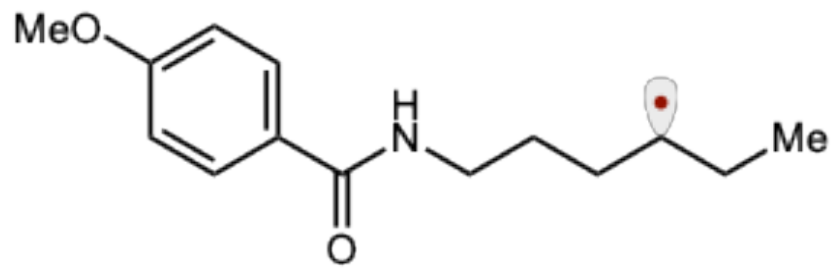

6-[(4-methoxyphenyl)formamido]hexan-3-yl
EE (radical): -750.93684 hartree
EE (N-1): -750.66986 hartree
EE (N+1): -750.92916 hartree
ω: 0.83 eV

N -0.7422755 -1.7374797 -0.5812794
C -2.0486829 -2.3623974 -0.3888651
C -4.6474316 2.0991293 -0.4479070
C -3.3997780 -0.1841588 -0.5852097
C -4.4564023 0.6894963 0.0035019
C -3.0921687 -1.4355442 0.2481768
C -5.6462319 2.8942525 0.4012158
H -2.3911614 -2.7078368 -1.3693567
H -4.9788186 2.1138511 -1.5024367
H -3.6751110 2.6144083 -0.4585771
H -3.6970199 -0.4969181 -1.6054400
H -2.4818012 0.4044729 -0.7246964
H -5.2166166 0.2349500 0.6341763
H -2.7415893 -1.1429852 1.2414969
H -4.0135917 -2.0120908 0.3946523
H -5.7500533 3.9198642 0.0369546
H -5.3230240 2.9379366 1.4451939
H -1.9046019 -3.2415513 0.2417020
H -6.6372795 2.4307437 0.3783268
C 0.1198337 -1.5436530 0.4641391
O -0.1305757 -1.9437019 1.5950846
C 1.3903666 -0.8146147 0.1456399
C 3.7876918 0.5783440 -0.3098802
C 1.9734942 -0.7876952 -1.1309359
C 2.0402422 -0.1493622 1.1862648
C 3.2235806 0.5519747 0.9708092
C 3.1575895 -0.1040177 -1.3589552
H 1.5238272 -1.3345142 -1.9525461
H 1.6033759 -0.1900754 2.1766151
H 3.6934001 1.0639414 1.7996491
H 3.6217561 -0.0927815 -2.3376328
O 4.9392858 1.2276594 -0.6328988
C 5.6325126 1.9386302 0.3862188
H 5.9589563 1.2684348 1.1885985
H 6.5046877 2.3737518 -0.0983800
H 5.0127755 2.7383473 0.8053628
H -0.5895571 -1.2135537 -1.4274716

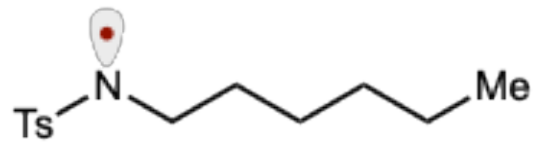

N-hexyl-4-methylbenzenesulfonamido

EE (radical): -1110.9706 hartree
EE (N-1): -1110.6324 hartree
EE (N+1): -1111.0599 hartree
ω: 2.50 eV

N 2.1614707 0.8327213 0.5682074
C 1.8880439 1.8719253 -0.3959251
H 1.3689880 1.4927615 -1.2850108
H 2.8669894 2.2216397 -0.7666709
C 1.1307574 3.0268163 0.2711028
H 1.0163537 3.8274199 -0.4690232
H 1.7490974 3.4239259 1.0814498
C -0.2437075 2.6201022 0.8240076
H -0.1083757 1.8045147 1.5428547
H -0.6553594 3.4640022 1.3892749
C -1.2580778 2.1958815 -0.2454119
H -1.3764253 3.0075499 -0.9756684
H -0.8793673 1.3334034 -0.8034861
C -2.6291910 1.8321219 0.3363316
H -2.5066417 1.0027590 1.0427299
H -3.0130758 2.6780501 0.9194811
C -3.6491483 1.4474669 -0.7397910
H -3.3104635 0.5750447 -1.3059442
H -3.7957948 2.2658447 -1.4517186
H -4.6243865 1.2083246 -0.3059089
S 2.3885029 -0.7364446 -0.0991013
O 2.7949671 -0.6790167 -1.5072484
O 3.1812748 -1.4764880 0.8742868
C 0.6763699 -1.2486627 -0.0221963
C -2.0325977 -1.8964329 0.1145503
C -0.0655521 -1.3359110 -1.1981003
C 0.0919086 -1.4876621 1.2246600
C -1.2578845 -1.8071369 1.2810581
C -1.4139997 -1.6709522 -1.1203410
H 0.4159981 -1.1545287 -2.1509734
H 0.6907300 -1.4236790 2.1245488
H -1.7219702 -1.9904132 2.2444161
H -1.9949954 -1.7517656 -2.0323960
C -3.5047337 -2.2057597 0.2011430
H -4.0542253 -1.3420700 0.5894018
H -3.6946317 -3.0449482 0.8751266
H -3.9218457 -2.4505137 -0.7771467

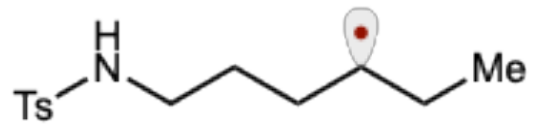

6-(4-methylbenzenesulfonyl)hexan-3-yl

EE (radical): -1110.9729 hartree
EE (N-1): -1110.6956 hartree
EE (N+1): -1110.976 hartree
ω: 0.98 eV

N -2.1899325 0.3858415 0.2683894
C -2.2316376 -0.5290424 -0.8852870
H -1.2260850 -0.7512372 -1.2657406
H -2.7856095 -0.0247376 -1.6773689
C -2.9218770 -1.8380205 -0.5024004
H -3.0610411 -2.4176242 -1.4205575
H -3.9192659 -1.6227931 -0.1043483
C -2.1281778 -2.7005308 0.5146700
H -2.0670057 -2.1800514 1.4792475
H -2.7222300 -3.6034141 0.7072477
C -0.7555605 -3.0753902 0.0554828
H -0.6649239 -3.7740048 -0.7725218
C 0.4745219 -2.4188902 0.5902475
H 0.4577705 -1.3412717 0.3675901
H 0.4651869 -2.4691577 1.6909304
C 1.7846815 -3.0095805 0.0600138
H 1.8421151 -2.9161897 -1.0287793
H 1.8691336 -4.0707577 0.3114374
H 2.6480914 -2.4899502 0.4824123
S -1.3527795 1.8327544 0.1357188
O -1.7543389 2.4197223 -1.1382623
O -1.5375265 2.5164451 1.4114787
C 0.3848922 1.3839414 0.0091479
C 3.0253314 0.4846471 -0.1543709
C 0.9311793 1.0828366 -1.2368619
C 1.1426104 1.2622919 1.1731958
C 2.4562268 0.8150886 1.0823669
C 2.2458169 0.6299951 -1.3076324
H 0.3363741 1.2125562 -2.1320138
H 0.7033314 1.5188892 2.1292070
H 3.0488258 0.7191125 1.9860987
H 2.6728747 0.3878943 -2.2751559
C 4.4570217 0.0200372 -0.2435619
H 4.7507412 -0.5446581 0.6442008
H 5.1346765 0.8765965 -0.3258445
H 4.6182710 -0.6115839 -1.1196907
H -1.9824824 -0.0562186 1.1576306

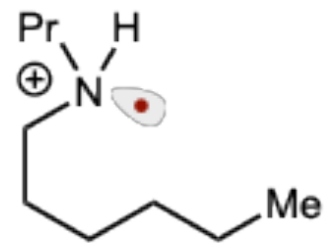

Hexyl(propyl)azaniumyl
EE (radical): -410.22586 hartree
EE (N-1): -409.72216 hartree
EE (N+1): -410.49312 hartree
ω: 8.55 eV

N -2.0811951 -1.2552905 -0.3369490
H -2.3057300 -1.5239524 -1.2946153
C -3.0495518 -0.4394304 0.3503684
H -2.6633698 -0.2116661 1.3448494
H -3.9622792 -1.0433043 0.4627454
C -3.4064342 0.8484276 -0.4402568
H -2.4942081 1.4314734 -0.5925720
H -3.7865331 0.5694739 -1.4282082
C -4.4531498 1.6707007 0.3173677
H -4.0885838 1.9788294 1.3002370
H -4.6866013 2.5749525 -0.2481737
H -5.3820980 1.1119731 0.4537473
C -0.7676239 -1.5489172 0.1383808
H -0.7867407 -1.6039781 1.2277336
H -0.4385417 -2.5000508 -0.2852392
C 0.2399585 -0.4168130 -0.3066723
H 0.2441485 -0.3582657 -1.3984459
H -0.1179312 0.5402133 0.0819072
C 1.6415281 -0.7327029 0.2302692
H 1.9657012 -1.7142093 -0.1321977
H 1.6121681 -0.7902938 1.3237732
C 2.6540654 0.3392126 -0.2048040
H 2.6899136 0.3873518 -1.2993262
H 2.3202367 1.3245850 0.1413814
C 4.0642178 0.0579347 0.3403841
H 4.0266856 0.0173635 1.4348398
H 4.3898985 -0.9329776 0.0041455
C 5.0784770 1.1162959 -0.1054064
H 6.0699855 0.8972818 0.2983115
H 4.7905989 2.1130519 0.2412360
H 5.1627227 1.1509329 -1.1952704

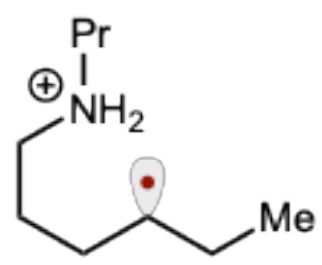

6-(propylazaniumyl)hexan-3-yl
EE (radical): -410.21781 hartree
EE (N-1): -409.83424 hartree
EE (N+1): -410.32642 hartree
ω: 3.00 eV

N -2.1925835 -1.4053633 -0.4169551
H -2.1426823 -1.1616163 -1.4105443
C -2.9599429 -0.3019743 0.3178166
H -2.4751280 -0.1805399 1.2869224
H -3.9678037 -0.6834409 0.4872836
C -2.9948842 0.9994075 -0.4761441
H -1.9761859 1.3332034 -0.6932250
H -3.4901375 0.8222384 -1.4385175
C -3.7466635 2.0913043 0.2947553
H -3.2482026 2.3268721 1.2385623
H -3.7903809 3.0074051 -0.2956607
H -4.7733896 1.7896658 0.5174958
C -0.7847075 -1.6998847 0.0848281
H -0.8923477 -1.9868770 1.1325802
H -0.4441285 -2.5729938 -0.4768054
C 0.1647779 -0.5234752 -0.0932083
H 0.1773720 -0.2079246 -1.1427688
H -0.1724090 0.3323590 0.4996698
C 1.5960459 -0.8960816 0.3351711
H 1.9517583 -1.7459363 -0.2614843
H 1.5709520 -1.2594539 1.3785295
C 2.5496874 0.2473786 0.2027672
H 2.1884706 1.2447942 0.4433757
C 4.0198503 0.0258639 0.0885026
H 4.4083571 -0.3669931 1.0453018
H 4.2152904 -0.7738798 -0.6394958
C 4.8075836 1.2855196 -0.2911816
H 5.8782921 1.0768532 -0.3409586
H 4.6580247 2.0803607 0.4451273
H 4.4939335 1.6673604 -1.2666097
H -2.7320522 -2.2722618 -0.3699336

Reaction Coordinate Geometries and Energies

Hydrogen Atom
ΔH = -313.62 kcal/mol

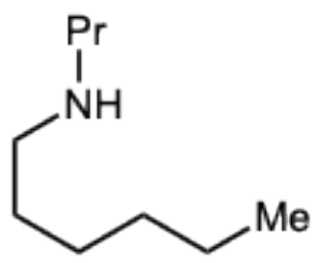

N-propylhexan-1-amine

ΔH = kcal/mol -257278.52

N 1.8715447 0.4380673 -0.1519725
H 1.8395030 1.0749120 0.6397093
C 3.1067769 -0.3325304 -0.0548018
H 3.1665764 -0.9933315 -0.9256541
H 3.1085599 -0.9859050 0.8329047
C 4.3276325 0.5684152 -0.0150234
H 4.3468250 1.1836446 -0.9188602
H 4.2347173 1.2561127 0.8321134
C 5.6229780 -0.2214712 0.1047219
H 5.7487381 -0.9011355 -0.7416167
H 6.4903570 0.4401067 0.1303074
H 5.6367525 -0.8221590 1.0173006
C 0.6878990 -0.4133648 -0.0884569
H 0.7008533 -1.0799515 -0.9567261
H 0.7034418 -1.0617735 0.8025745
C -0.5908324 0.4040396 -0.0915216
H -0.5765708 1.0940983 0.7601808
H -0.6198159 1.0208593 -0.9954456
C -1.8416814 -0.4616479 -0.0211418
H -1.8012532 -1.0879951 0.8770973
H -1.8552212 -1.1479275 -0.8754566
C -3.1301749 0.3501443 -0.0091164
H -3.1210252 1.0317384 0.8492738
H -3.1689698 0.9825913 -0.9035190
C -4.3836862 -0.5131527 0.0495379
H -4.3929172 -1.1924889 -0.8090180
H -4.3428349 -1.1459889 0.9420991
C -5.6654281 0.3079228 0.0644861
H -6.5506997 -0.3298295 0.1022791
H -5.7429067 0.9311653 -0.8299923
H -5.6965718 0.9712725 0.9325121

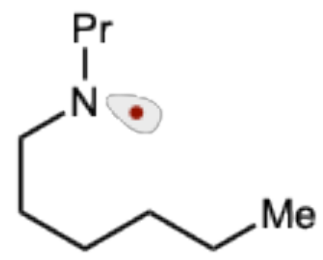

Propylhexylaminyl

ΔG = -256908.82 kcal/mol
ΔH = -256875.22 kcal/mol
N-H BDE = 89.7 kcal/mol

N 1.1397899 0.8137979 1.0254210
C 0.6040404 2.0875988 0.6180929
C -0.3704478 2.0786245 -0.5700812
C -1.6990191 1.3765460 -0.3013412
C -1.6283004 -0.1445773 -0.2303238
C -2.9938154 -0.7894855 -0.0284592
H 1.4485445 2.7411412 0.3526061
H 0.1003827 2.5411591 1.4752217
H 0.1118987 1.6486673 -1.4520066
H -0.5673974 3.1272251 -0.8080488
H -2.4004523 1.6521269 -1.0958765
H -2.1252336 1.7646030 0.6310886
H -0.9652051 -0.4515272 0.5847194
H -1.1806889 -0.5296516 -1.1543164
H -3.4448396 -0.3963899 0.8886032
H -3.6568757 -0.4953850 -0.8486423
C 1.9068672 0.1694505 -0.0024717
C 2.6646007 -1.0416617 0.5138933
H 2.6015143 0.8902794 -0.4618685
H 1.2371296 -0.1444741 -0.8165400
H 3.3372903 -0.7267389 1.3162879
C 3.4549956 -1.7309841 -0.5887259
H 1.9503994 -1.7420928 0.9559701
H 4.1824670 -1.0498177 -1.0369663
H 2.7947491 -2.0819805 -1.3856722
H 4.0012632 -2.5941722 -0.2054771
C -2.9189447 -2.3080313 0.0496889
H -2.2881015 -2.6268340 0.8833083
H -2.4930990 -2.7272137 -0.8655246
H -3.9055629 -2.7537937 0.1908563

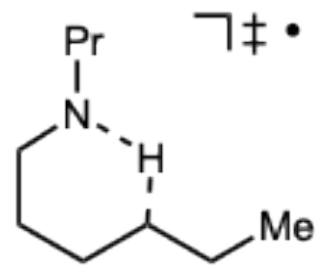

Neutral_amine_1-5-HAT_TS

ΔG = -256889.97 kcal/mol

N 0.4916706 0.1484504 0.8048684
C 0.3751402 1.6052454 0.7661537
C -0.4666408 2.1210186 -0.4070482
C -1.8427544 1.4605473 -0.3873995
C -1.7080066 -0.0409525 -0.4486931
C -2.8125572 -0.8762192 0.1423482
H 1.3775823 2.0449180 0.7237721
H -0.0847528 1.9424819 1.6995707
H 0.0354083 1.8999732 -1.3537165
H -0.5528360 3.2079815 -0.3370949
H -2.4493482 1.8294170 -1.2232430
H -2.3665545 1.7430313 0.5325303
H -0.6261373 -0.1901699 0.3140321
H -1.3616491 -0.4029334 -1.4205316
H -2.9979170 -0.5516982 1.1717354
H -3.7448460 -0.6835117 -0.4063660
C 1.4615889 -0.3538662 -0.1665053
C 2.8591590 -0.4383839 0.4364083
H 1.5023973 0.2580237 -1.0779252
H 1.1502170 -1.3573481 -0.4726570
H 3.1576367 0.5481469 0.8026926
C 3.8809729 -0.9431751 -0.5731000
H 2.8288749 -1.1021235 1.3046895
H 3.9562525 -0.2682372 -1.4290270
H 3.6045531 -1.9302655 -0.9521352
H 4.8732103 -1.0247663 -0.1260379
C -2.5057214 -2.3677309 0.1152640
H -1.6026419 -2.5900105 0.6891503
H -2.3431112 -2.7155451 -0.9078105
H -3.3236511 -2.9521560 0.5402041

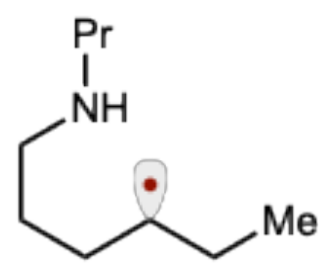

6-(propylamino)hexan-3-yl

ΔG = -256906.50 kcal/mol
ΔH = -256871.80 kcal/mol
C-H BDE = 93.1 kcal/mol

N 1.8216243 -0.4322960 -0.1366861
C 3.0684051 0.3195255 -0.0373542
H 3.0817116 0.9679974 0.8536231
H 3.1356650 0.9835441 -0.9051338
C 4.2758945 -0.5995388 -0.0058736
H 4.1741081 -1.2918675 0.8364668
H 4.2847523 -1.2083433 -0.9141112
C 5.5828450 0.1704347 0.1186321
H 5.6107420 0.7551399 1.0411296
H 6.4410204 -0.5034167 0.1271162
H 5.7124884 0.8624348 -0.7170466
C 0.6519627 0.4391573 -0.0926639
H 0.6650143 1.0915445 0.7953569
H 0.6875570 1.1006575 -0.9640981
C -0.6382712 -0.3589470 -0.1109899
H -0.6717452 -0.9687266 -1.0187607
H -0.6444369 -1.0526554 0.7368432
C -1.8758653 0.5282620 -0.0458497
H -1.8391674 1.2559623 -0.8738305
H -1.8433597 1.1430201 0.8647156
C -3.1558226 -0.2234547 -0.0951679
H -3.1480943 -1.2590816 -0.4209880
C -4.4583957 0.4710844 0.0684312
H -4.4267522 1.1048879 0.9646994
H -4.6092302 1.1764888 -0.7646270
C -5.6498794 -0.4753817 0.1443906
H -6.5863710 0.0731398 0.2574380
H -5.5552603 -1.1562718 0.9929870
H -5.7247070 -1.0811351 -0.7614293
H 1.7724850 -1.0588797 0.6622767

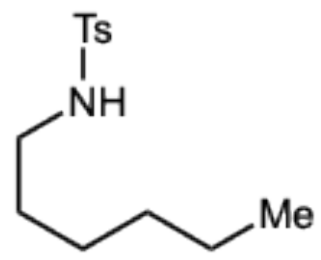

N-hexyl-4-methylbenzenesulfonamide

ΔH = -697215.34 kcal/mol

N -2.3107883 0.7323719 -0.3792811
H -3.1163409 0.9892465 -0.9409462
C -1.9325032 1.6773377 0.6811354
H -1.2277234 1.1709384 1.3426549
H -2.8060746 1.9380073 1.2818180
C -1.2970119 2.9236414 0.0857574
H -1.1504850 3.6409143 0.8988870
H -2.0081918 3.3800652 -0.6091541
C 0.0331500 2.6746540 -0.6211327
H -0.1098831 1.9568301 -1.4357975
H 0.3541983 3.6100661 -1.0900987
C 1.1415913 2.1815889 0.3030633
H 1.2157242 2.8539946 1.1665341
H 0.8900449 1.1927214 0.7022925
C 2.5002669 2.0990543 -0.3821906
H 2.4342029 1.4153795 -1.2351503
H 2.7585751 3.0804607 -0.7935120
C 3.6002585 1.6371899 0.5640968
H 3.3611910 0.6630420 0.9986893
H 3.7247391 2.3428724 1.3895696
H 4.5620825 1.5502228 0.0545523
S -2.2888215 -0.8539714 -0.0534345
O -2.7970687 -1.1448566 1.2664491
O -2.9266197 -1.4956917 -1.1784206
C -0.5726454 -1.2284027 -0.0504240
C 2.1505227 -1.7694813 -0.0754002
C 0.0589877 -1.5930993 1.1264587
C 0.1334086 -1.1297684 -1.2456216
C 1.4869148 -1.4022887 -1.2496244
C 1.4216166 -1.8604554 1.1054400
H -0.5037034 -1.6619409 2.0476936
H -0.3724172 -0.8410798 -2.1586810
H 2.0448866 -1.3268731 -2.1755964
H 1.9239097 -2.1381436 2.0243112
C 3.6202893 -2.0711623 -0.1048585
H 4.1559352 -1.3468853 -0.7199756
H 3.7962416 -3.0586676 -0.5389011
H 4.0479836 -2.0650835 0.8971479

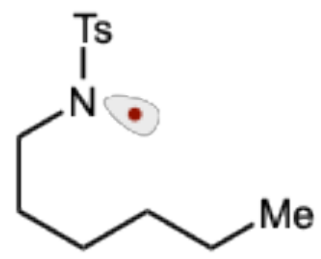

N-hexyl-4-methylbenzenesulfonamido

ΔG = -696842.70 kcal/mol
ΔH = -696802.60 kcal/mol
N-H BDE = 99.1 kcal/mol

N 1.5531311 -0.6208442 0.6620655
H 1.1396805 1.1628301 -0.9951659
C 2.9459335 -0.5831885 0.3066455
H 3.1291177 -0.9432508 -0.7106902
H 3.4393901 -1.2961847 0.9877560
C 3.5370623 0.8048438 0.5266737
H 4.6236526 0.6970154 0.5492350
H 3.2342265 1.1660528 1.5118596
C 3.1633287 1.8028515 -0.5691135
H 3.6826997 2.7445499 -0.3646535
H 3.5590965 1.4340997 -1.5207634
C 1.6711842 2.0847611 -0.7277437
H 1.5351262 2.7570083 -1.5815103
C 1.0123233 2.7081508 0.4971902
H 1.1469209 2.0566821 1.3643020
H 1.5179559 3.6504220 0.7337988
C -0.4752322 2.9563596 0.2871493
H -1.0015298 2.0178638 0.0911927
H -0.6495634 3.6172187 -0.5661836
H -0.9341222 3.4171937 1.1644189
S 0.6312731 -1.7600747 -0.1014436
O 1.0313001 -1.9417743 -1.4741788
O 0.6465842 -2.9086146 0.7736723
C -0.9463133 -1.0064130 -0.0687452
C -3.4336808 0.2172357 -0.0071967
C -1.5065385 -0.5480301 -1.2508284
C -1.6081086 -0.8699419 1.1479648
C -2.8456259 -0.2575648 1.1684365
C -2.7527720 0.0591917 -1.2103124
H -0.9783388 -0.6644997 -2.1876387
H -1.1613656 -1.2401890 2.0620460
H -3.3700094 -0.1454689 2.1098895
H -3.1998700 0.4177167 -2.1294195
C -4.7664609 0.9041025 0.0419079
H -5.1962295 1.0078683 -0.9536221
H -4.6612670 1.9034281 0.4713666
H -5.4670580 0.3531187 0.6704705

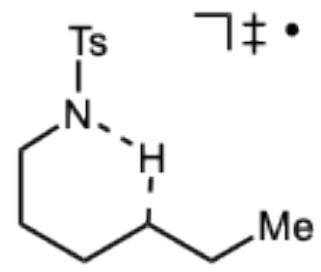

N-tosyl_1-5-HAT_TS

ΔG = -696832.84 kcal/mol

N 1.4886410 -0.5052785 0.7291319
H 1.3375196 0.6171084 -0.0335066
C 2.9439856 -0.7131209 0.6919102
H 3.2524359 -1.3189200 -0.1653112
H 3.2274007 -1.2419364 1.6049807
C 3.6416739 0.6410681 0.6381420
H 4.7196175 0.4746656 0.6458085
H 3.3918716 1.2157090 1.5336305
C 3.2239971 1.4081529 -0.6199297
H 3.7542339 2.3663451 -0.6538086
H 3.5241482 0.8372674 -1.5014039
C 1.7333037 1.6517284 -0.6383532
H 1.2646240 1.5762150 -1.6208175
C 1.2056725 2.7923912 0.1949778
H 1.6491473 2.7537519 1.1943130
H 1.5560450 3.7280818 -0.2587963
C -0.3137948 2.8053654 0.2948751
H -0.6830382 1.8927596 0.7701008
H -0.7706354 2.8772330 -0.6949781
H -0.6616421 3.6542167 0.8859078
S 0.6186561 -1.6273252 -0.0819213
O 1.0337251 -1.7489017 -1.4629906
O 0.6355510 -2.8400589 0.7072439
C -0.9815876 -0.9136488 -0.0399858
C -3.4843800 0.2818110 0.0508048
C -1.5194947 -0.3747526 -1.1966378
C -1.6747691 -0.8730644 1.1658013
C -2.9186745 -0.2741551 1.2021610
C -2.7728001 0.2192246 -1.1423412
H -0.9675715 -0.4159824 -2.1262403
H -1.2432541 -1.3017601 2.0614617
H -3.4652992 -0.2345573 2.1370296
H -3.1996997 0.6436570 -2.0429555
C -4.8371149 0.9284829 0.1146279
H -5.1148204 1.3617448 -0.8453874
H -4.8520395 1.7185868 0.8675264
H -5.5997678 0.1995201 0.3958969

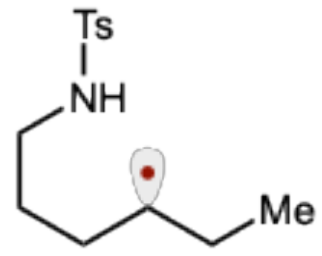

6-(4-methylbenzenesulfonyl)hexan-3-yl

ΔG = -696848.99 kcal/mol
ΔH = -696808.85 kcal/mol
C-H BDE = 92.9 kcal/mol

N -2.1858982 0.4224381 0.2582235
C -2.2067982 -0.5264402 -0.8694794
H -1.1925996 -0.7463096 -1.2214308
H -2.7521672 -0.0546457 -1.6848820
C -2.8918282 -1.8181261 -0.4576517
H -3.0395284 -2.4108385 -1.3633662
H -3.8830497 -1.5932309 -0.0546361
C -2.0898457 -2.6543130 0.5561828
H -1.9821911 -2.1065424 1.4986221
H -2.6932789 -3.5388270 0.7910427
C -0.7477055 -3.0582683 0.0549628
H -0.6932019 -3.7185602 -0.8052647
C 0.5038747 -2.4653284 0.5939844
H 0.4761043 -1.3719130 0.4662693
H 0.5425293 -2.6121118 1.6833361
C 1.7706048 -3.0167585 -0.0465821
H 1.7888782 -2.8081044 -1.1192134
H 1.8336736 -4.0997858 0.0835412
H 2.6655606 -2.5749609 0.3955456
S -1.3402699 1.8005436 0.0850896
O -1.7238351 2.3682938 -1.1813517
O -1.5388381 2.5544737 1.2954210
C 0.3608114 1.3626457 -0.0077672
C 2.9979056 0.4913094 -0.1228309
C 0.9227458 1.0433474 -1.2374276
C 1.0989320 1.2574184 1.1647749
C 2.4128120 0.8266911 1.0976640
C 2.2369680 0.6066456 -1.2844484
H 0.3428036 1.1380771 -2.1461455
H 0.6500838 1.5079595 2.1173989
H 2.9933856 0.7434148 2.0087503
H 2.6791115 0.3504744 -2.2400101
C 4.4258011 0.0336437 -0.1868495
H 4.7248800 -0.4575364 0.7390388
H 5.0918374 0.8869513 -0.3377079
H 4.5814435 -0.6554689 -1.0170160
H -1.9905106 -0.0027124 1.1605293

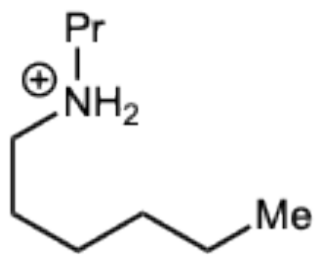

N-propylhexan-1-aminium

ΔH = -257560.96 kcal/mol

N -1.8518152 0.3947974 -0.0009641
H -1.8305539 1.0074143 -0.8202924
C -3.1298856 -0.3757903 -0.0015690
H -3.1124225 -1.0127865 0.8818627
H -3.1152757 -1.0065357 -0.8895434
C -4.3232302 0.5563272 0.0034387
H -4.2760626 1.1974767 0.8877378
H -4.2749079 1.2081491 -0.8729813
C -5.6261318 -0.2299307 -0.0023934
H -5.6998639 -0.8751016 0.8753006
H -6.4828702 0.4439269 0.0039591
H -5.7010212 -0.8600655 -0.8908329
C -0.6266571 -0.4579304 0.0027026
H -0.6862960 -1.0872162 0.8897674
H -0.6840918 -1.0918926 -0.8811925
C 0.6259571 0.3922066 0.0021273
H 0.6222752 1.0430888 -0.8777075
H 0.6256630 1.0389369 0.8849831
C 1.8788167 -0.4743500 -0.0022002
H 1.8634451 -1.1279757 -0.8806719
H 1.8681195 -1.1302368 0.8746582
C 3.1607026 0.3471244 -0.0044177
H 3.1705304 1.0018349 -0.8829991
H 3.1714326 1.0050742 0.8717521
C 4.4175376 -0.5131616 -0.0032601
H 4.4032651 -1.1690838 0.8730770
H 4.4048807 -1.1693141 -0.8794595
C 5.6958862 0.3132052 -0.0023002
H 6.5834216 -0.3221966 0.0021898
H 5.7449442 0.9592147 0.8777656
H 5.7499805 0.9537845 -0.8860248
H -1.8332008 1.0111985 0.8156250

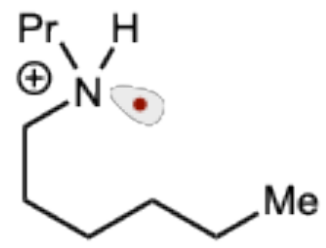

Hexyl(propyl)azaniumyl

ΔG = -257186.07 kcal/mol
ΔH = -257151.49 kcal/mol
N-H BDE = 95.9 kcal/mol

N -2.0761992 -1.2476943 -0.2964080
H -2.3968898 -1.6000033 -1.1979044
C -2.9353743 -0.3425587 0.3985339
H -2.4082902 0.0329522 1.2740120
H -3.8022975 -0.9274529 0.7369454
C -3.4244092 0.7810345 -0.5202521
H -2.5605388 1.3438484 -0.8777116
H -3.9150746 0.3391148 -1.3901013
C -4.3847097 1.6929455 0.2270448
H -3.9006443 2.1505312 1.0917159
H -4.7319218 2.4934982 -0.4262357
H -5.2575011 1.1402143 0.5792419
C -0.7529096 -1.5693350 0.1279866
H -0.7631786 -1.6832391 1.2136597
H -0.4480612 -2.4949954 -0.3554244
C 0.2118048 -0.4223536 -0.2532116
H 0.1745623 -0.2701388 -1.3337184
H -0.1269398 0.4964878 0.2300678
C 1.6280481 -0.7659284 0.1882427
H 1.9454384 -1.6926044 -0.2996810
H 1.6361680 -0.9563525 1.2661399
C 2.6127625 0.3484317 -0.1414172
H 2.5939742 0.5435622 -1.2193572
H 2.2896727 1.2738088 0.3477944
C 4.0382418 0.0225927 0.2866093
H 4.0527694 -0.1828124 1.3617722
H 4.3603963 -0.8989587 -0.2083813
C 5.0165584 1.1441042 -0.0335884
H 6.0314921 0.8904748 0.2780374
H 4.7332703 2.0684533 0.4756955
H 5.0395153 1.3505749 -1.1065666

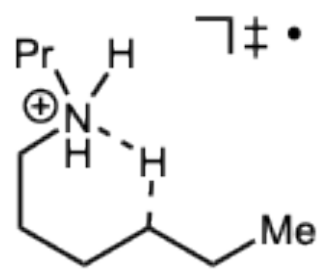

Cationic_amminium_1-5-HAT_TS

ΔG = -257174.83 kcal/mol

N -0.0144887 -0.0139678 -0.0063794
H 0.0098289 -0.0020518 1.0120141
H 1.3782079 0.0137756 -0.3188729
C 2.3569901 -0.3250942 -0.9945458
H 2.3529989 0.4686452 -1.7469433
C -0.5692751 1.2170236 -0.5794313
H -0.5928535 1.0995172 -1.6627719
H 0.1166136 2.0297051 -0.3367486
C -0.4138435 -1.3016477 -0.5809974
H -0.2505891 -2.0644451 0.1784823
H -1.4812153 -1.2568769 -0.8096292
C 3.5603107 -0.2987461 -0.0925127
H 3.4226181 -1.0203603 0.7171982
H 4.4166050 -0.6567018 -0.6785183
C 1.8836588 -1.6517163 -1.5267811
H 2.0920594 -2.4415279 -0.7998308
H 2.4391900 -1.8920302 -2.4396659
C 3.8577615 1.0844421 0.4672172
H 4.7476044 1.0704703 1.0976772
H 4.0266305 1.8042918 -0.3363945
H 3.0245938 1.4479649 1.0735822
C -1.9569378 1.5155506 -0.0266189
H -2.6283199 0.6840265 -0.2514495
H -1.8945560 1.5995706 1.0616734
C -2.5033085 2.8047269 -0.6226011
H -3.4933311 3.0252665 -0.2223900
H -1.8524729 3.6508910 -0.3932523
H -2.5891326 2.7299807 -1.7086969
C 0.3897950 -1.6171794 -1.8398795
H 0.0491887 -2.5805560 -2.2191544
H 0.1815388 -0.8735345 -2.6130475

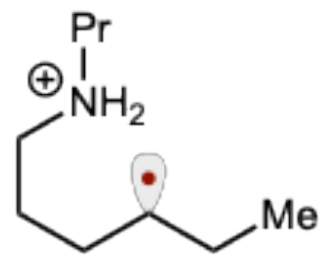

6-(propylazaniumyl)hexan-3-yl

ΔG = -257185.70 kcal/mol
ΔH = -257151.27 kcal/mol
C-H BDE = 96.1 kcal/mol

N -2.2046033 -1.4145061 -0.3571144
H -2.2127264 -1.2199752 -1.3615019
C -2.8985086 -0.2943753 0.3711499
H -2.3527641 -0.1288066 1.2988455
H -3.8959887 -0.6513046 0.6205421
C -2.9782789 0.9638158 -0.4710759
H -1.9763146 1.2774741 -0.7706341
H -3.5352363 0.7411896 -1.3854237
C -3.6647596 2.0810906 0.3005504
H -3.1028478 2.3384219 1.2007194
H -3.7457667 2.9790765 -0.3121243
H -4.6724286 1.7891842 0.6035807
C -0.7964227 -1.6898431 0.0717275
H -0.8422516 -1.9545663 1.1274074
H -0.4719777 -2.5636150 -0.4922326
C 0.1258558 -0.5141614 -0.1698944
H 0.0680184 -0.2103099 -1.2191062
H -0.1840326 0.3396896 0.4371904
C 1.5641431 -0.8827789 0.1815852
H 1.8947523 -1.7215889 -0.4444484
H 1.5909967 -1.2703062 1.2136327
C 2.5105908 0.2533363 0.0424782
H 2.1219014 1.2666122 0.0648405
C 3.9770628 0.0370066 0.1306661
H 4.2348302 -0.3450654 1.1315496
H 4.2708746 -0.7675578 -0.5563330
C 4.7965268 1.2890415 -0.1546477
H 5.8661932 1.0888407 -0.0746934
H 4.5516571 2.0847301 0.5522428
H 4.5994352 1.6647800 -1.1609340
H -2.7411640 -2.2736390 -0.2333478

XIII. References

36) Garwood, J. J.; A., Chen, A. D.; Nagib, D. A. Radical Polarity. *J. Am. Chem. Soc*., **2024**, *146,* 28034-28059.

55) Berman, A. M.; Johnson, J. S. Copper-Catalyzed Electrophilic Amination of Organozinc Nucleophiles: Documentation of O-Benzoyl Hydroxylamines as Broadly Useful $R_2N(+)$ and RHN(+) Synthons. *J. Org. Chem.*, **2006**, *71*, 219-224.

63) Huang, H.-T.; Lacy, T. C.; Błachut, B.; Ortiz, G. X. J.; Wang, Q. An Efficient Synthesis of Fluorinated Azaheterocycles by Aminocyclization of Alkenes. *Org. Lett*., **2013**, *15*, 1818–1821.

68) Spartan'18 Wavefunction Inc., Irvine, CA

69) Halgren, T. A. Merck molecular force field. I. Basis, form, scope, parameterization, and performance of MMFF94. *J. Comput. Chem.* **1996**, *17*, 490–519.

70) Mardirossian, N.; Head-Gordon, M. ω B97M-V: A Combinatorially Optimized, Range-Separated Hybrid, Meta-GGA Density Functional with VV10 Nonlocal Correlation. *J. Chem. Phys.* **2016**, *144*, 214110.

71) Epifanovsky, E. *et al.* Software for the Frontiers of Quantum Chemistry: An Overview of Developments in the Q-Chem 5 Package. *J. Chem. Phys.* **2021**, *155*, 084801.

72) Stephen, P. J.; Devlin, F. J.; Chabalowski, C. F.; Frisch, M. J. Ab Initio Calculation of Vibrational Absorption. *J. Phys. Chem.* **1994**, *98*, 11623–11627.

73) Grimme, S.; Antony, J.; Ehrlich, S.; Krieg, H. A Consistent and Accurate Ab Initio Parametrization of Density Functional Dispersion Correction (DFT-D) for the 94 Elements H-Pu. *J. Chem. Phys.* **2010**, *132*. 10024-10035.

74) Zhang, G.; Musgrave, C. B. Comparison of DFT Methods for Molecular Orbital Eigenvalue Calculations. *J. Phys. Chem. A* **2007**, *111*, 1554–1561.

O-benzoyl-N,N-dihexylhydroxylamine (S1)

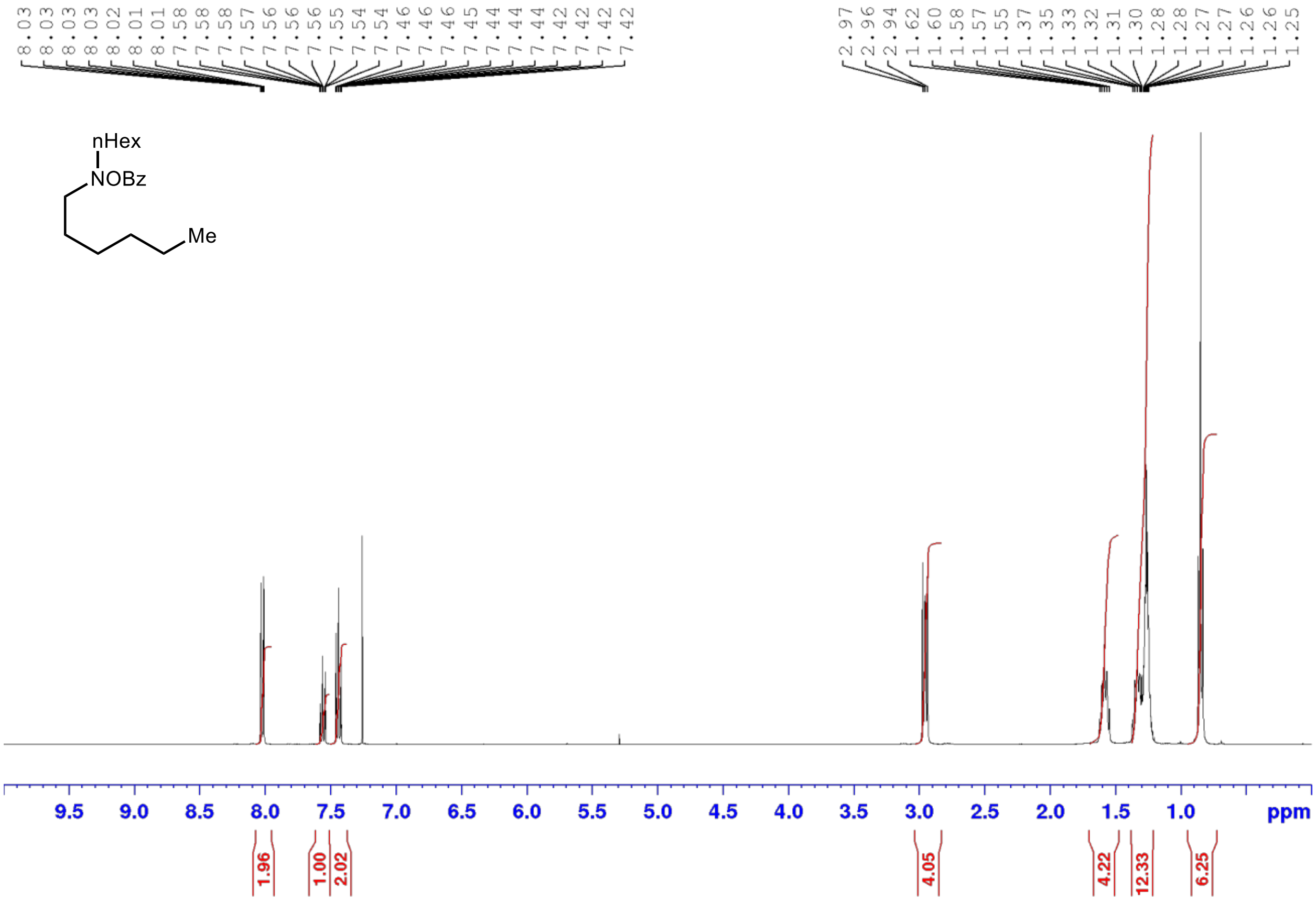

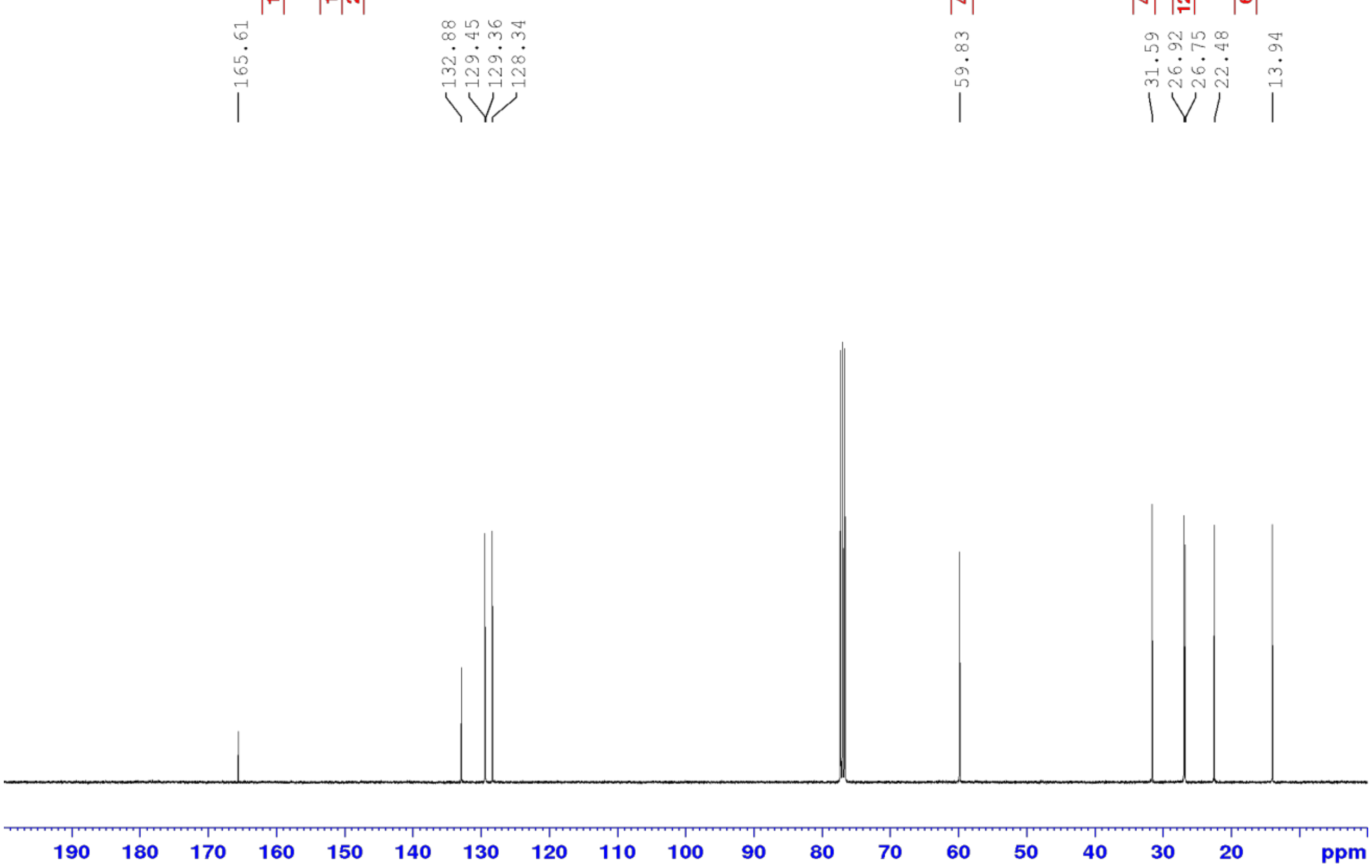

O-benzoyl-N-hexyl-N-propylhydroxylamine (S2)

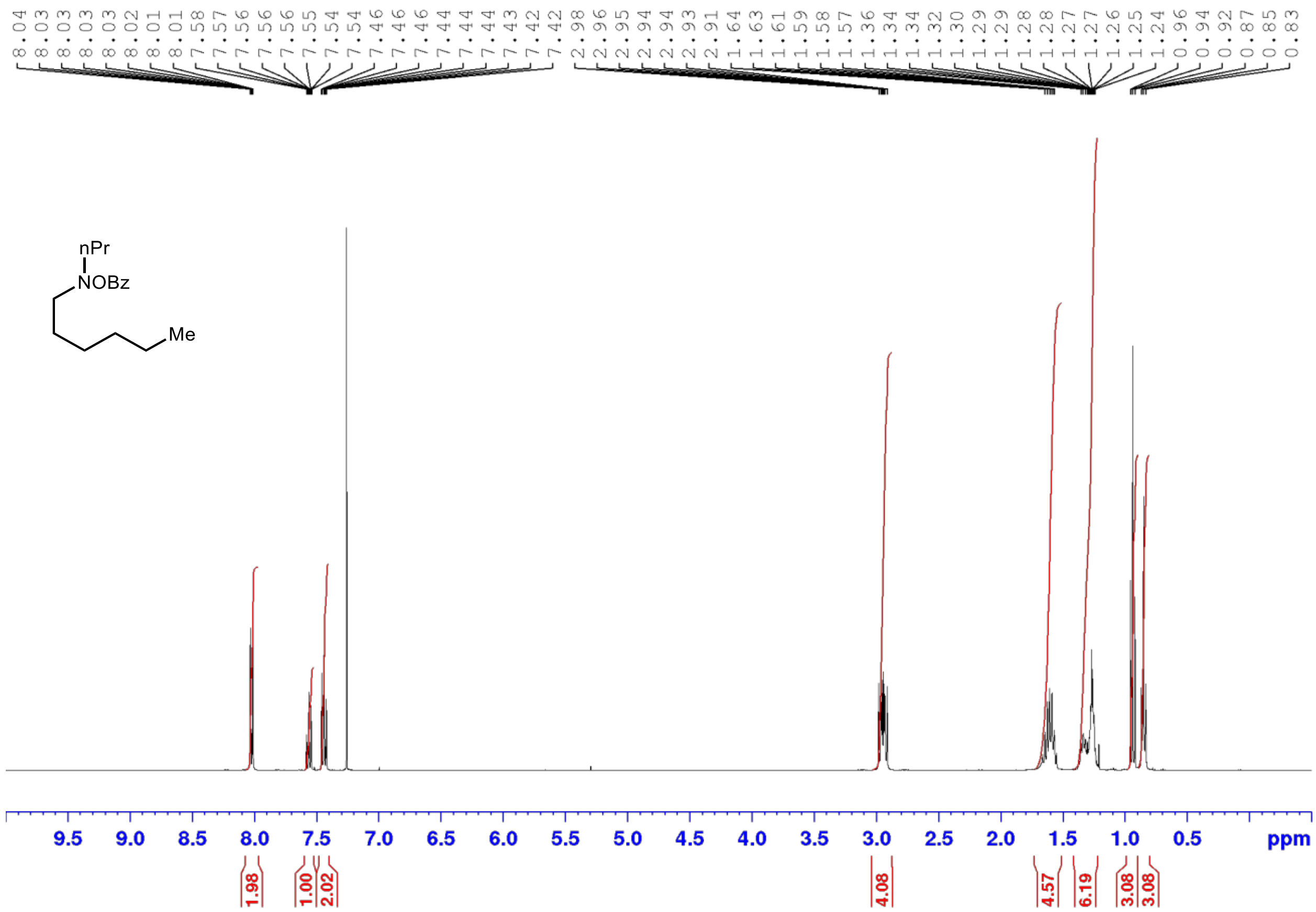

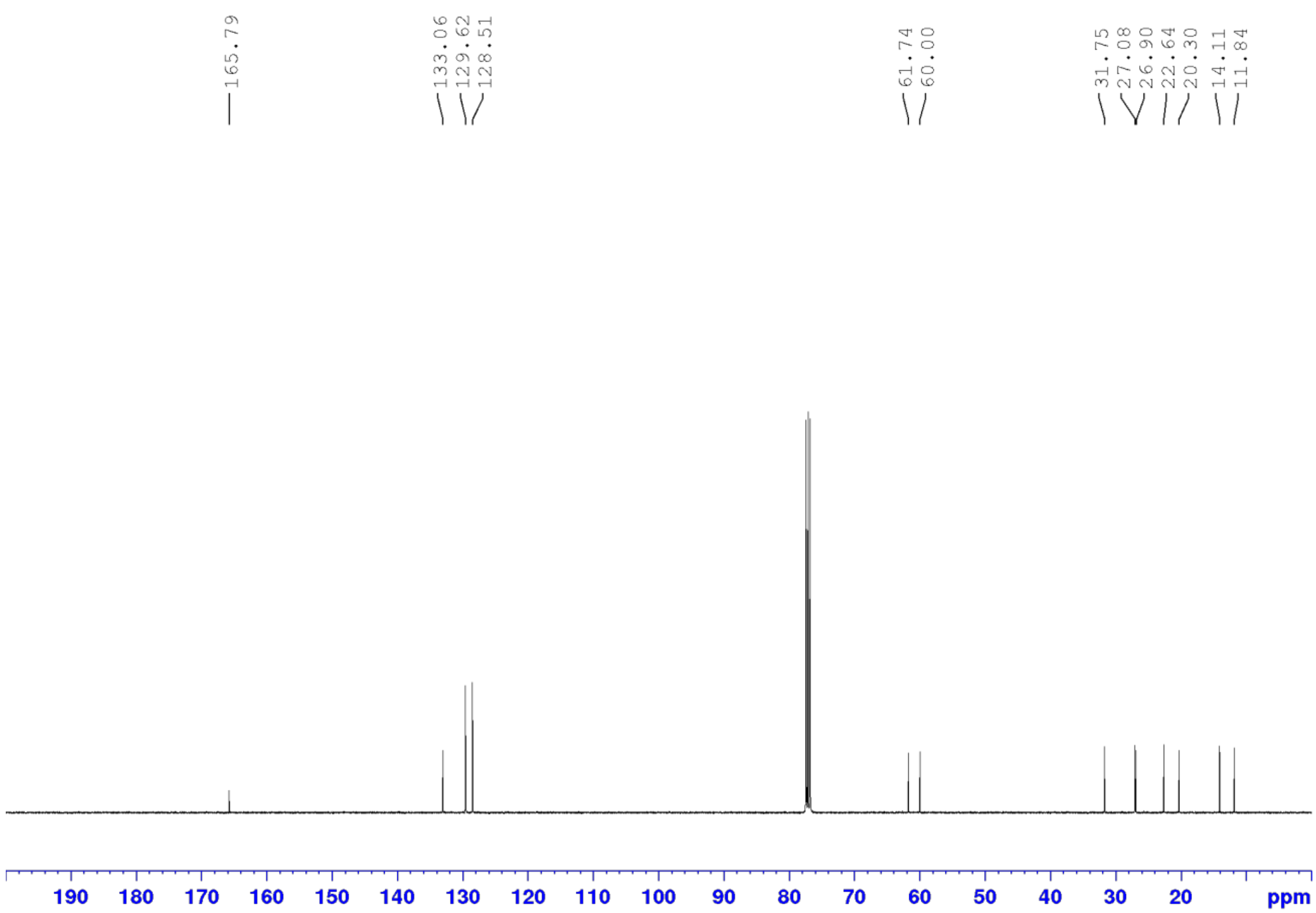

O-benzoyl-N-(tert-butyl)-N-hexylhydroxylamine (S3)

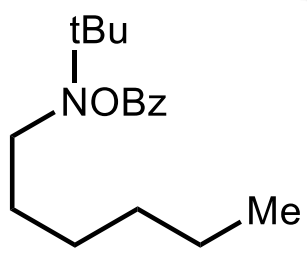

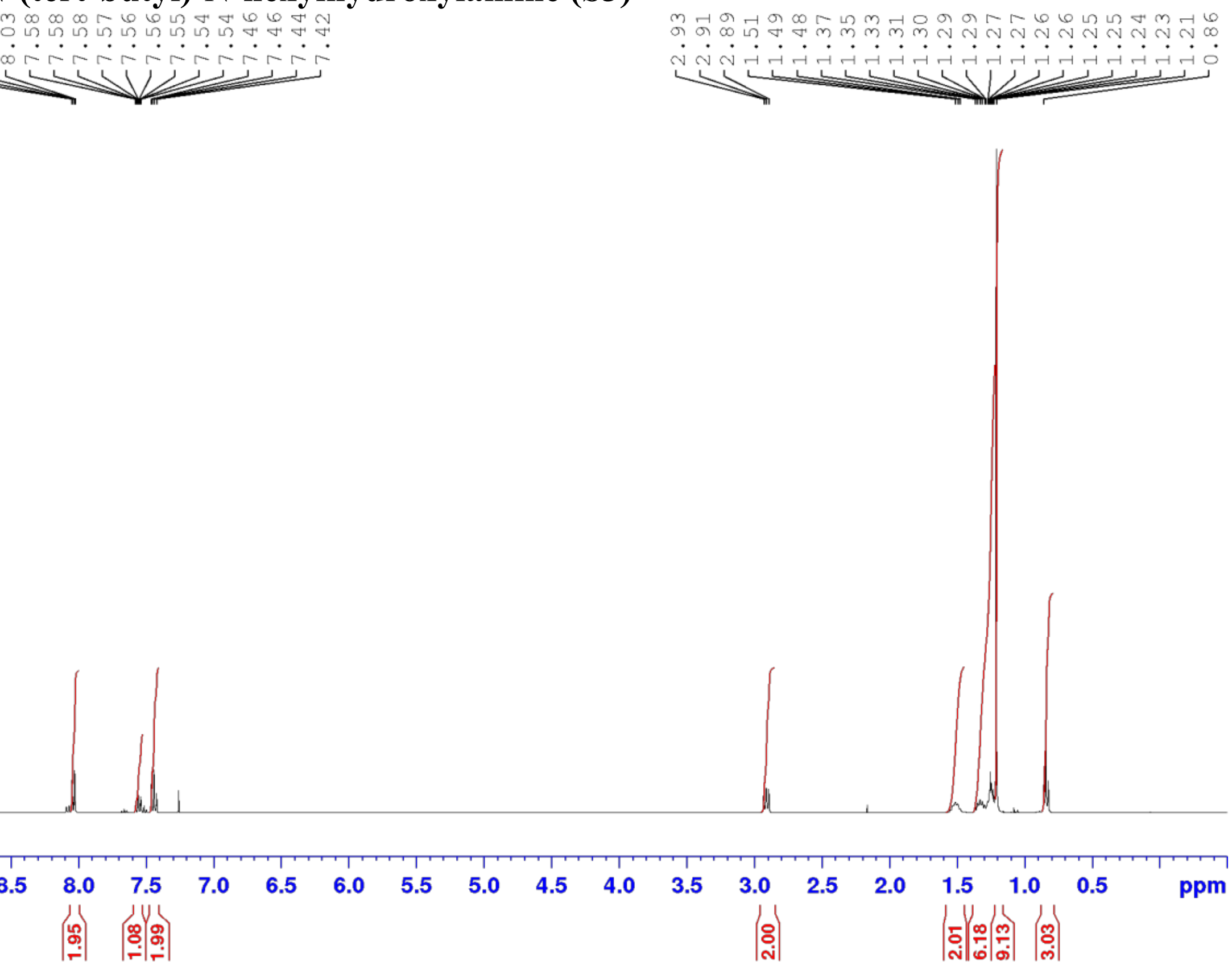

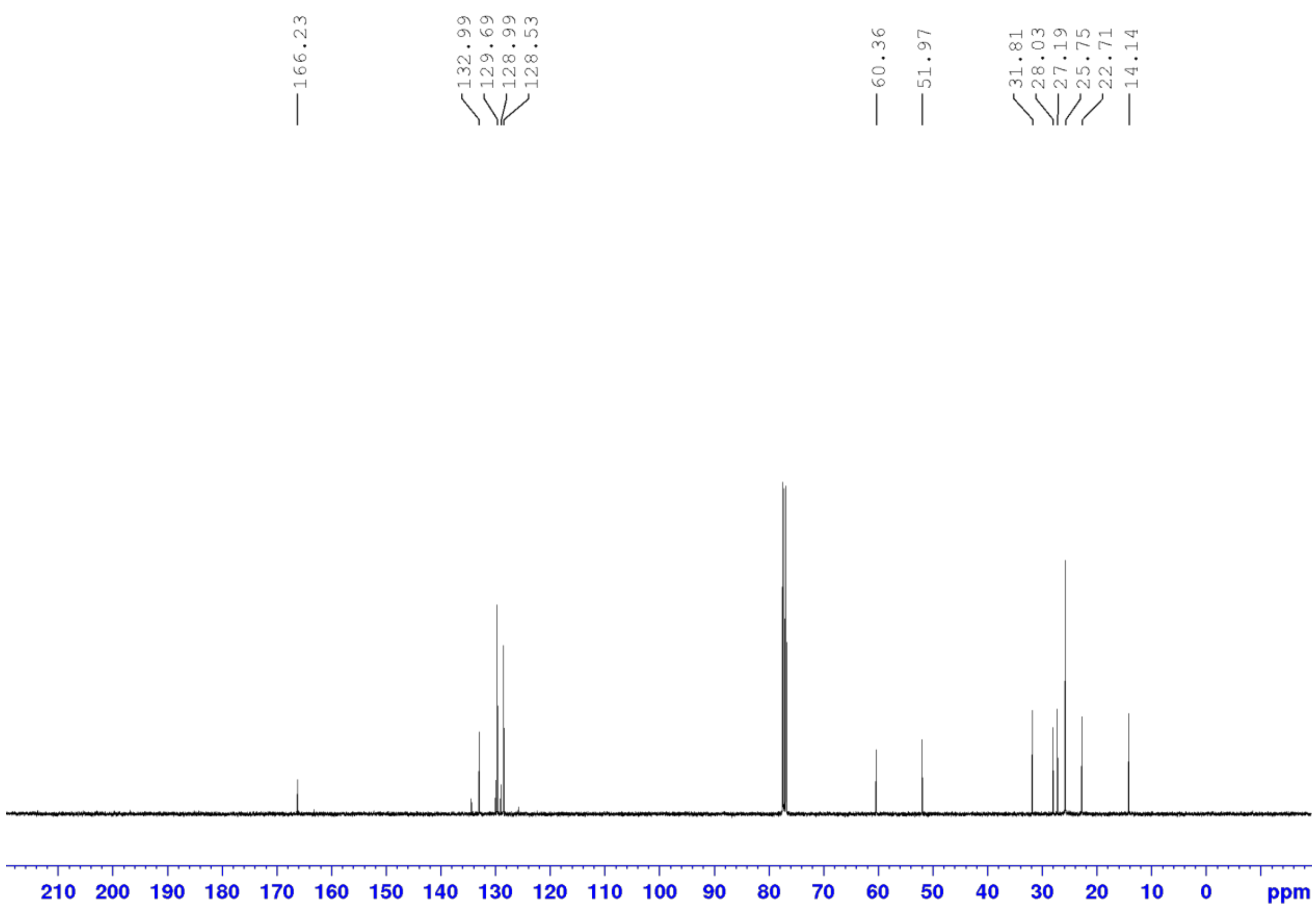

O-benzoyl-N-(heptan-2-yl)-N-propylhydroxylamine (S4)

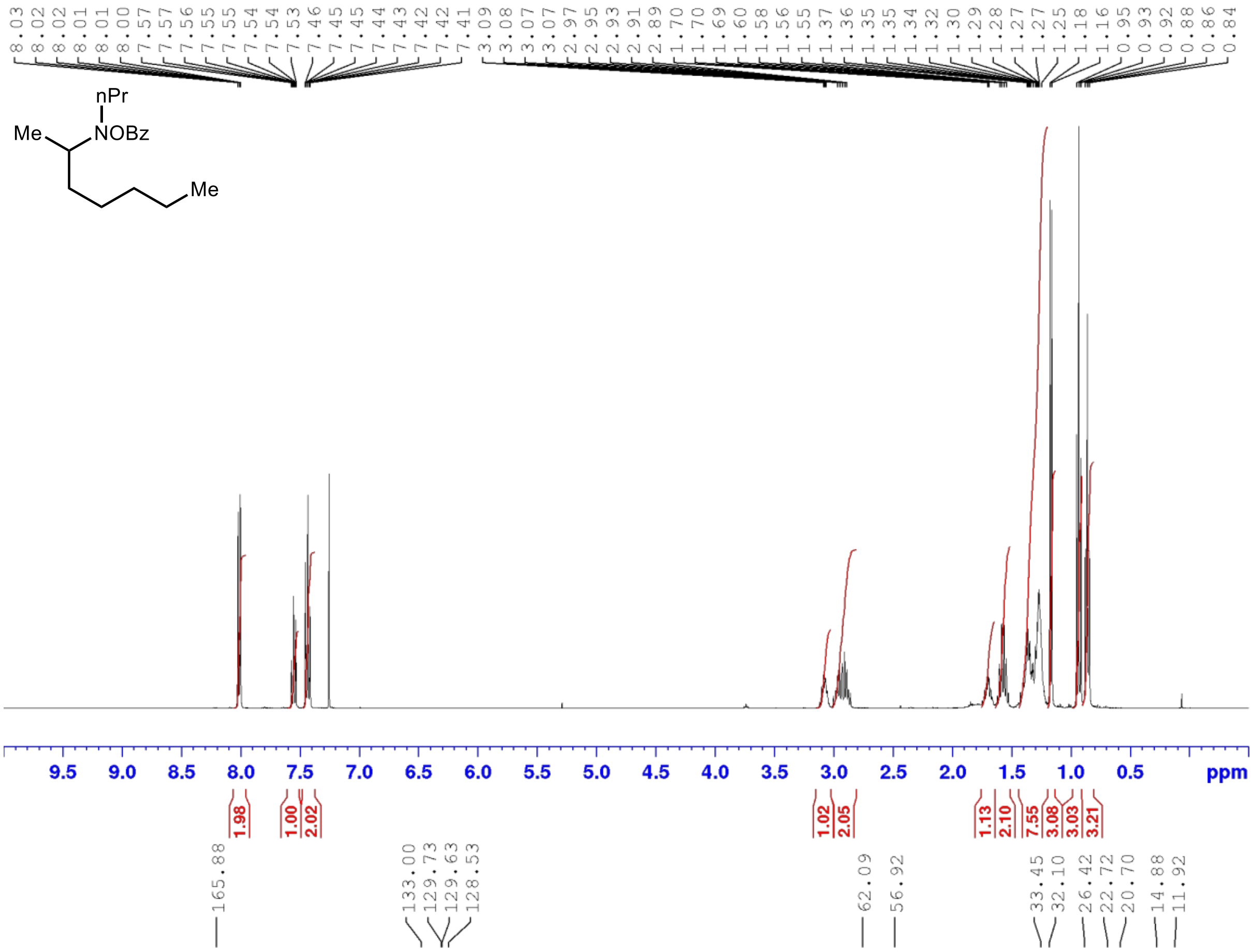

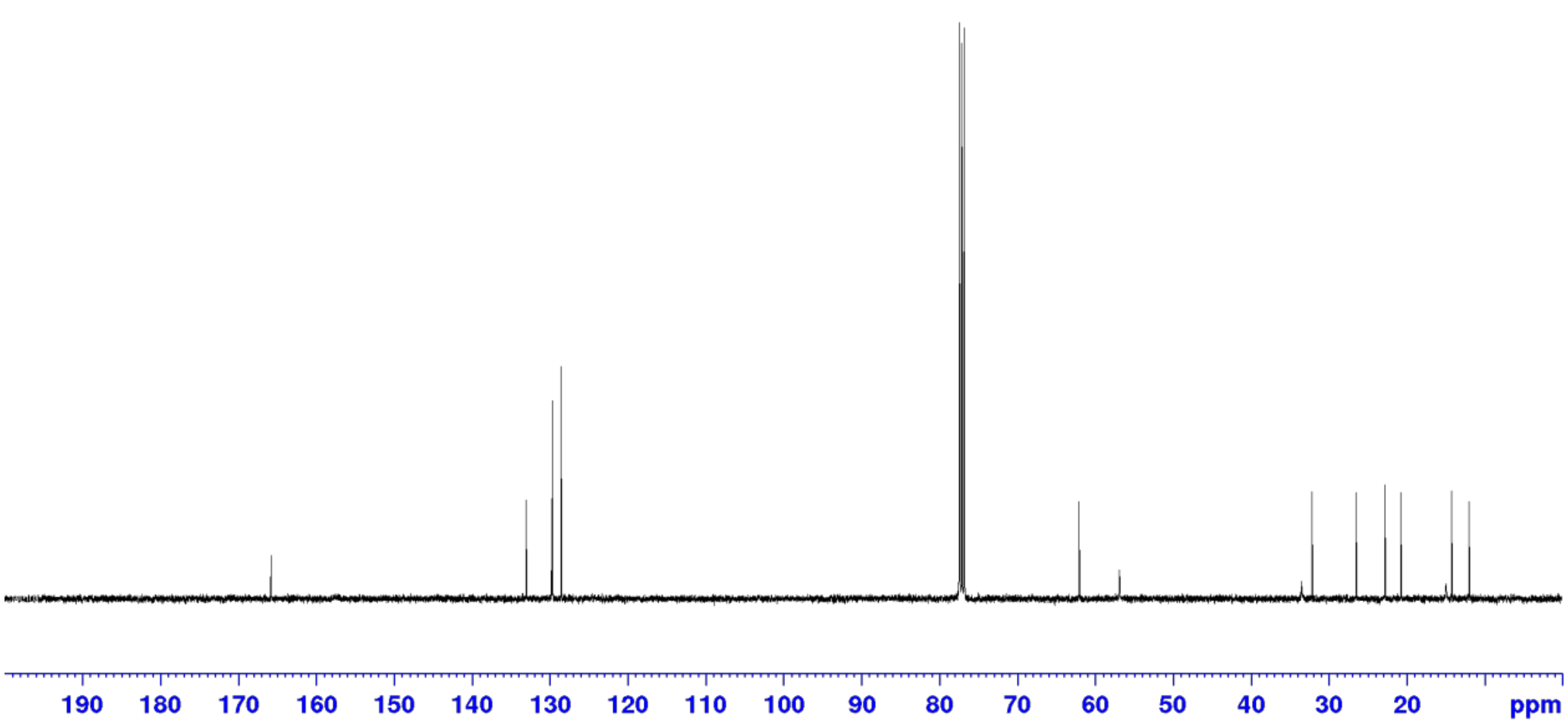

O-benzoyl-N-(2-methylhexyl)-N-propylhydroxylamine (S5)

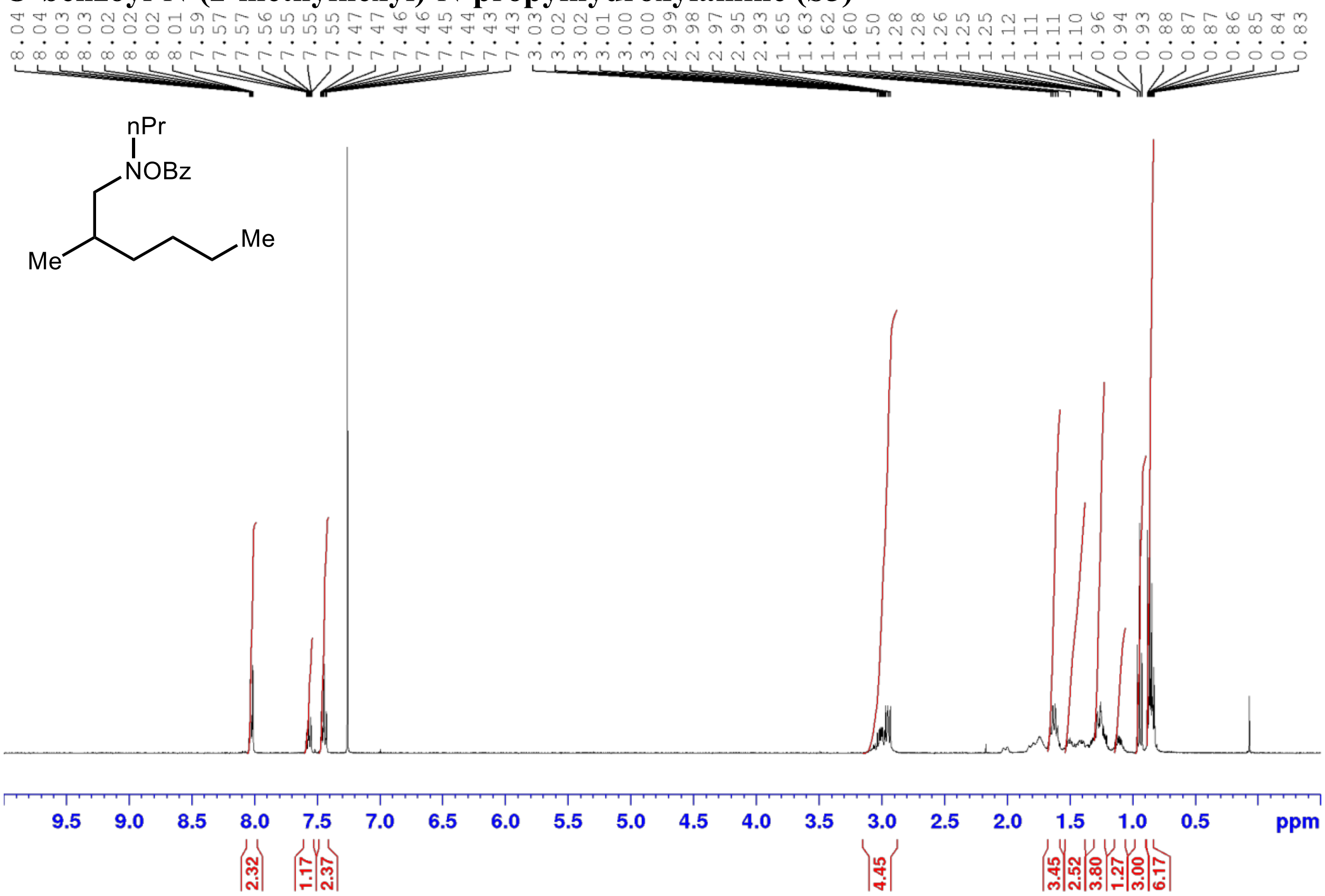

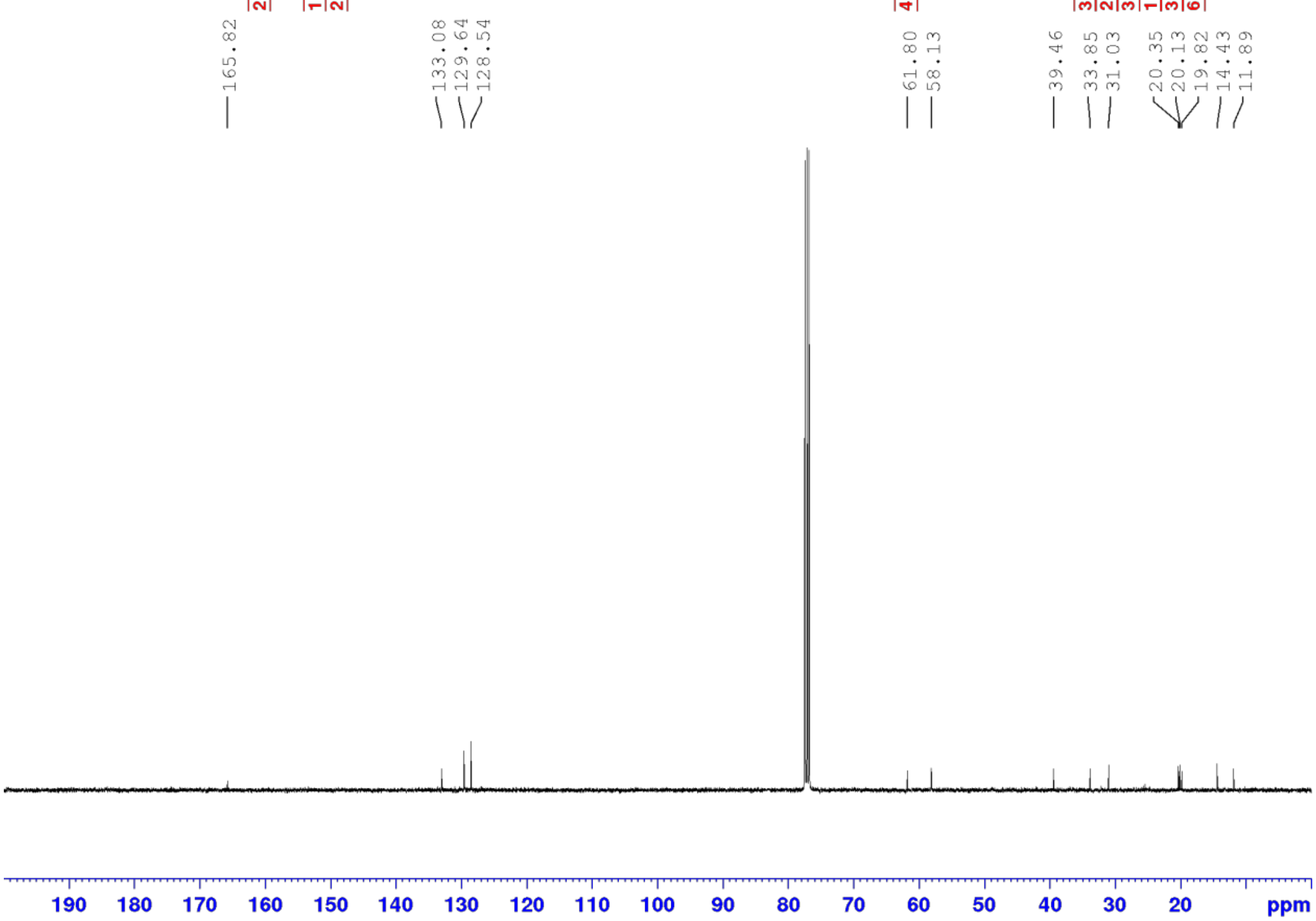

O-benzoyl-N-(3-methylhexyl)-N-propylhydroxylamine (S6)

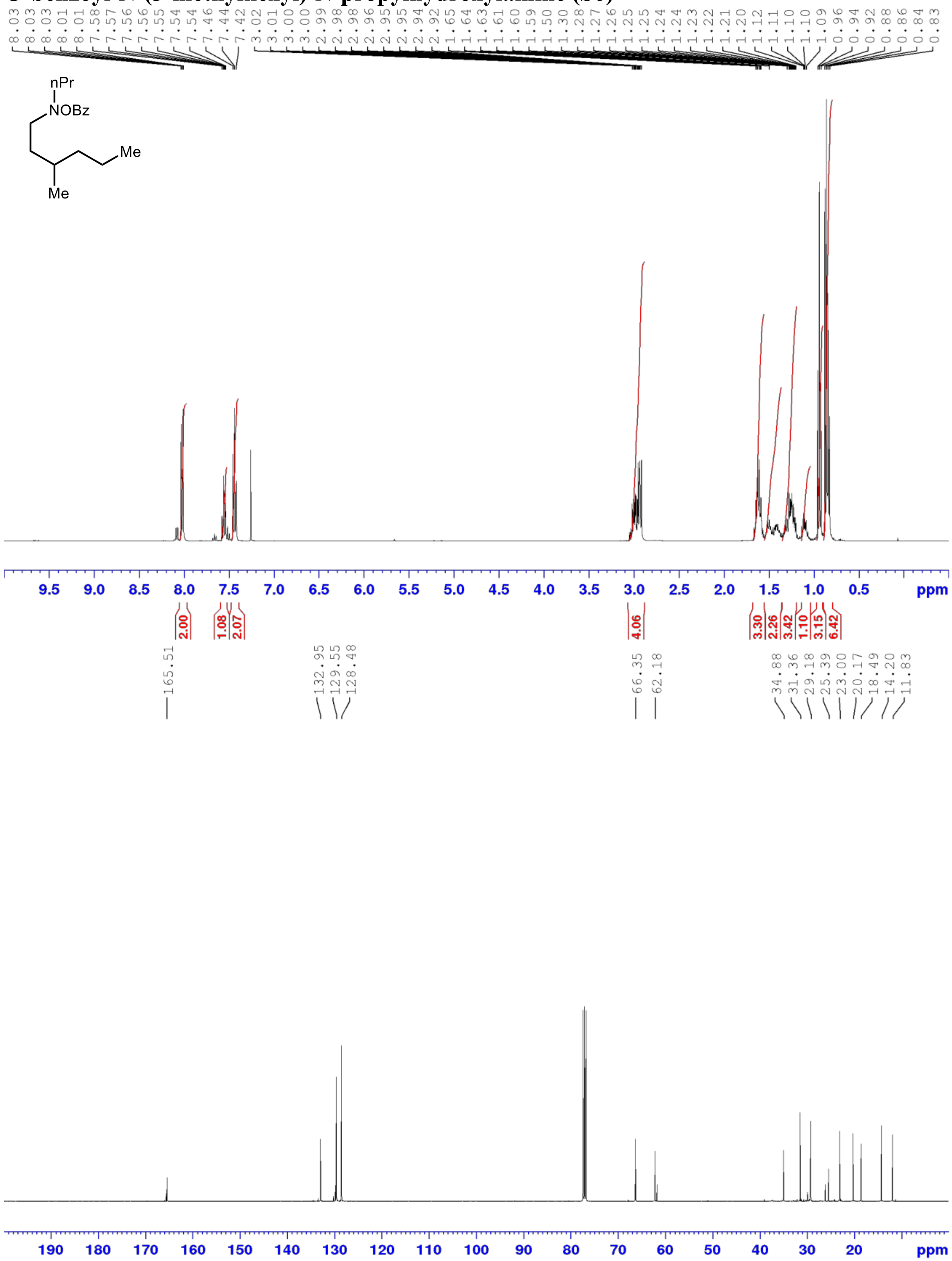

O-benzoyl-N-(2-cyclopentylethyl)-N-propylhydroxylamine (S7)

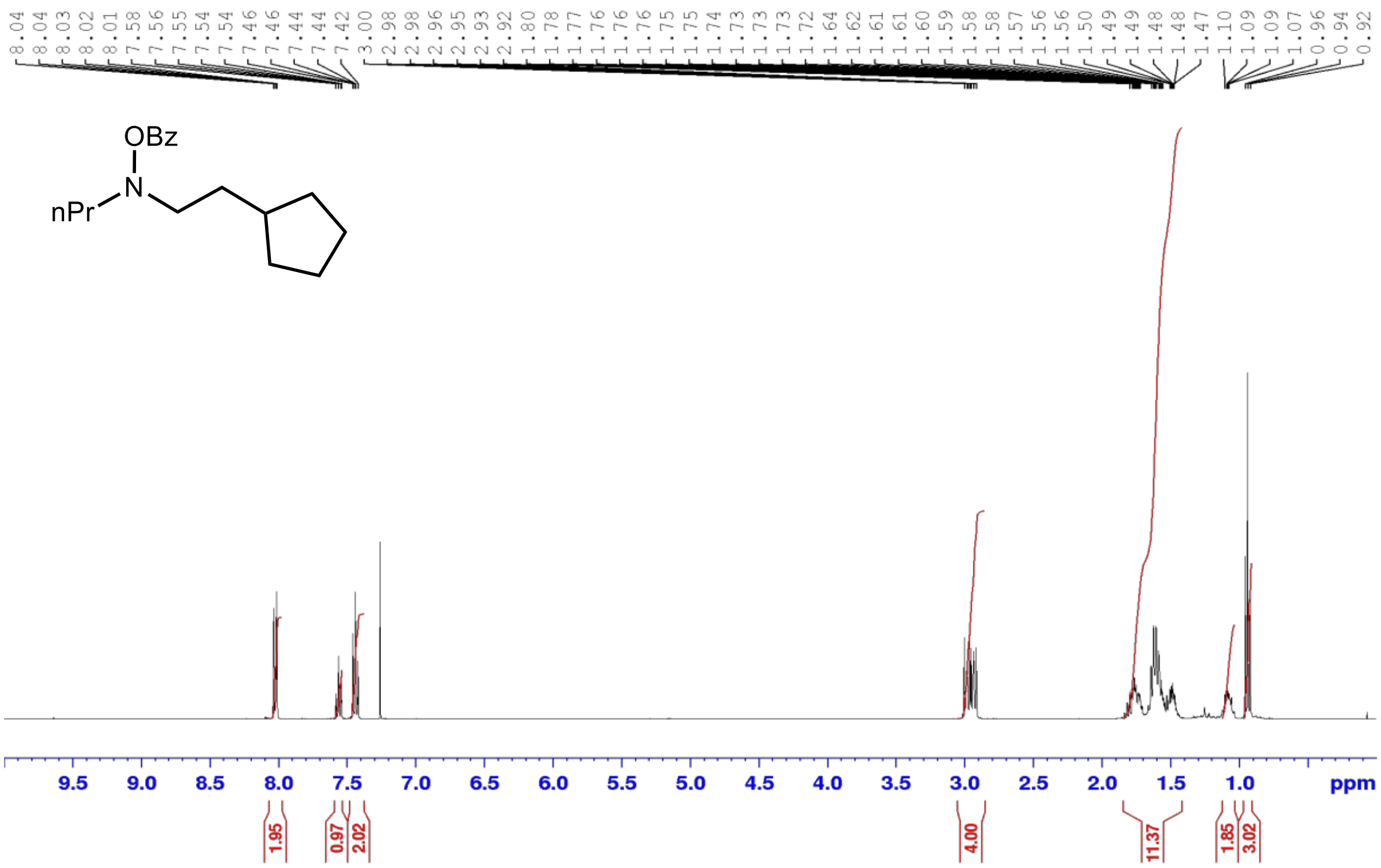

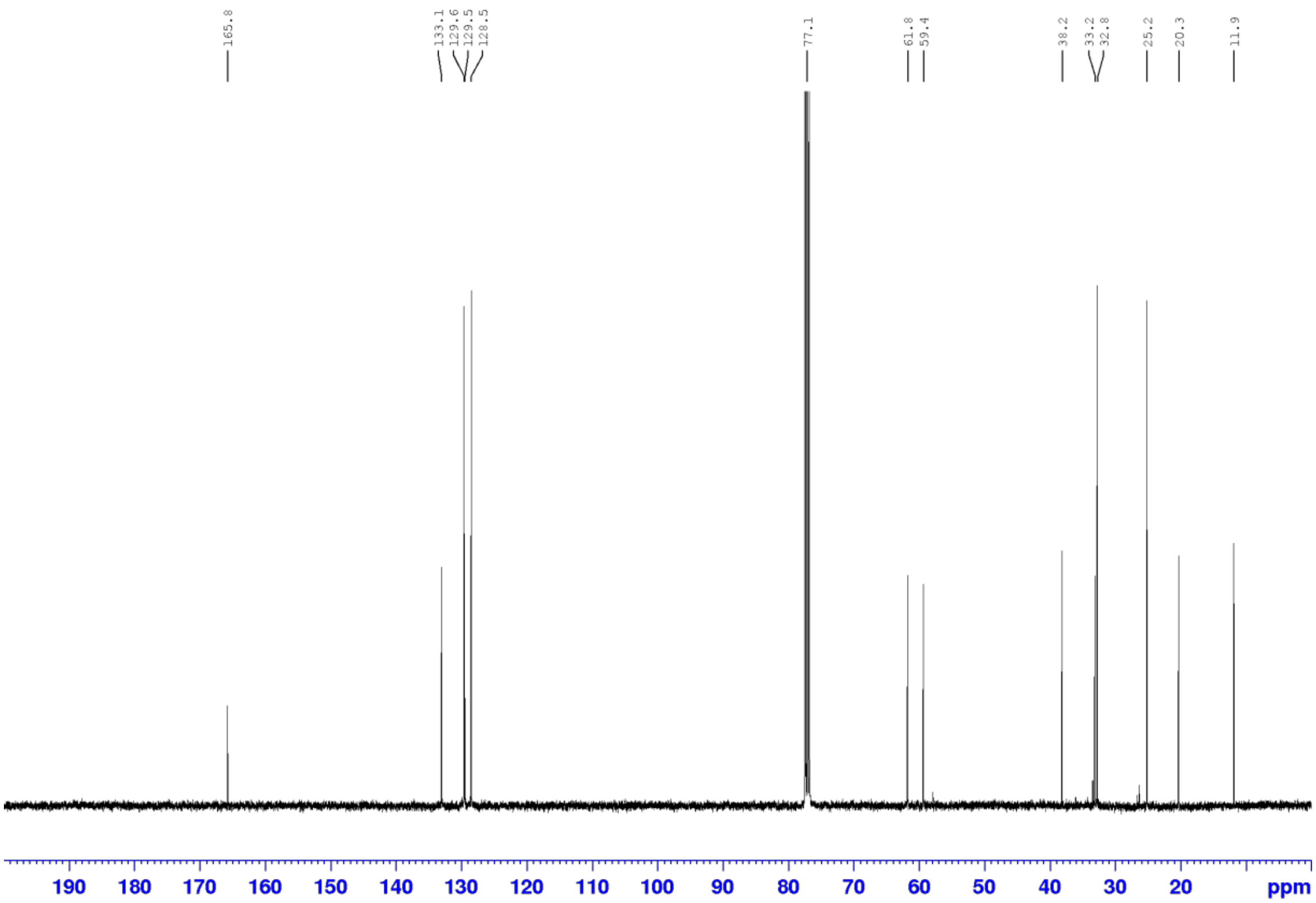

O-benzoyl-N-(2-cyclohexylethyl)-N-propylhydroxylamine (S8)

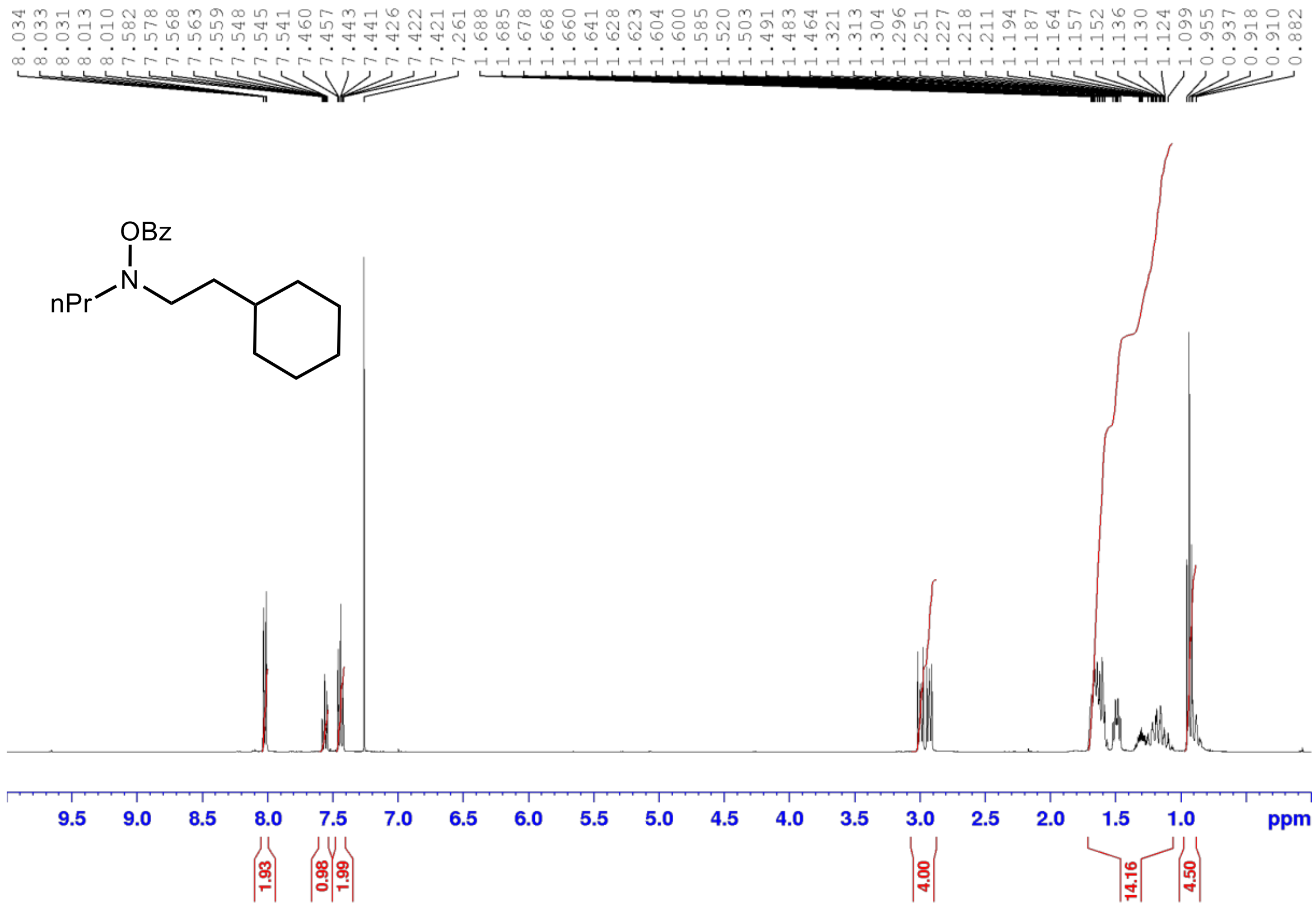

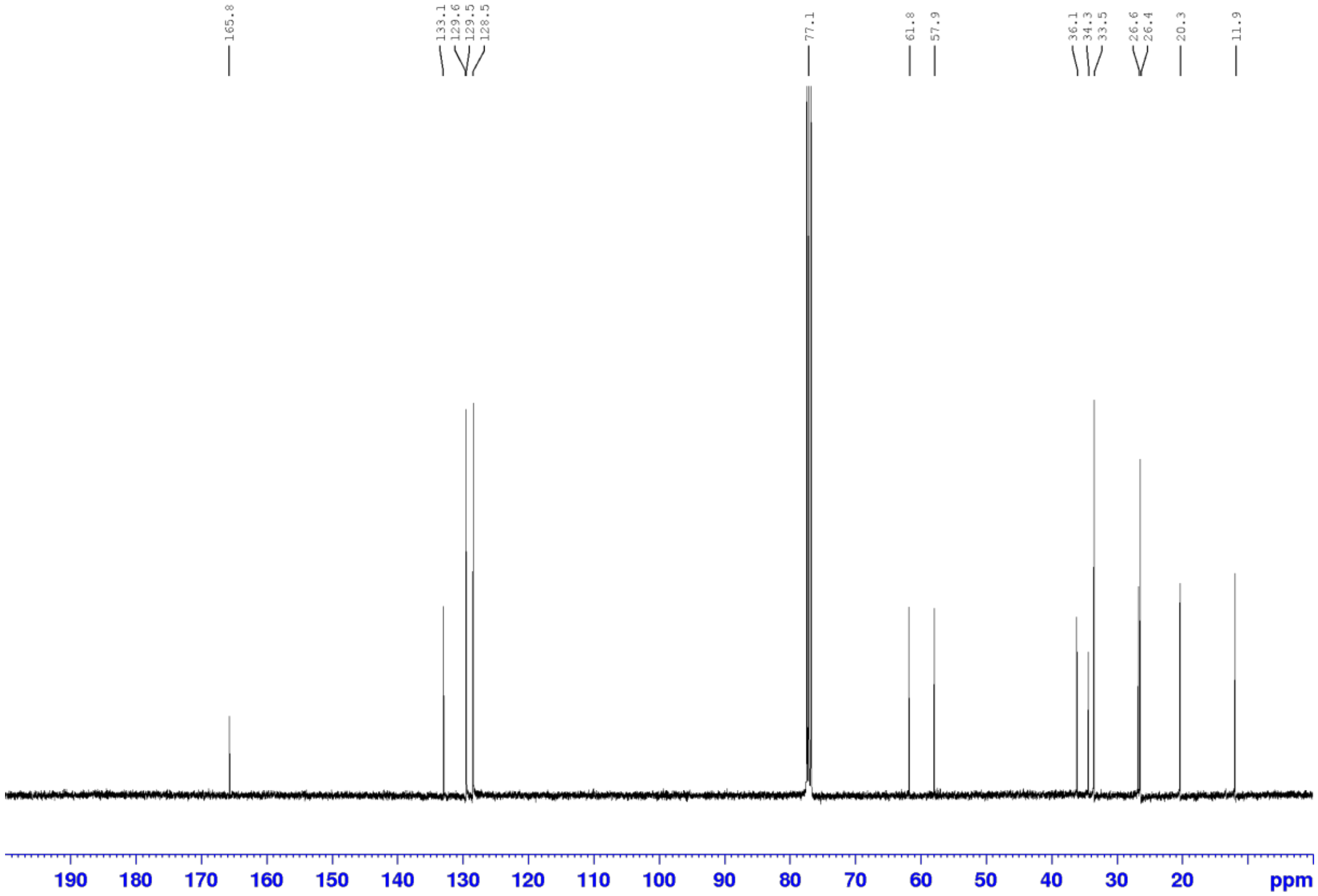

2-pentylpiperidin-1-yl benzoate (S9)

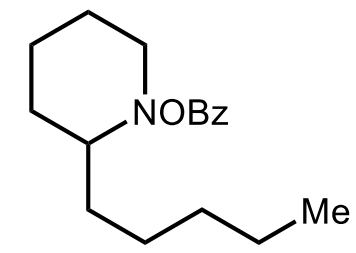

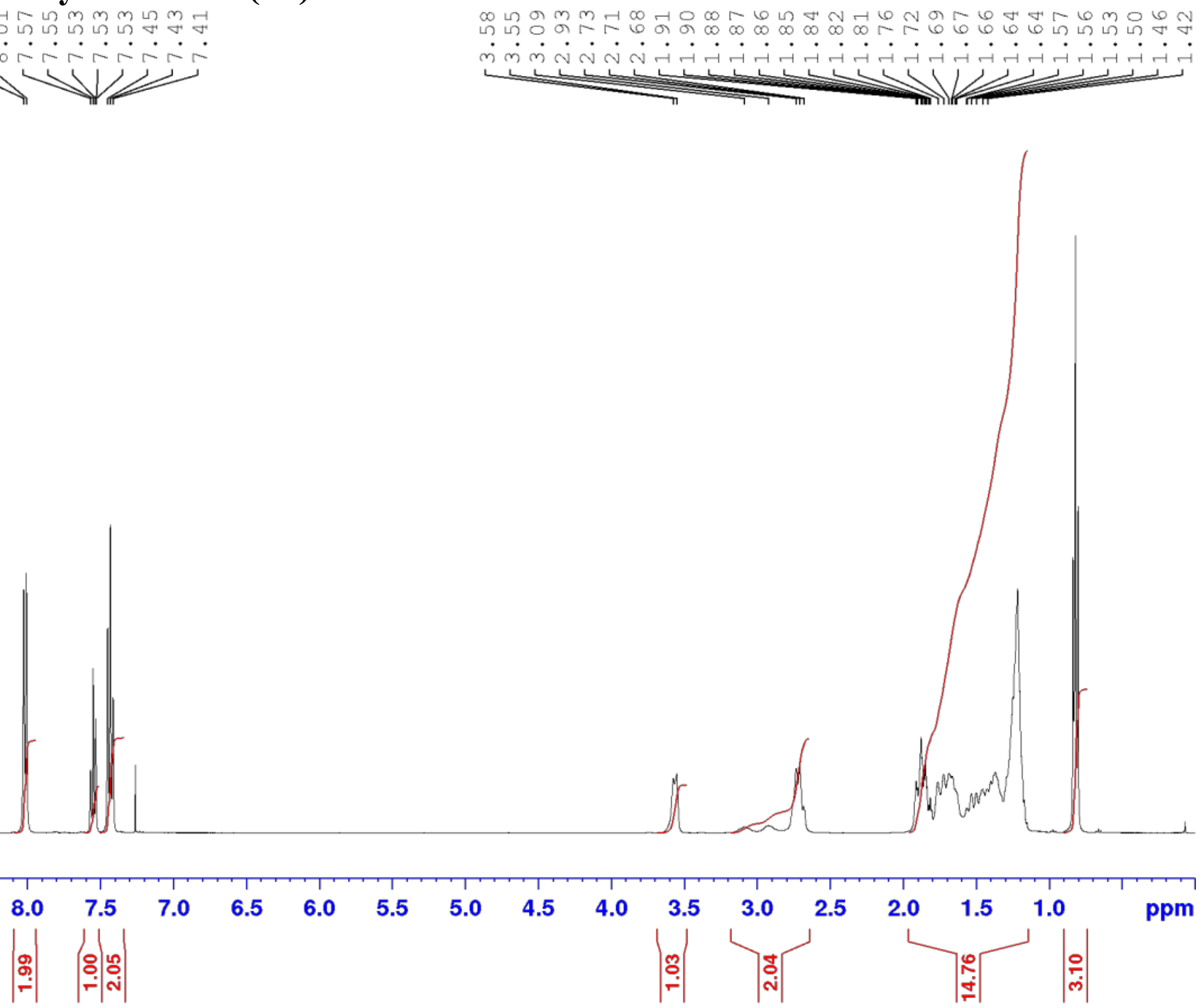

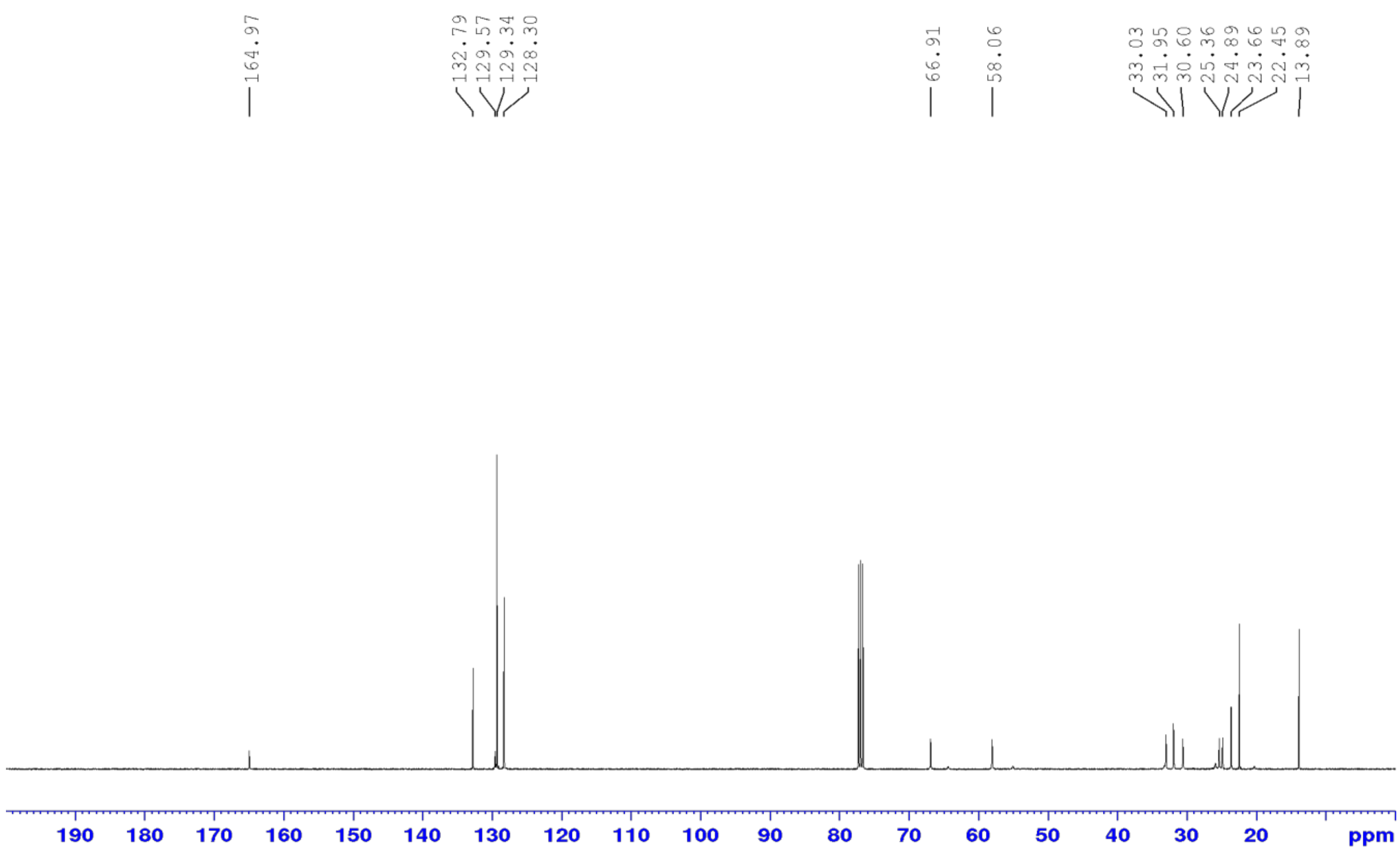

O-benzoyl-N-pentyl-N-propylhydroxylamine (S10)

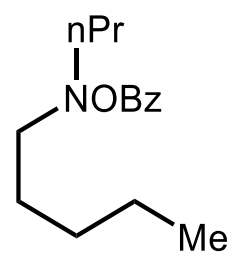

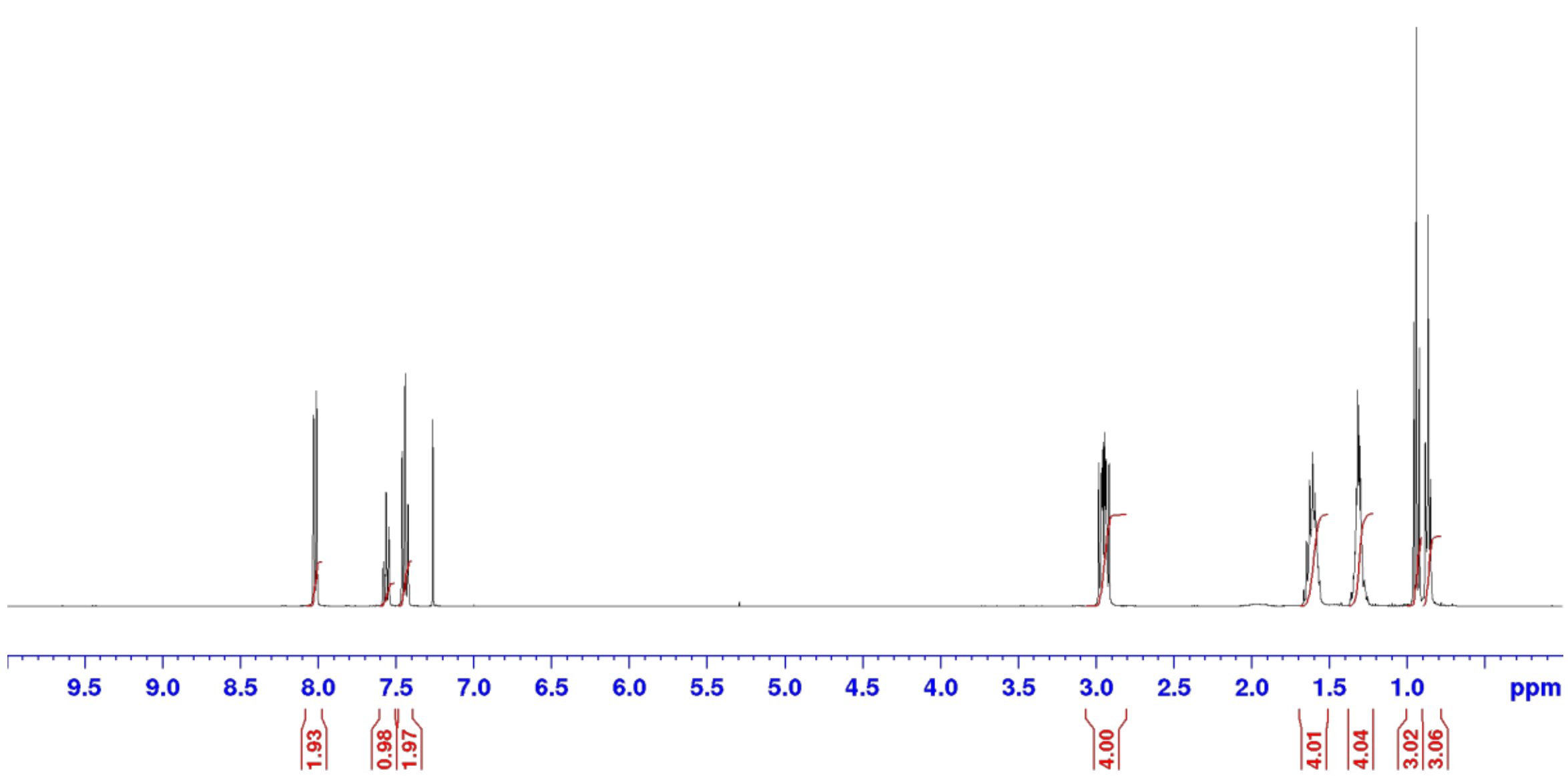

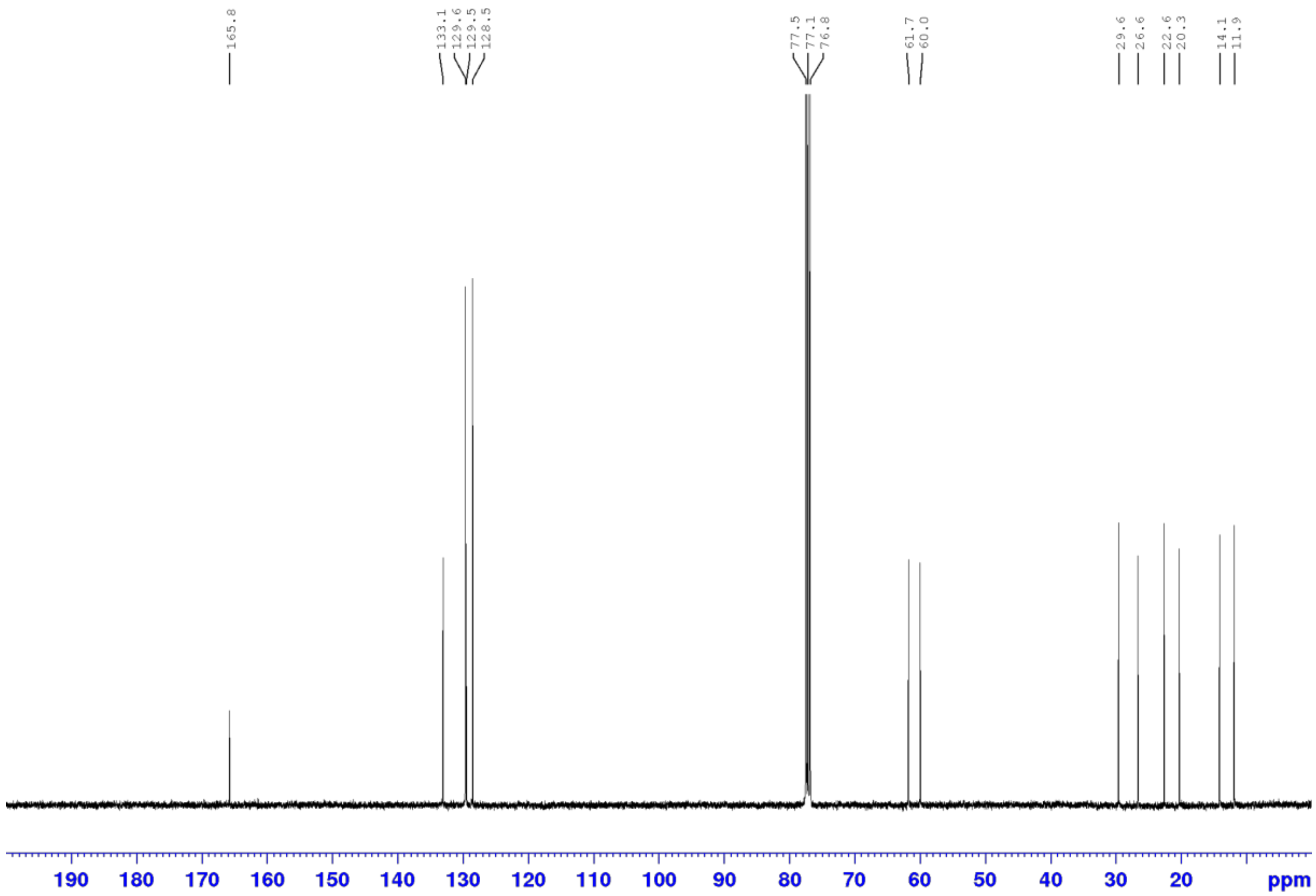

O-benzoyl-N-(2,2-dimethylpentyl)-N-propylhydroxylamine (S11)

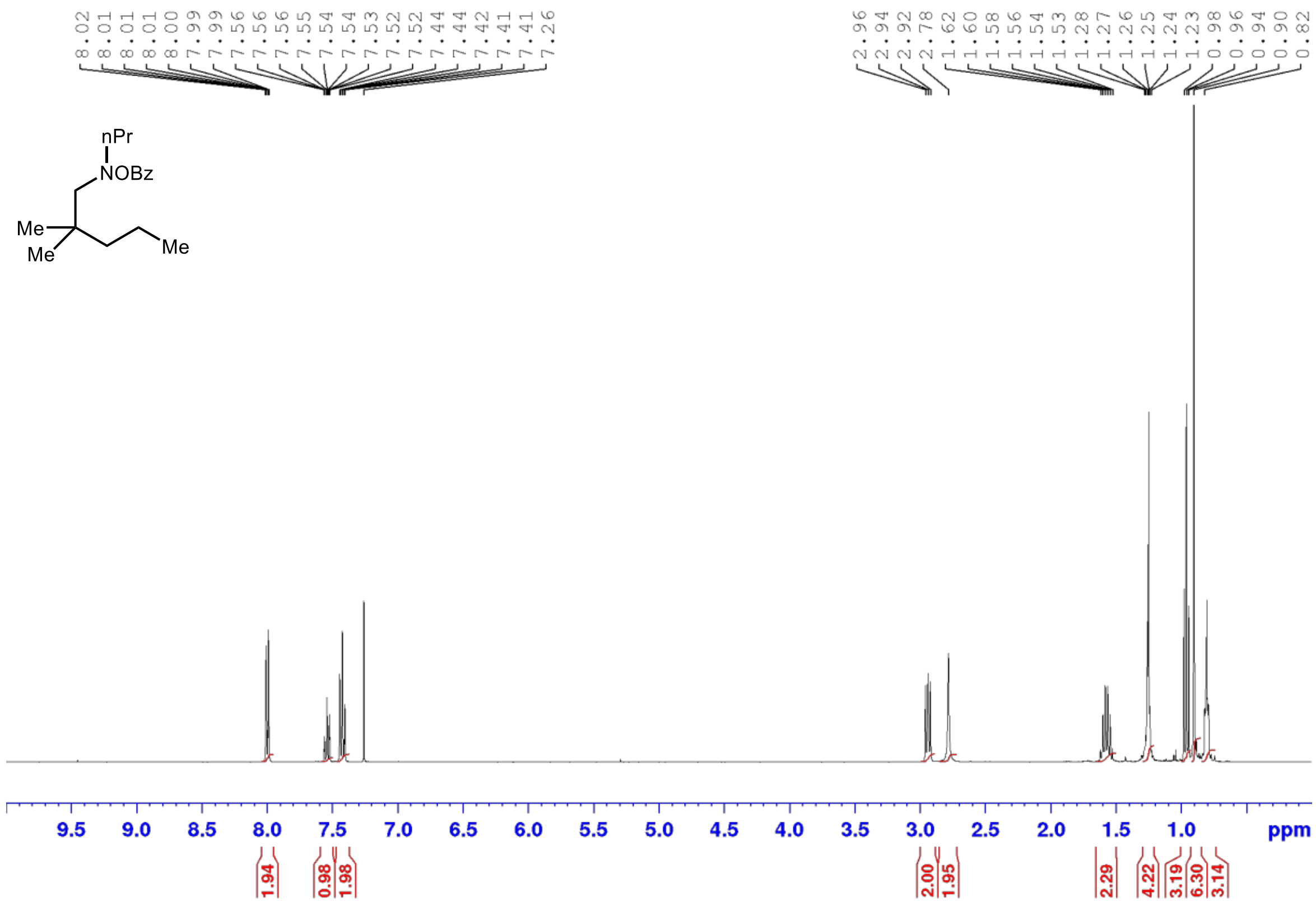

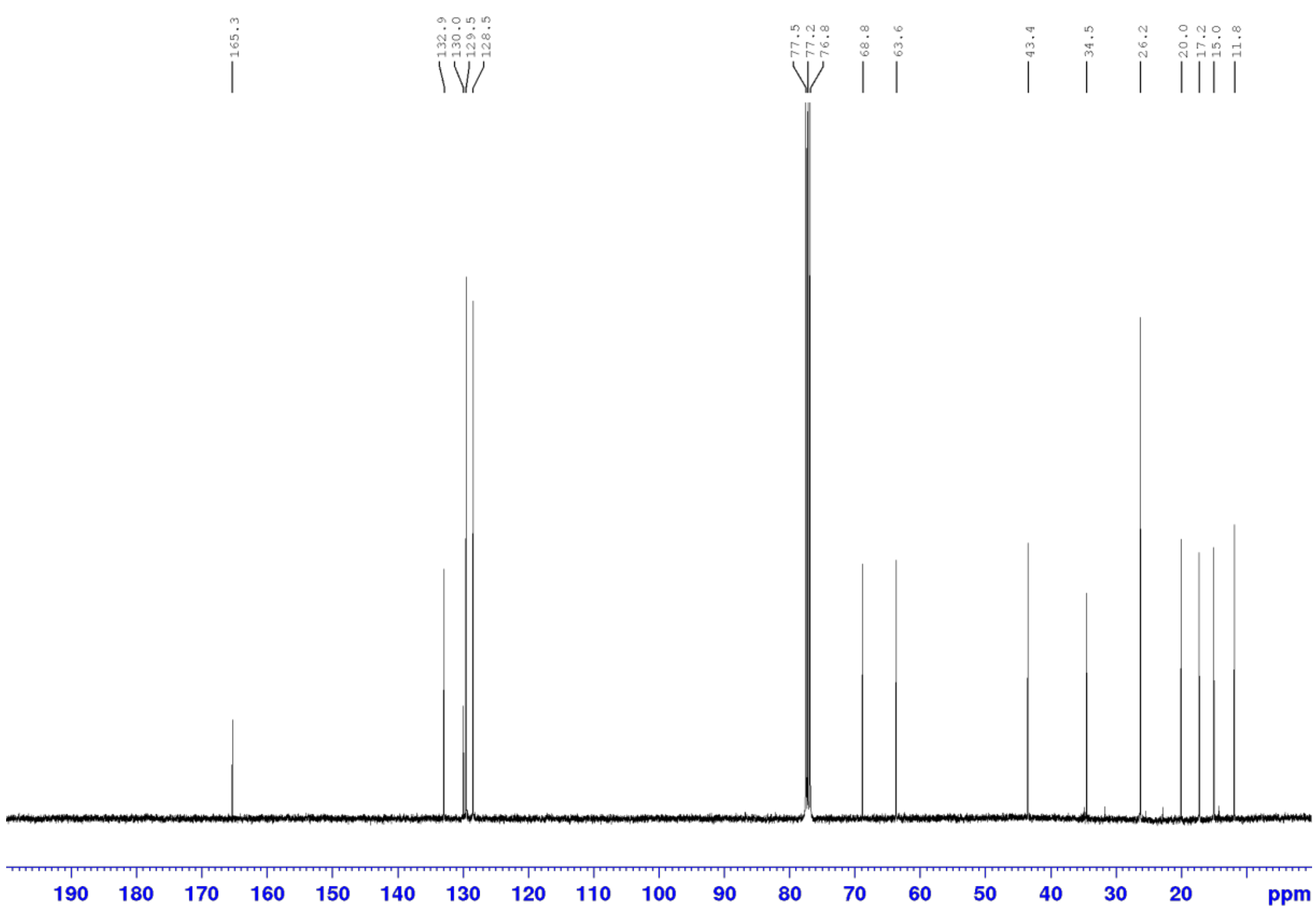

***O*-benzoyl-*N*-hexylhydroxylamine (S12)**

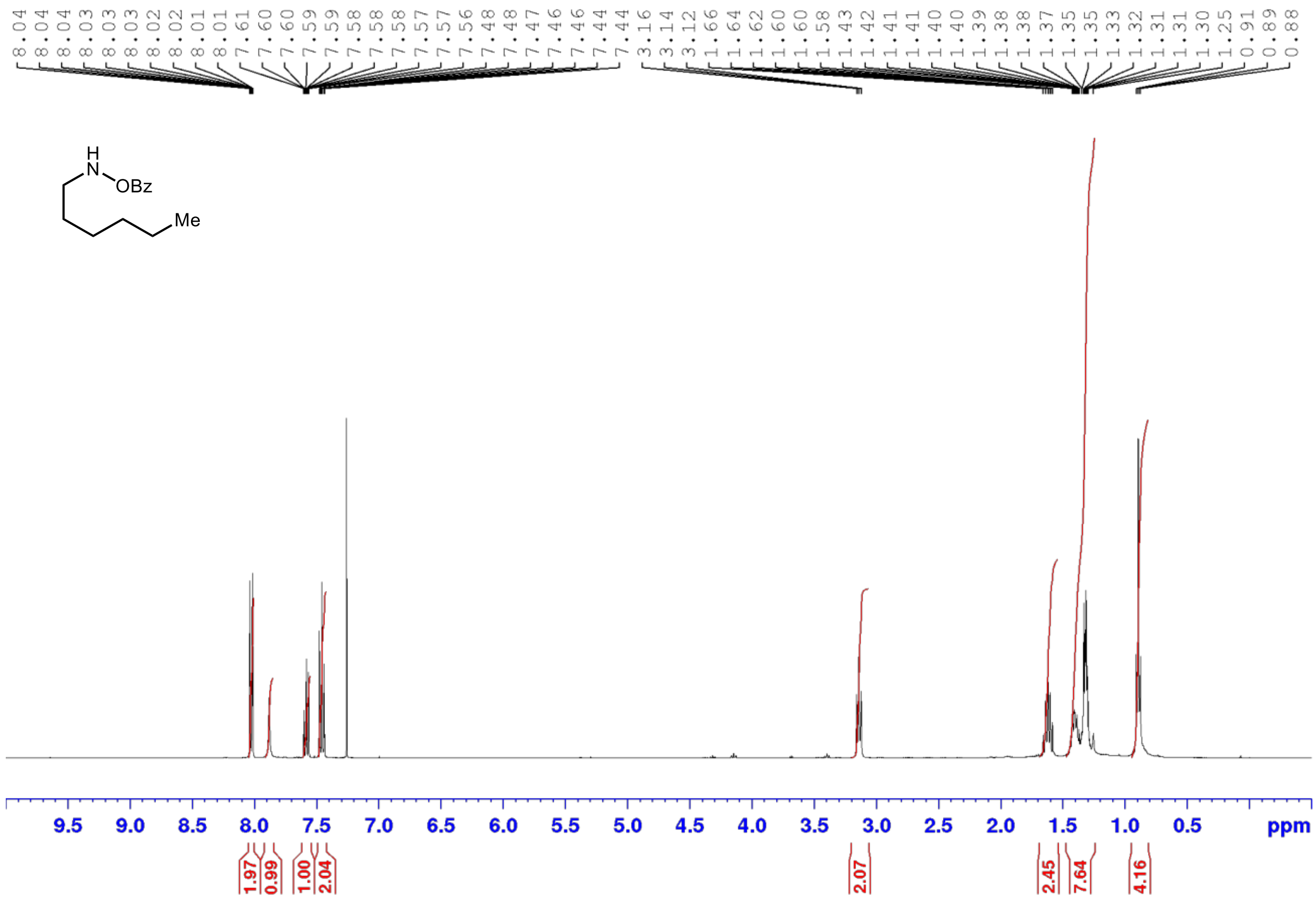

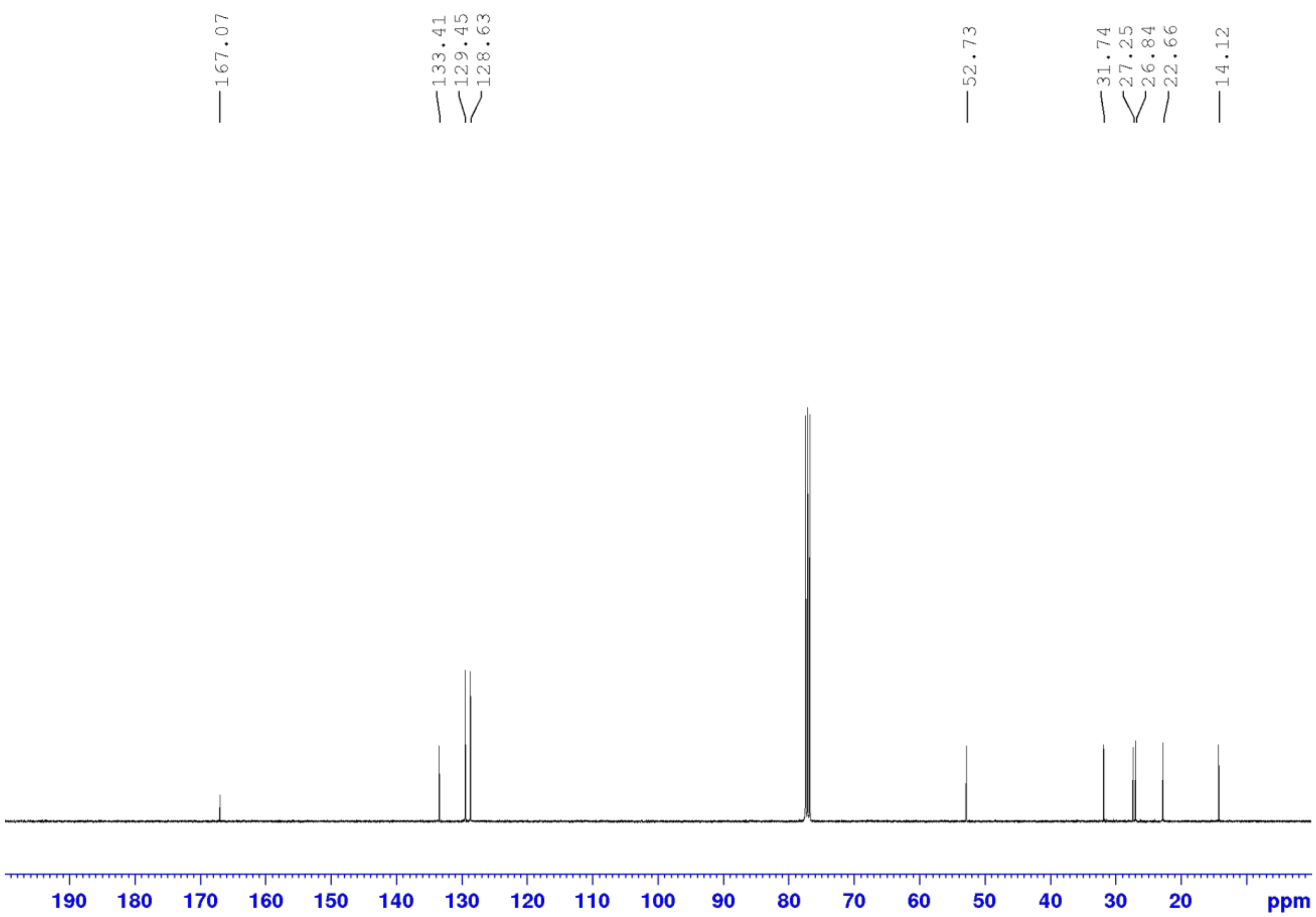

***O*-benzoyl-*N*-(heptan-2-yl)hydroxylamine (S13)**

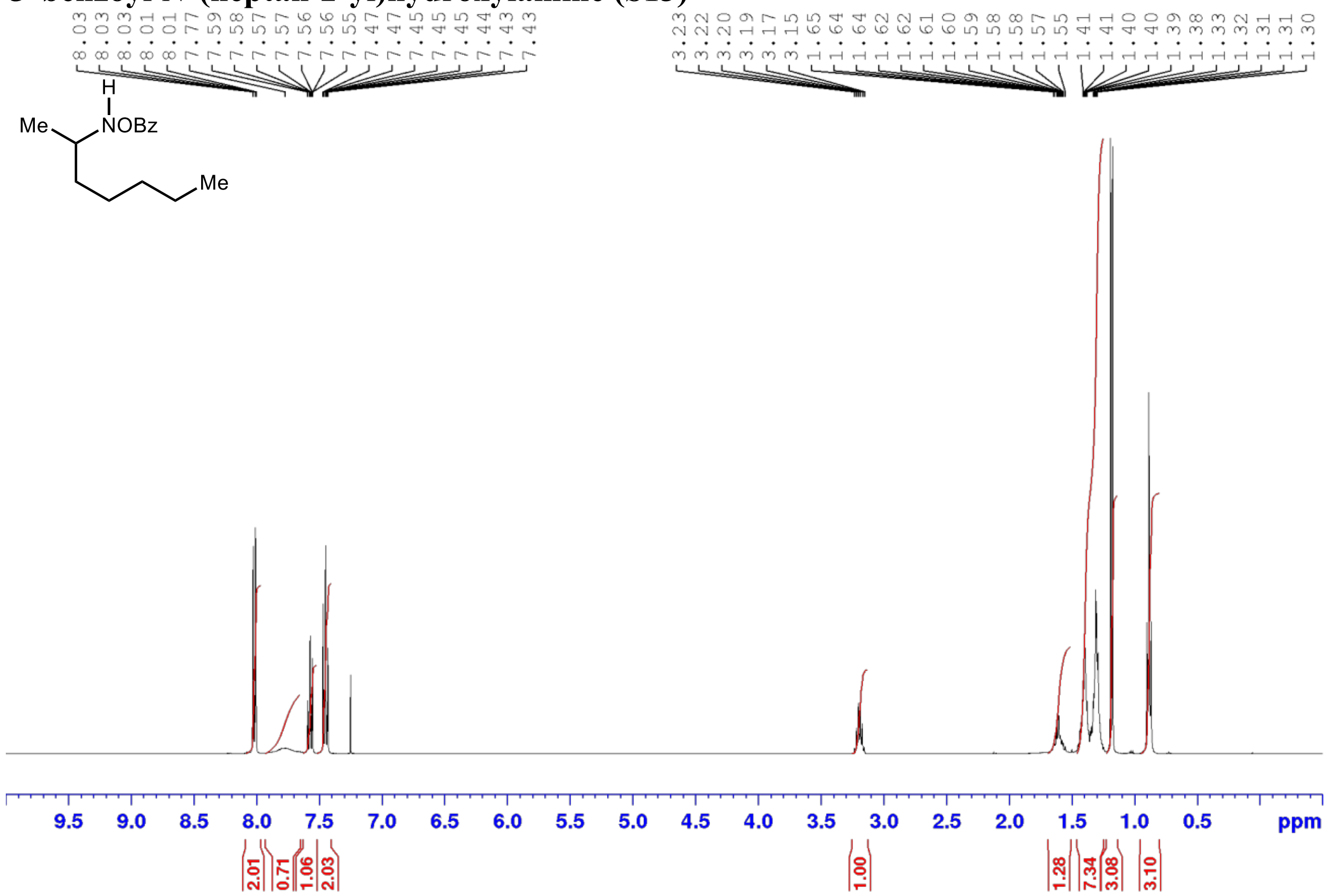

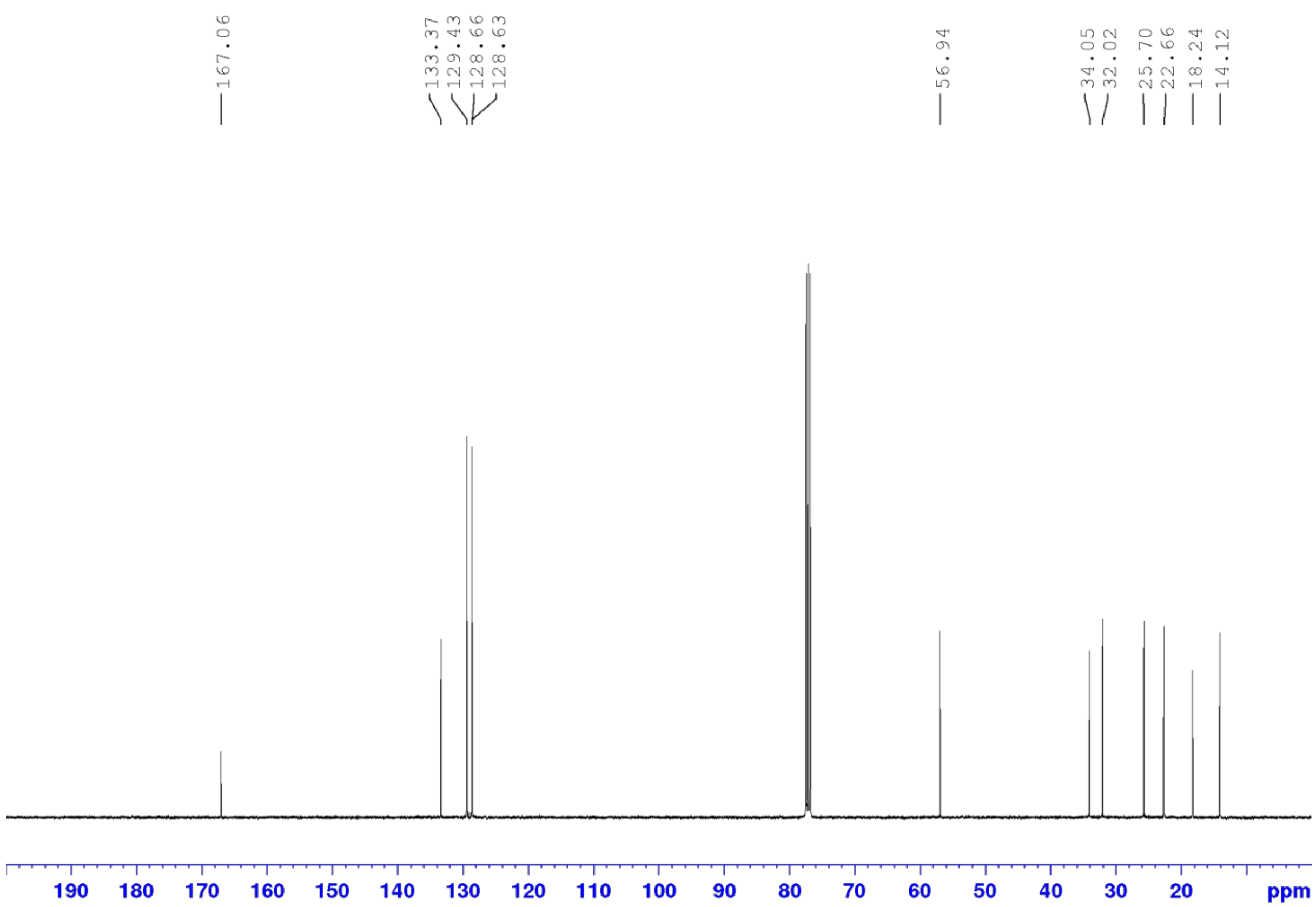

***O*-benzoyl-*N*-hexylhydroxylamine (S14)**

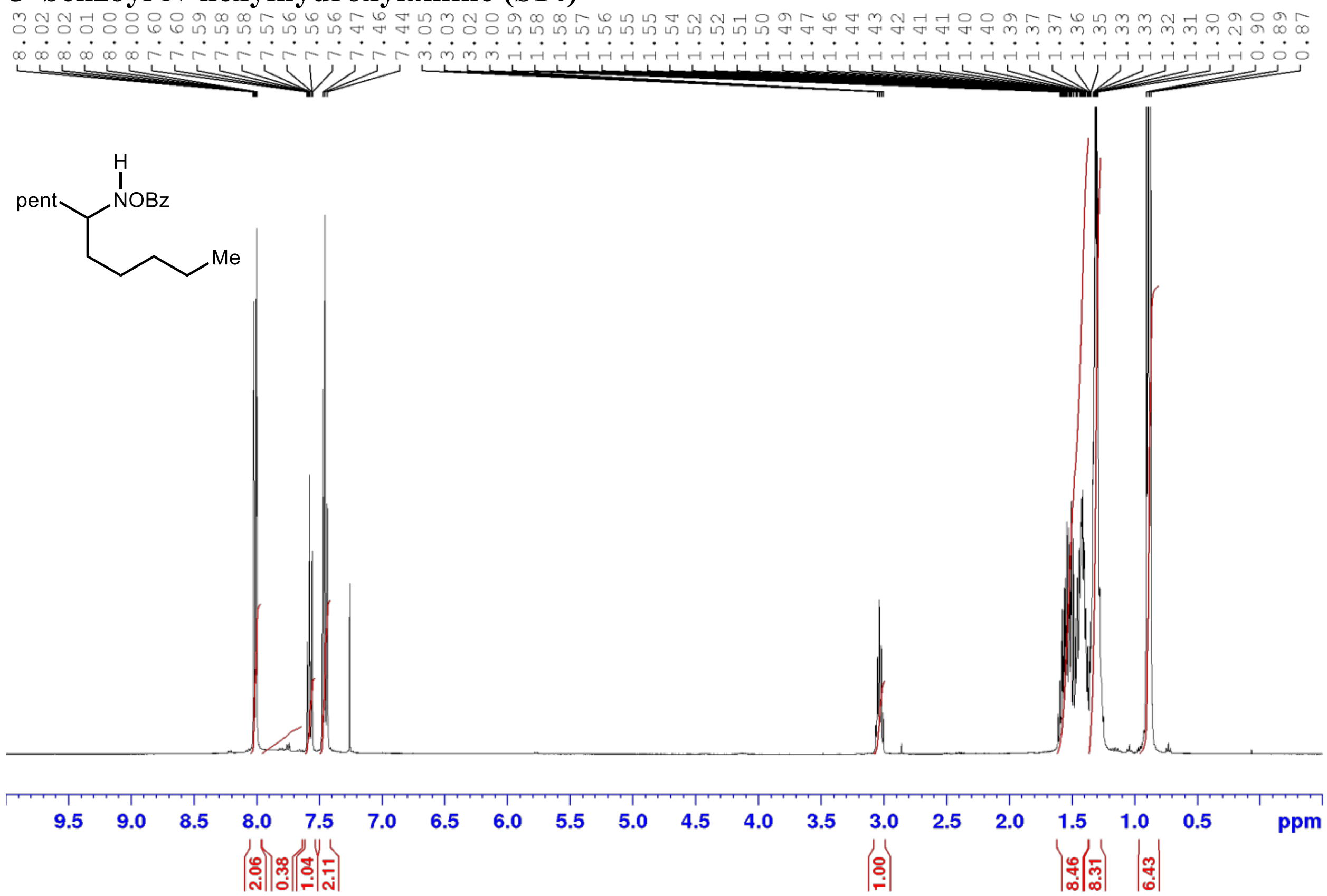

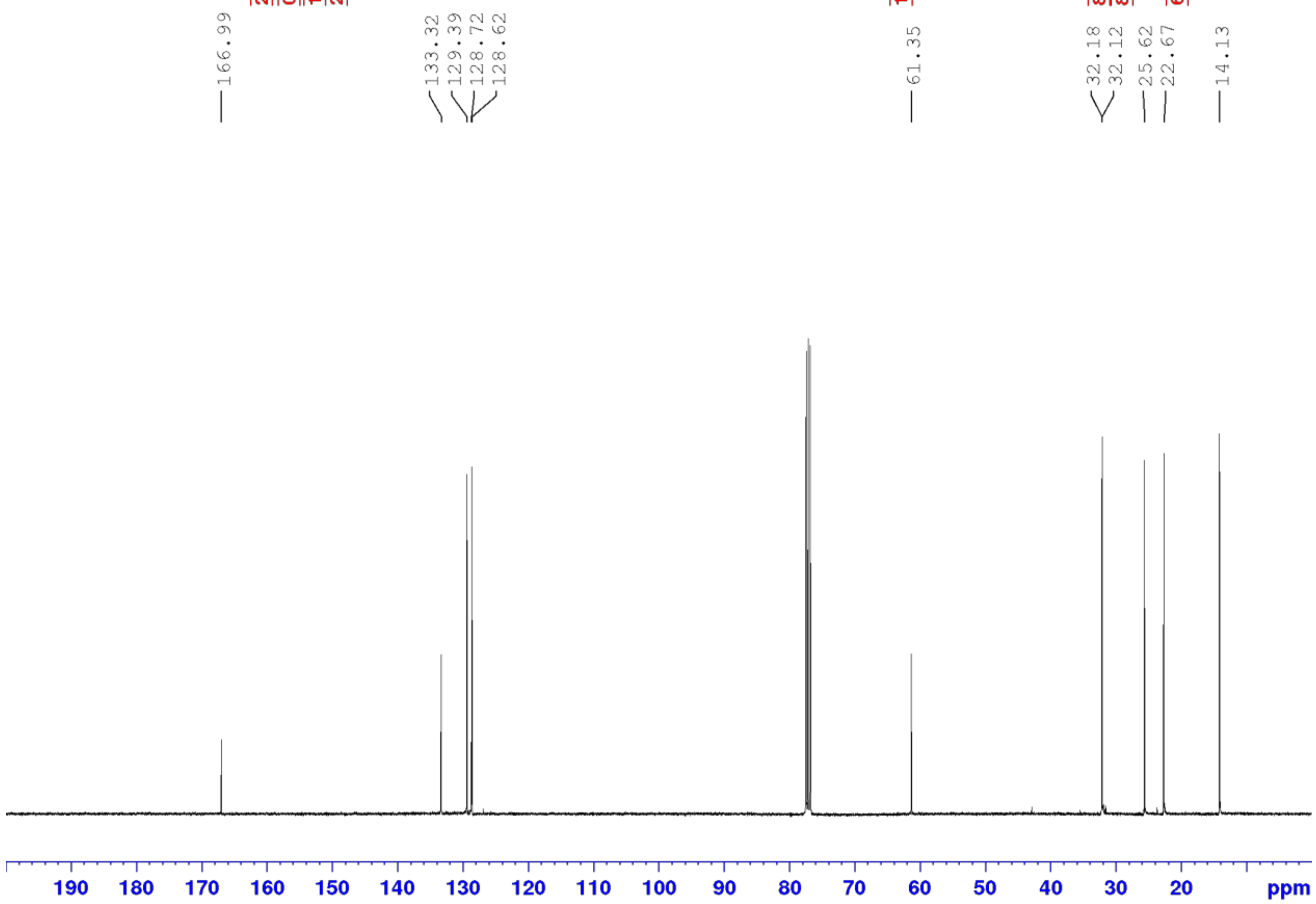

***O*-benzoyl-*N*-(((1*S*,4a*R*,10a*S*)-7-isopropyl-1,4a-dimethyl-1,2,3,4,4a,9,10,10a-octahydrophenanthren-1-yl)methyl)hydroxylamine** **(S15)**

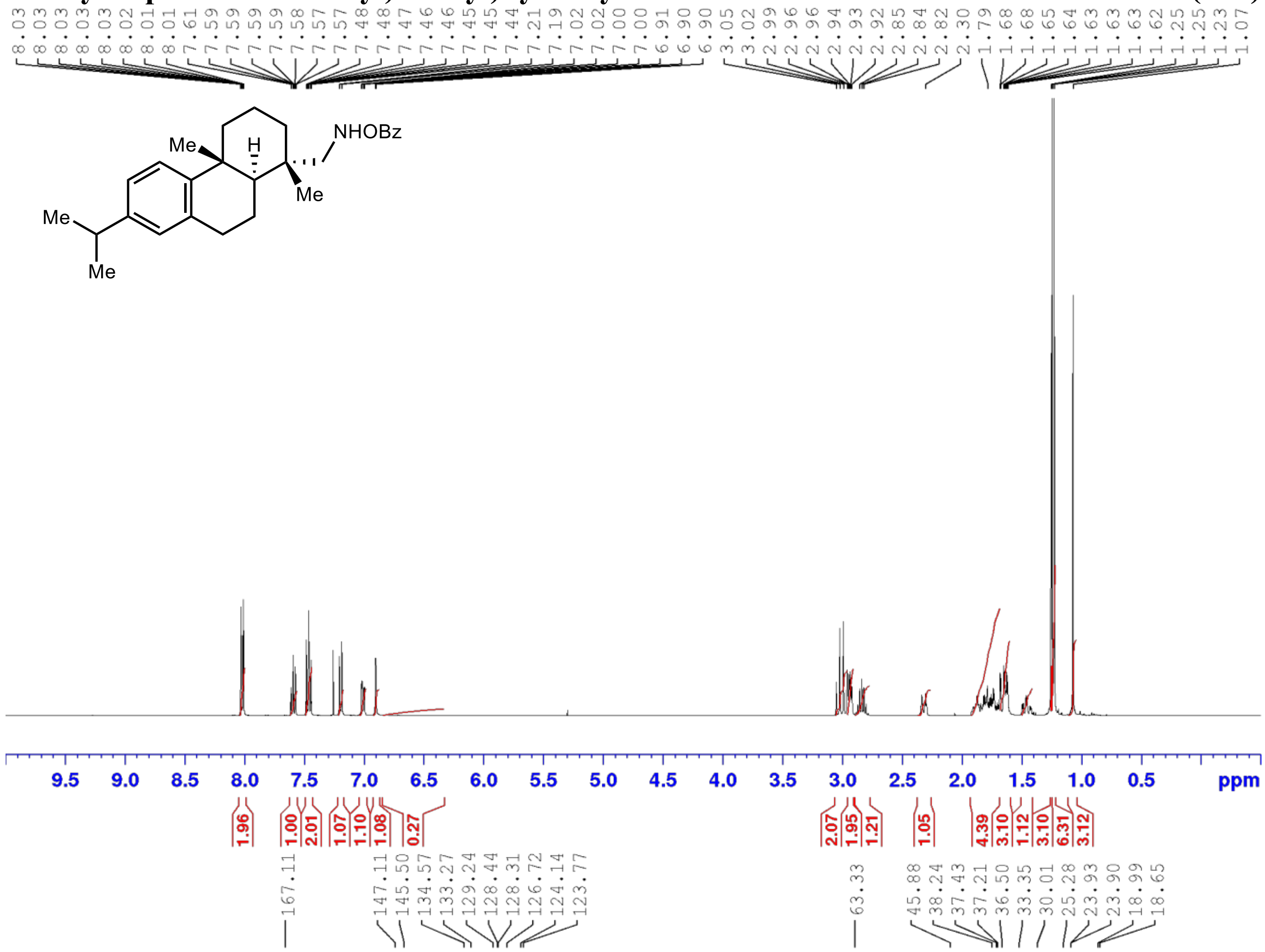

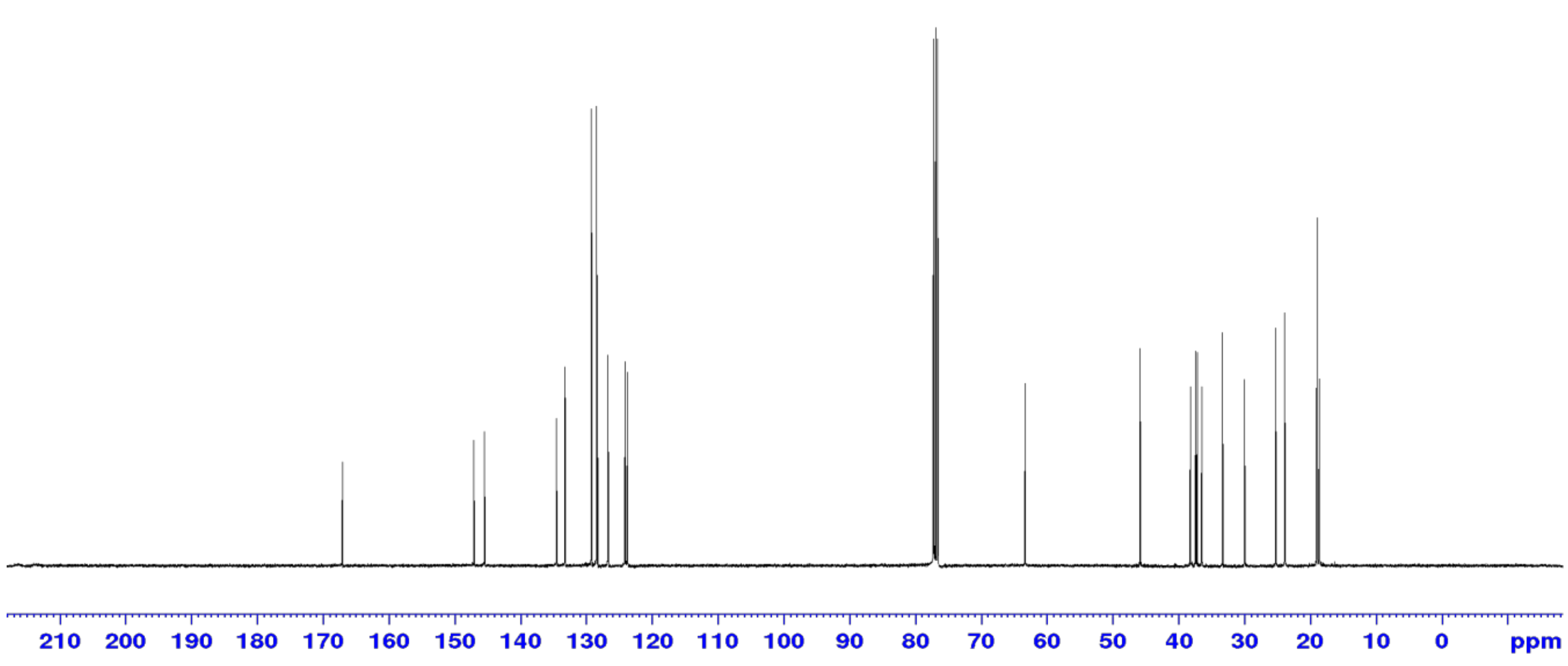

methyl 2-((benzoyloxy)amino)heptanoate (S16)

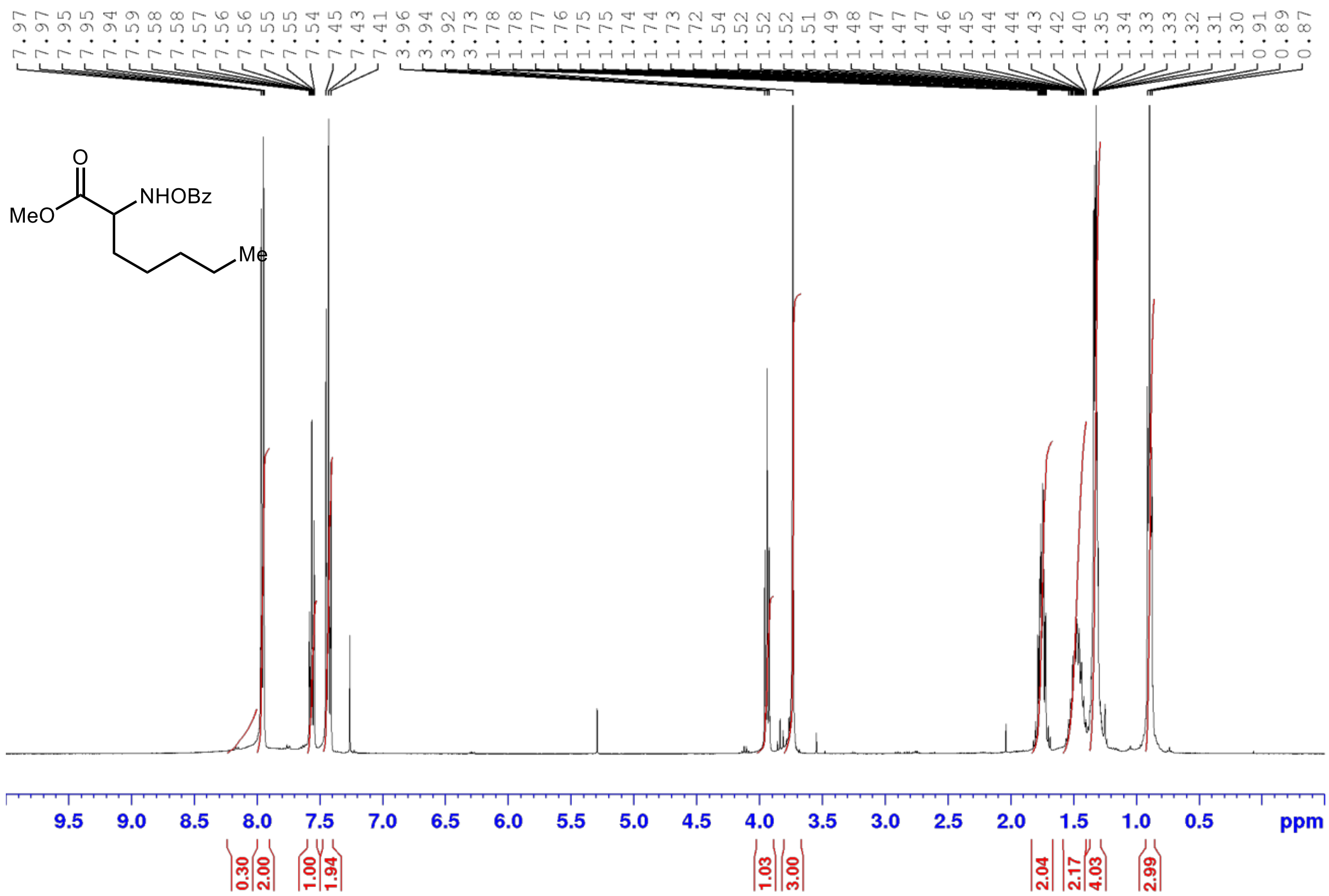

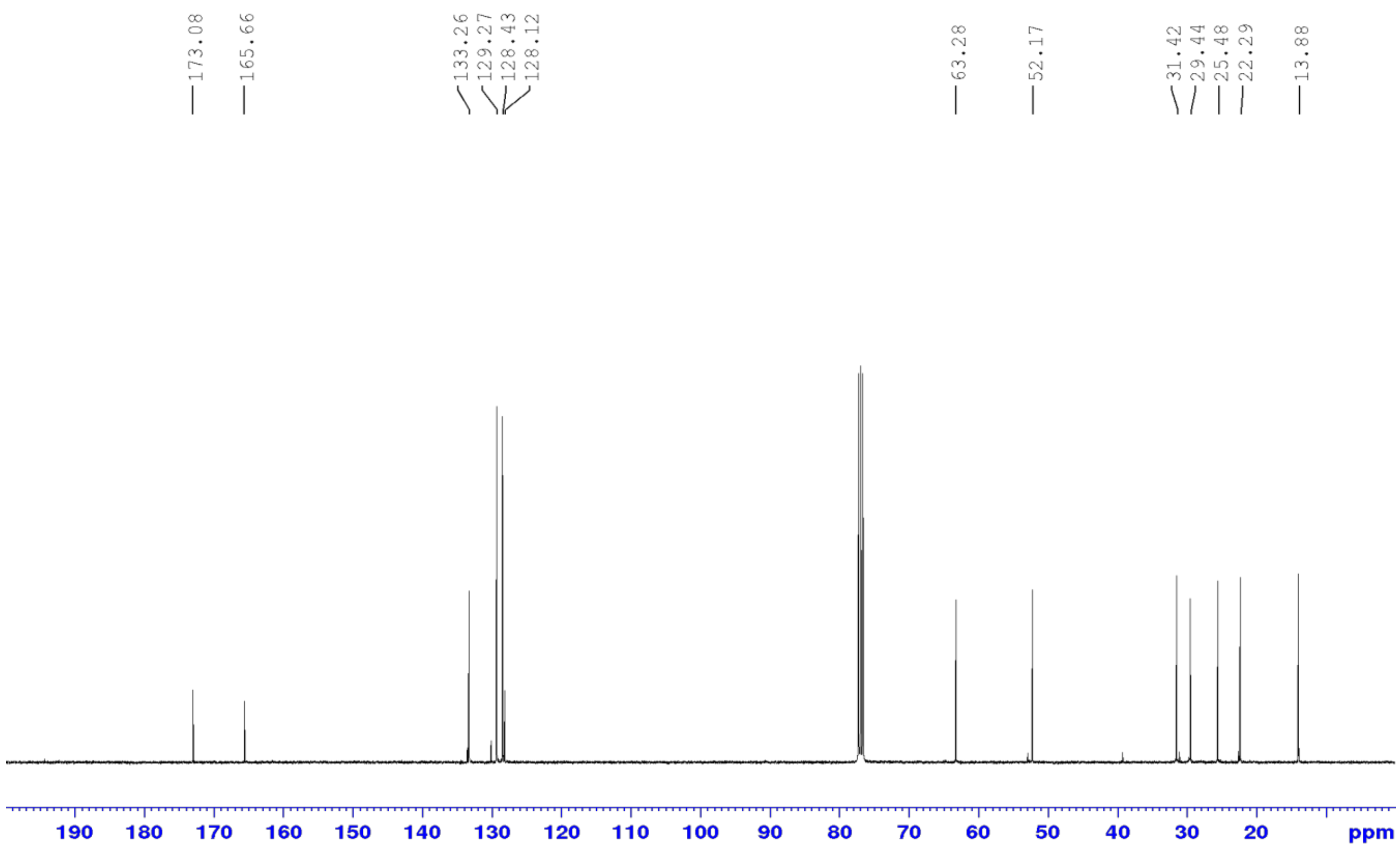

O-benzoyl-N-(pent-4-en-1-yl)-N-propylhydroxylamine (S17)

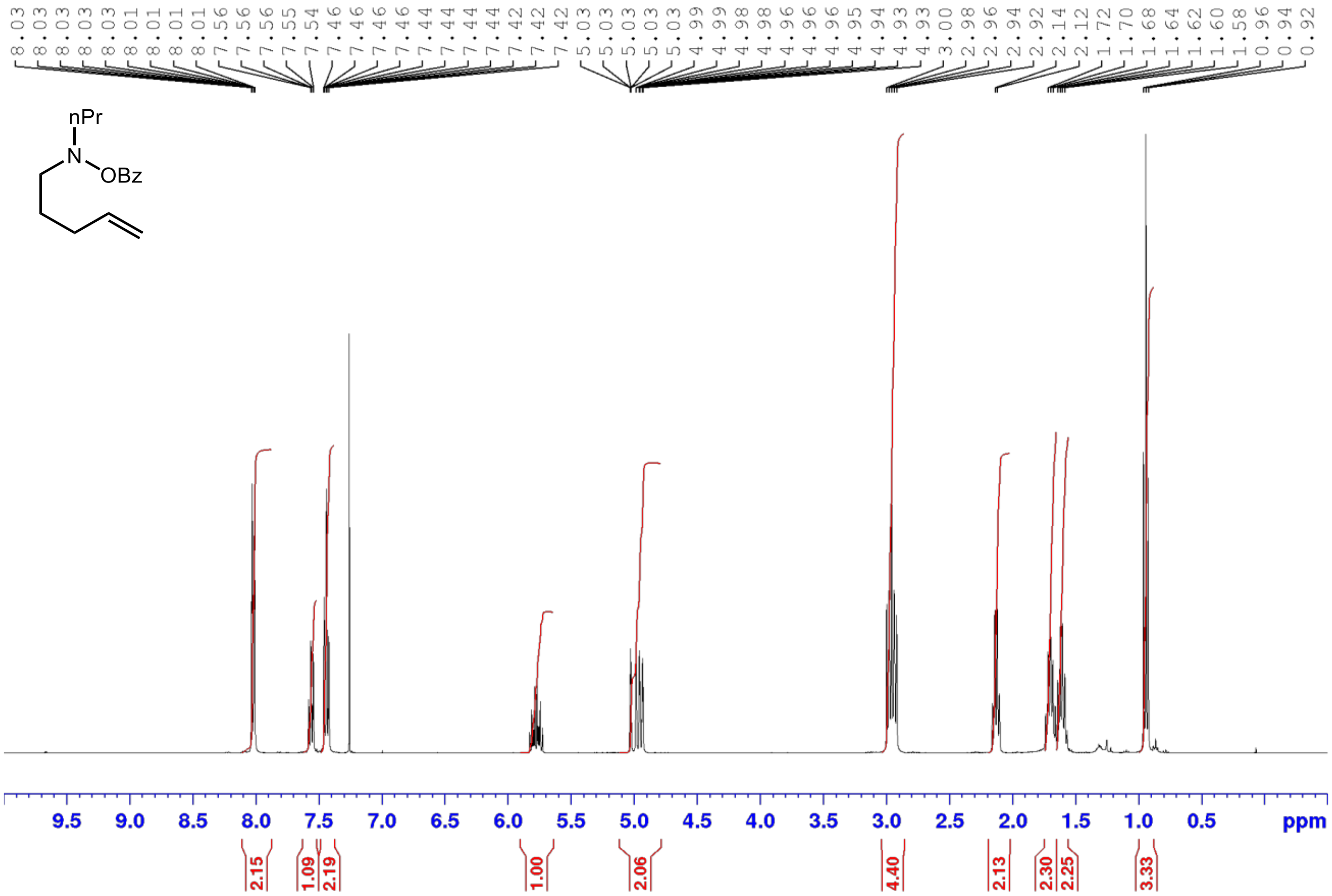

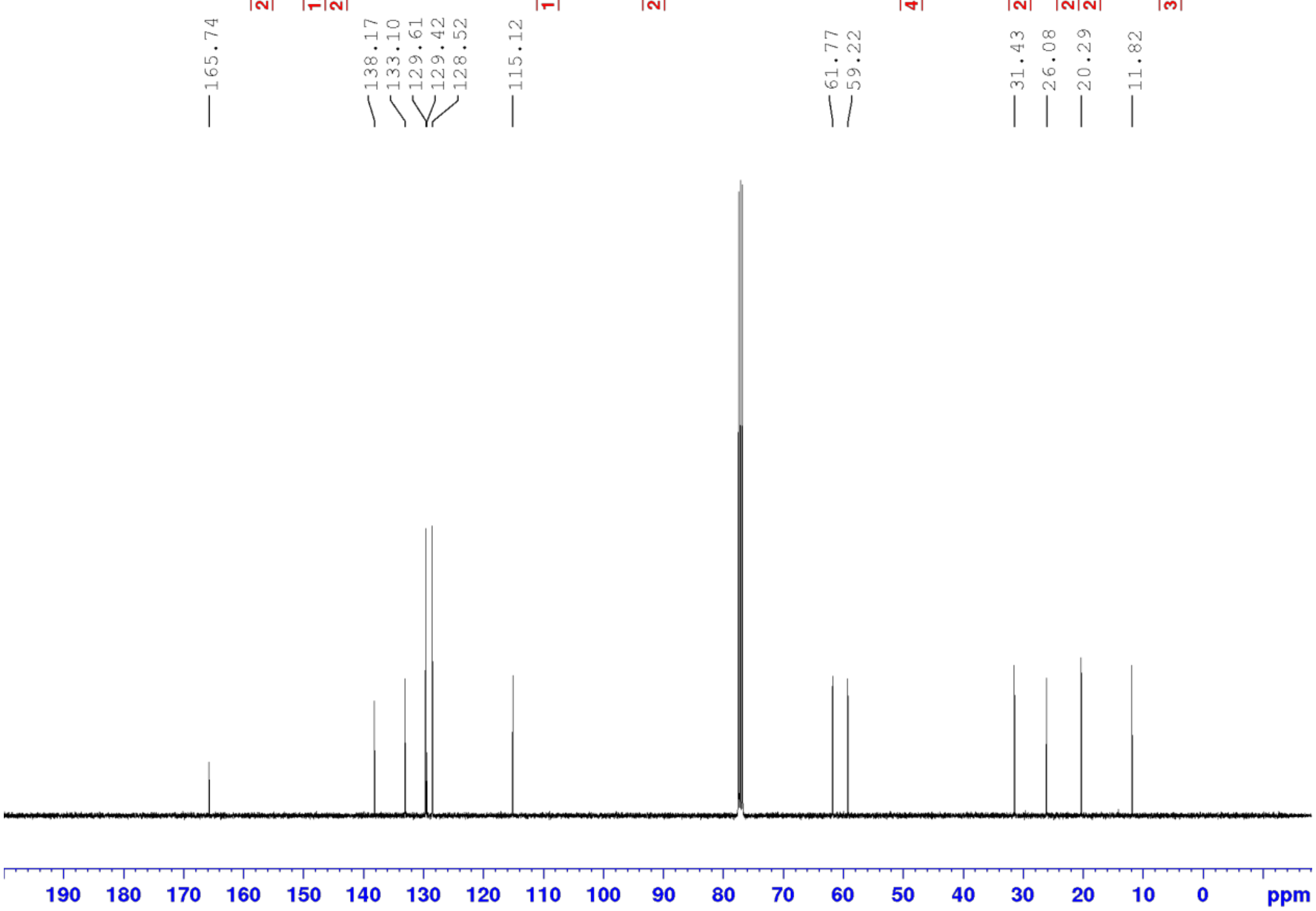

O-benzoyl-N-(3,7-dimethyloctyl)-N-propylhydroxylamine (S18)

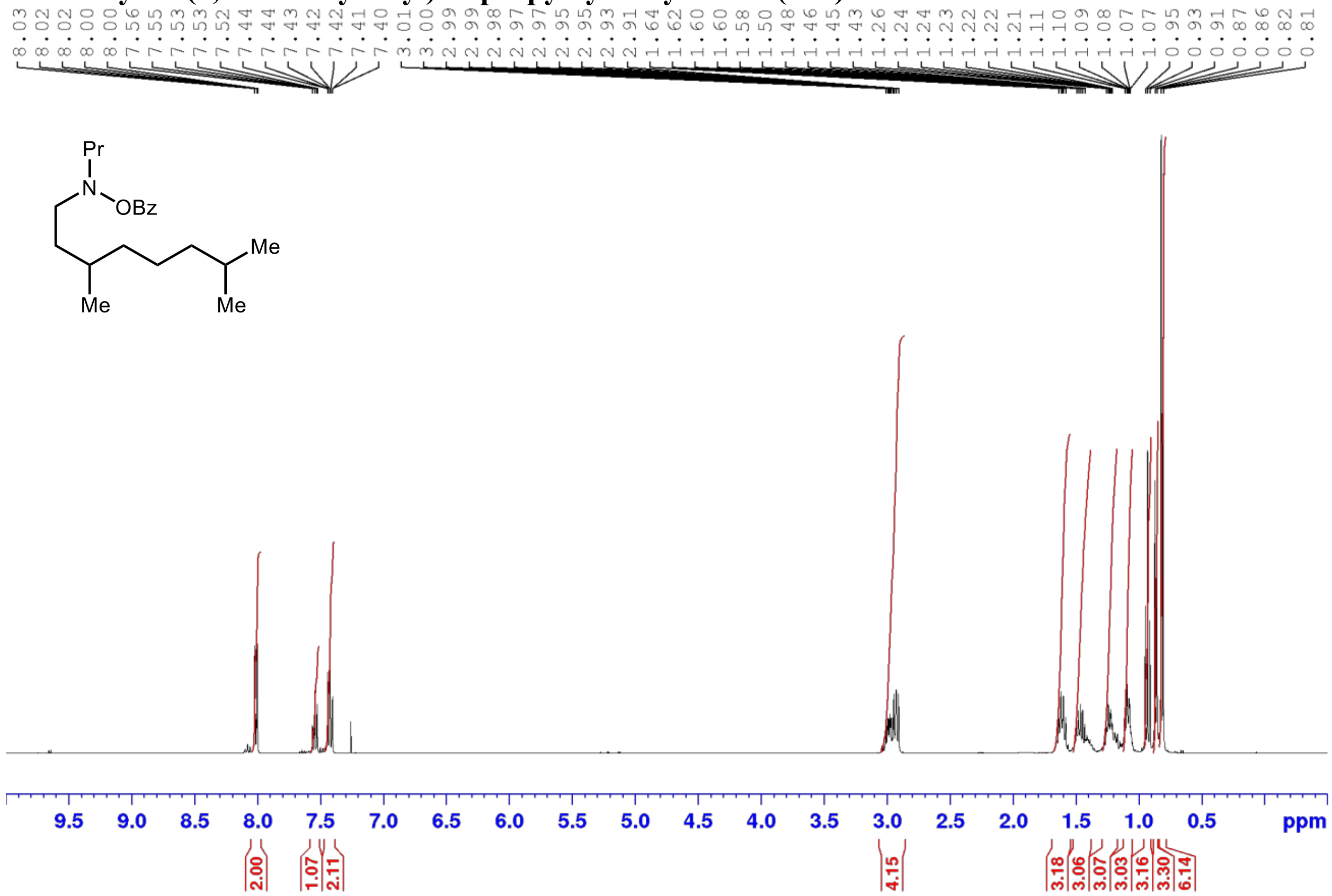

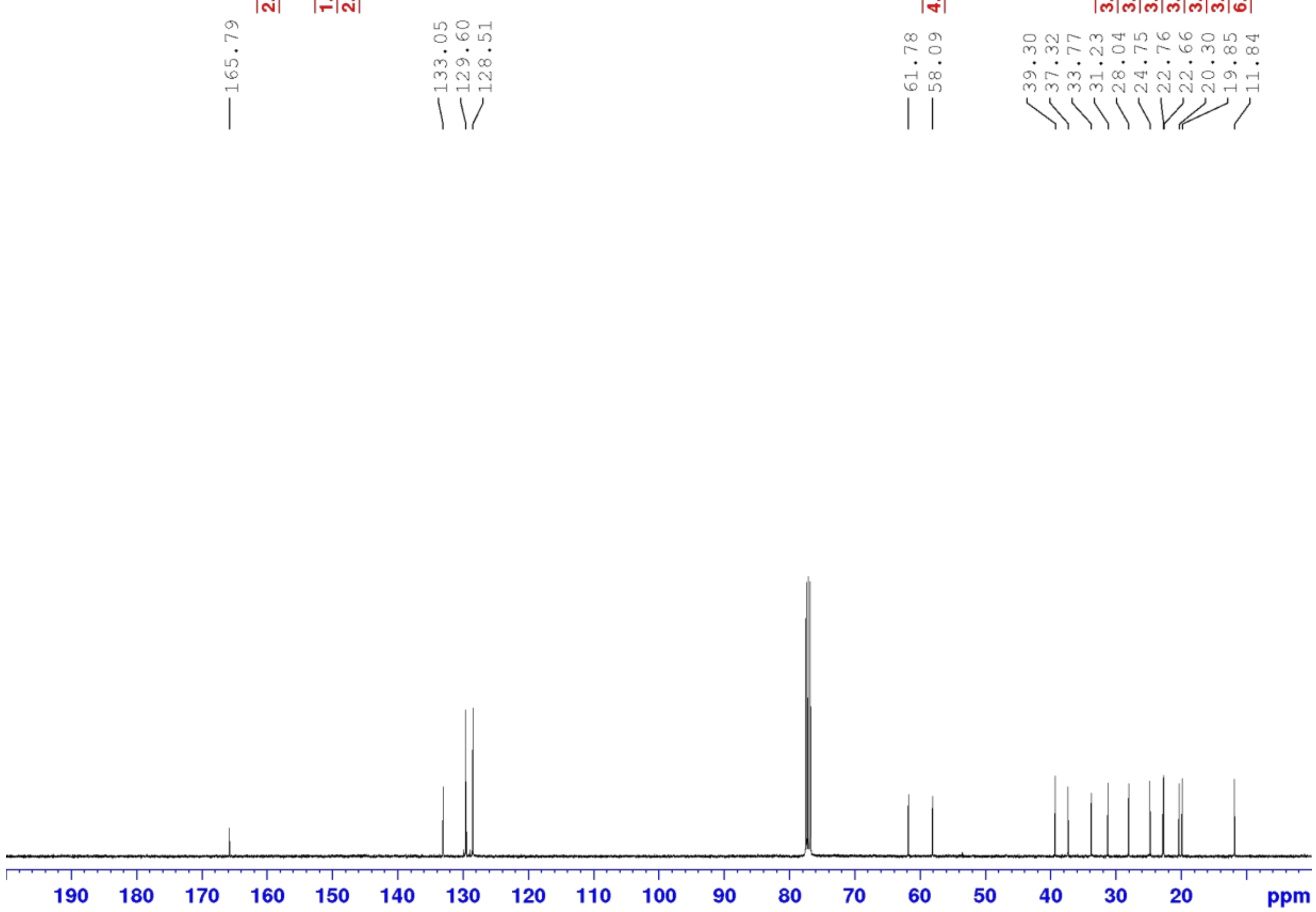

O-benzoyl-N-(oct-1-en-3-yl)hydroxylamine (S19)

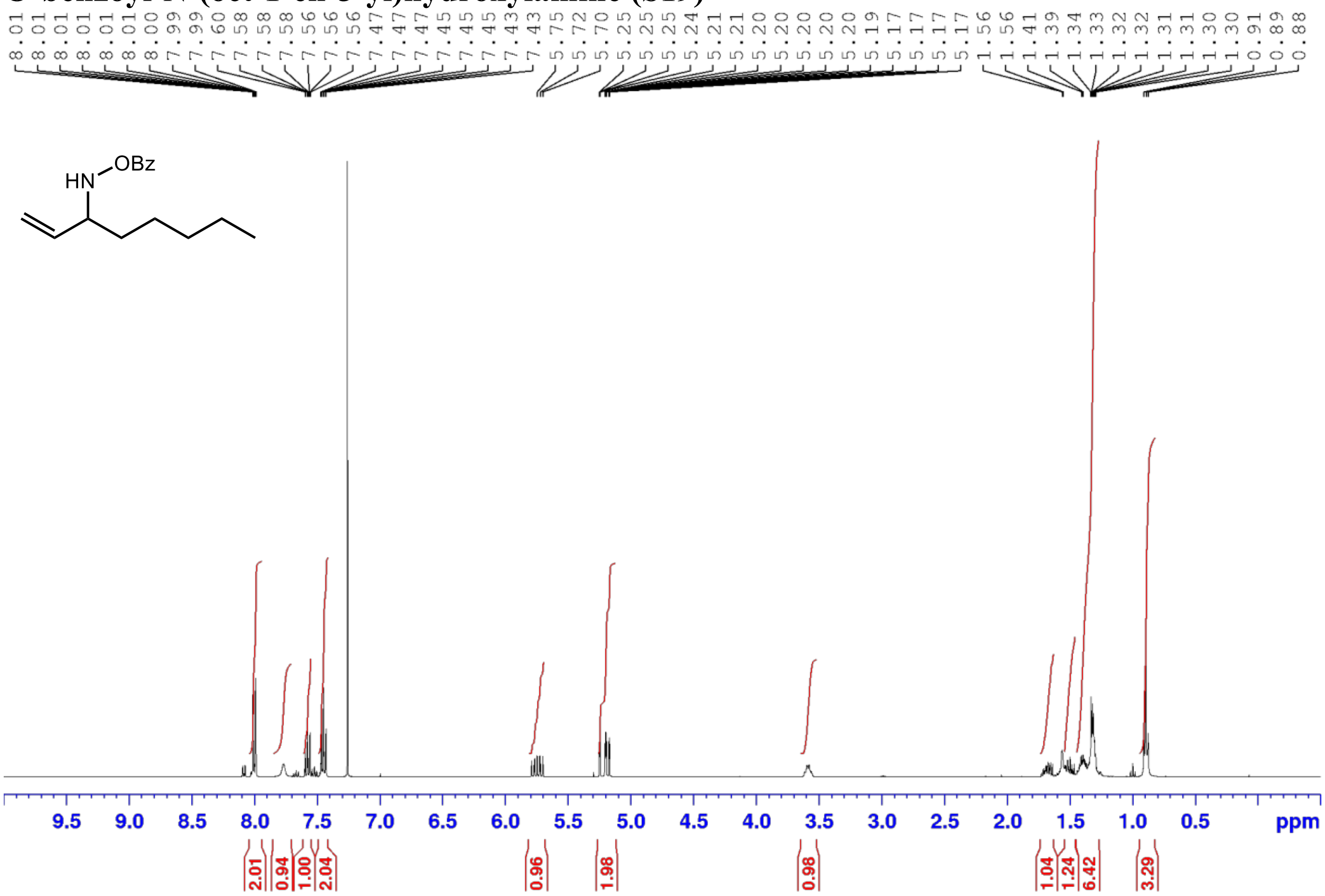

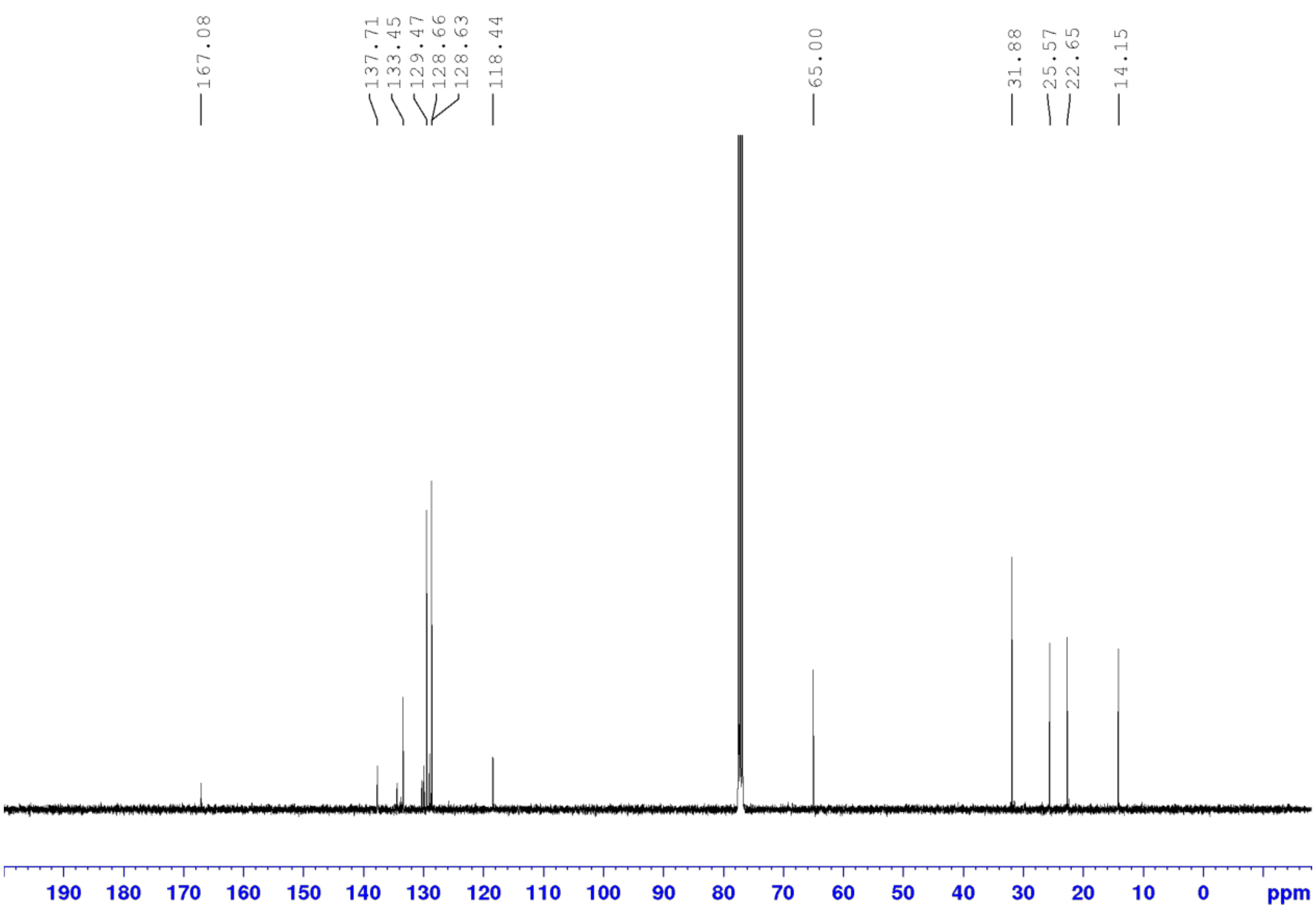

_O_-Benzoyl-N,N-dipropylhydroxylamine (S20)

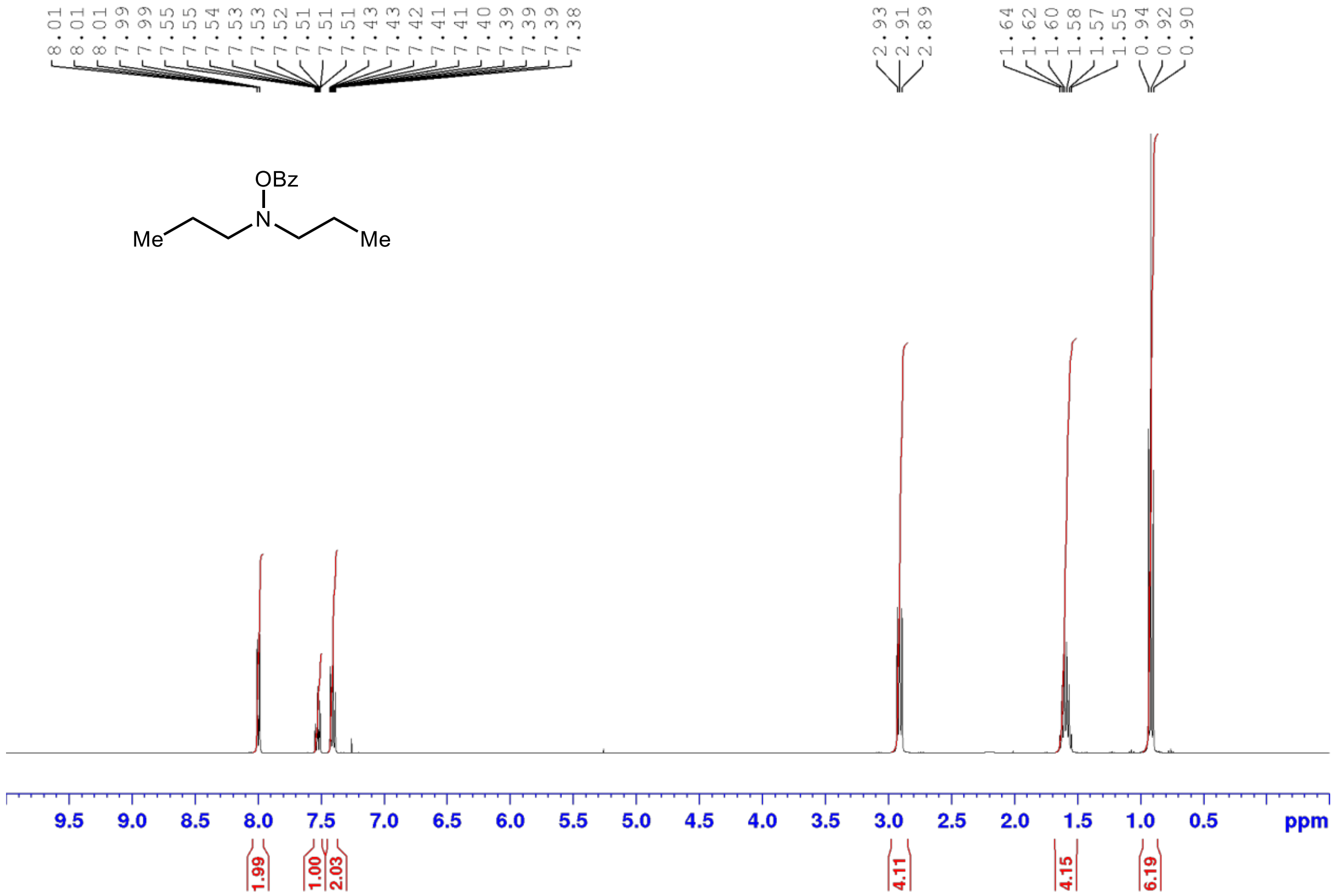

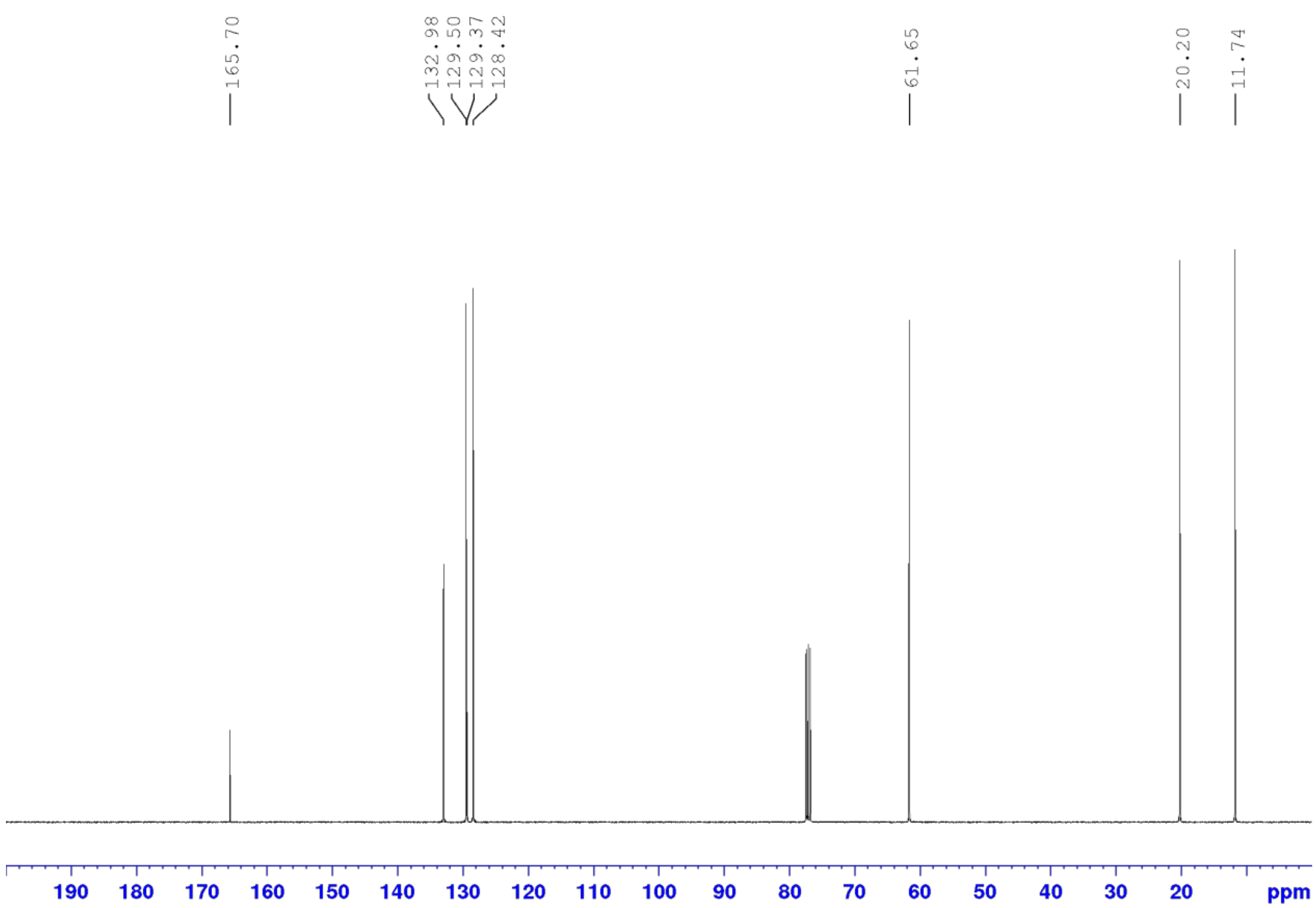

O-benzoyl-N-benzyl-N-hexylhydroxylamine (S21)

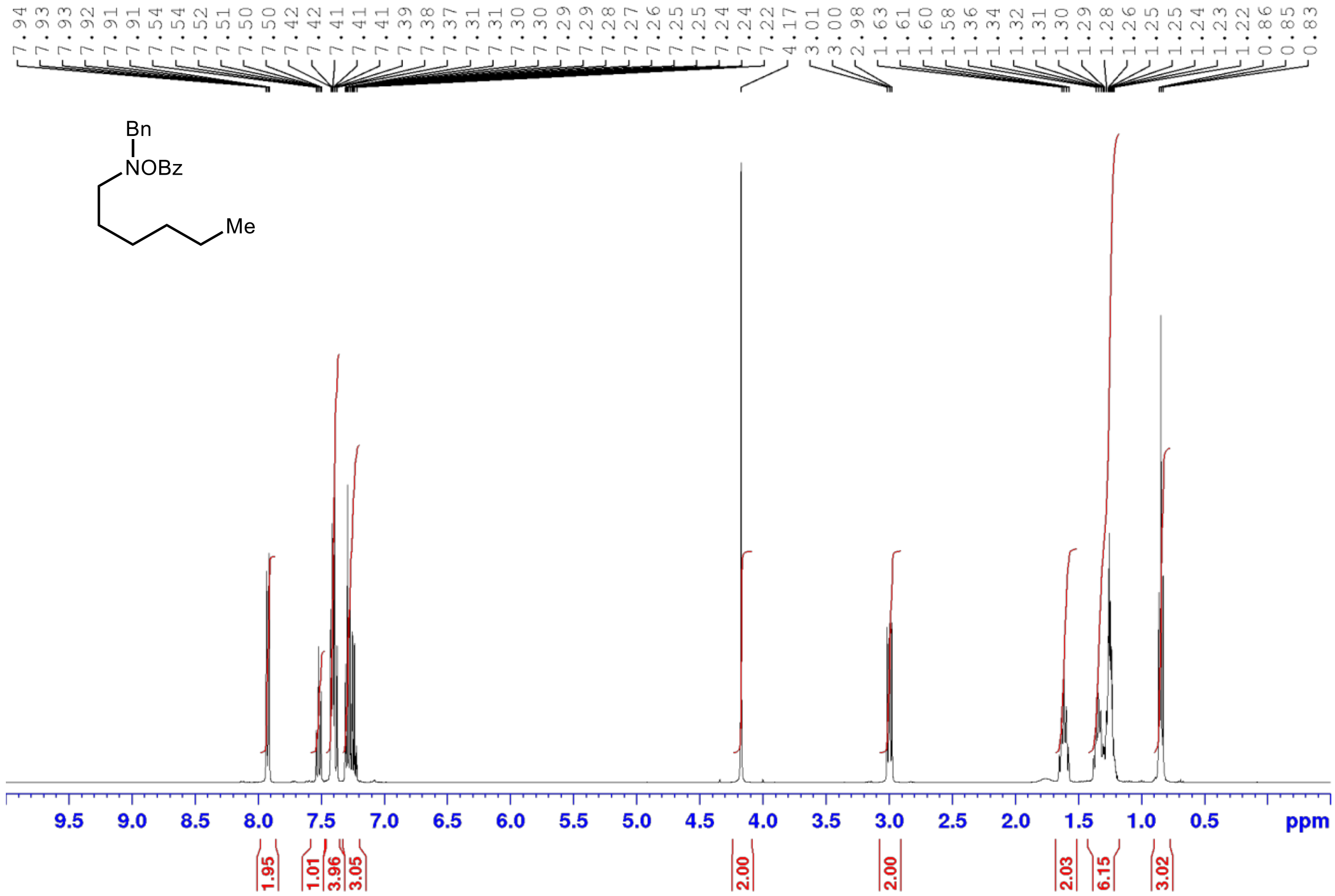

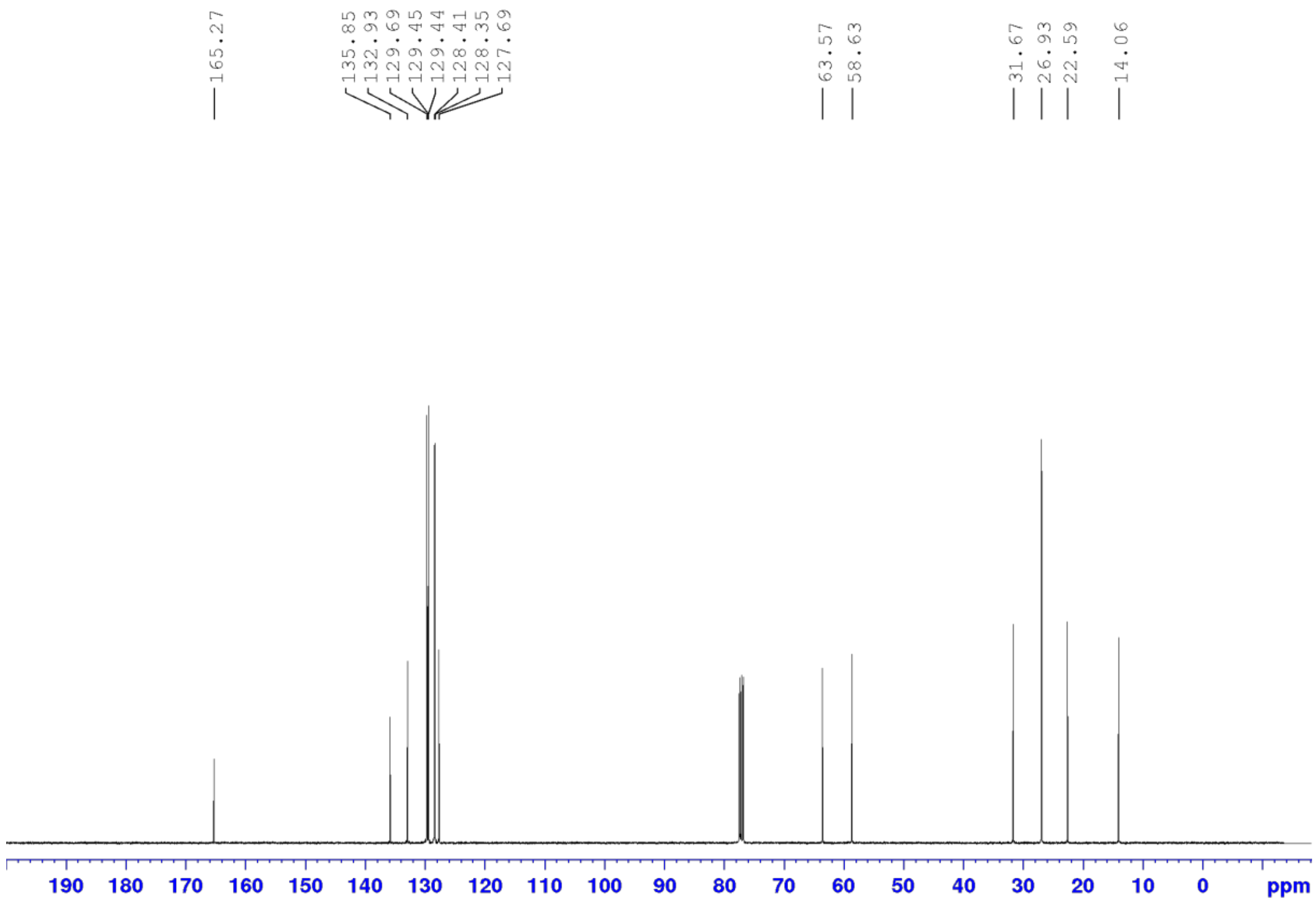

3-((benzoyloxy)(pentyl)amino)propanenitrile (S22)

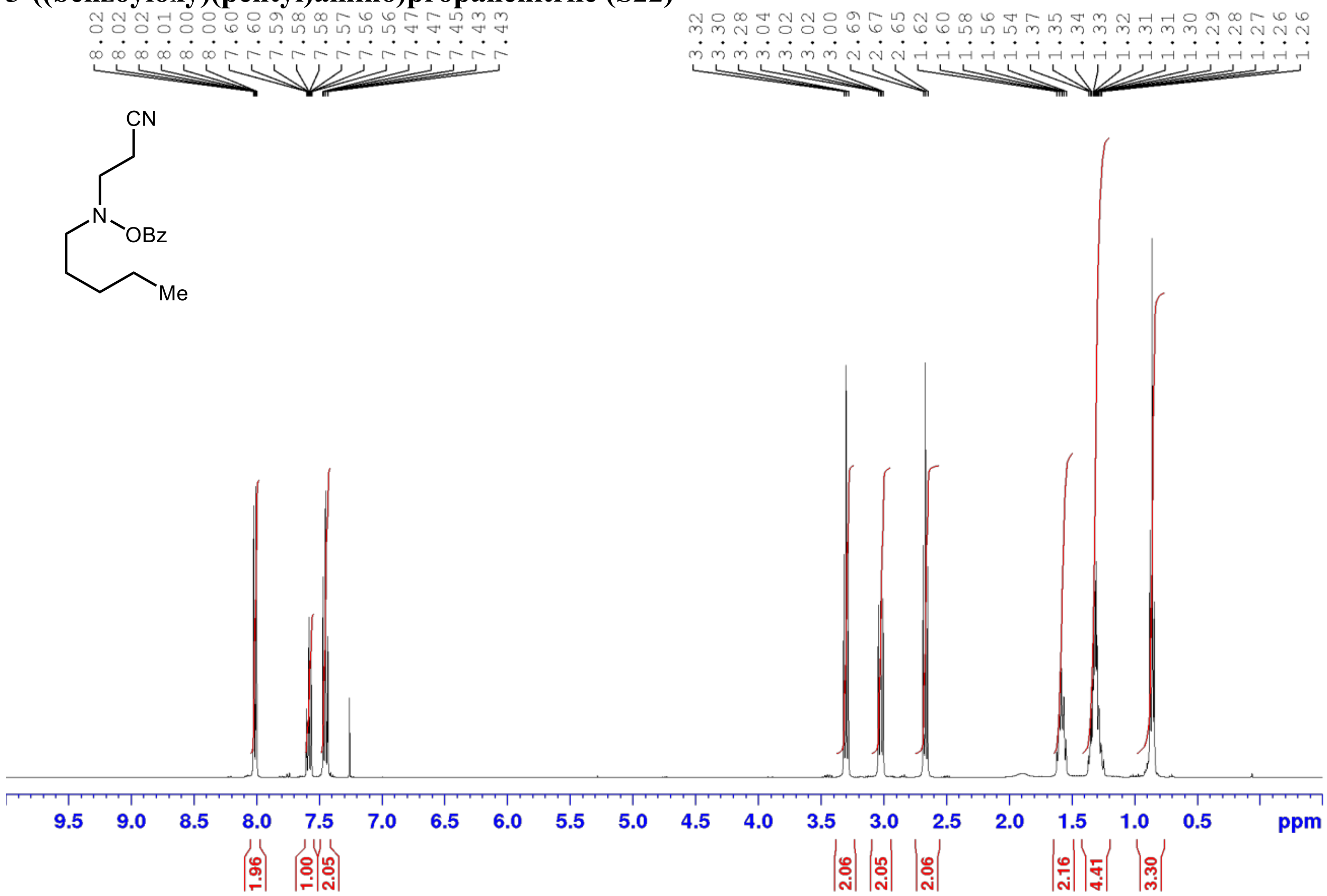

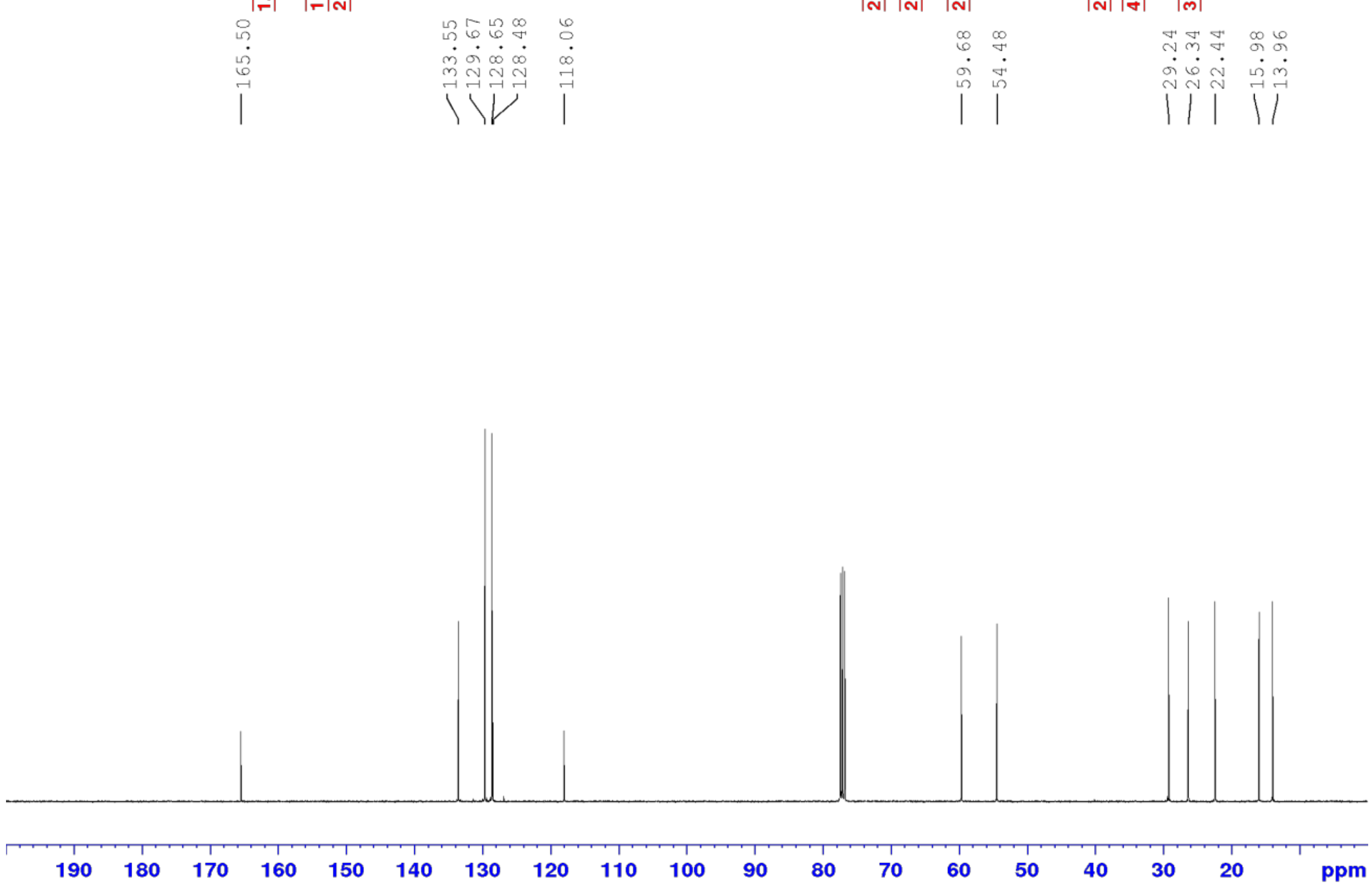

3-((benzoyloxy)(hexyl)amino)propan-1-ol (S23)

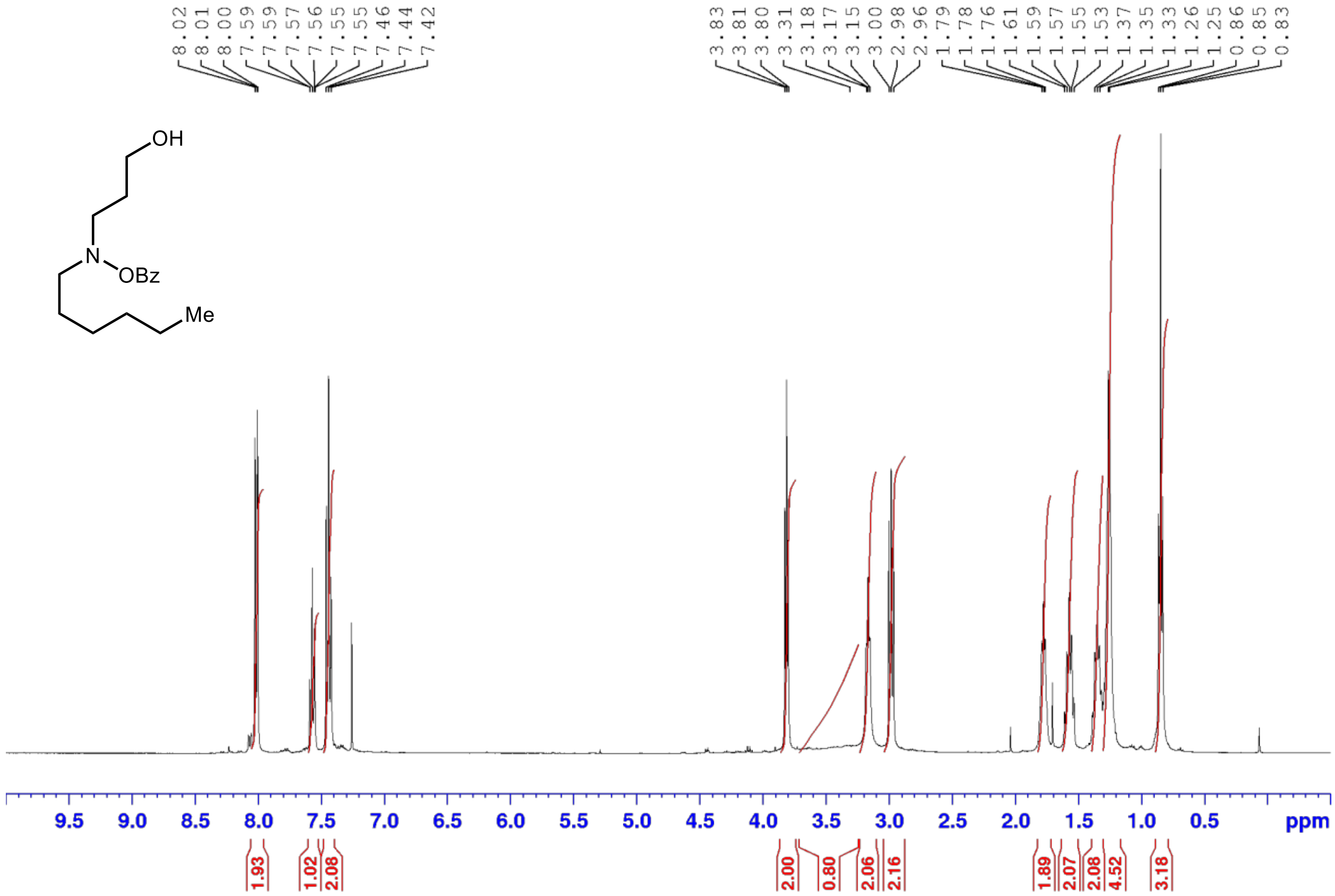

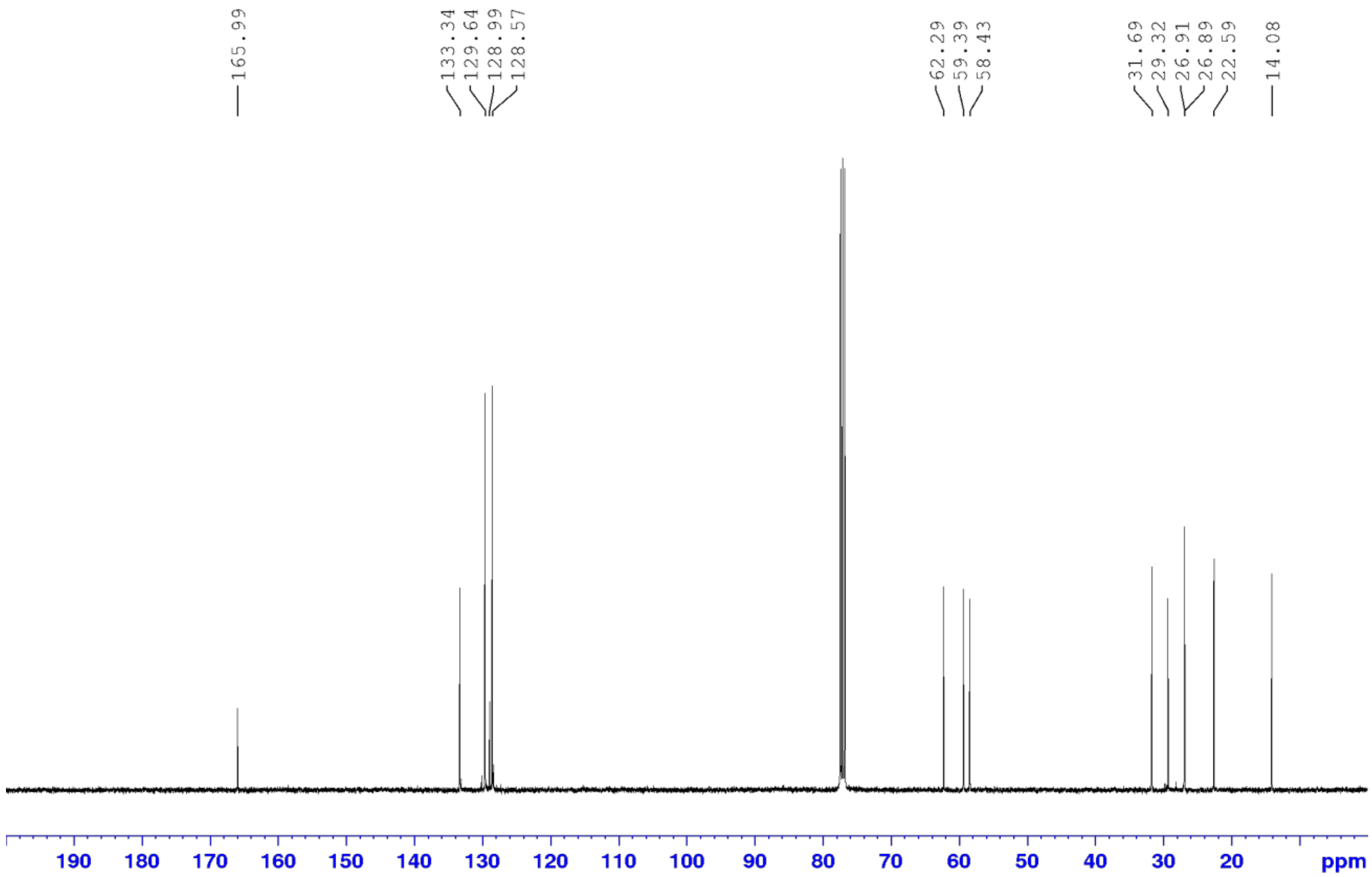

3-((benzoyloxy)(hexyl)amino)propyl benzoate (S24)

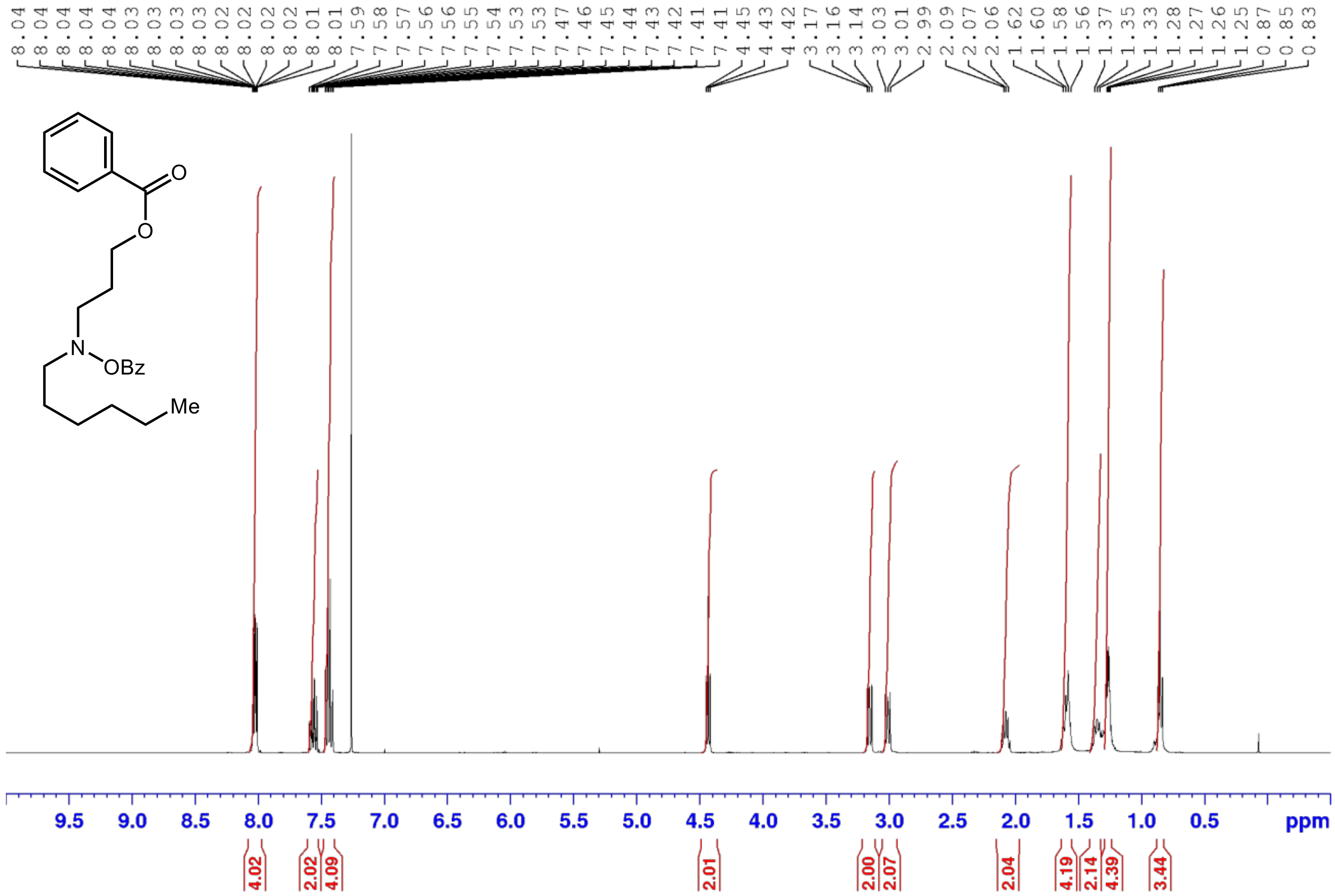

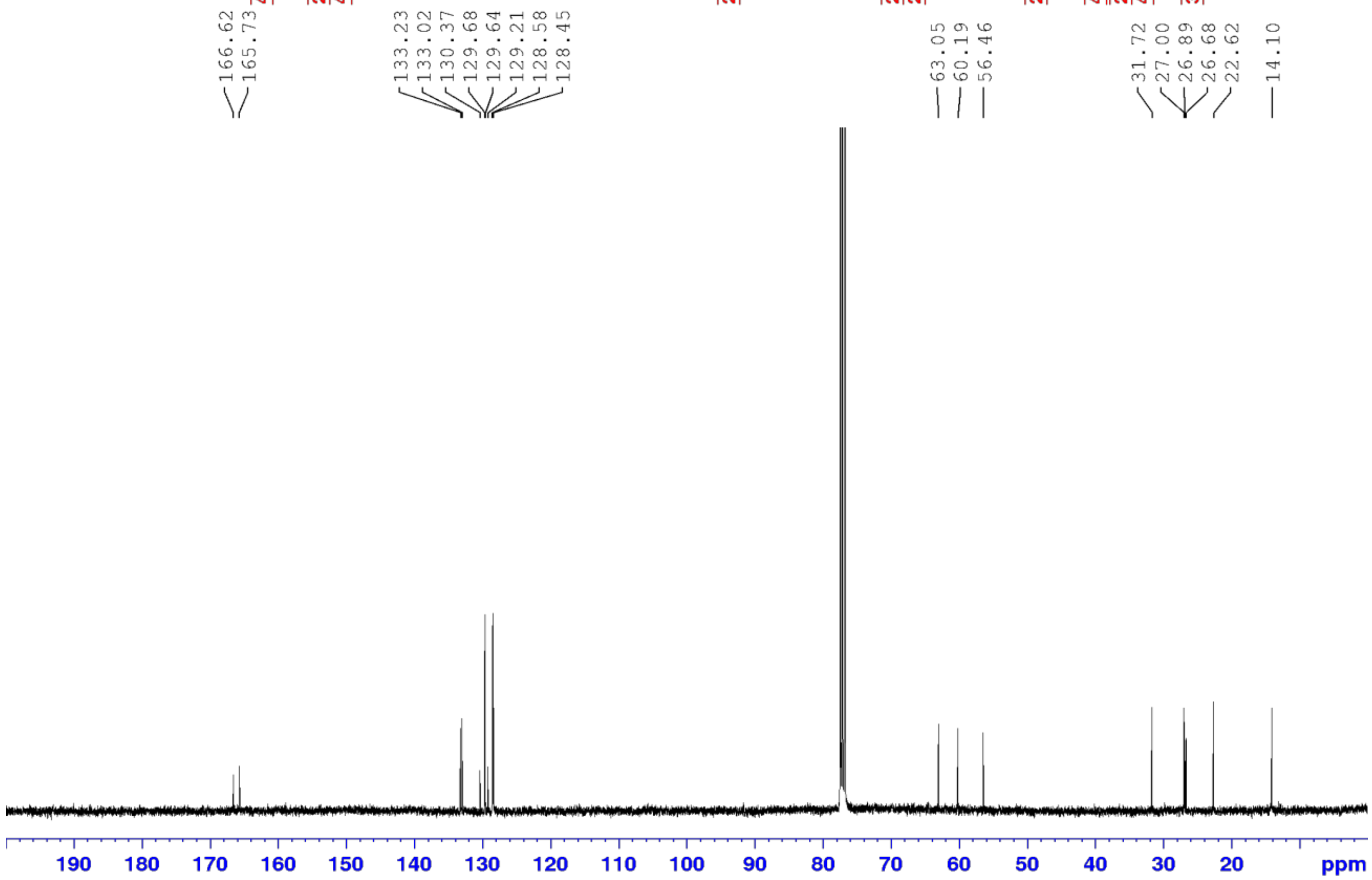

3-((benzoyloxy)(hexyl)amino)propyl 4-(trifluoromethyl)benzoate (S25)

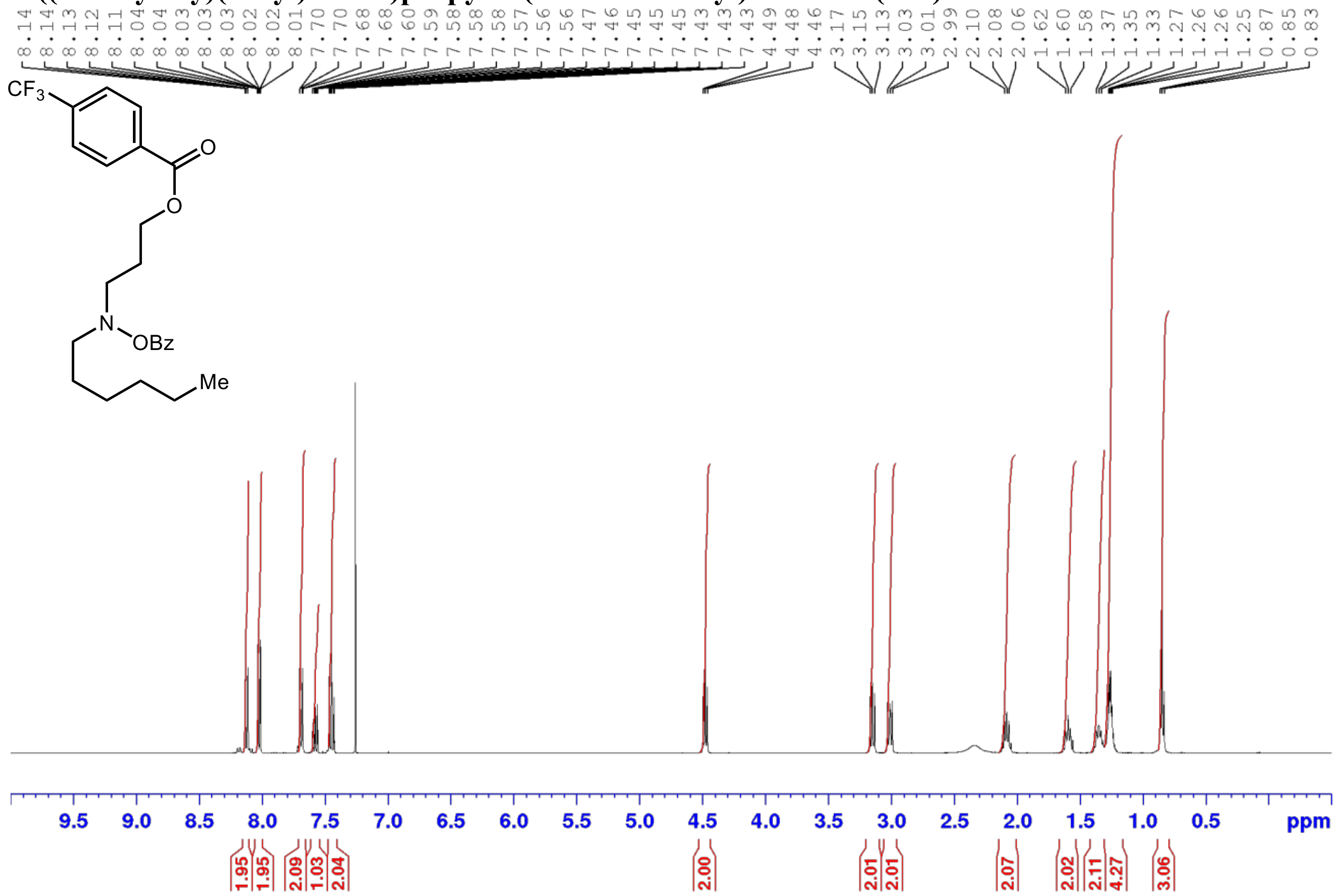

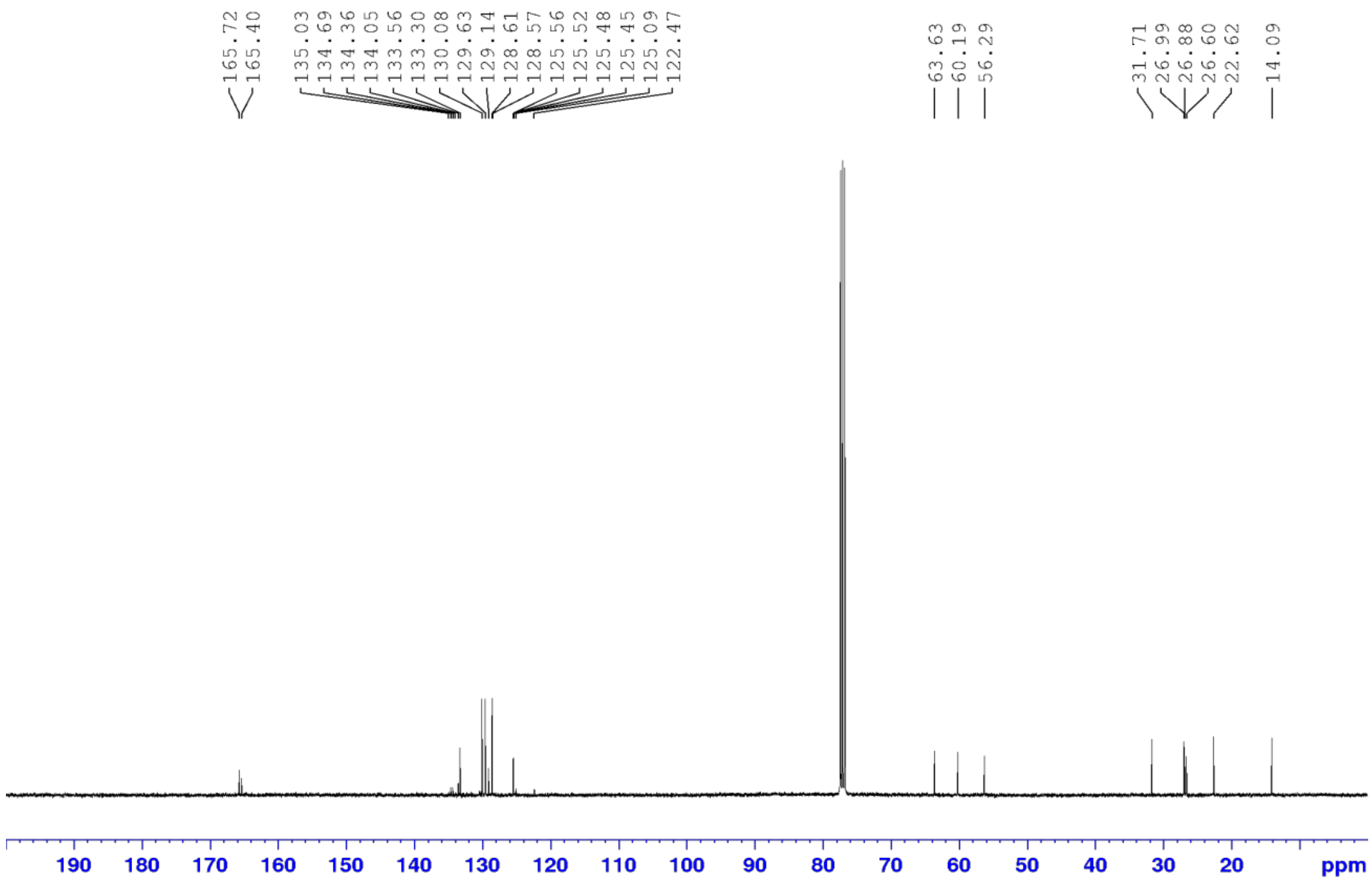

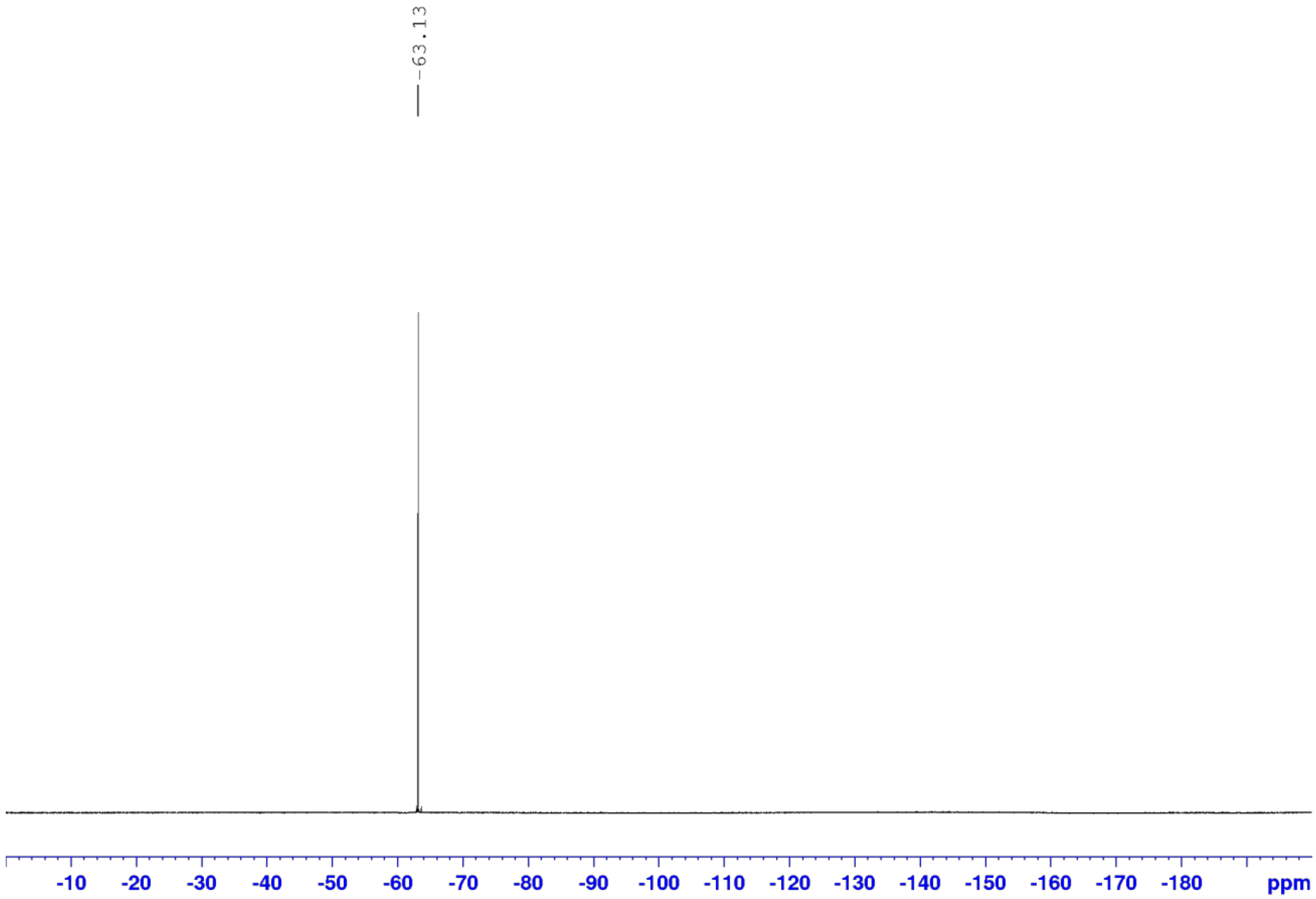

-63.13
-10
-20
-30
-40
-50
-60
-70
-80
-90
-100
-110
-120
-130
-140
-150
-160
-170
-180
ppm

3-((benzoyloxy)(hexyl)amino)propyl 2-(4-methoxyphenyl)acetate (S26)

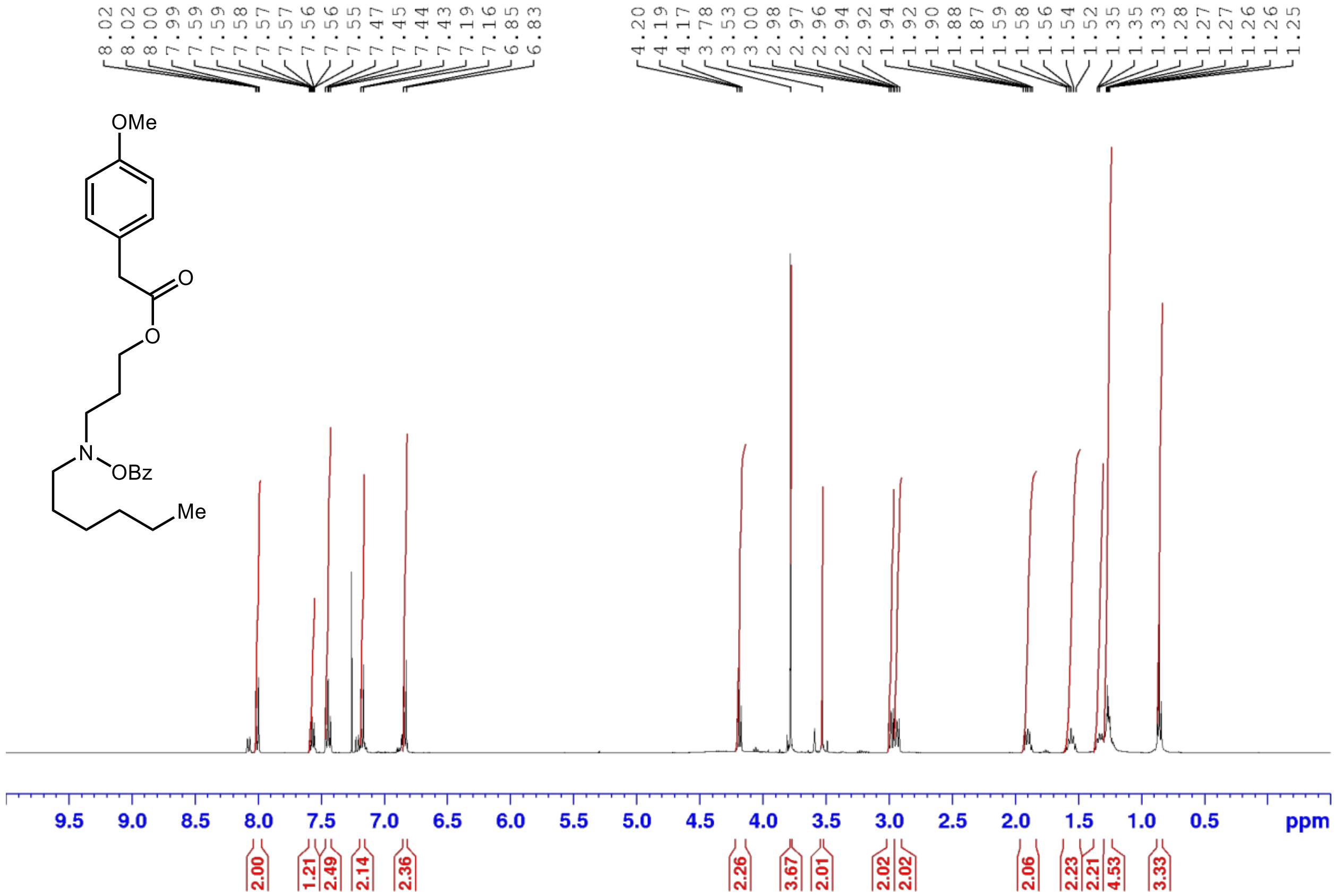

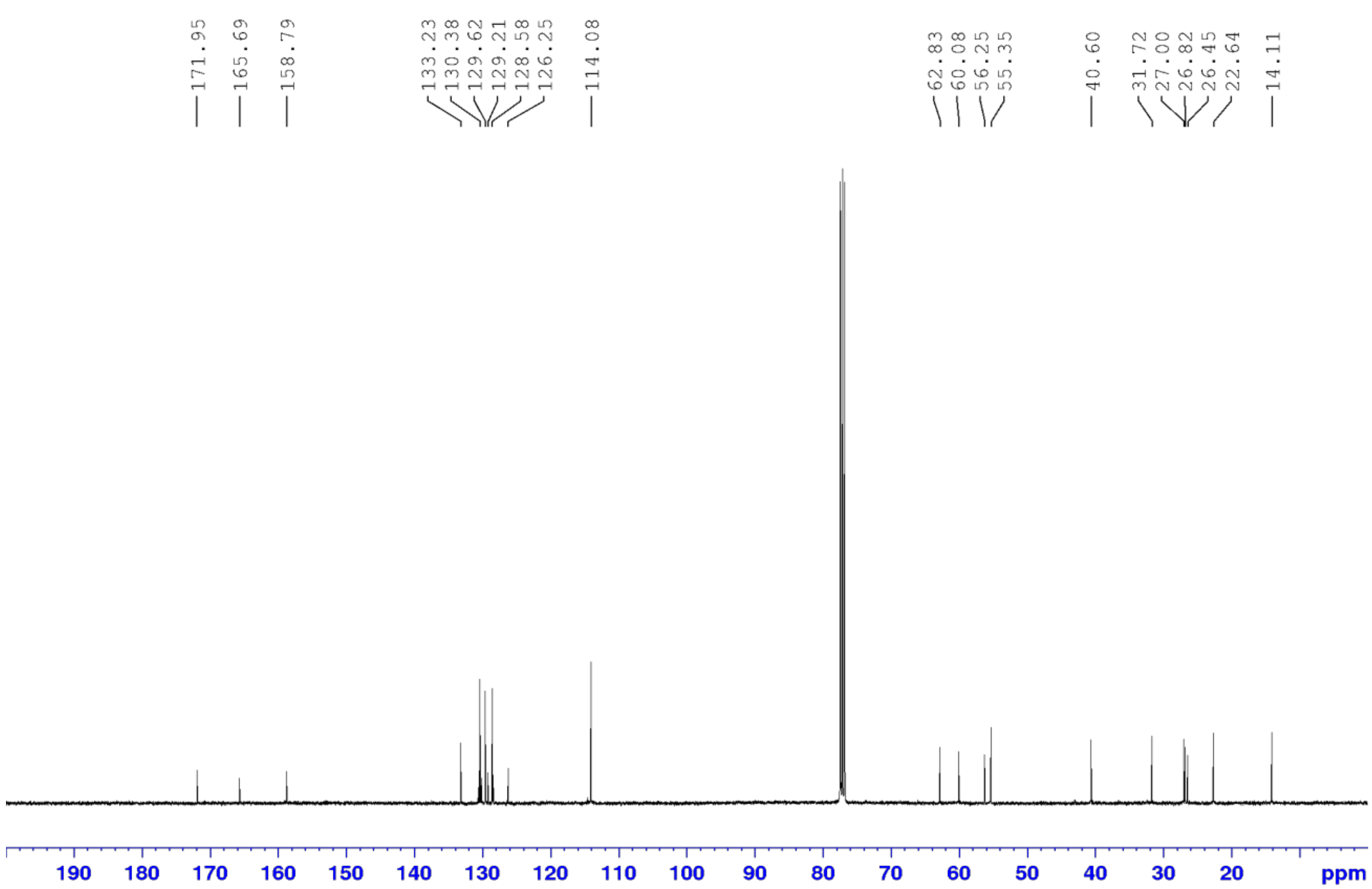

3-((benzoyloxy)(hexyl)amino)propyl 4-(5-(2-fluorophenyl)-1,2,4-oxadiazol-3-yl)benzoate (S27)

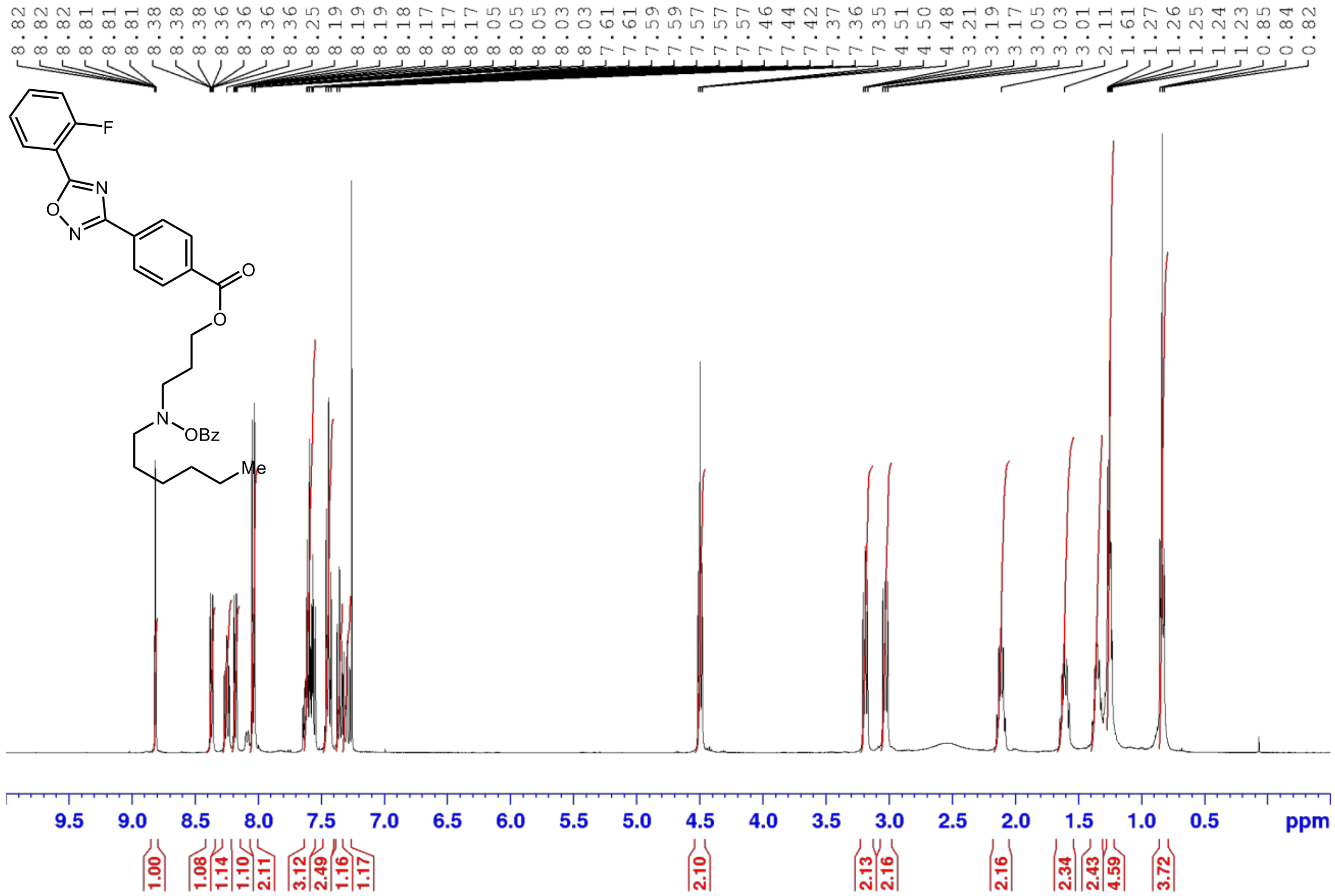

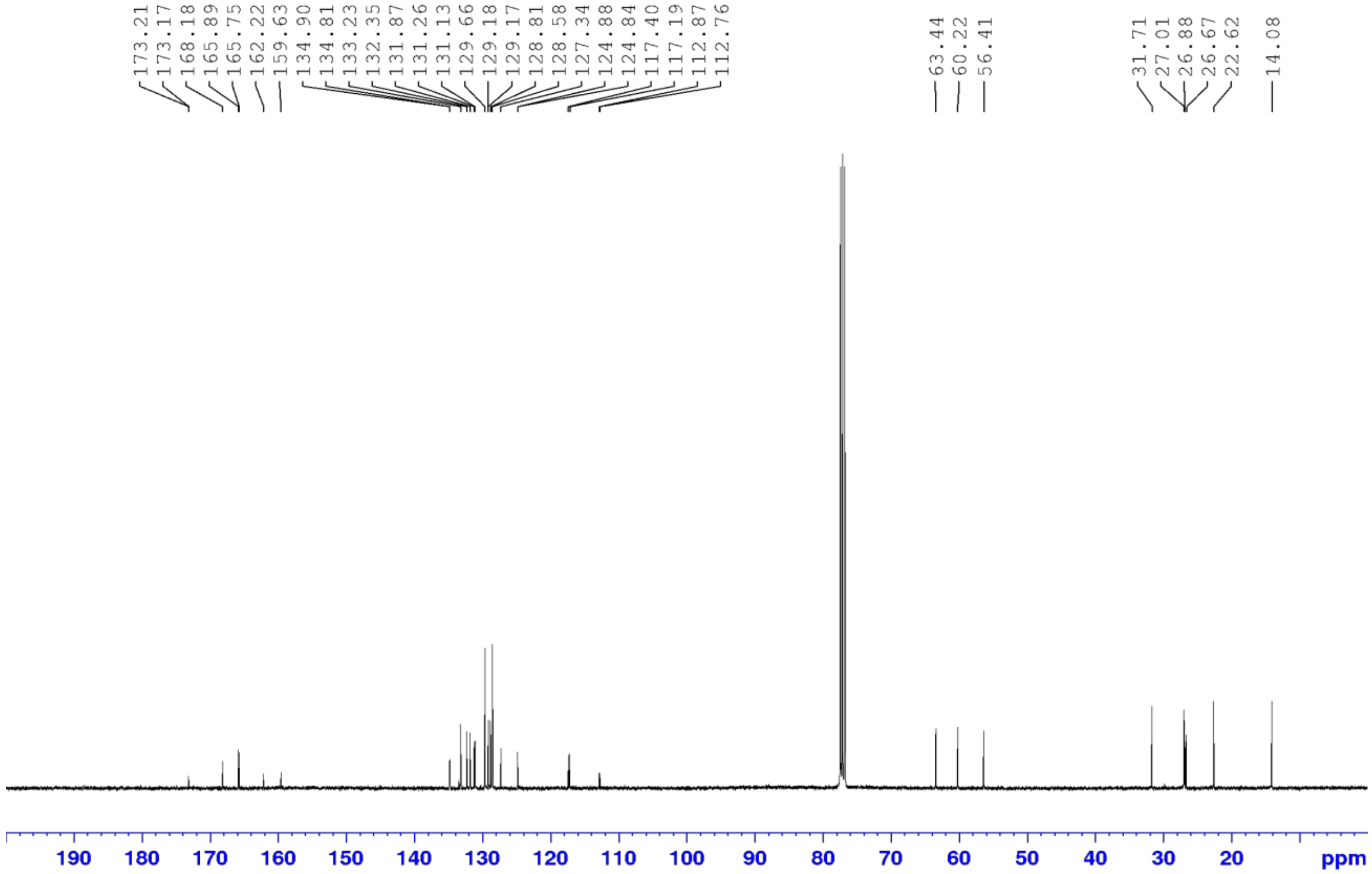

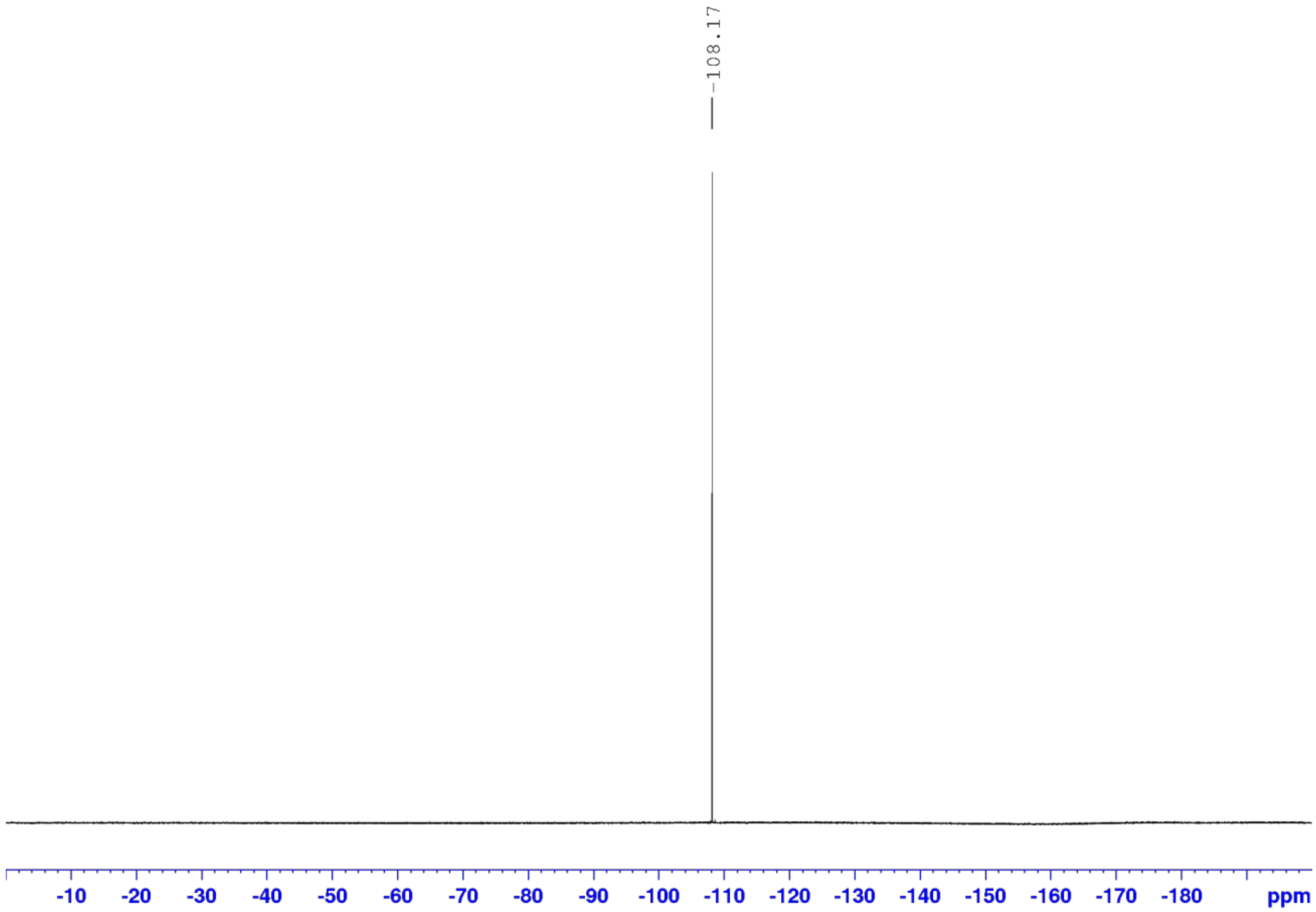
-108.17
-10
-20
-30
-40
-50
-60
-70
-80
-90
-100
-110
-120
-130
-140
-150
-160
-170
-180
ppm

(E)-N-hexylhex-4-en-1-aminium (1)

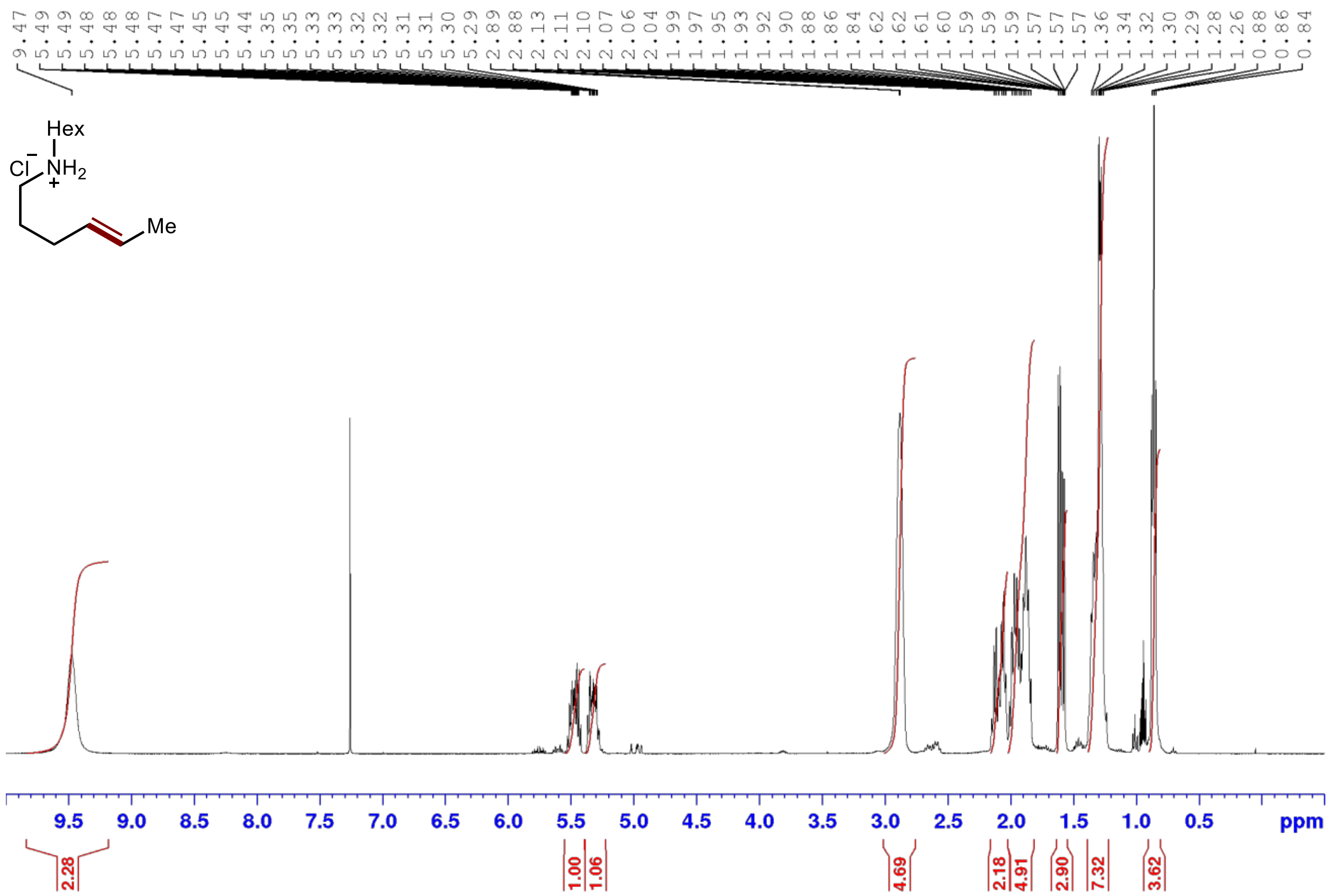

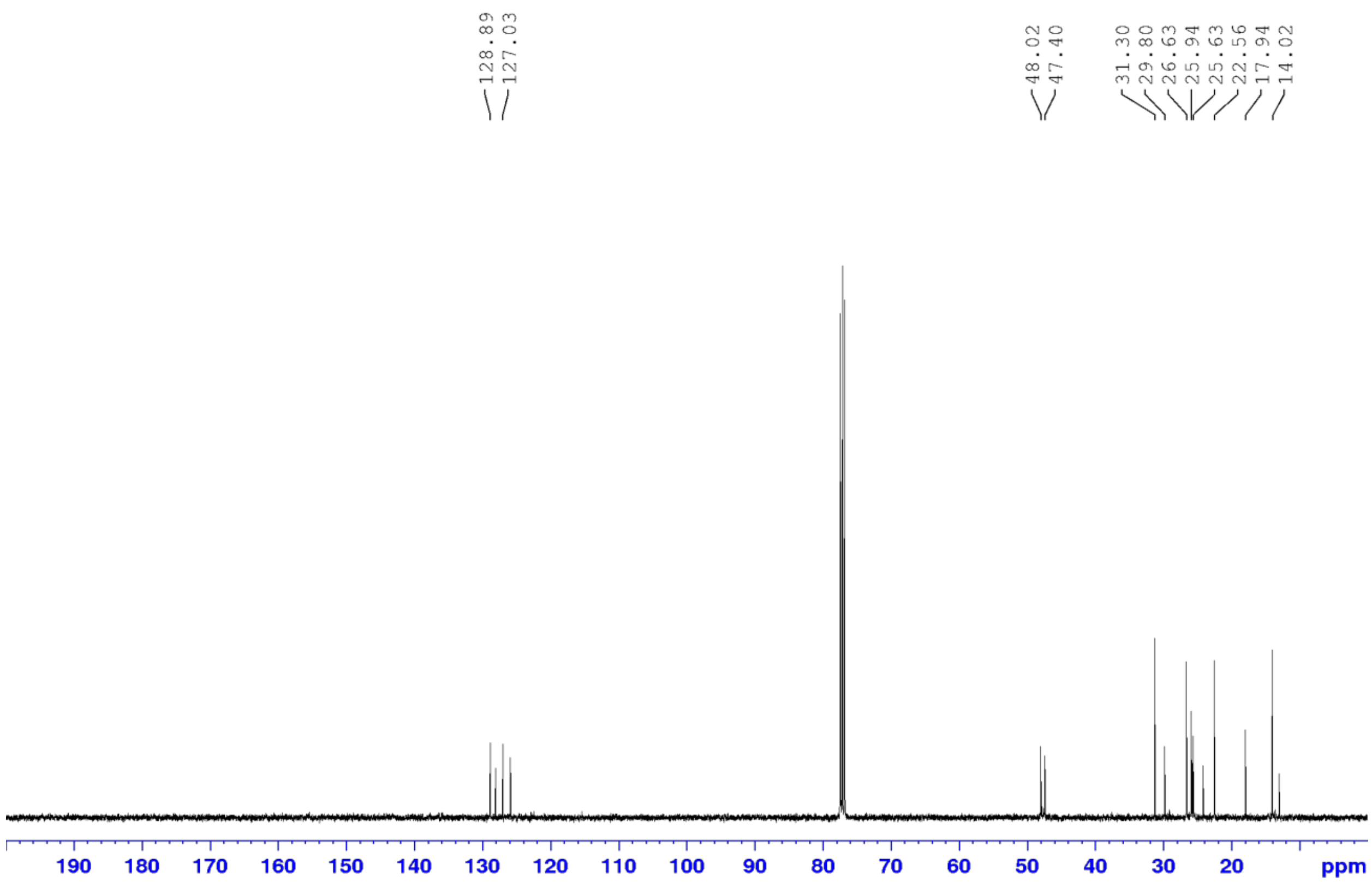

(E)-N-propylhex-4-en-1-aminium (2)

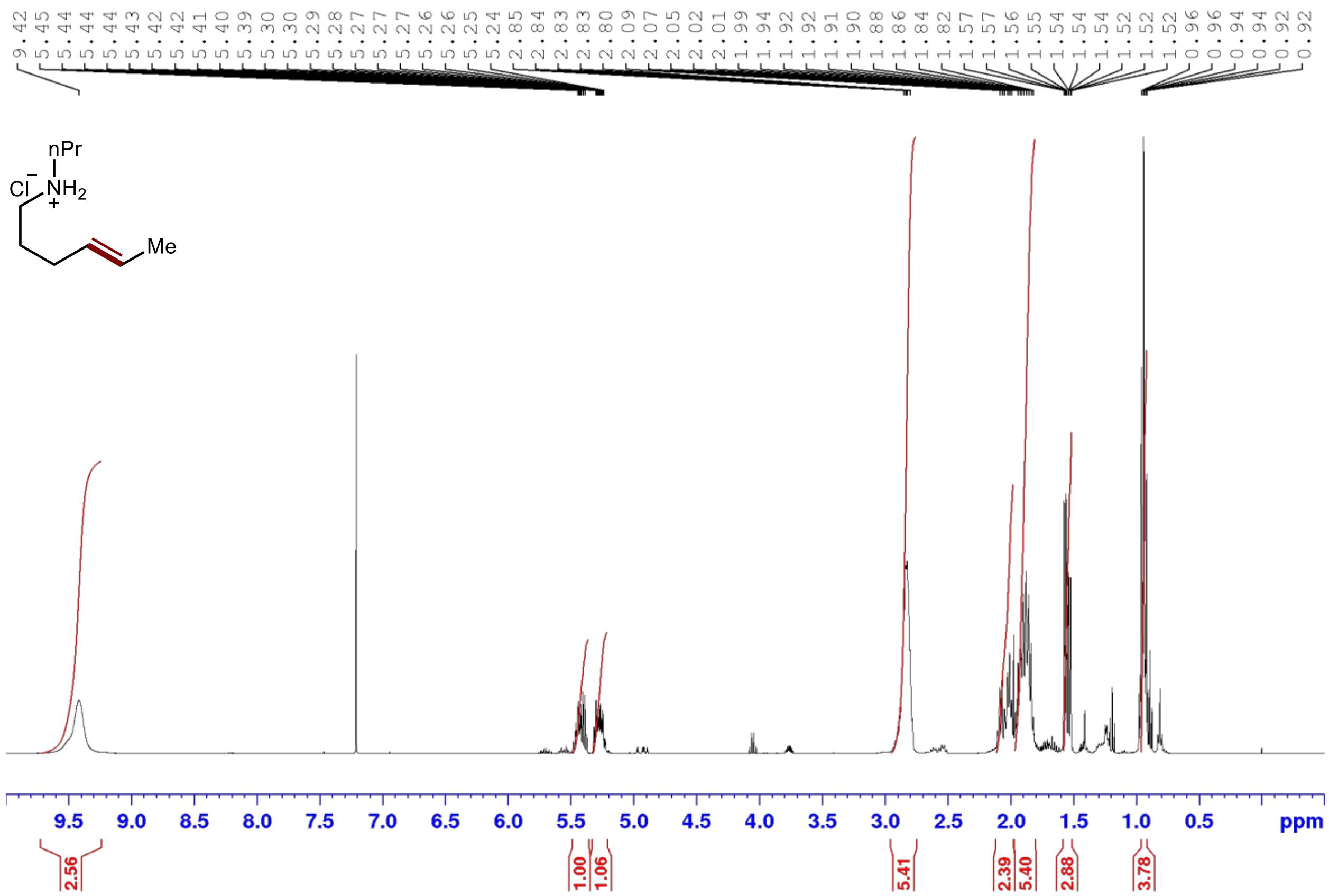

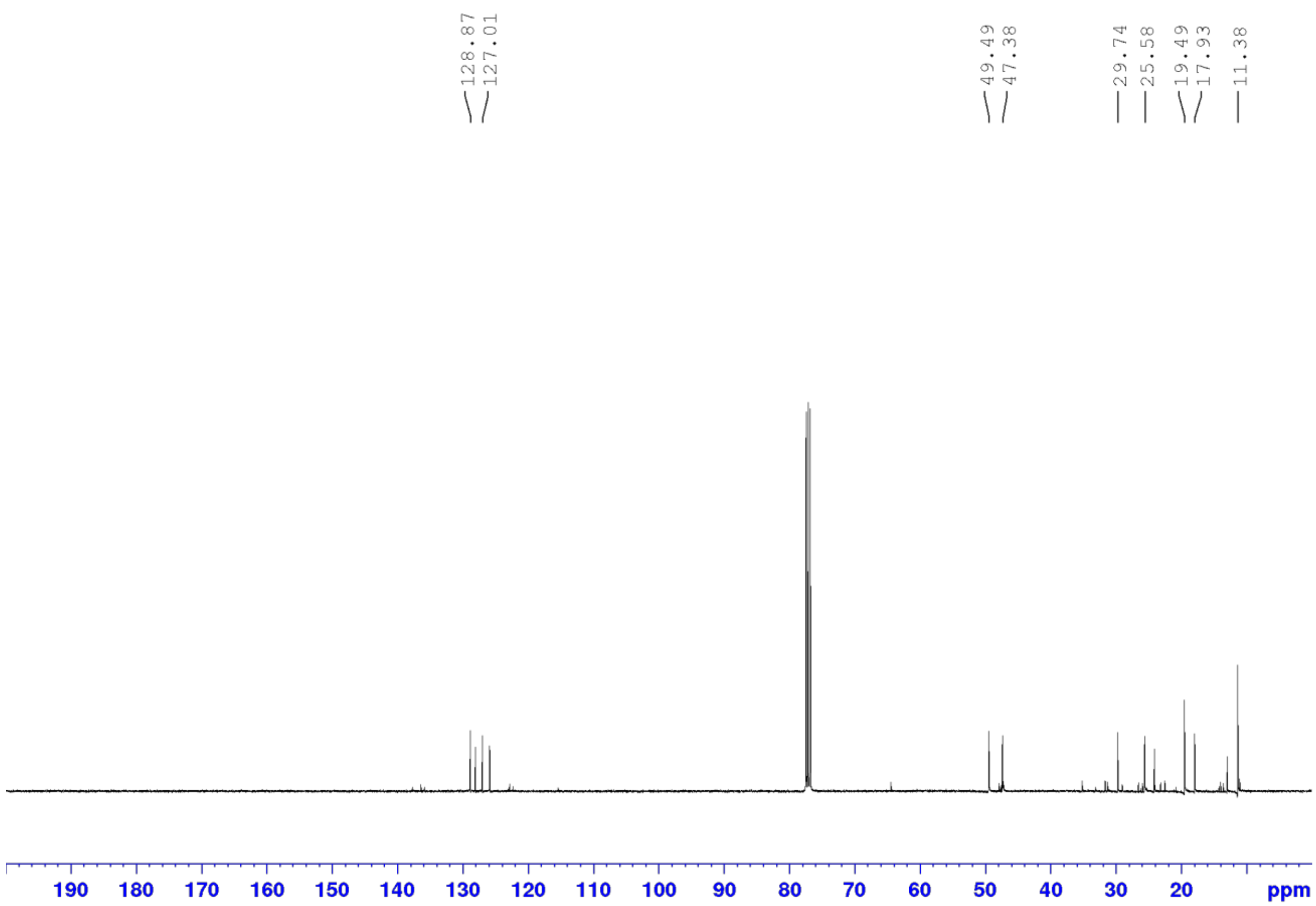

(E)-N-(tert-butyl)hex-4-en-1-aminium (3)

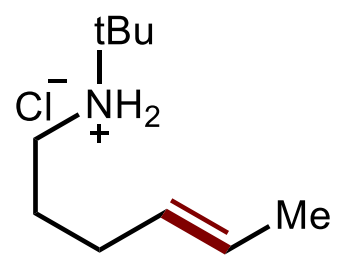

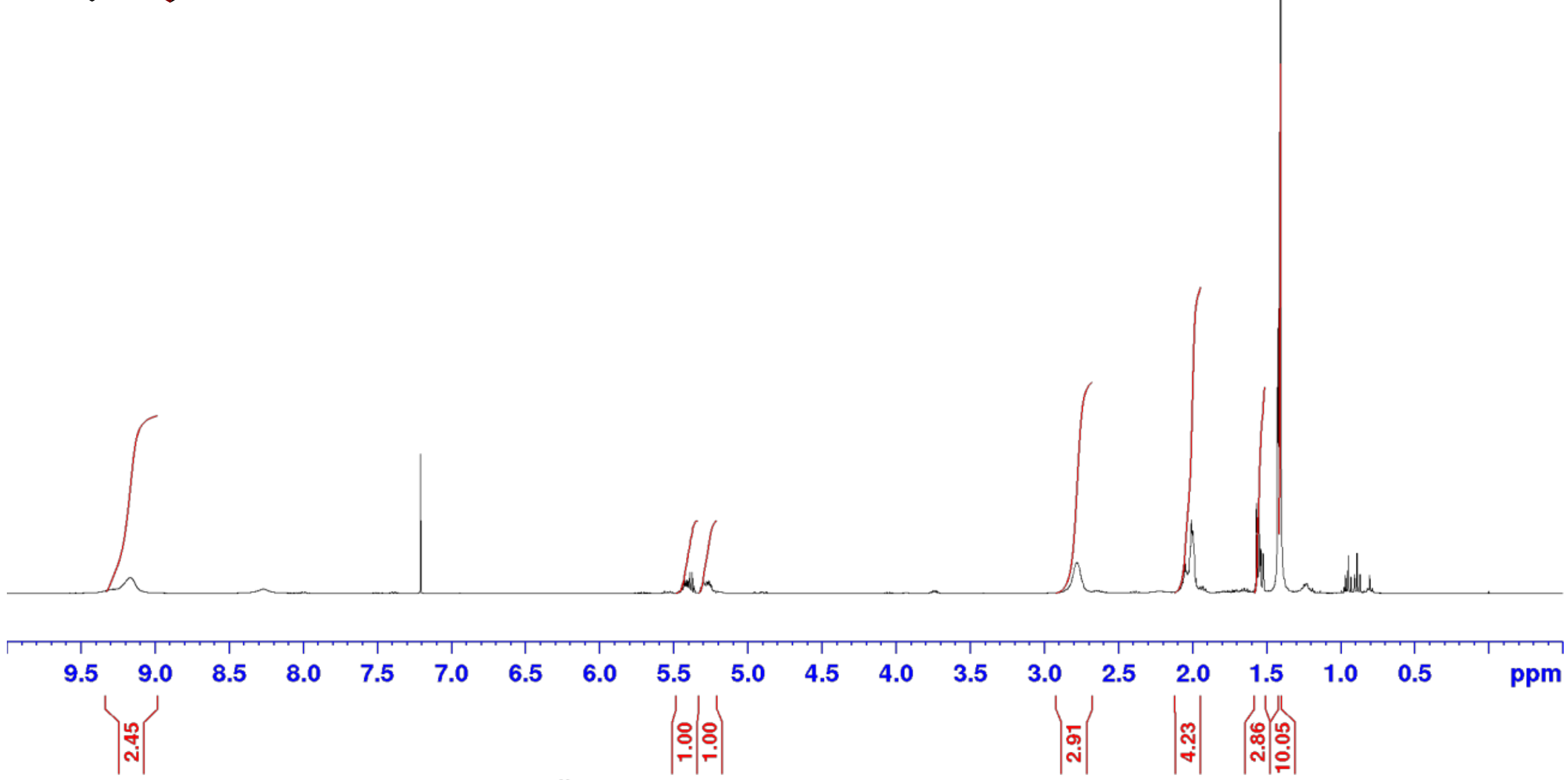

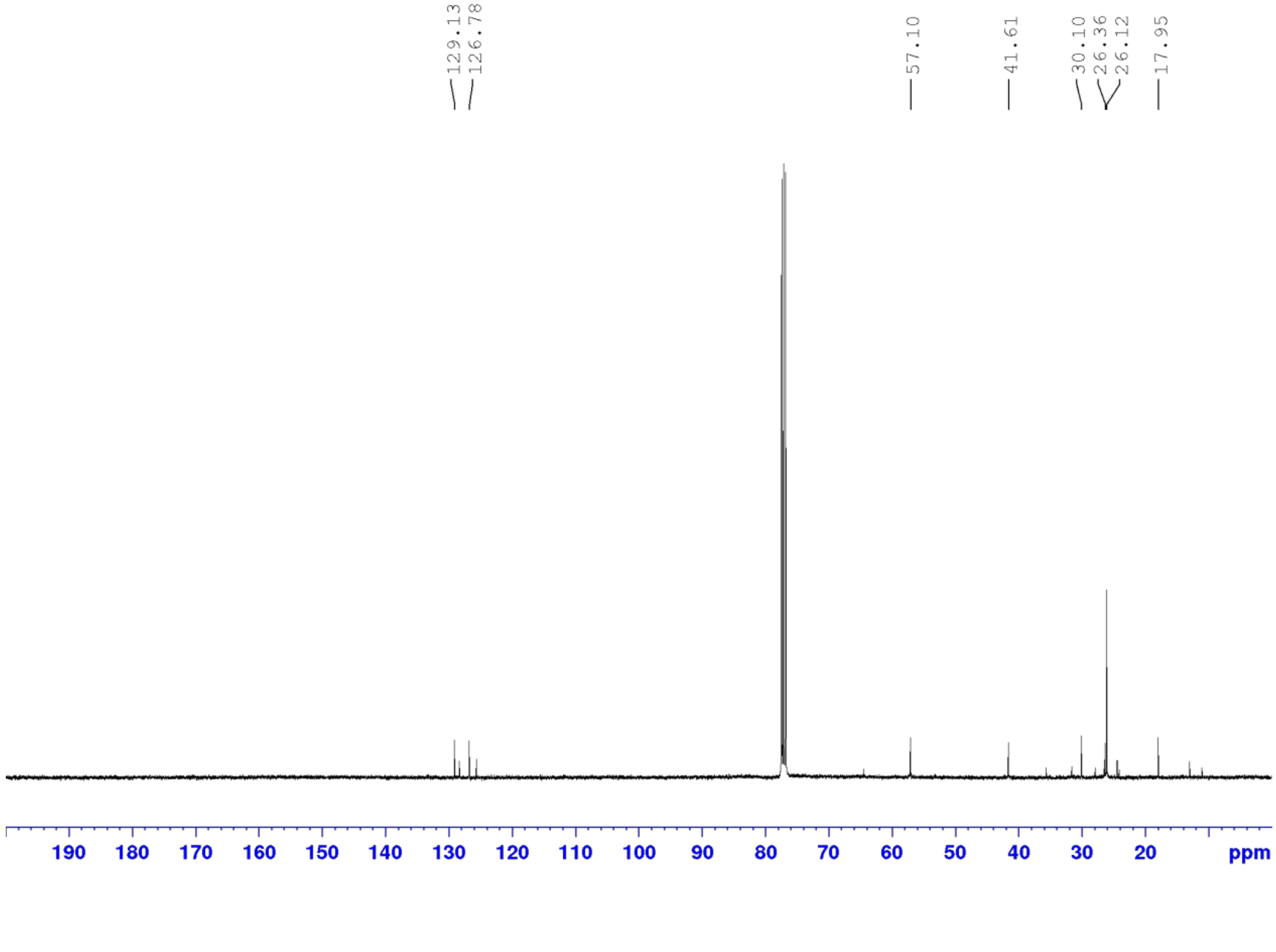

(E)-N-propylhept-5-en-2-aminium (4)

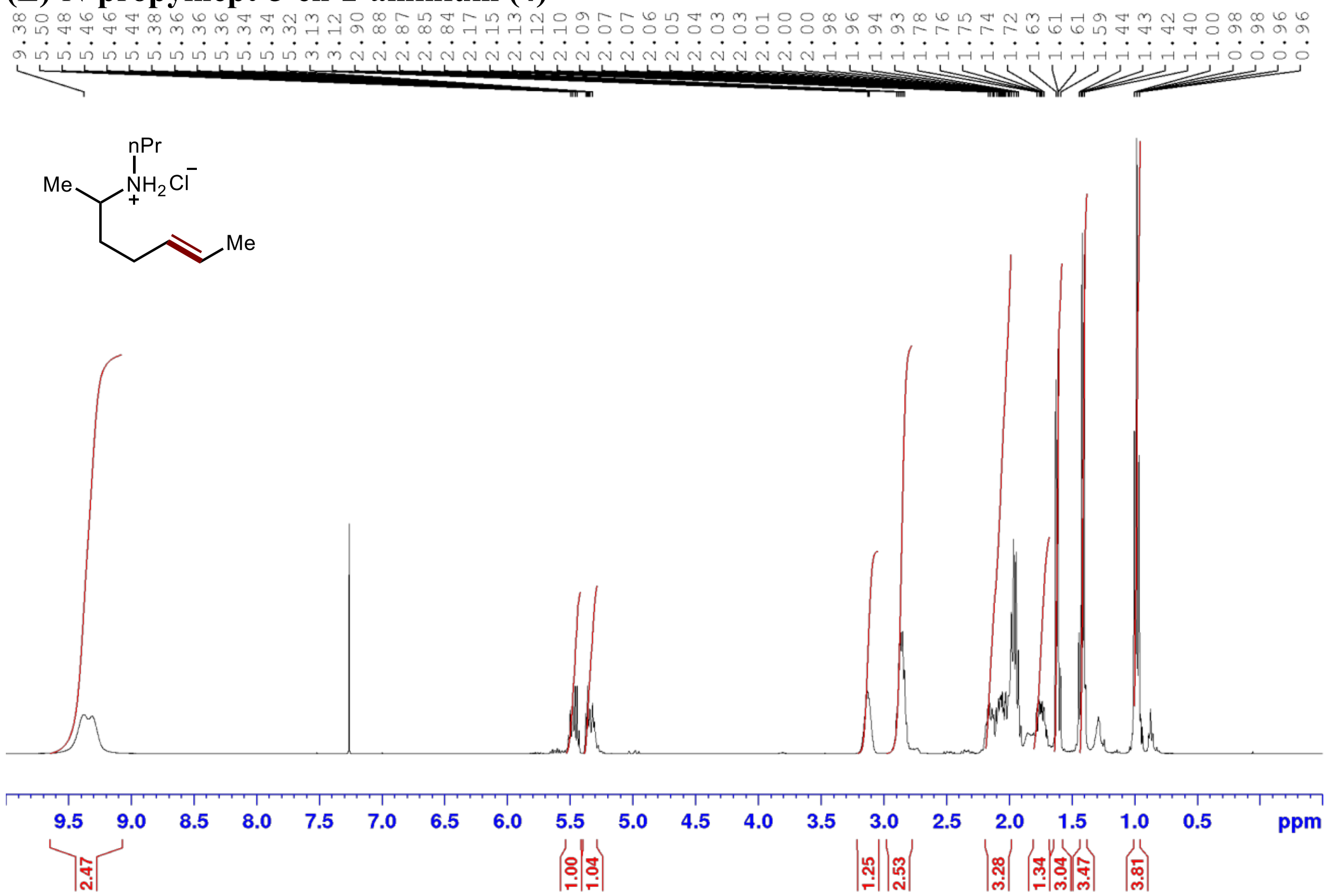

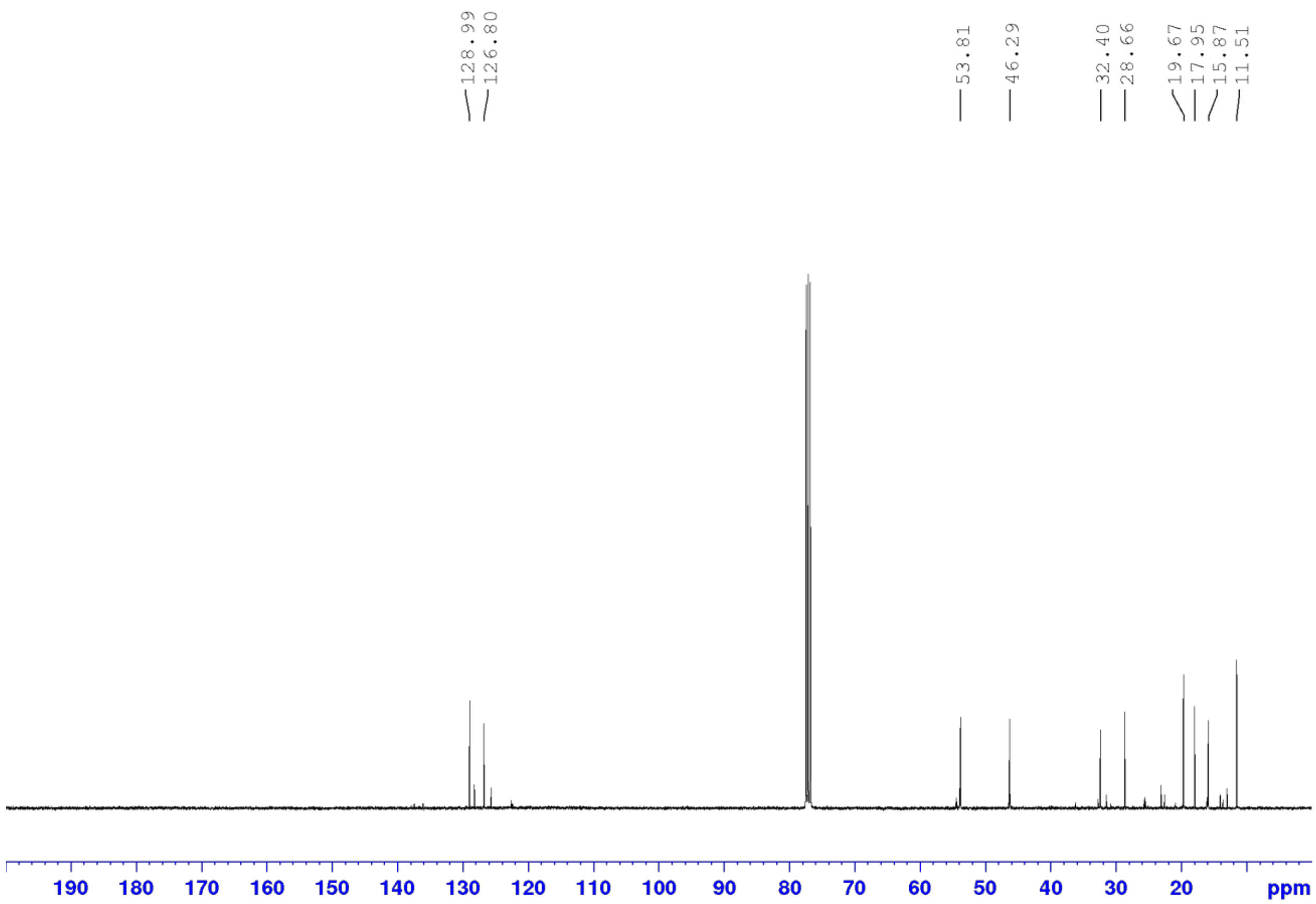

(E)-2-methyl-N-propylhex-4-en-1-aminium (5)

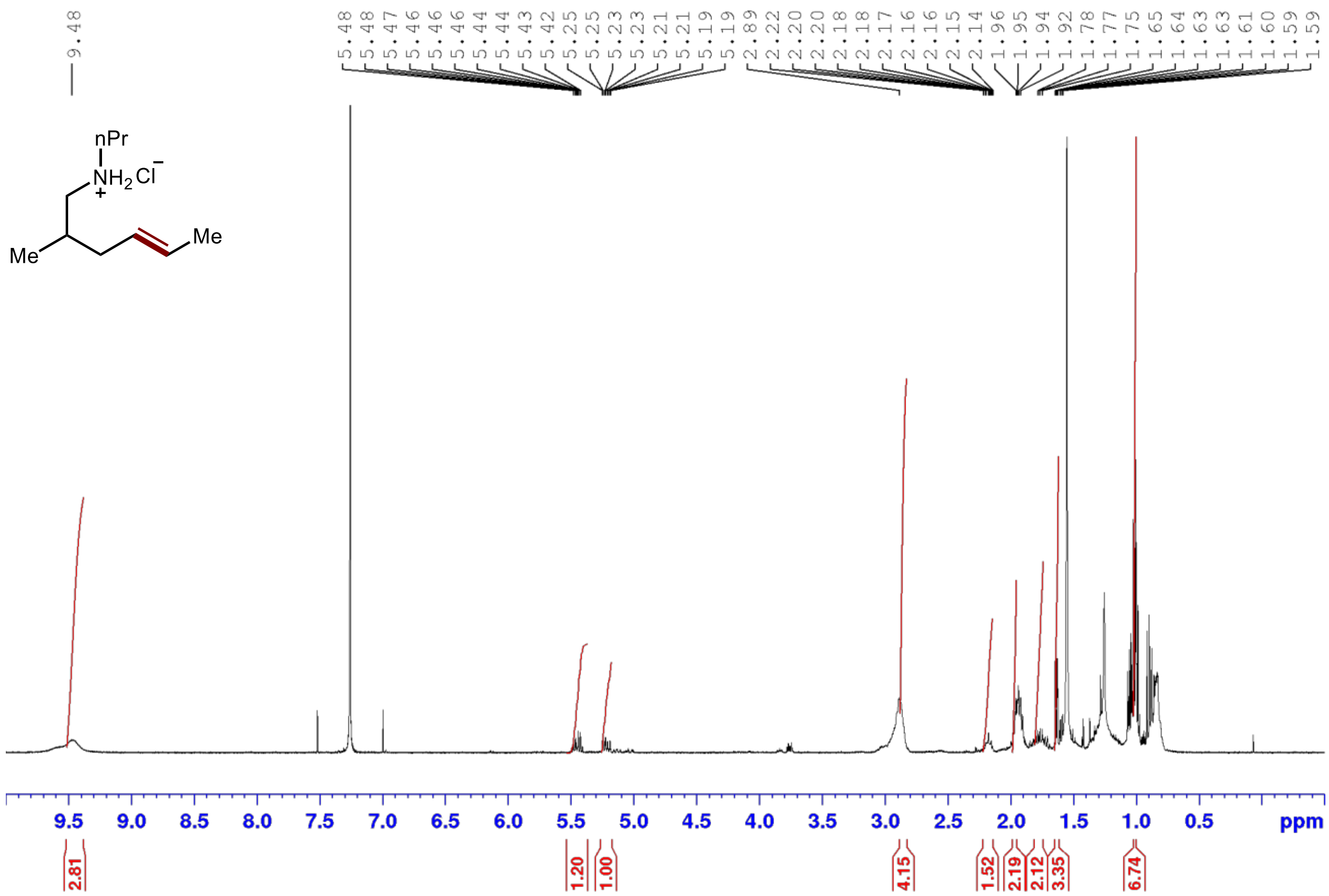

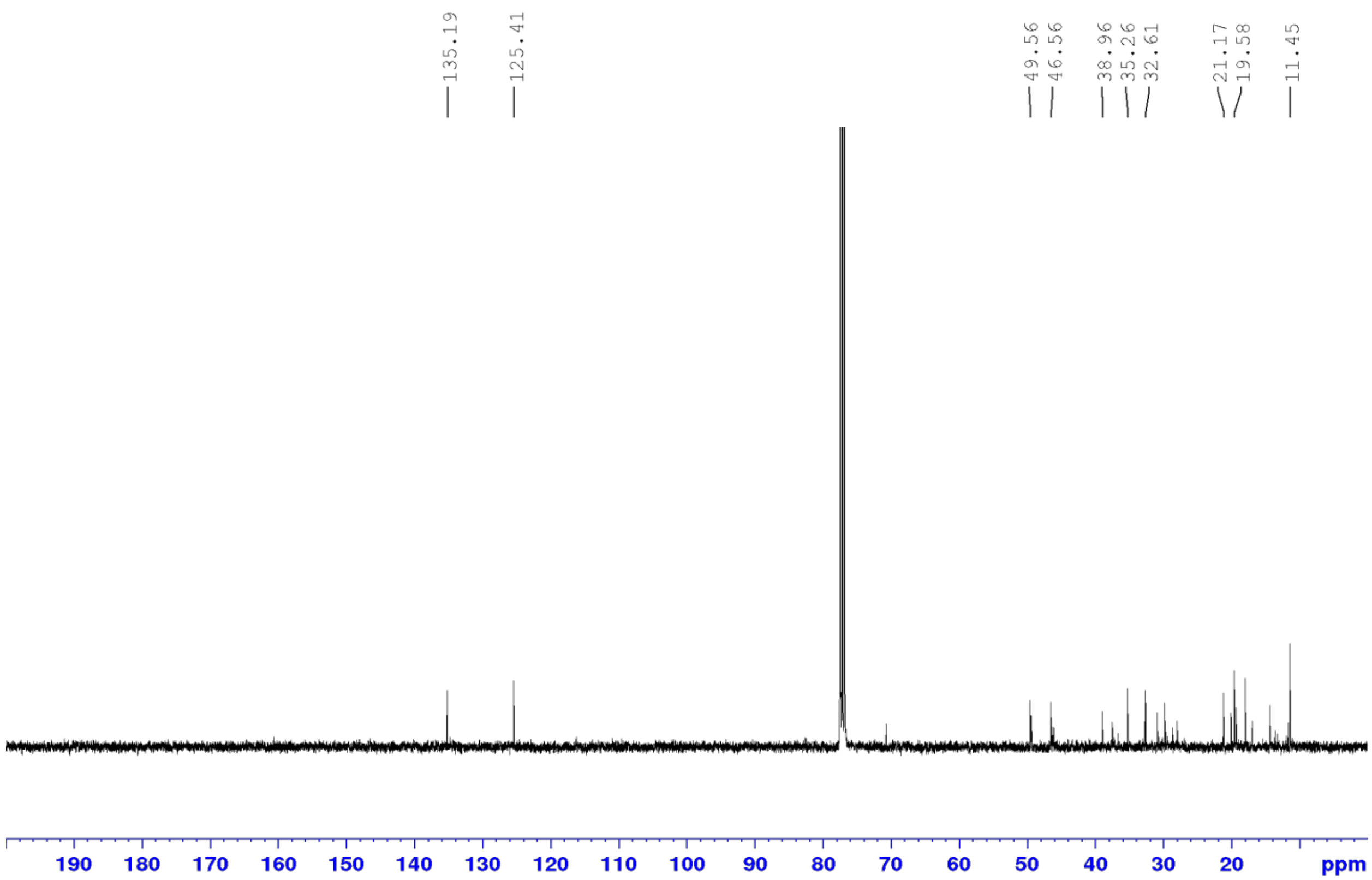

(E)-3-methyl-N-propylhex-4-en-1-aminium (6)

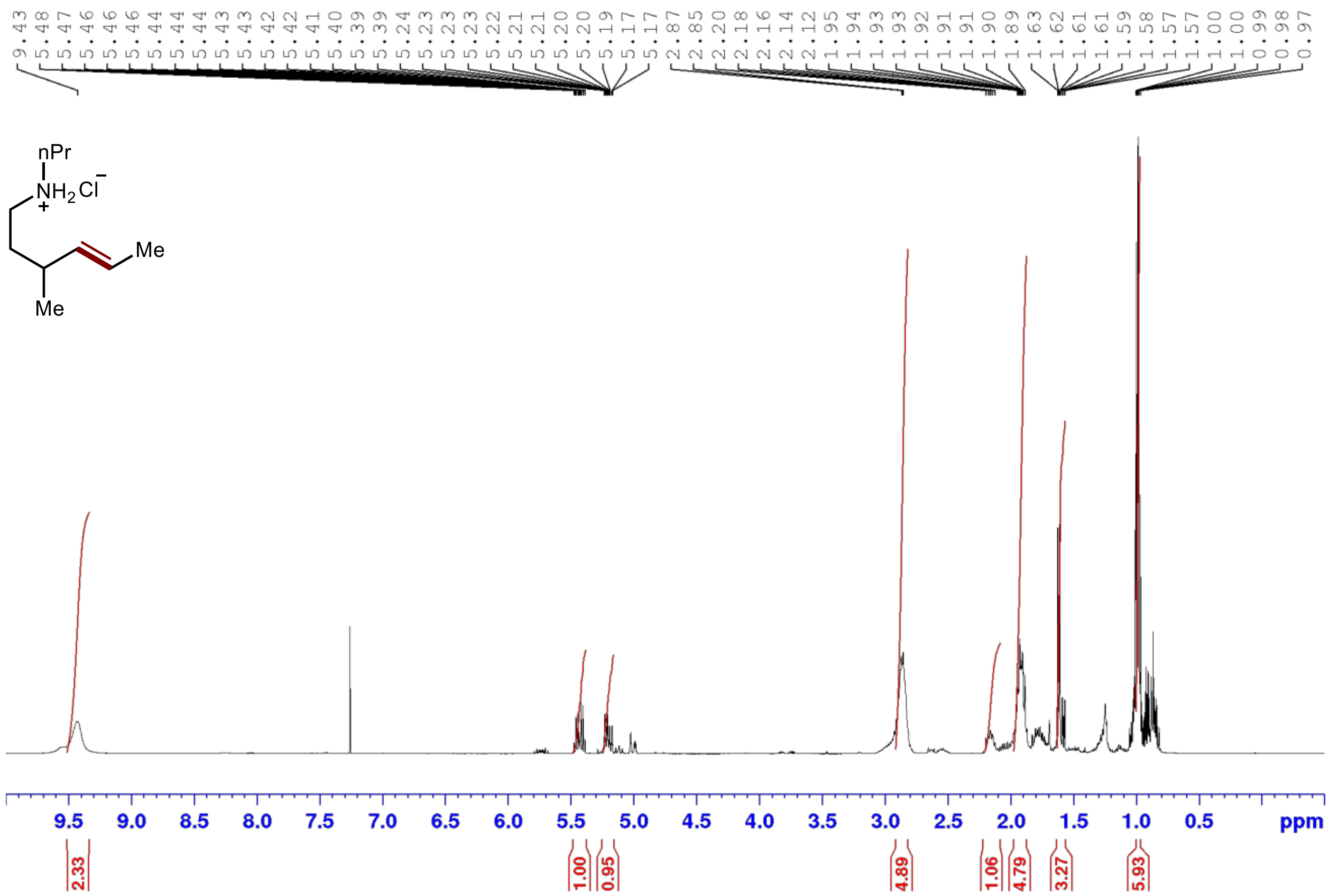

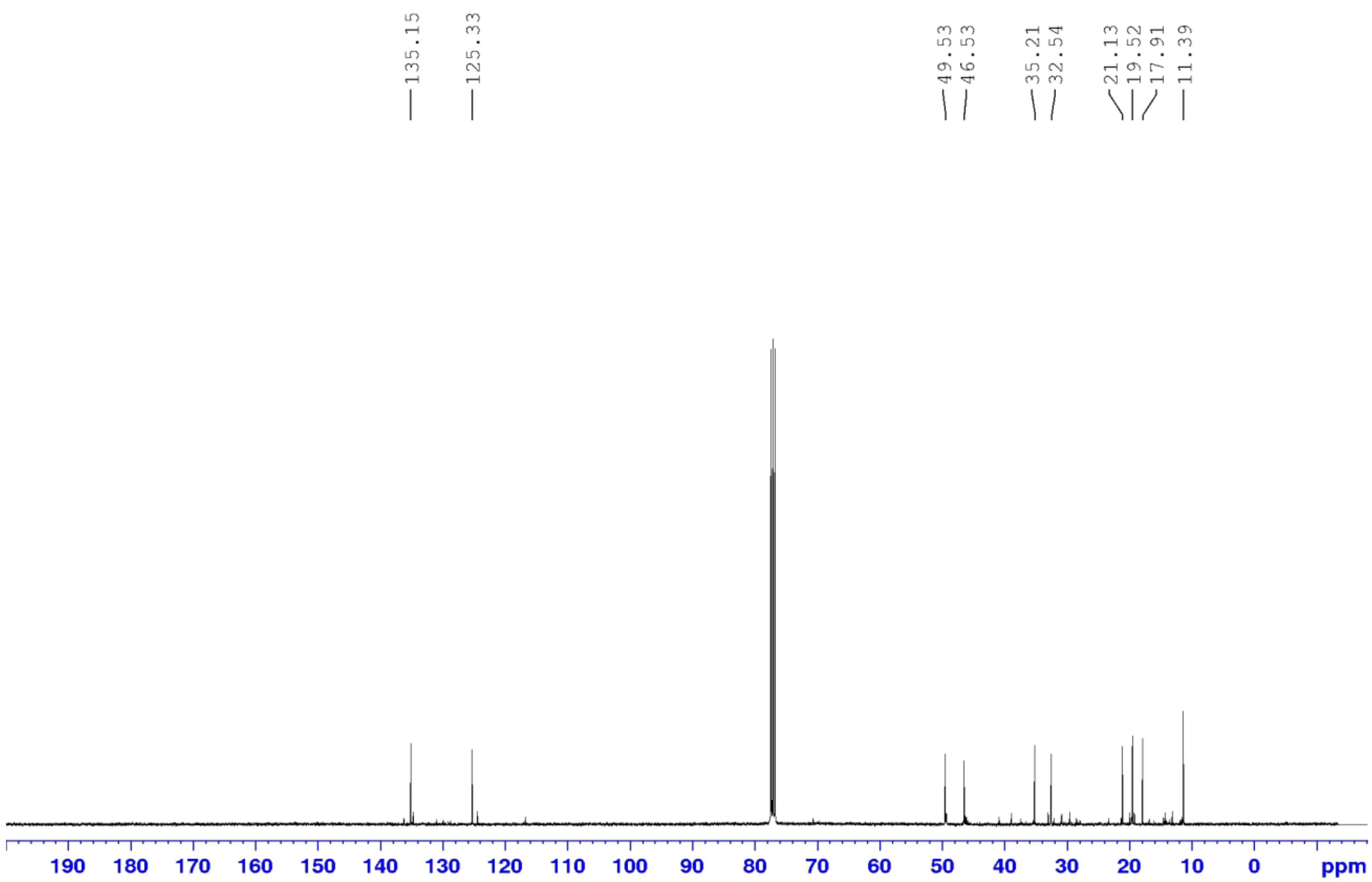

N-(2-(cyclopent-2-en-1-yl)ethyl)propan-1-aminium (7)

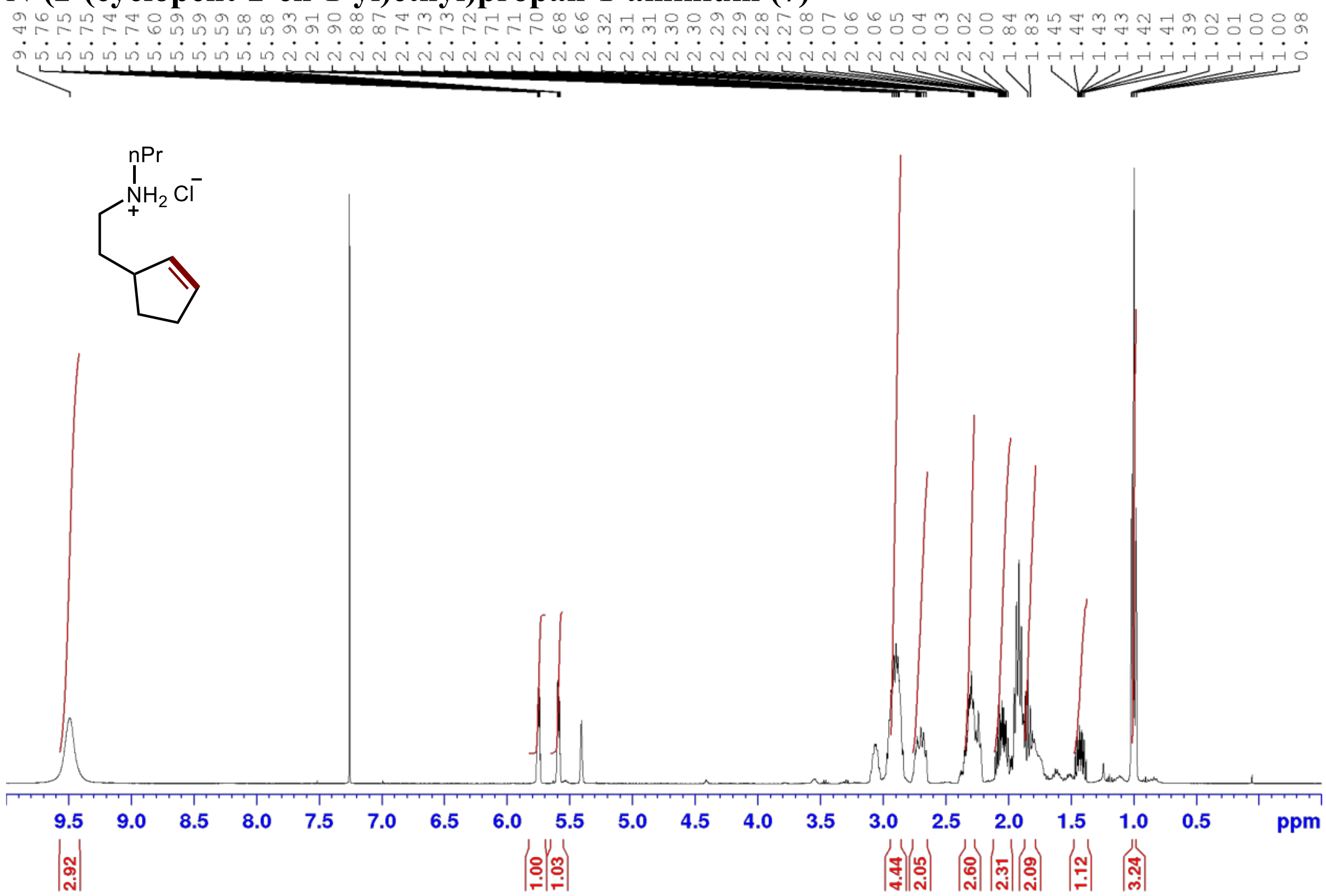

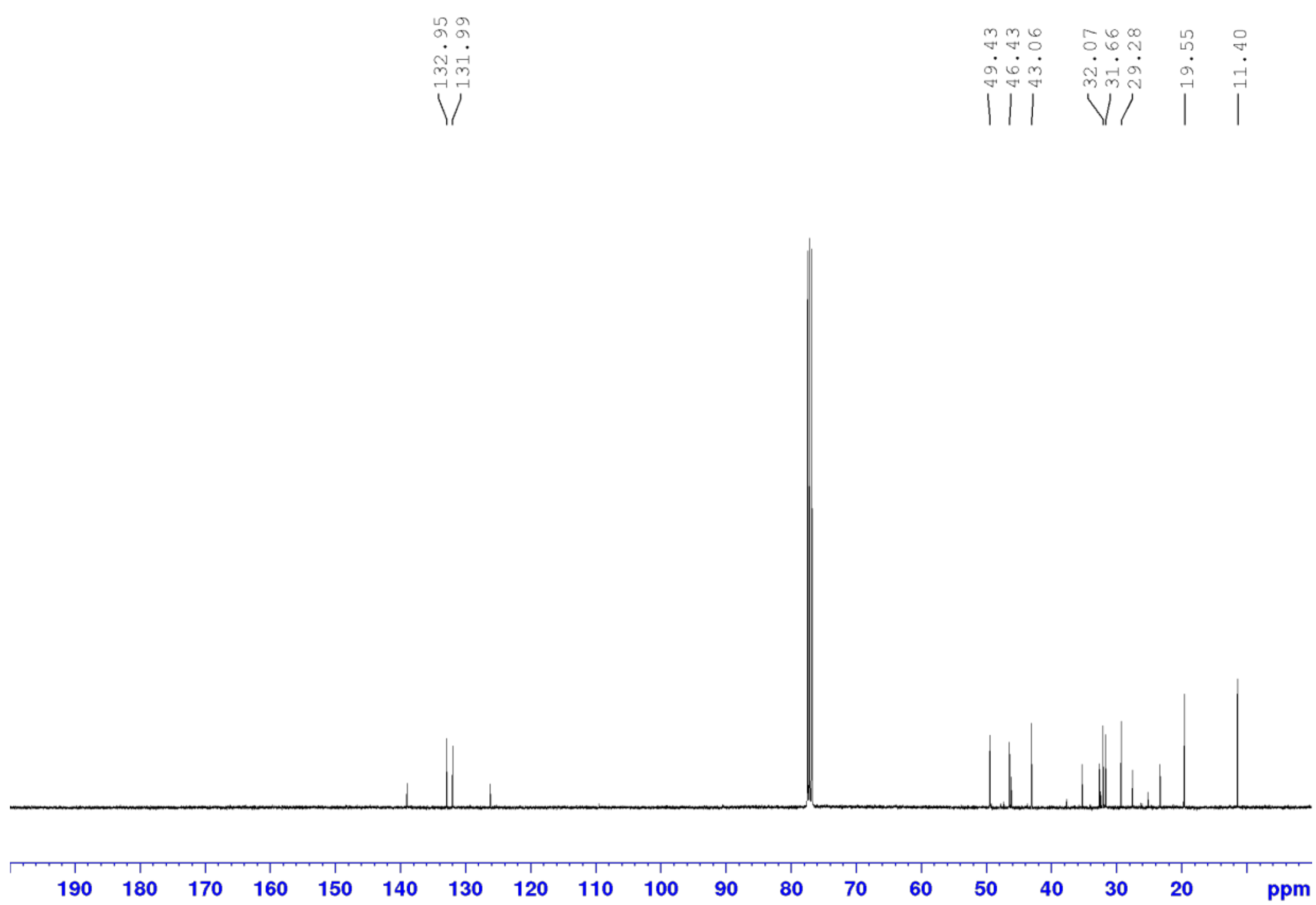

N-(2-(cyclohex-2-en-1-yl)ethyl)propan-1-aminium (8)

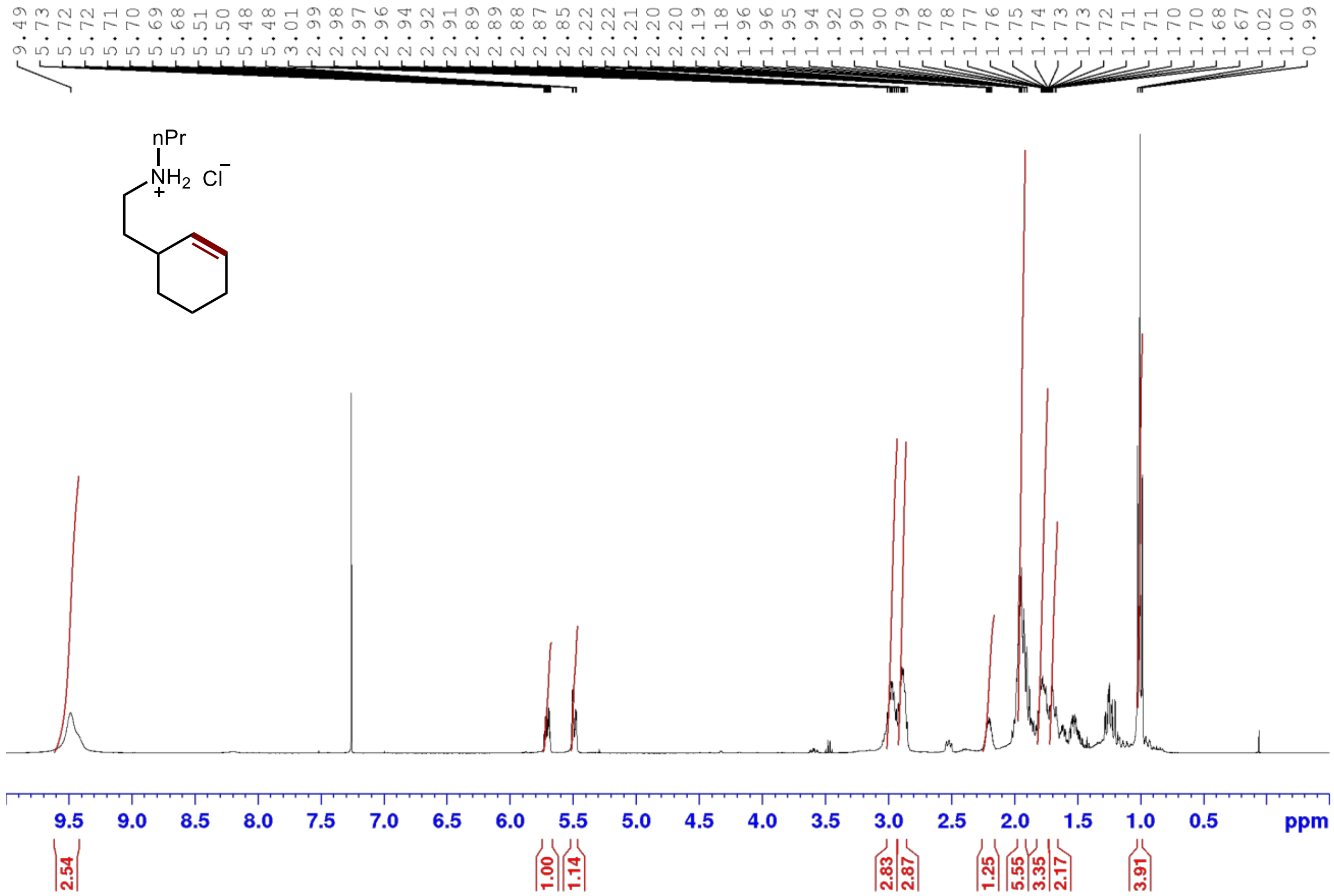

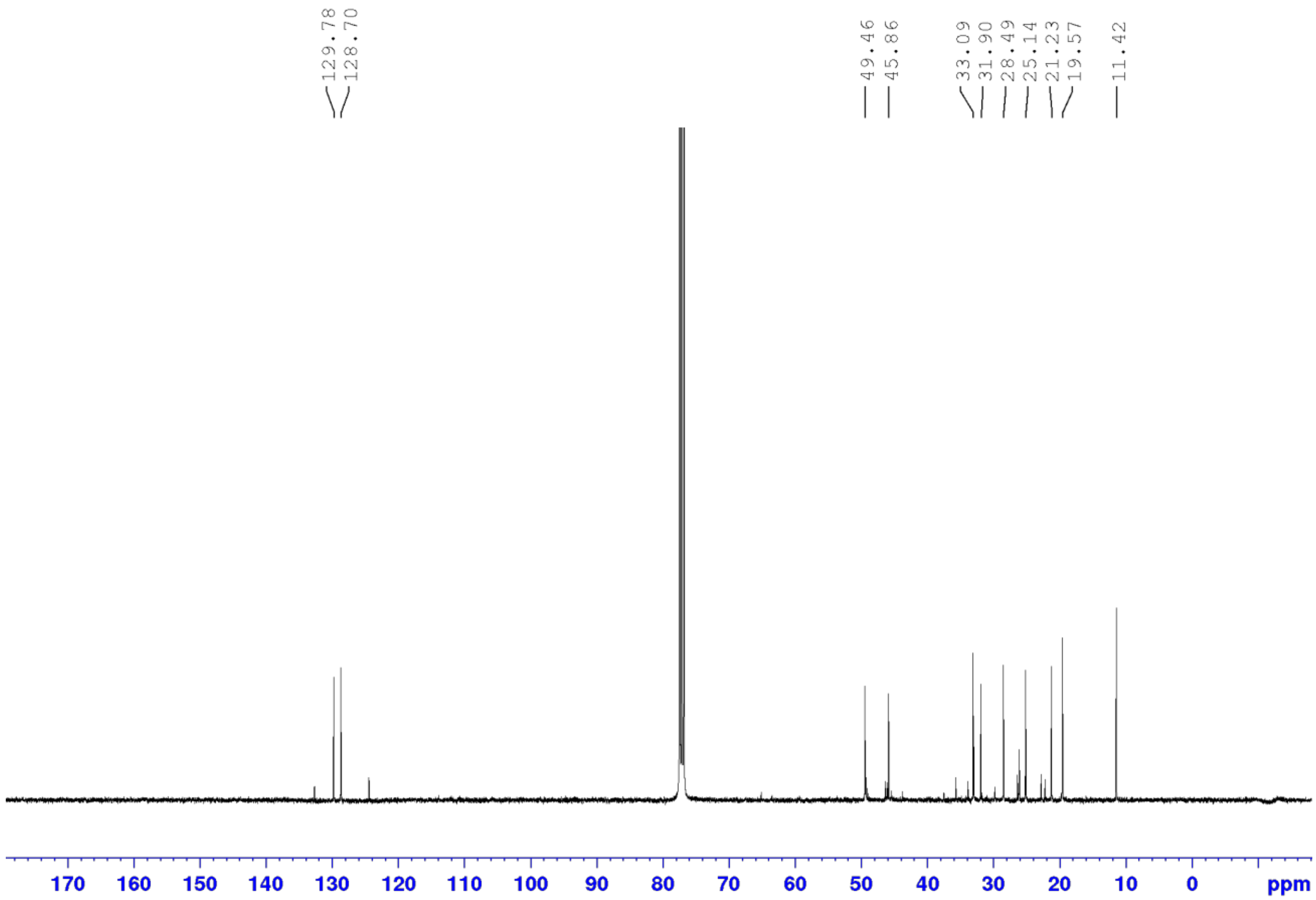

(E)-2-(pent-3-en-1-yl)piperidin-1-ium (9)

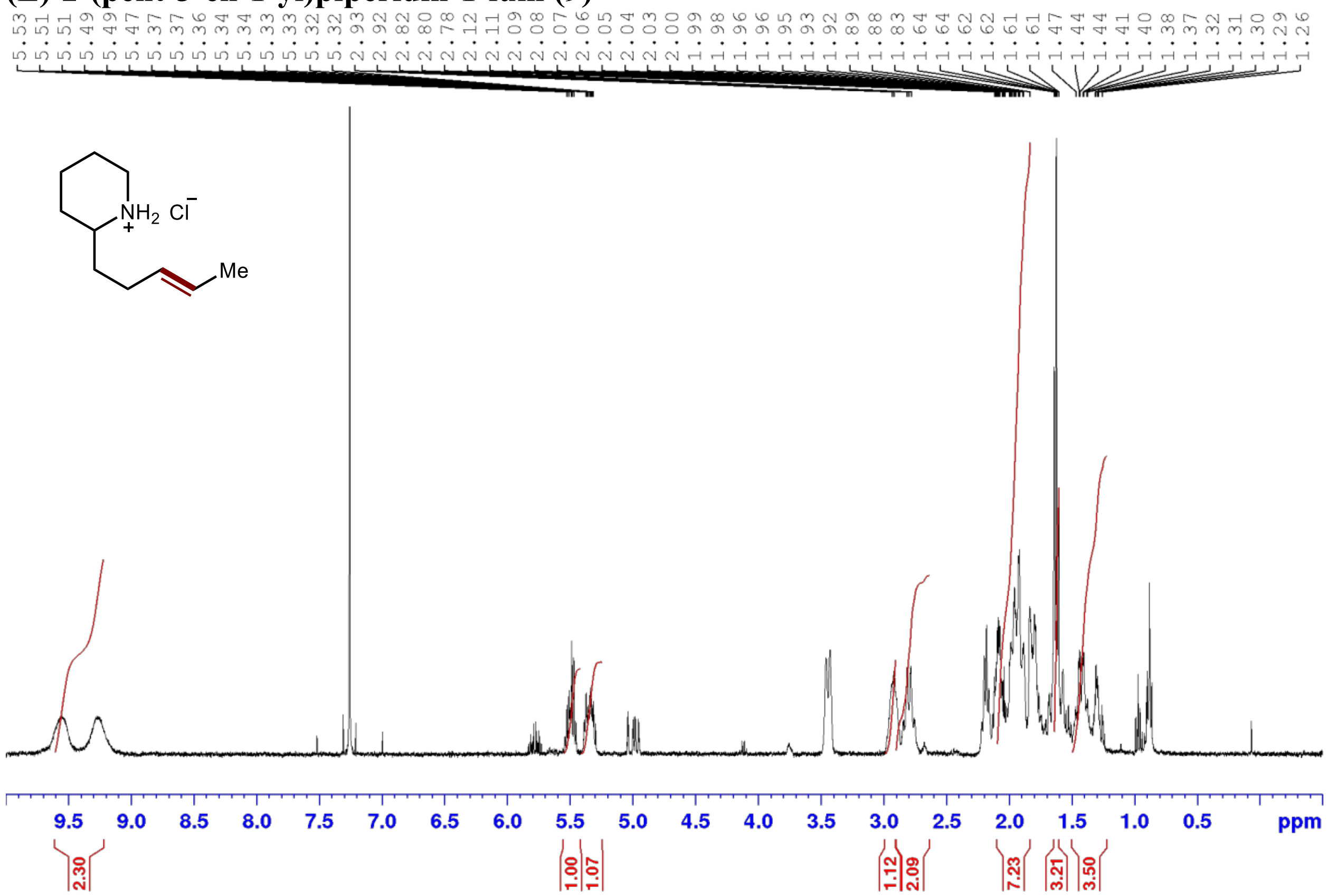

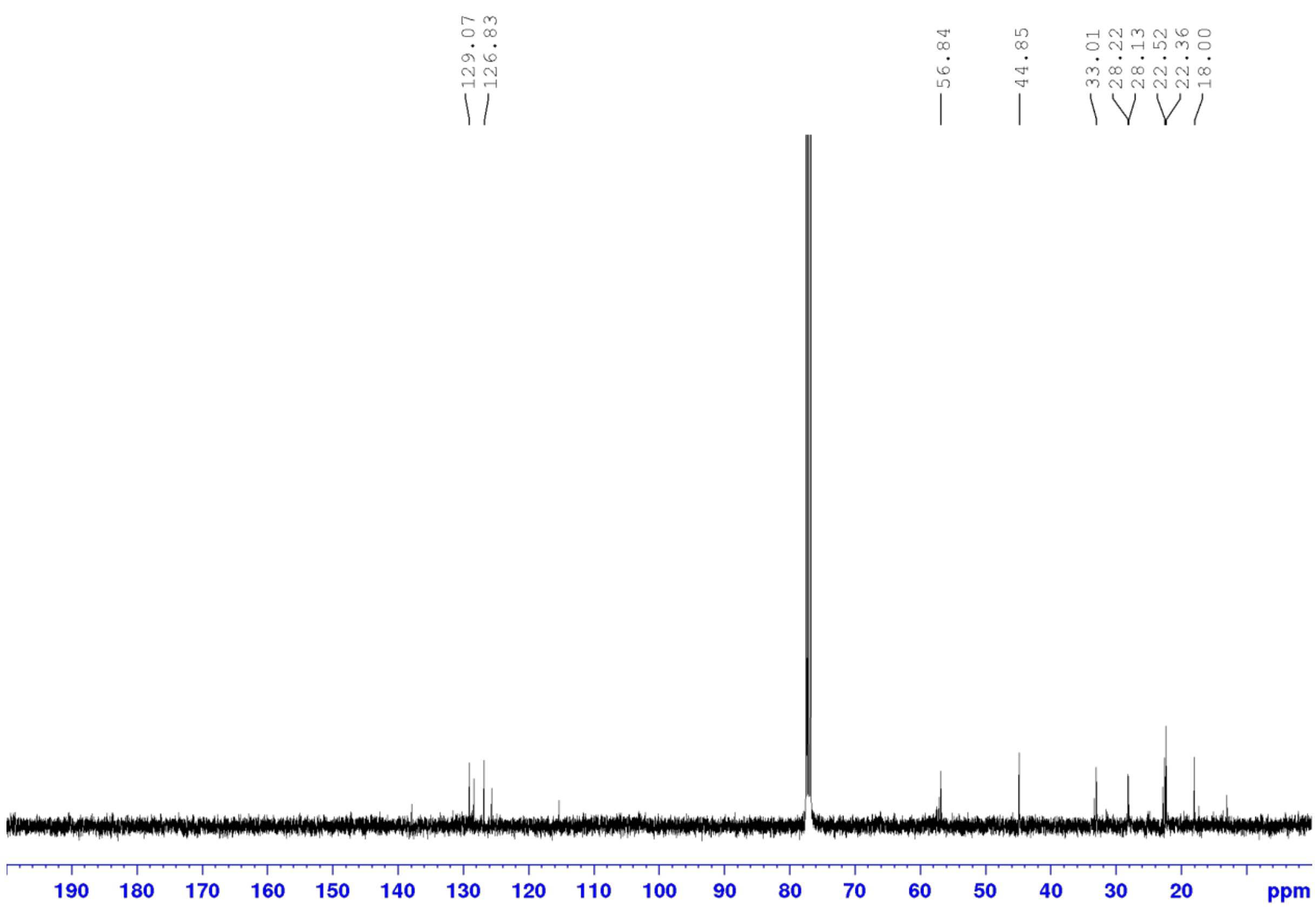

N-propylpent-4-en-1-aminium (10)

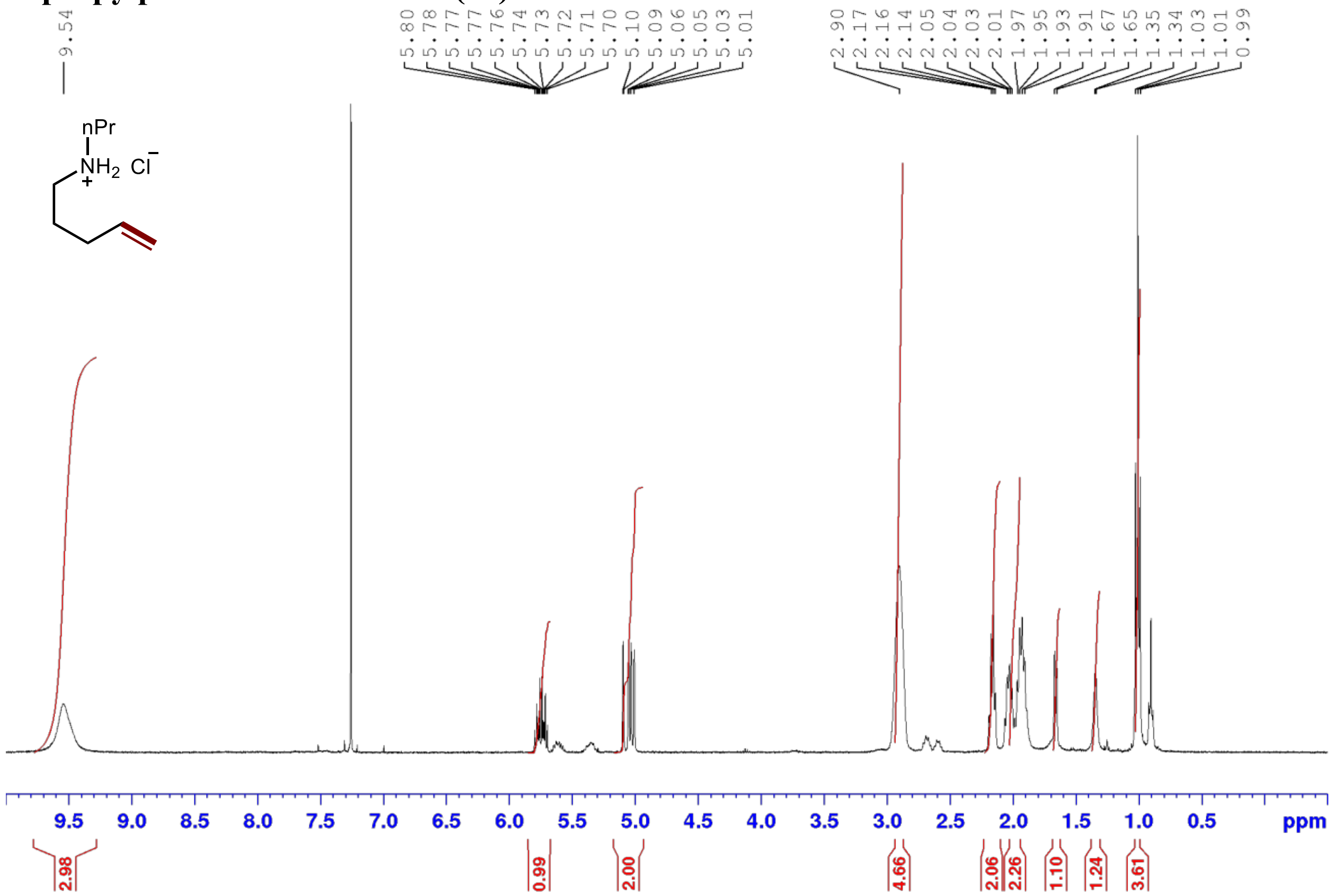

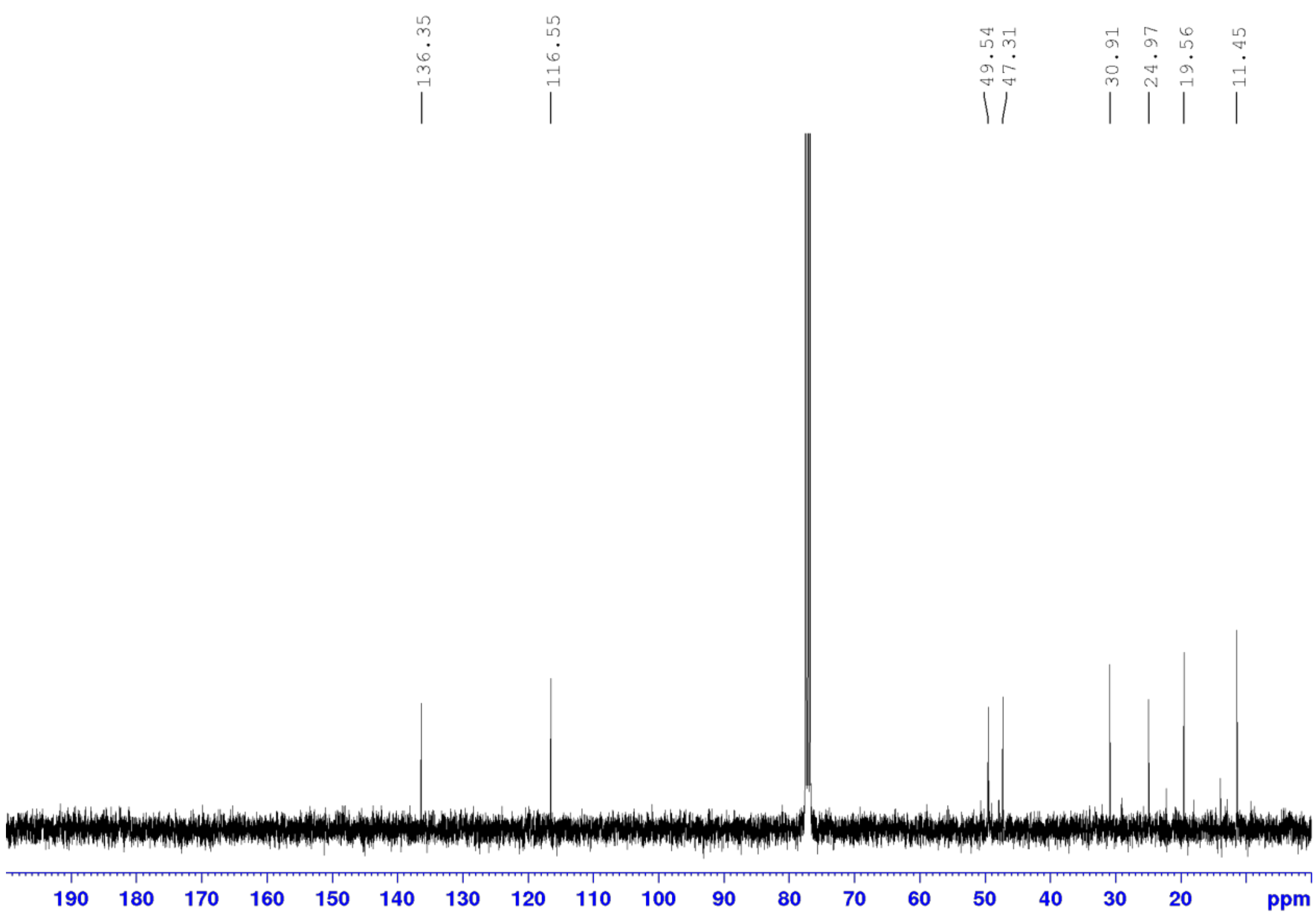

2,2-dimethyl-N-propylpent-4-en-1-aminium (11)

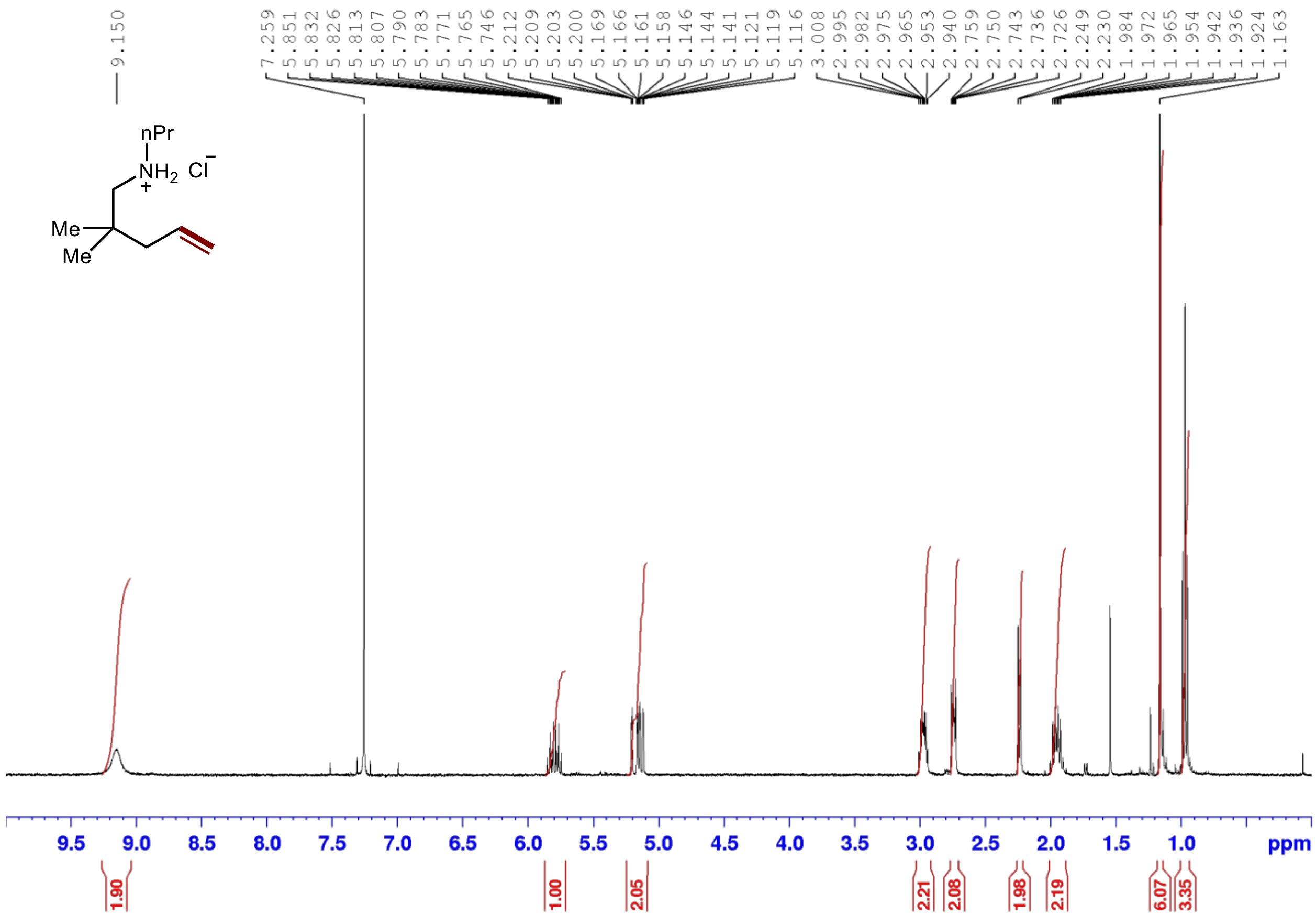

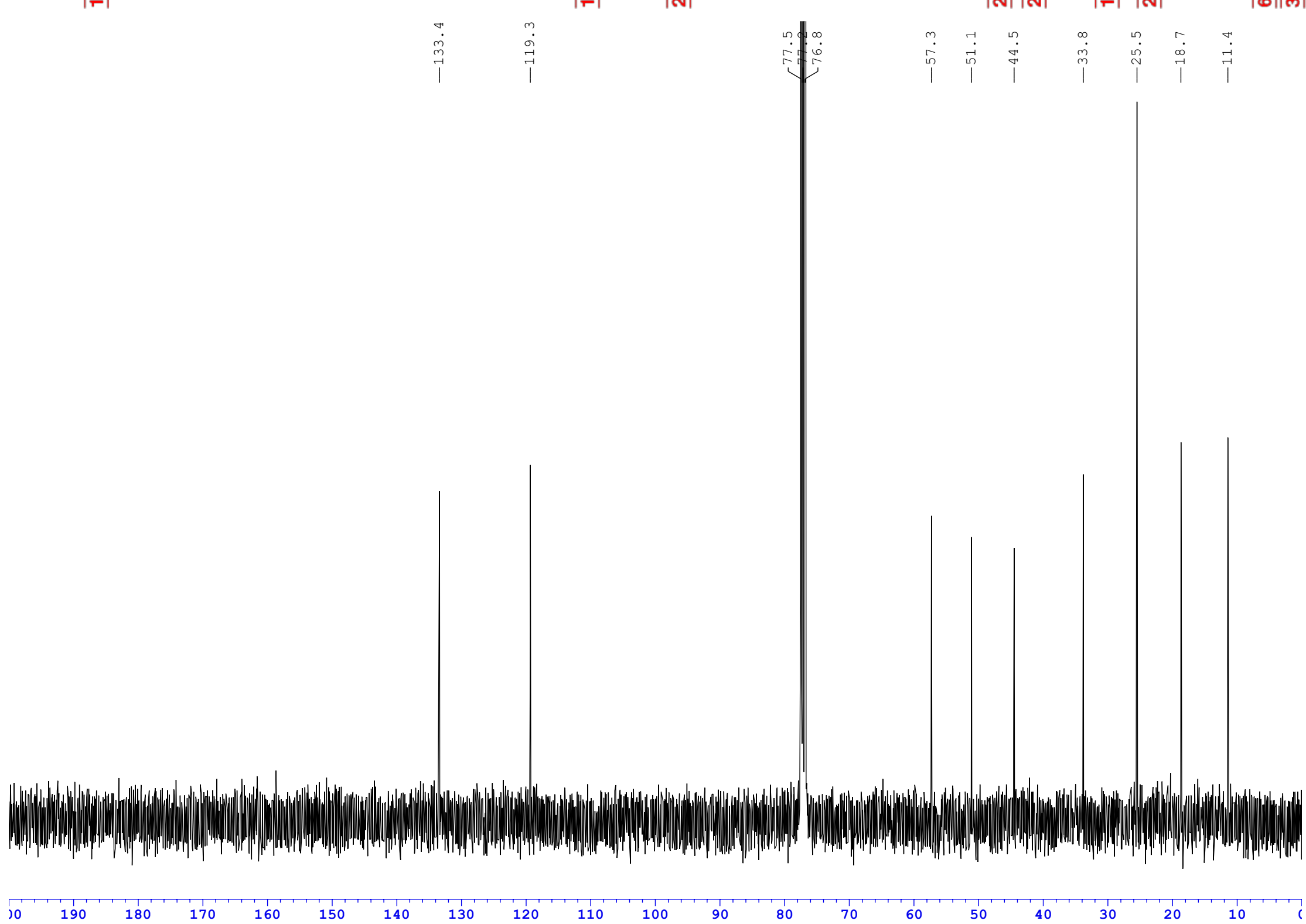

(E)-hex-4-en-1-aminium (12)

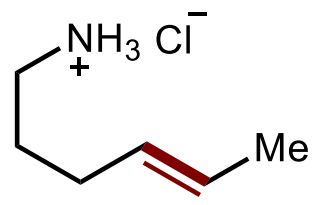

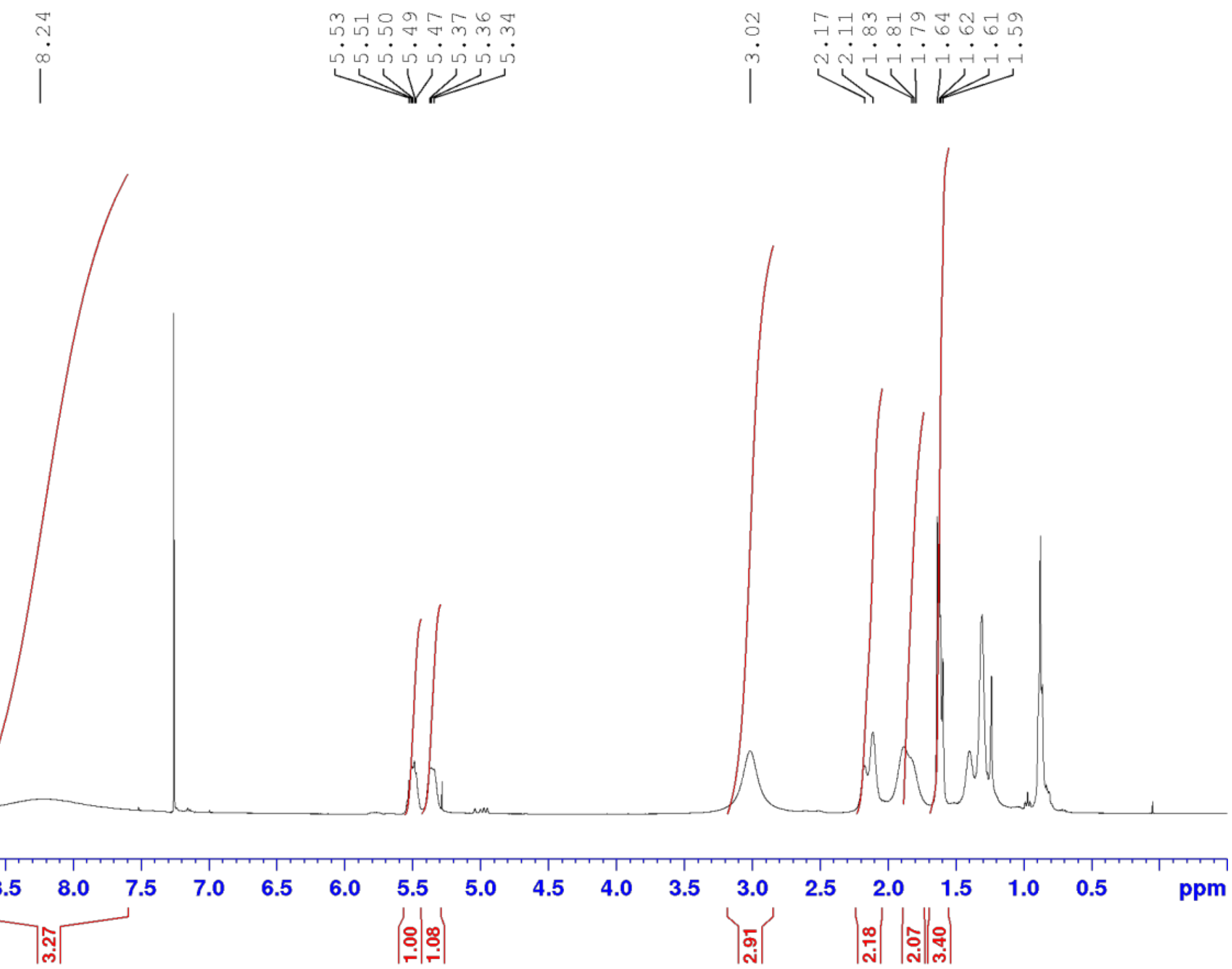

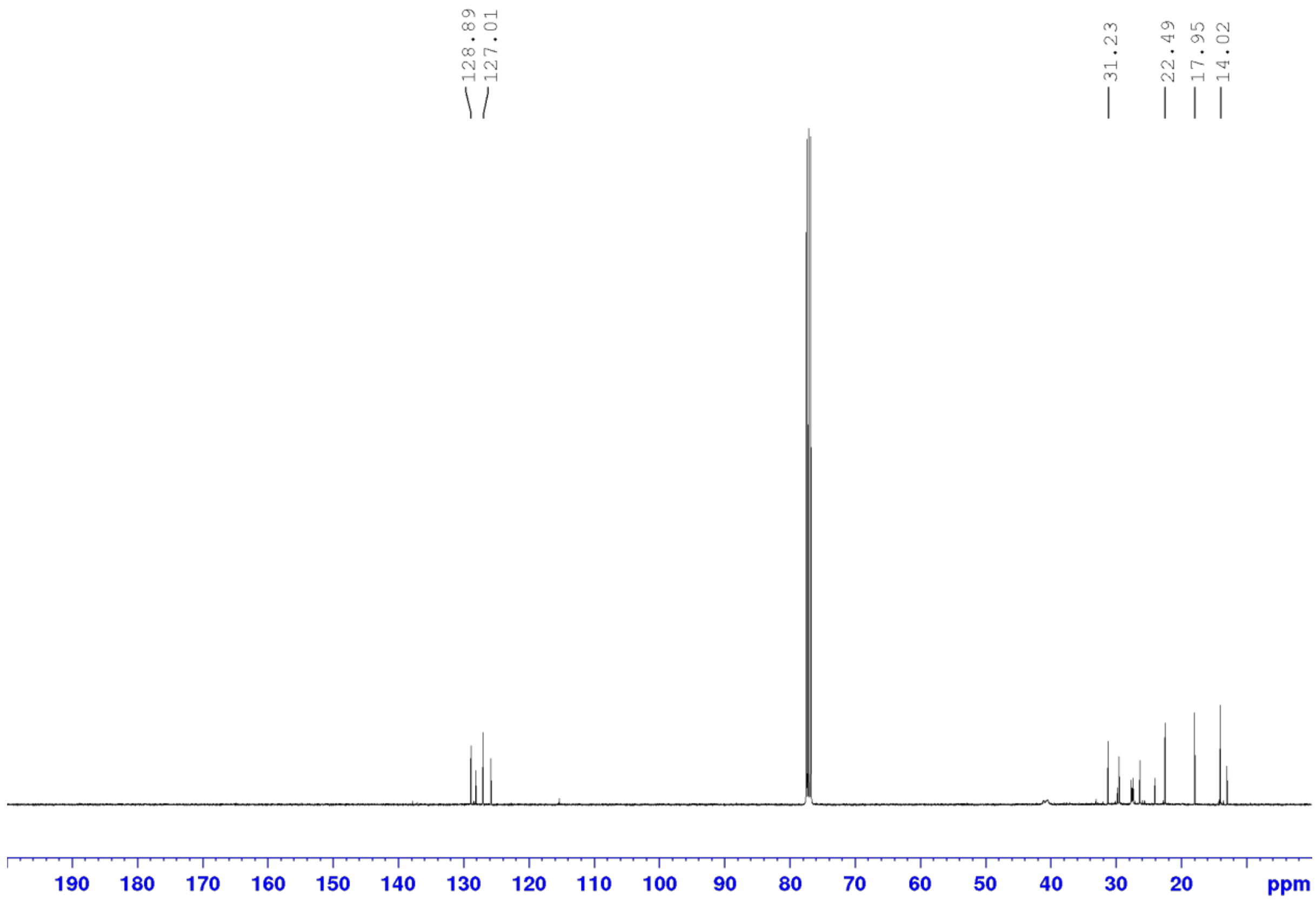

(E)-hept-5-en-2-aminium (13)

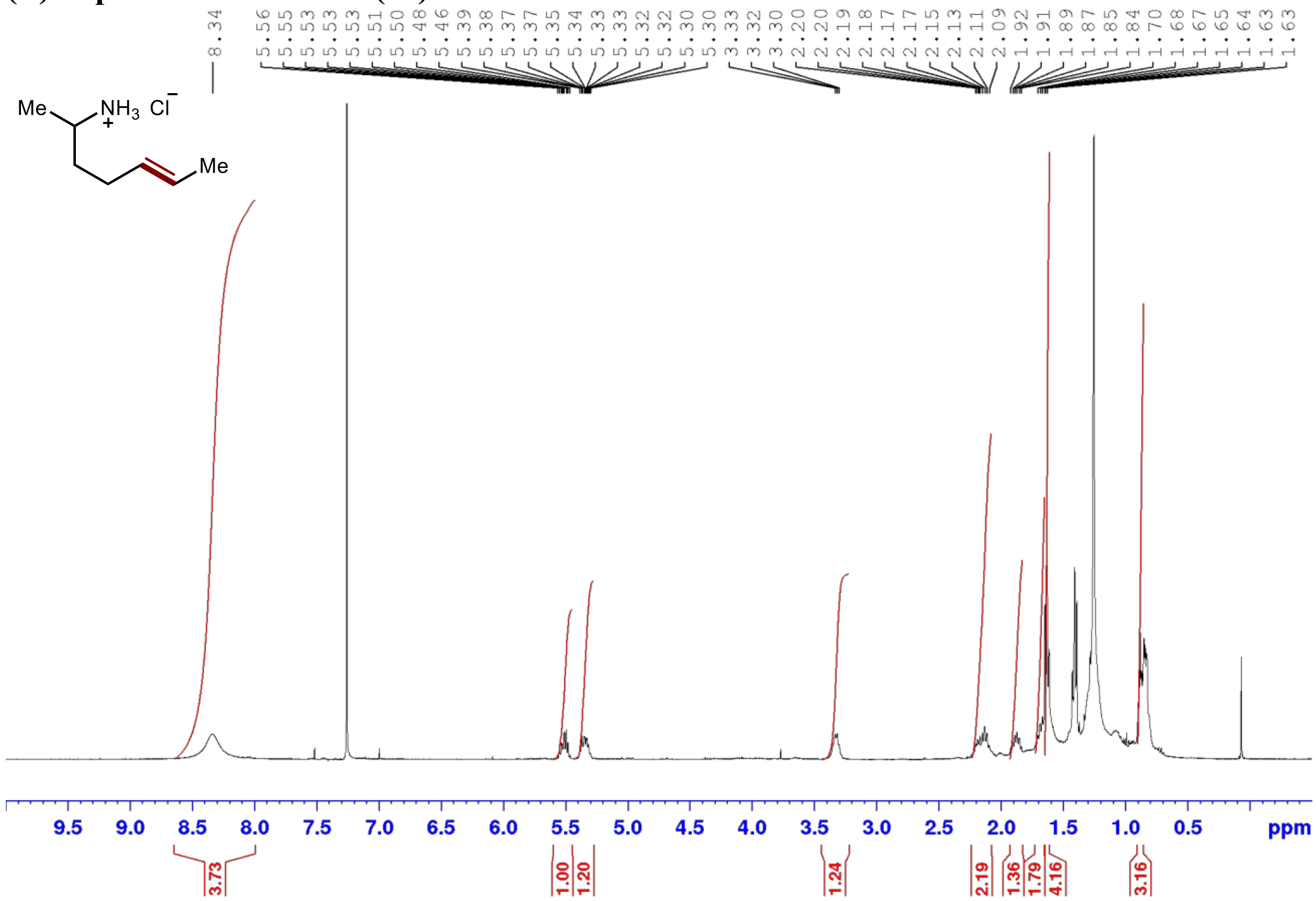

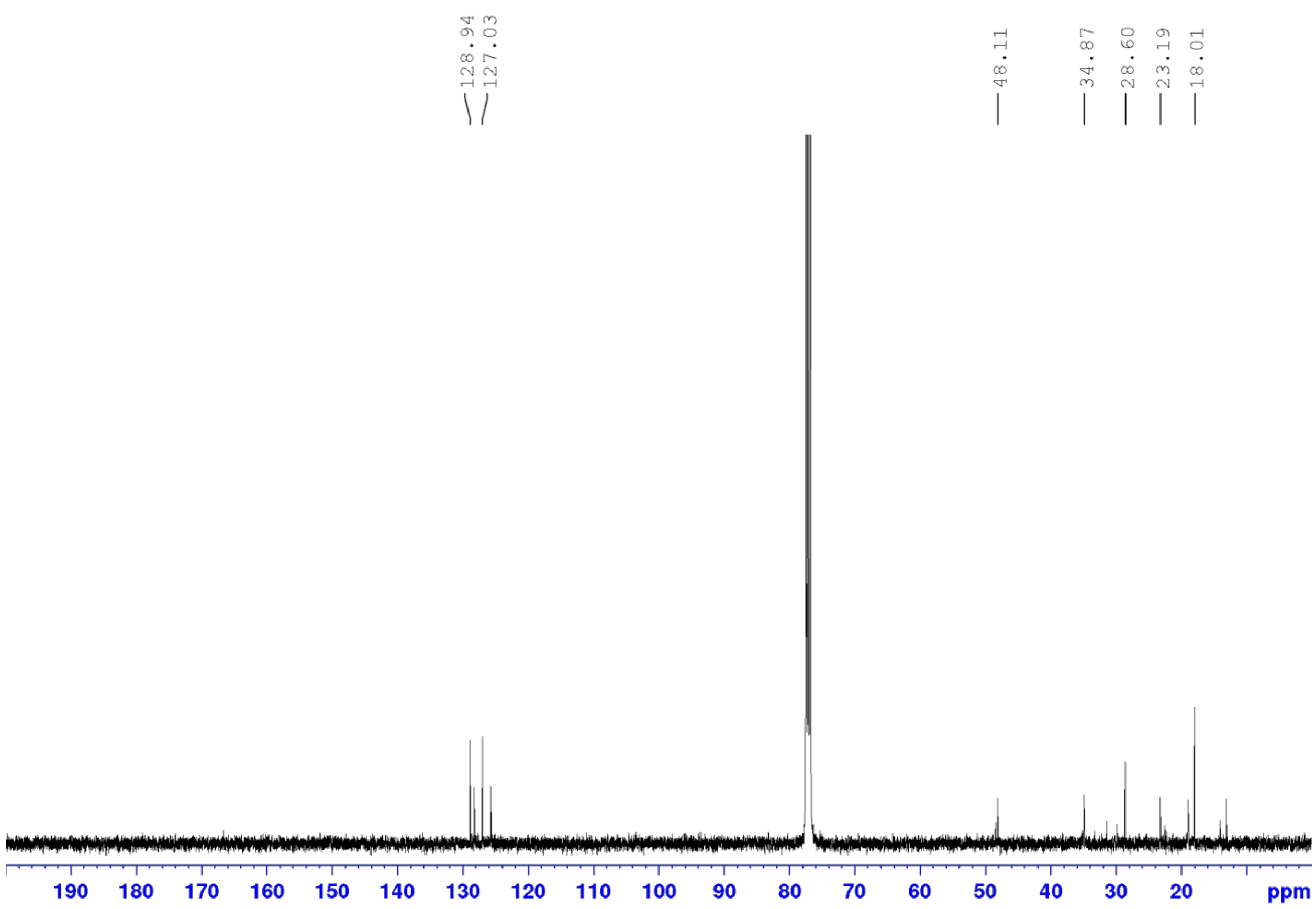

(E)-undec-2-en-6-aminium (14)

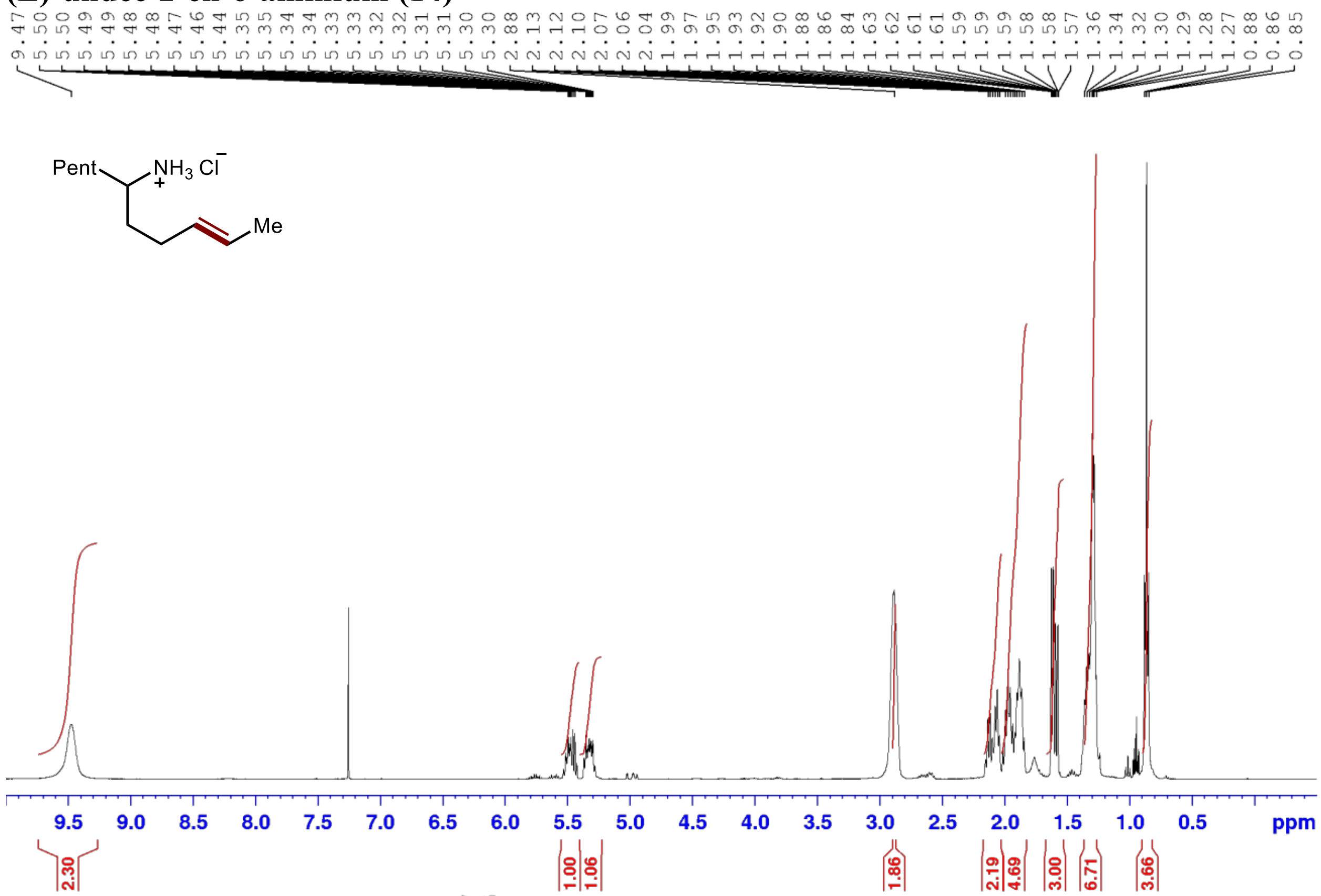

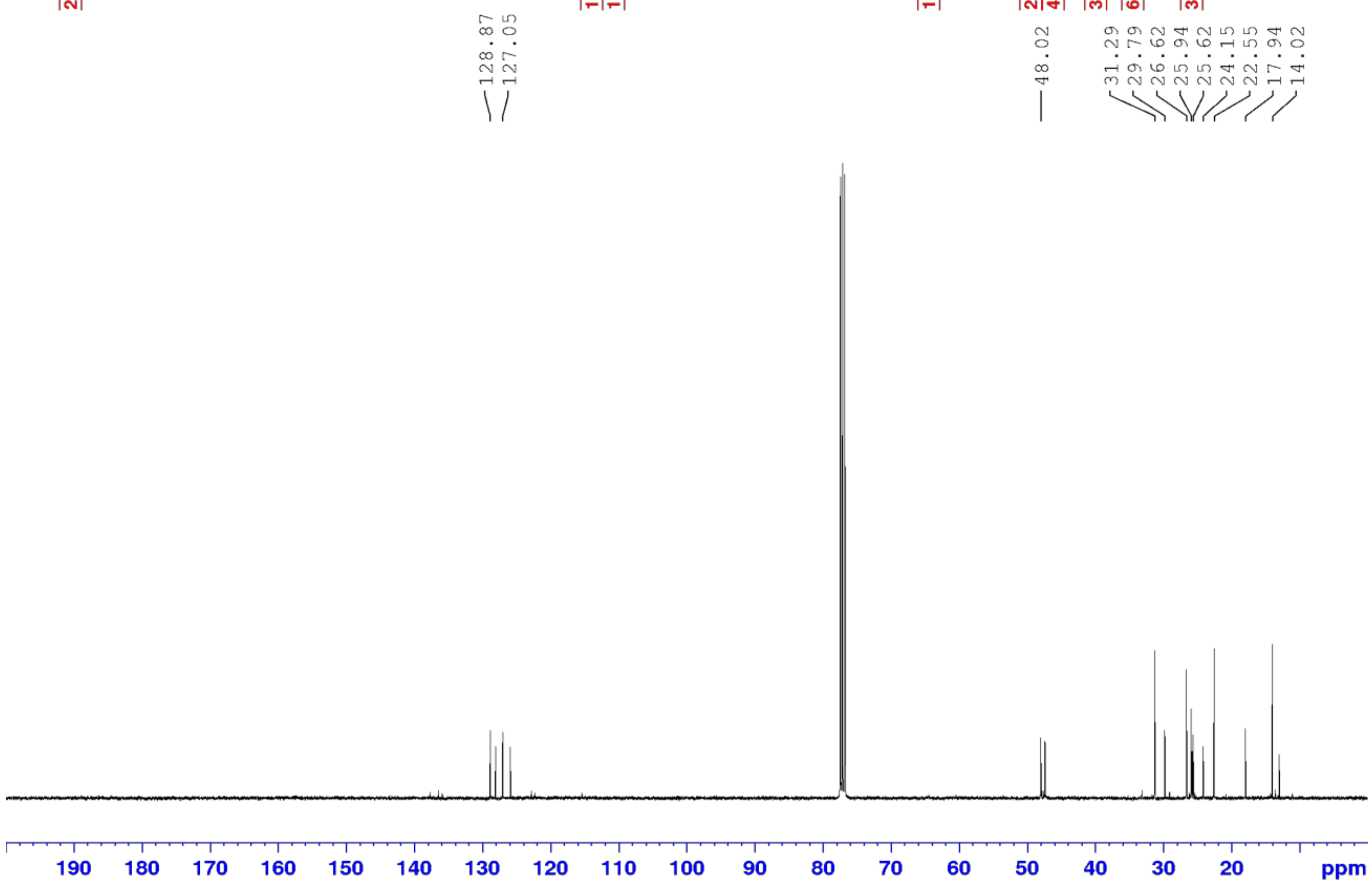

((1S,4aR,10aS)-7-isopropyl-1,4a-dimethyl-1,2,3,4,4a,10a-hexahydrophenanthren-1-yl)methanaminium (15)

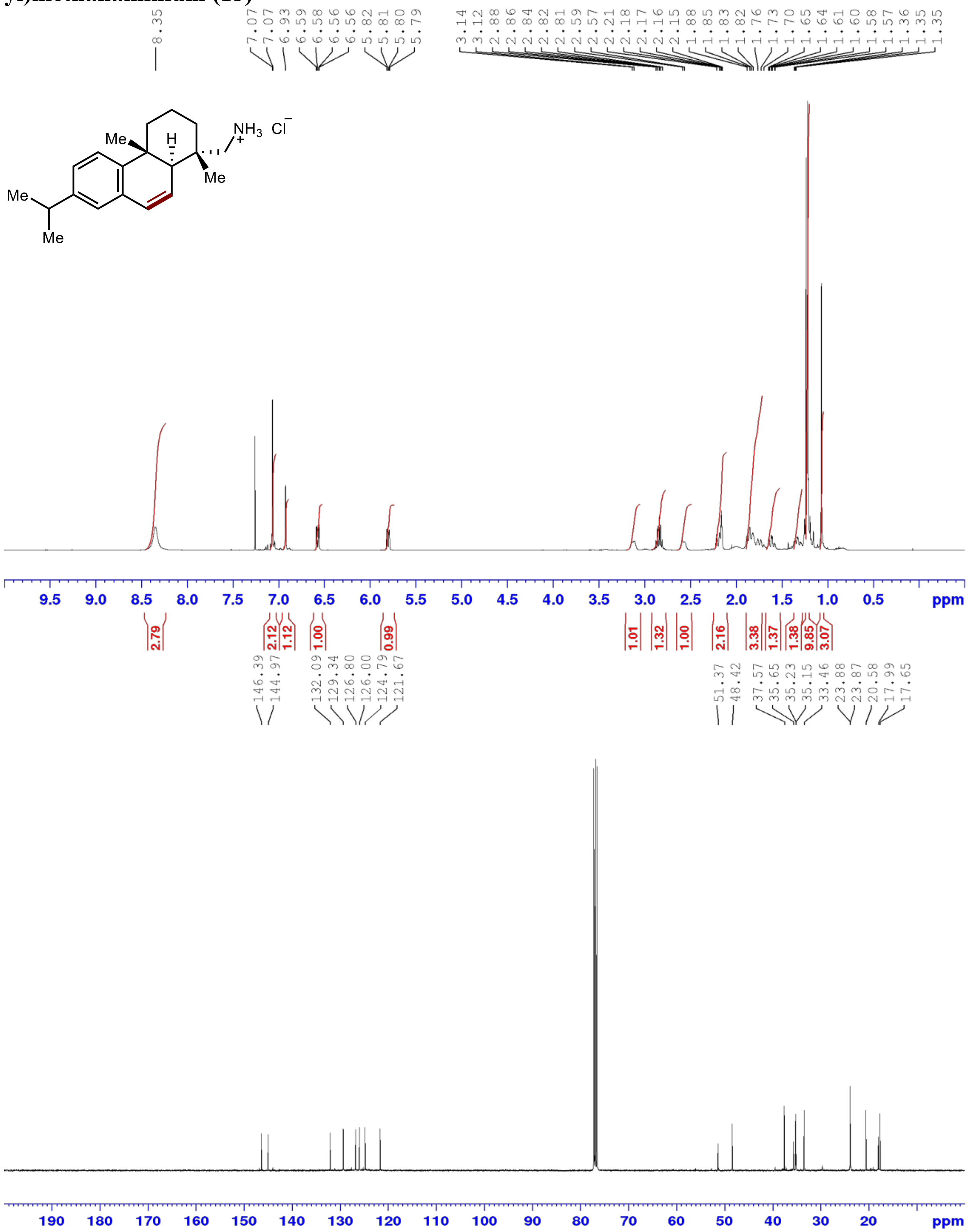

(E)-1-methoxy-1-oxohept-5-en-2-aminium (16)

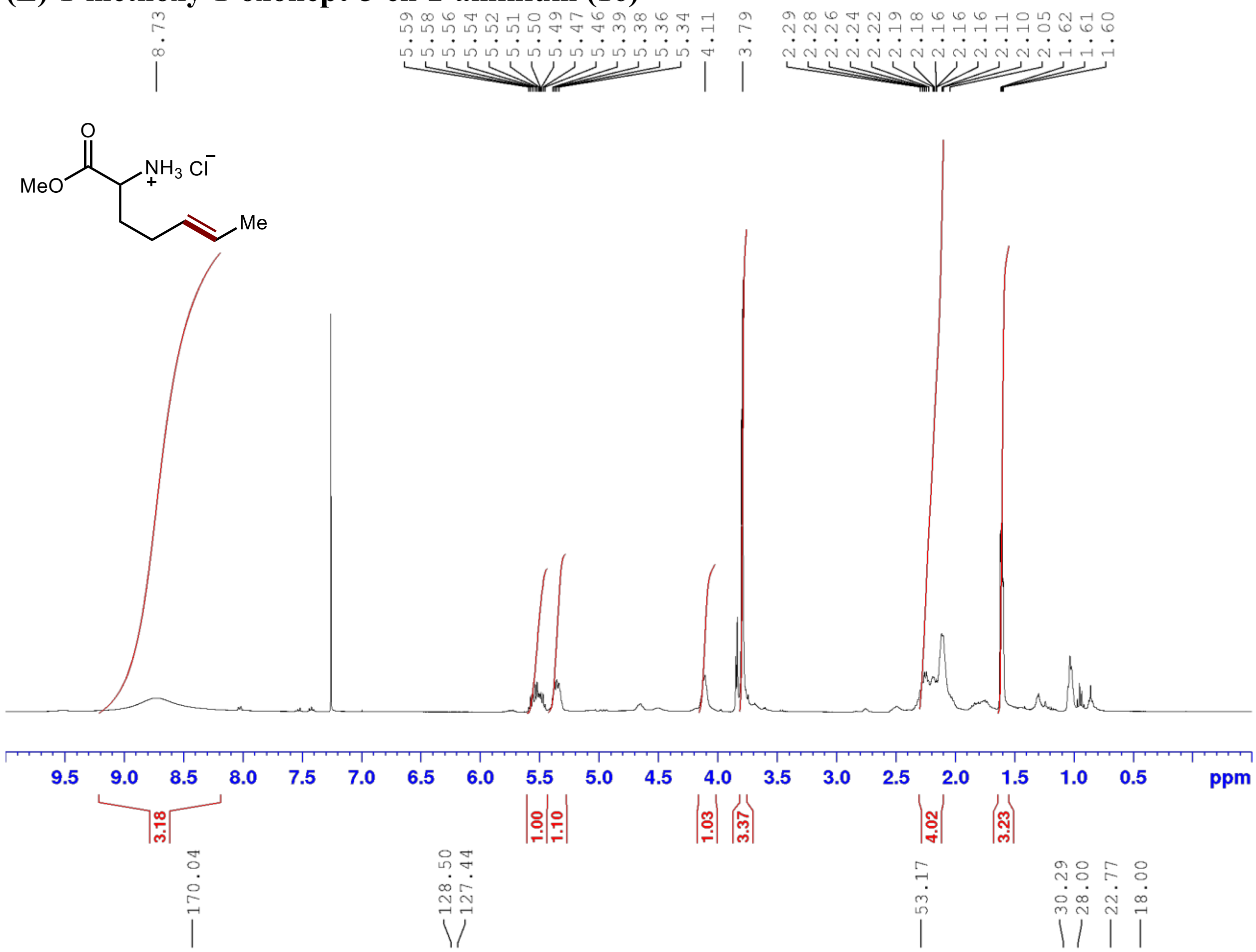

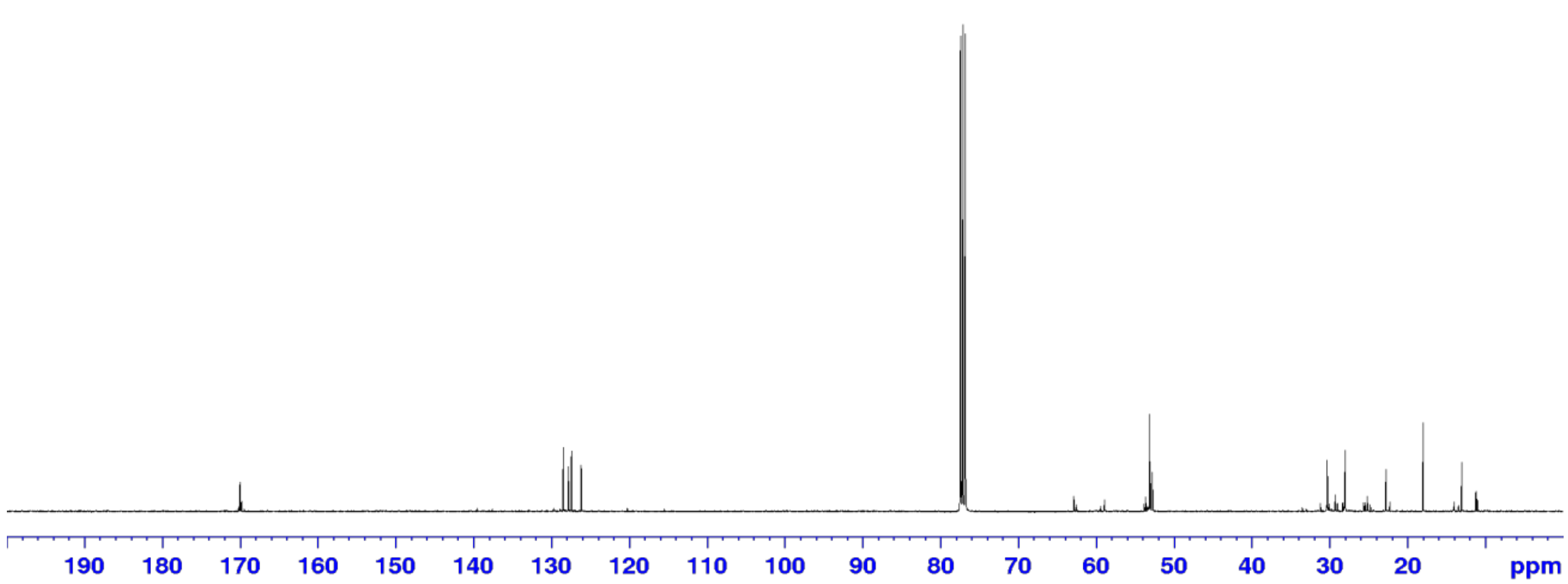

2-(chloromethyl)-1-propylpyrrolidin-1-ium(17)

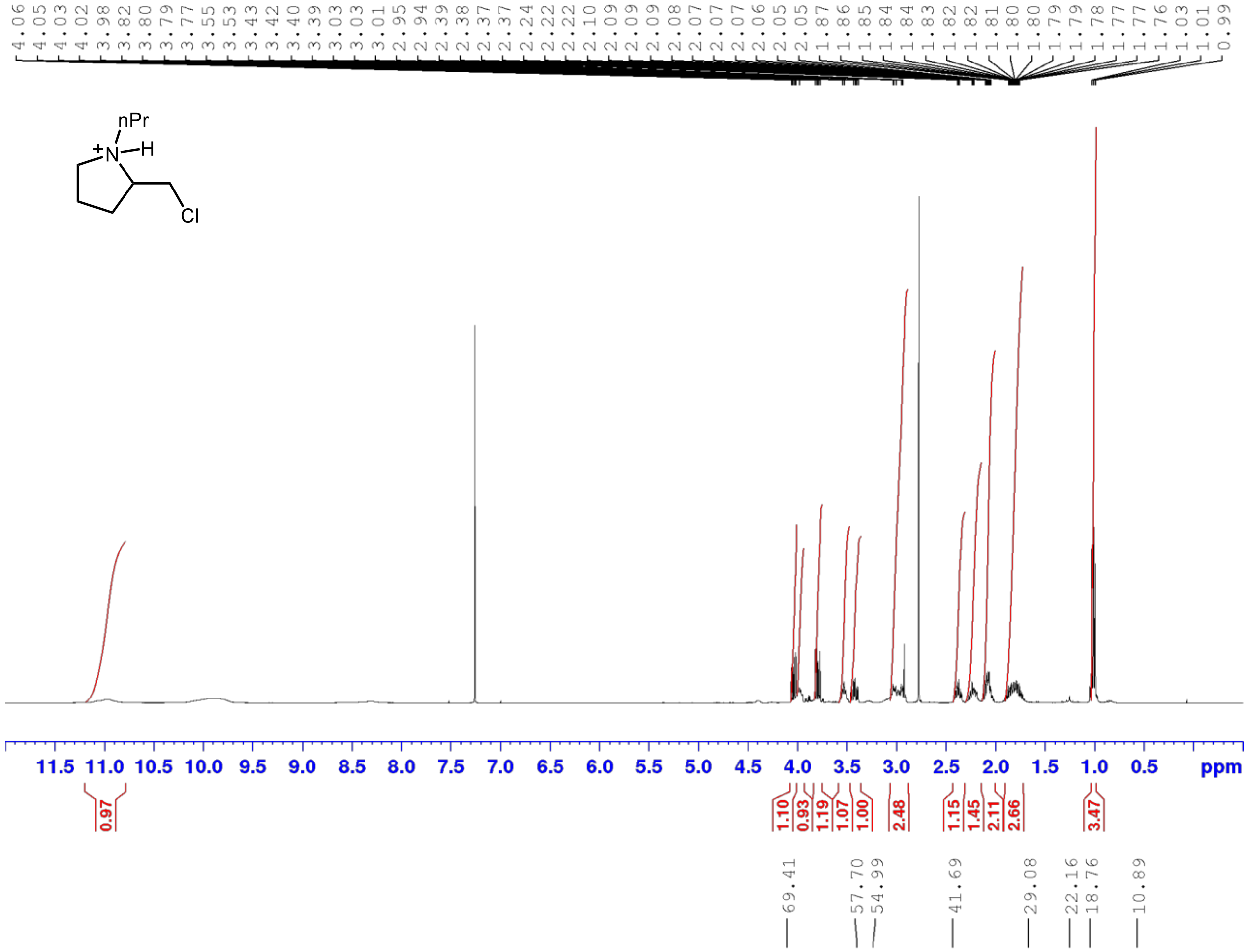

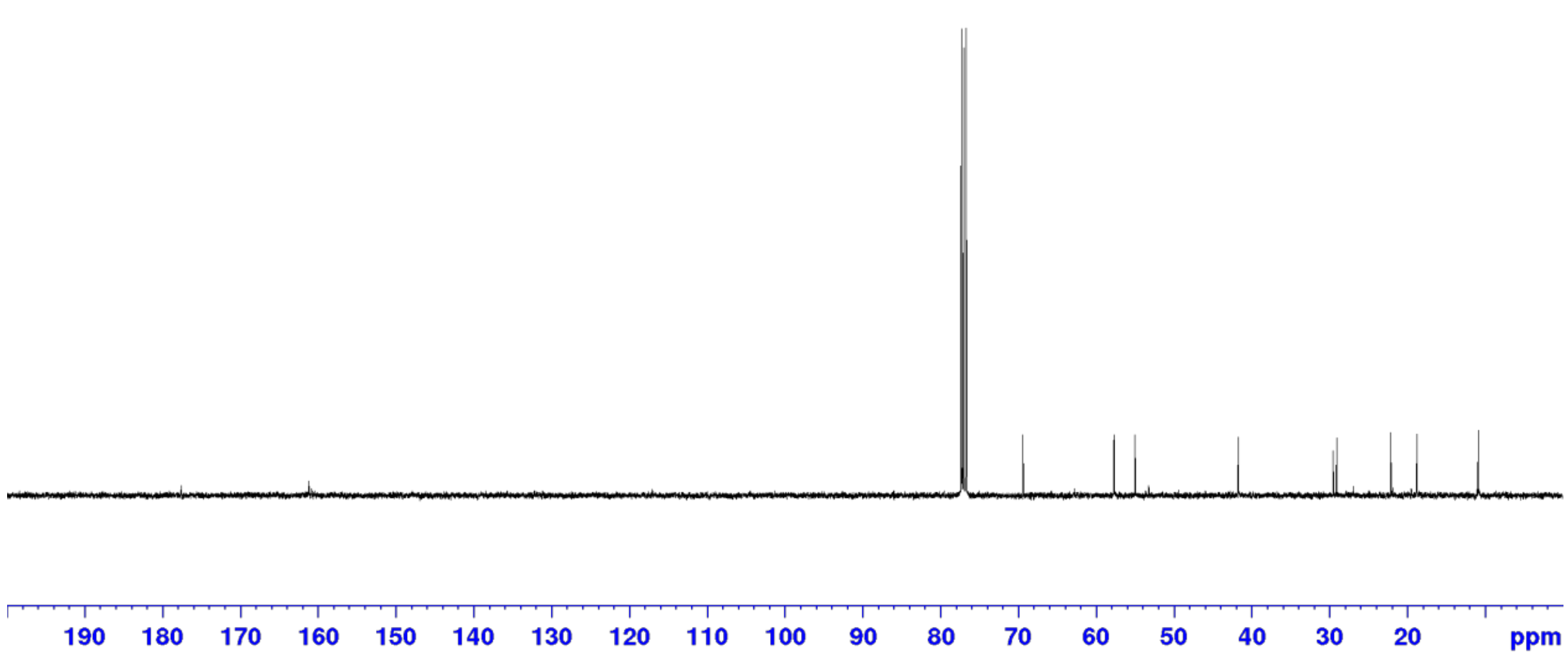

2-(hydroxymethyl)-1-propylpyrrolidin-1-ium **(18)**

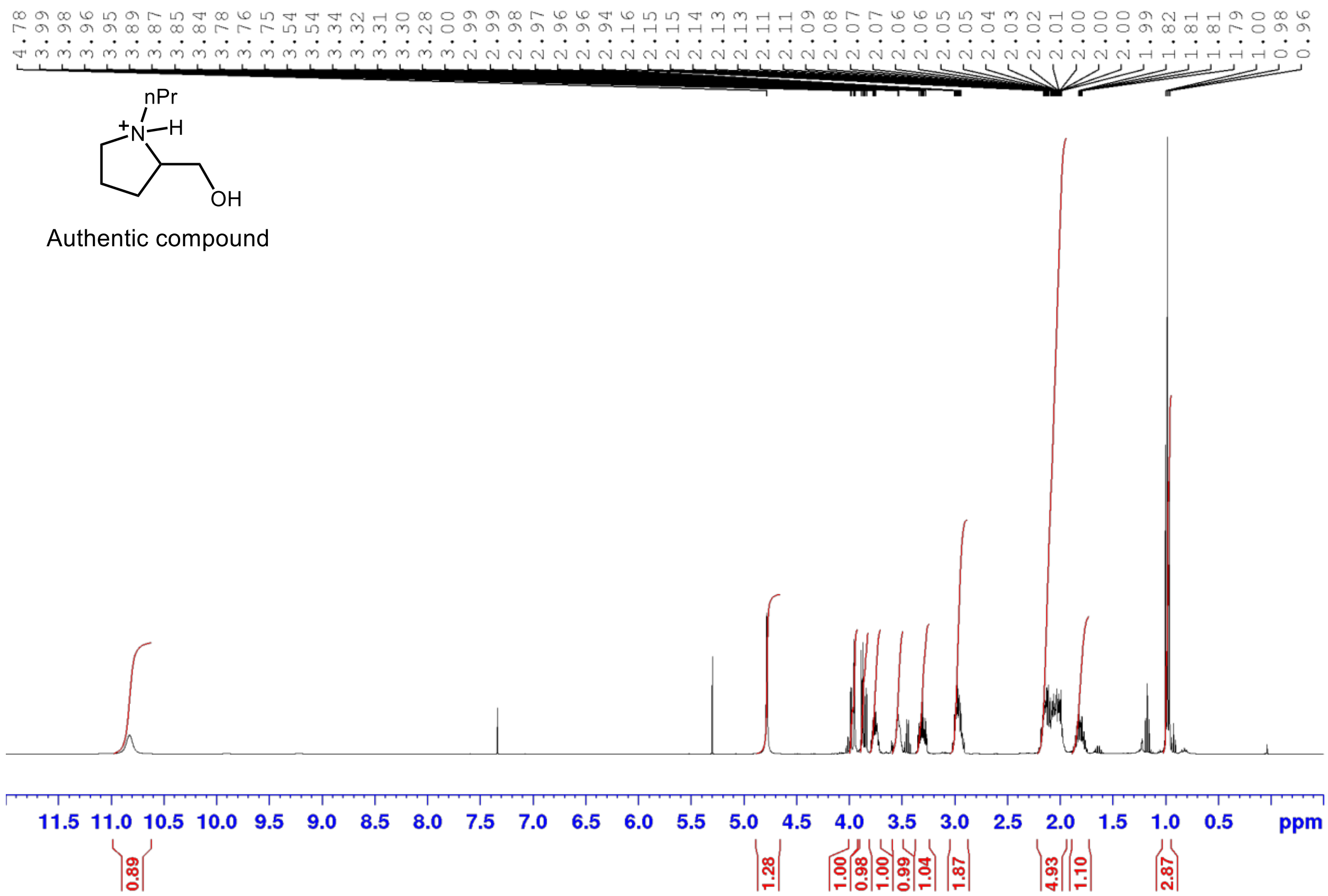

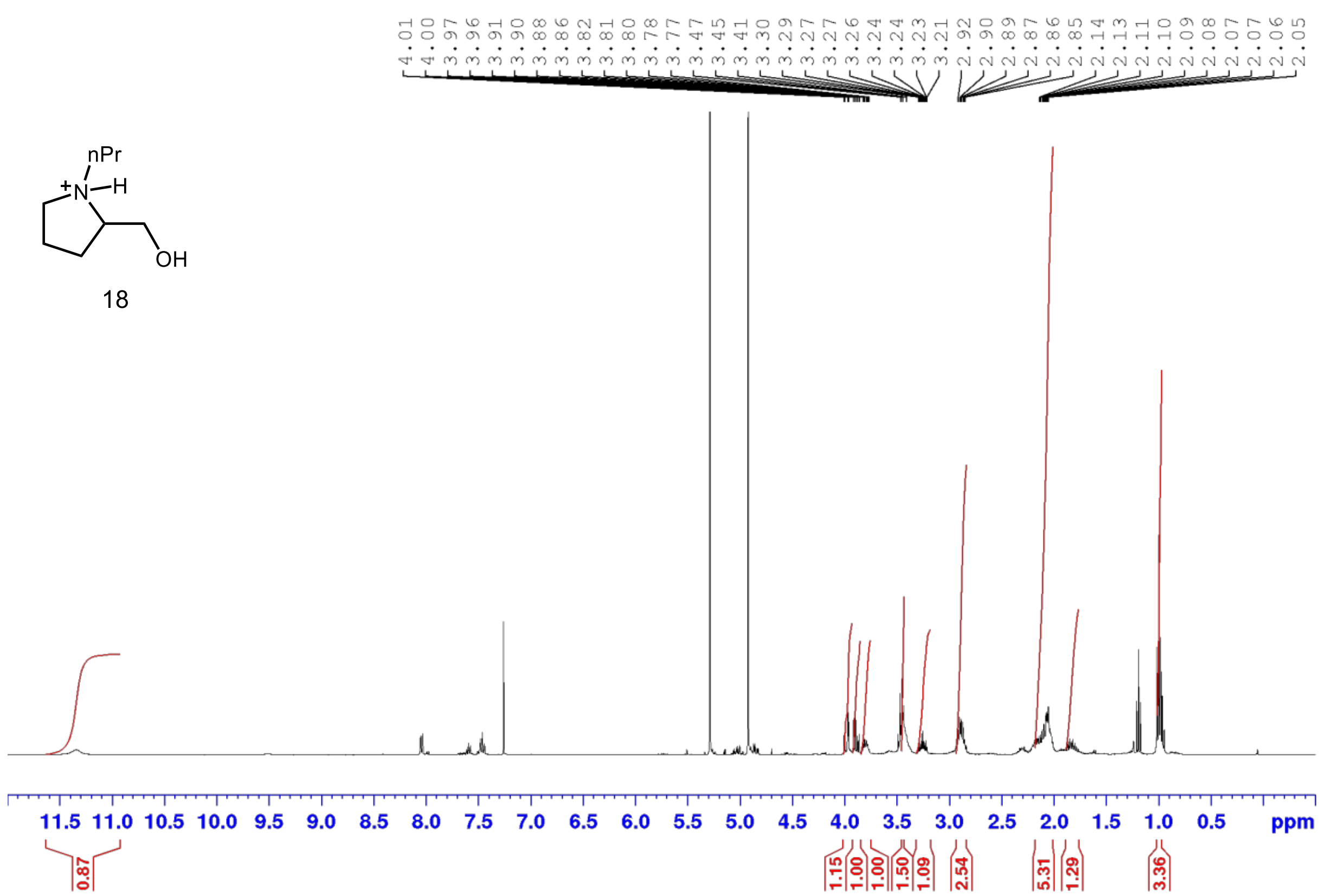

5-chloro-3,3-dimethyl-1-propylpiperidine (19)

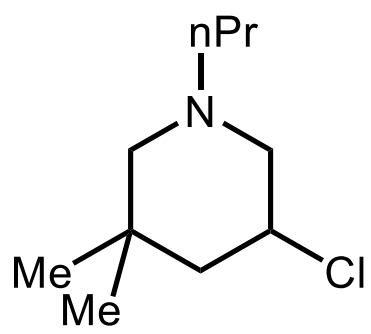

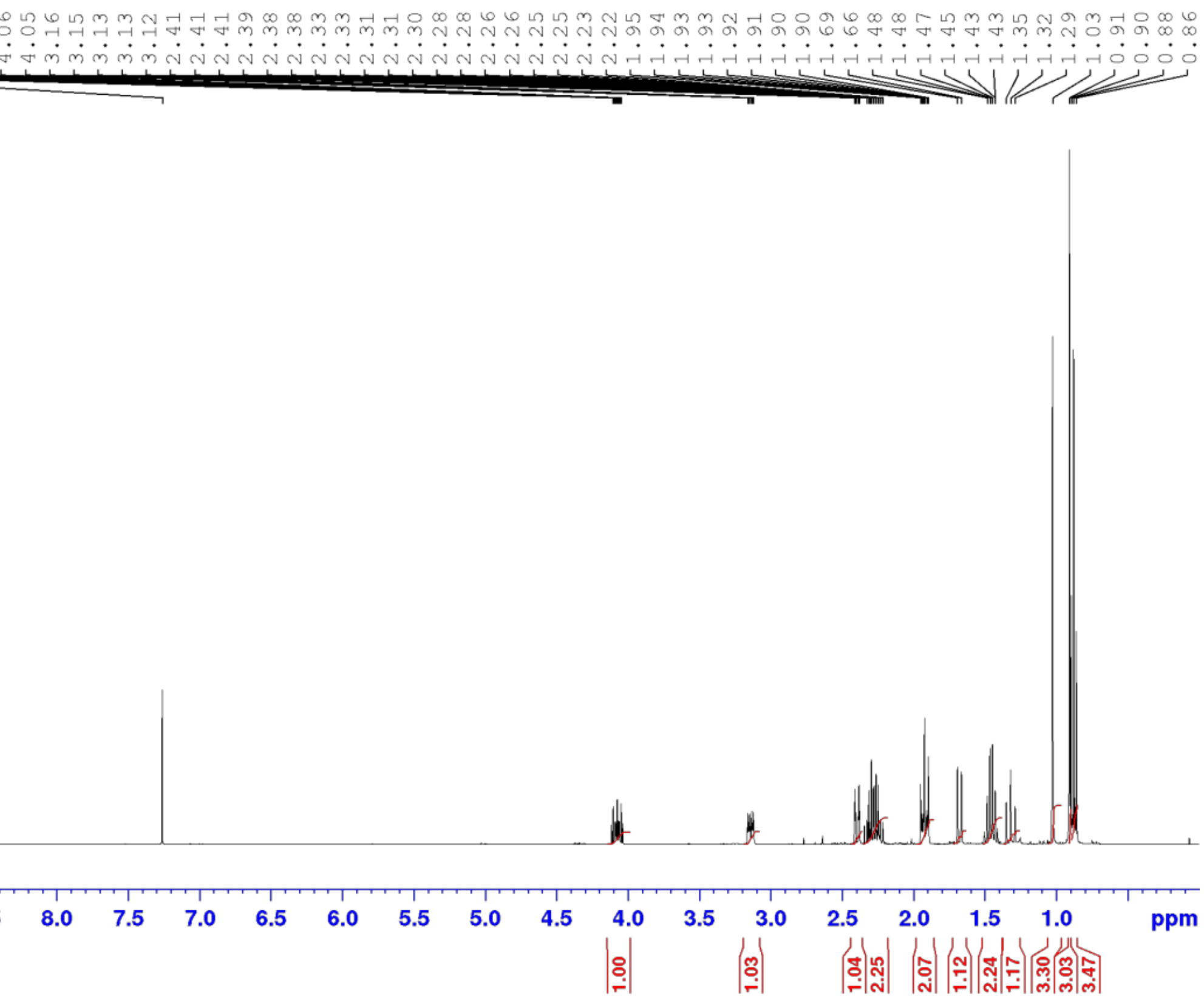

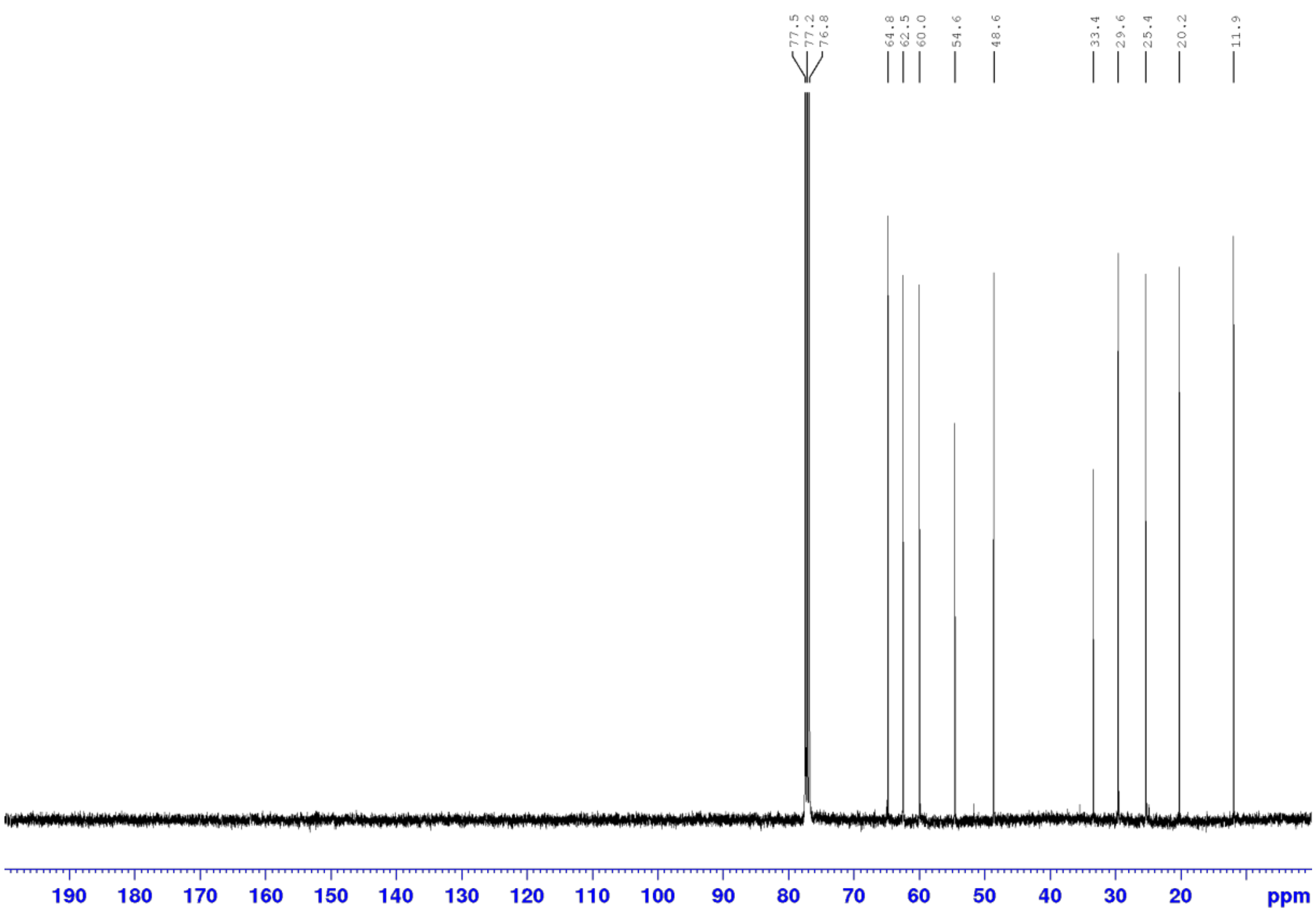

5-fluoro-3,3-dimethyl-1-propylpiperidine (20)

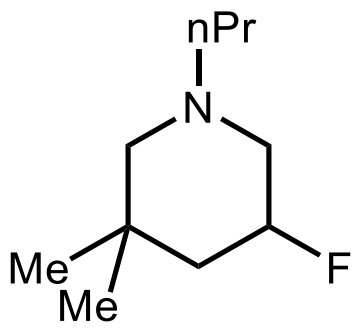

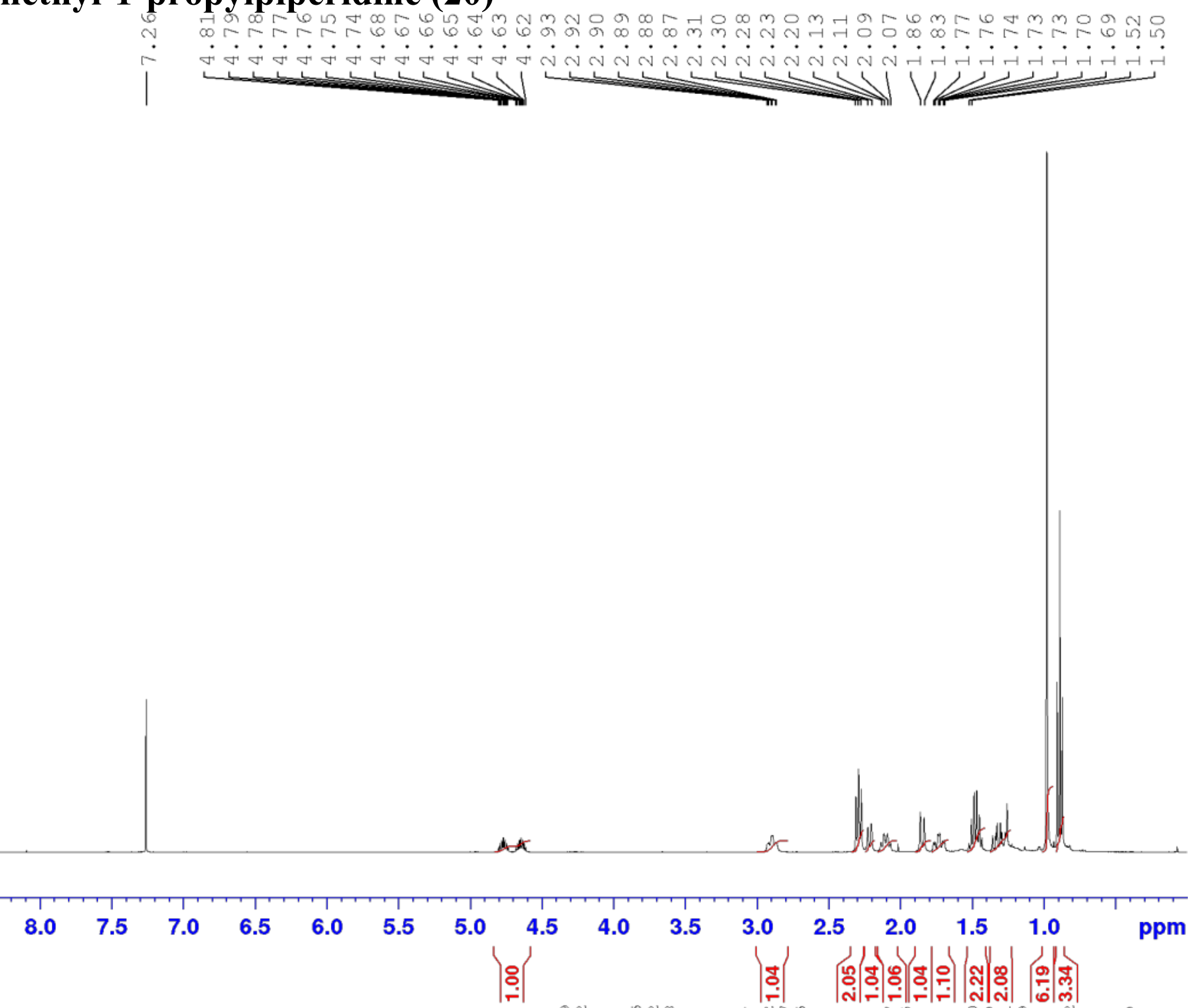

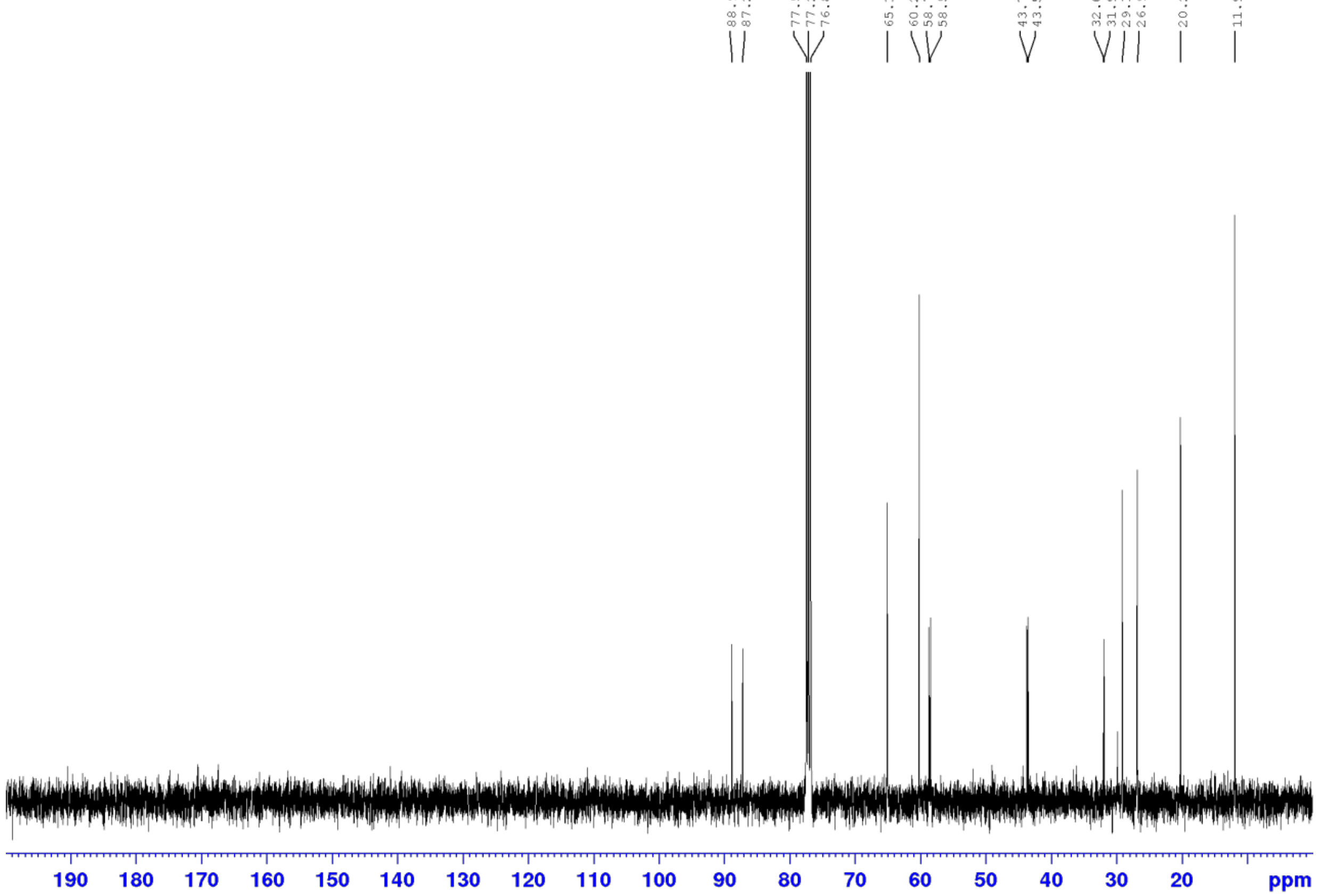

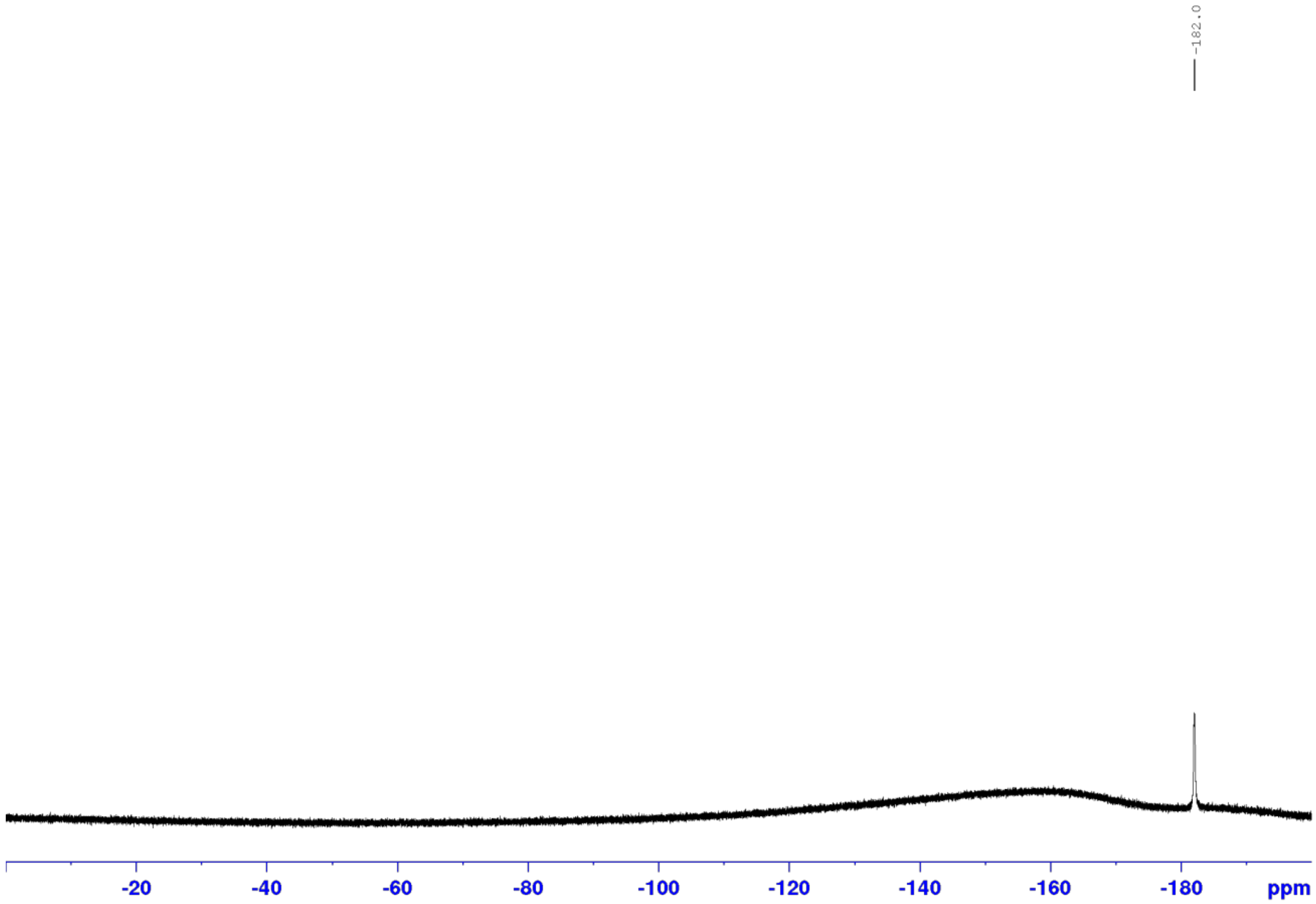

-182.0
-20
-40
-60
-80
-100
-120
-140
-160
-180
ppm

5-azido-3,3-dimethyl-1-propylpiperidine (21)

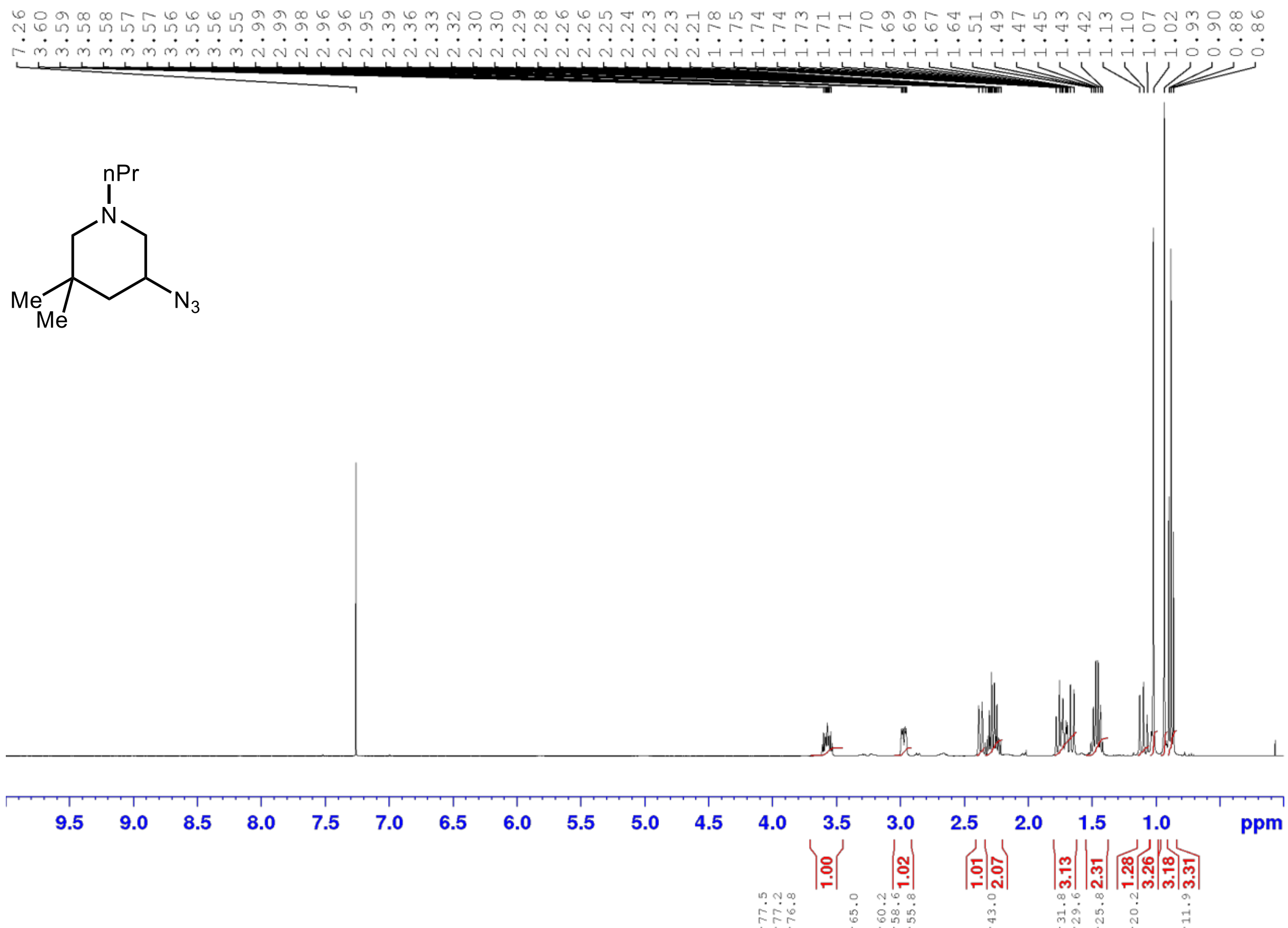

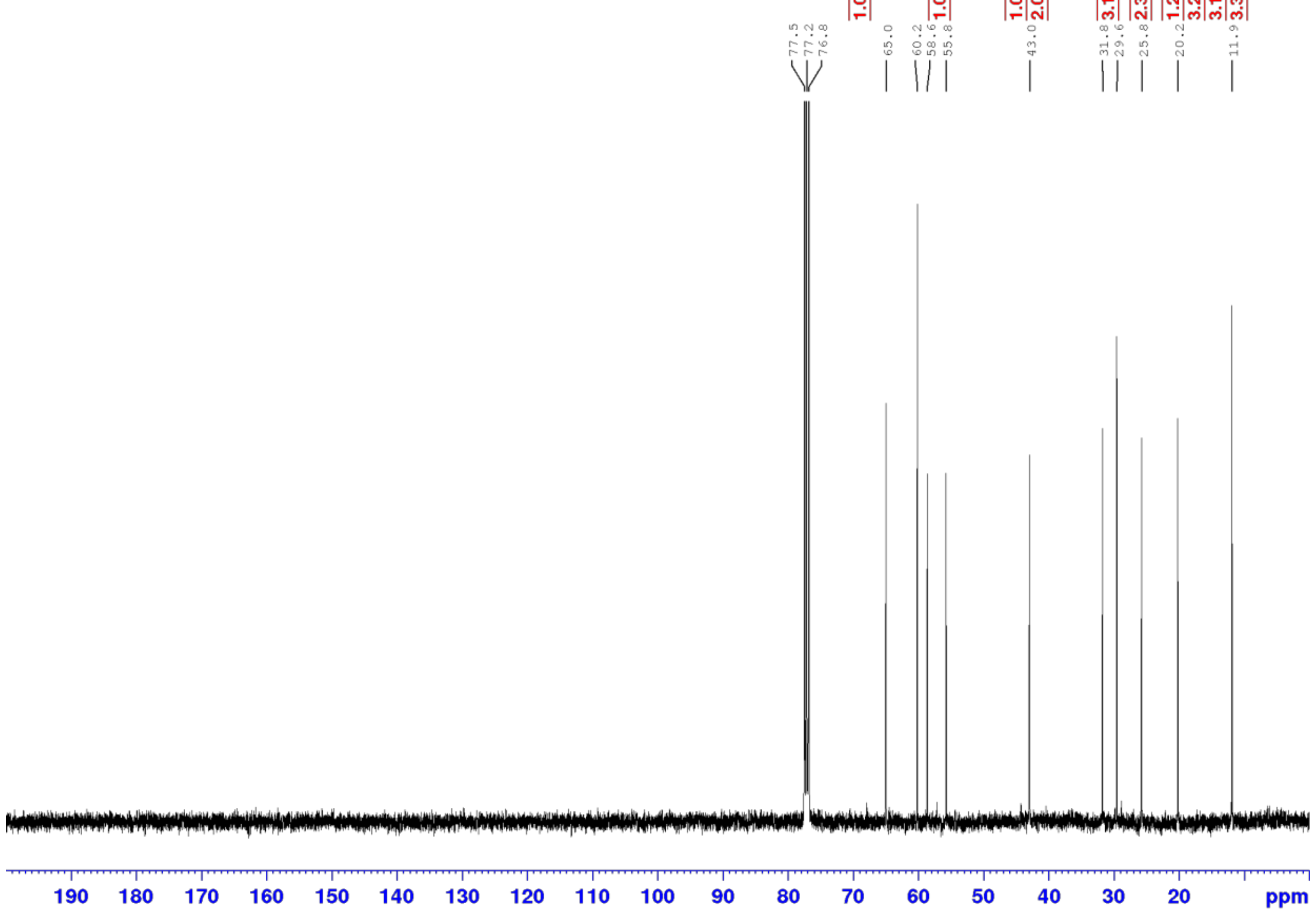

3,3-dimethyl-5-(4-phenyl-1H-1,2,3-triazol-1-yl)-1-propylpiperidine (22)

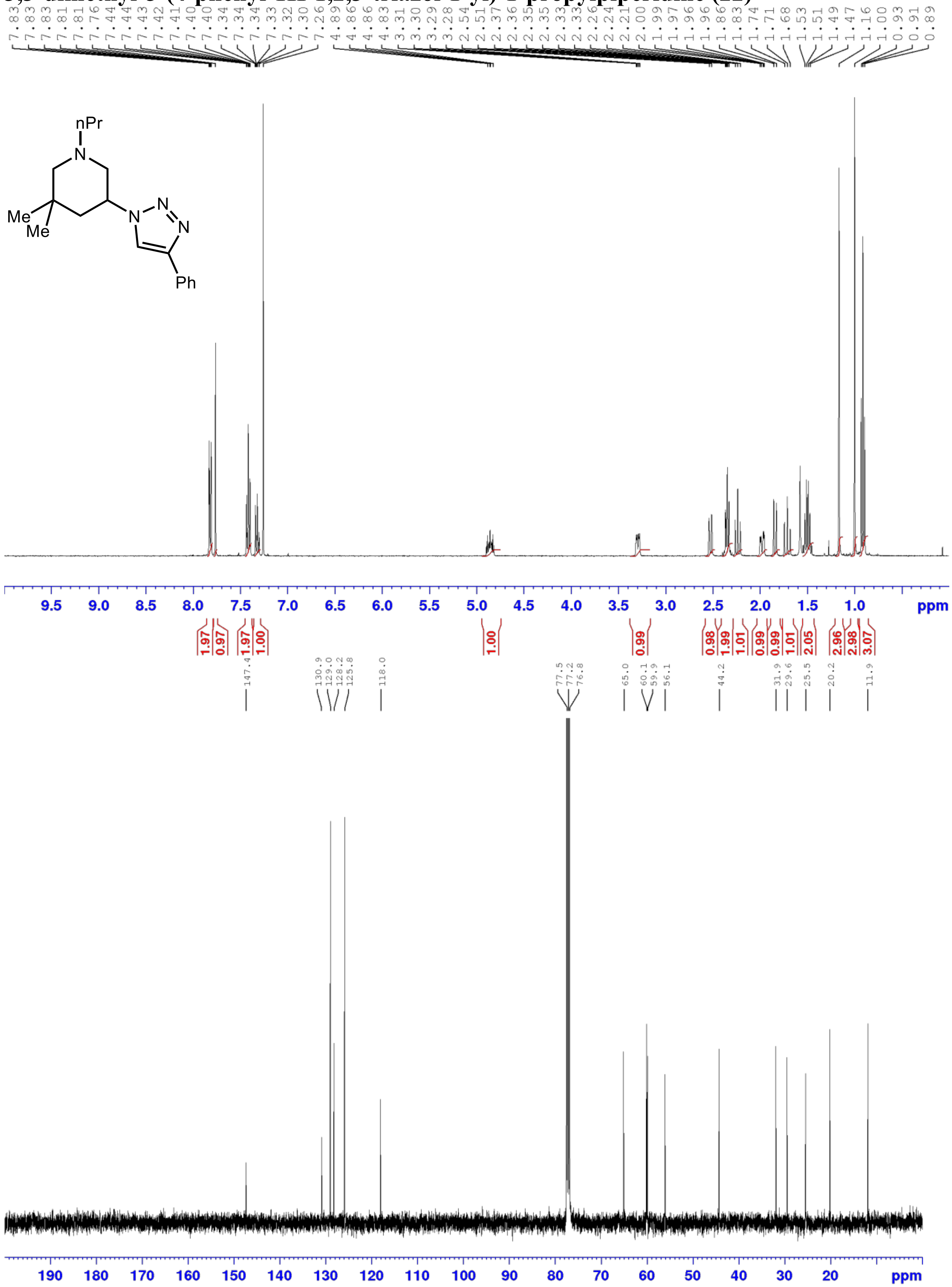

***N*-isobutyl-5,5-dimethyl-1-propylpiperidin-3-amine (23)**

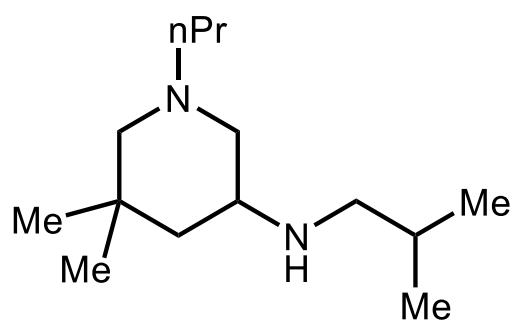

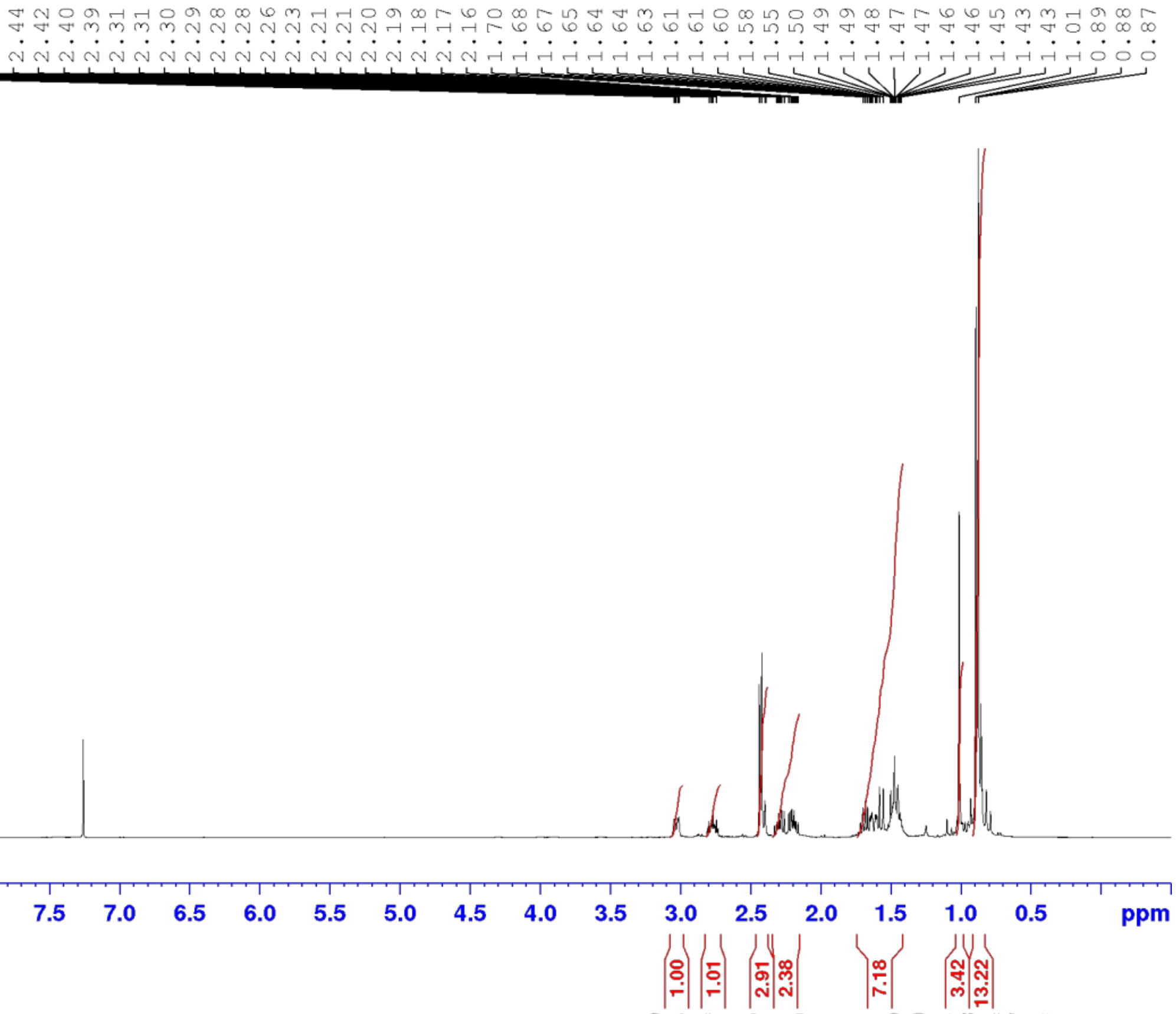

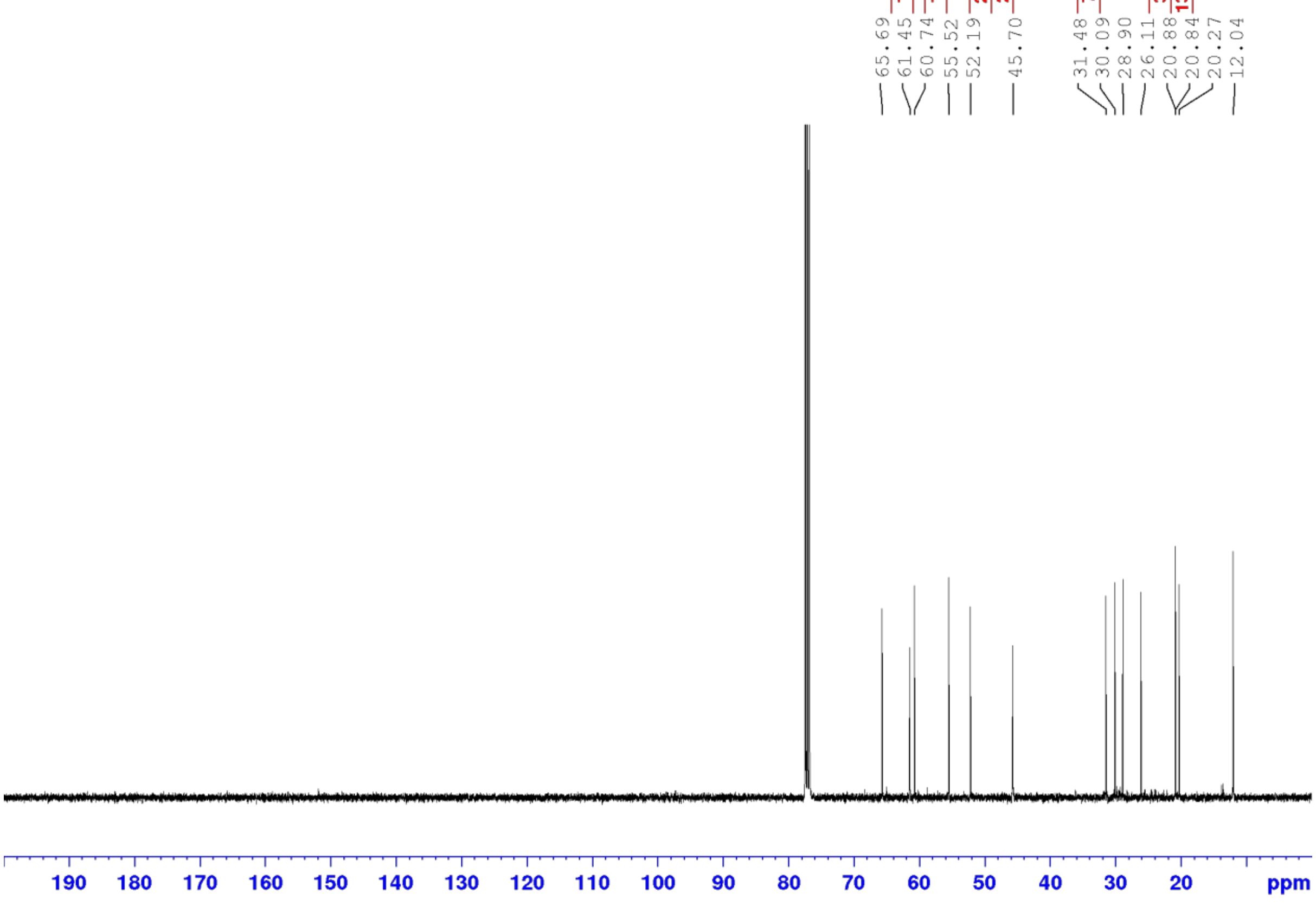

5,5-dimethyl-1-propylpiperidin-3-amine (24)

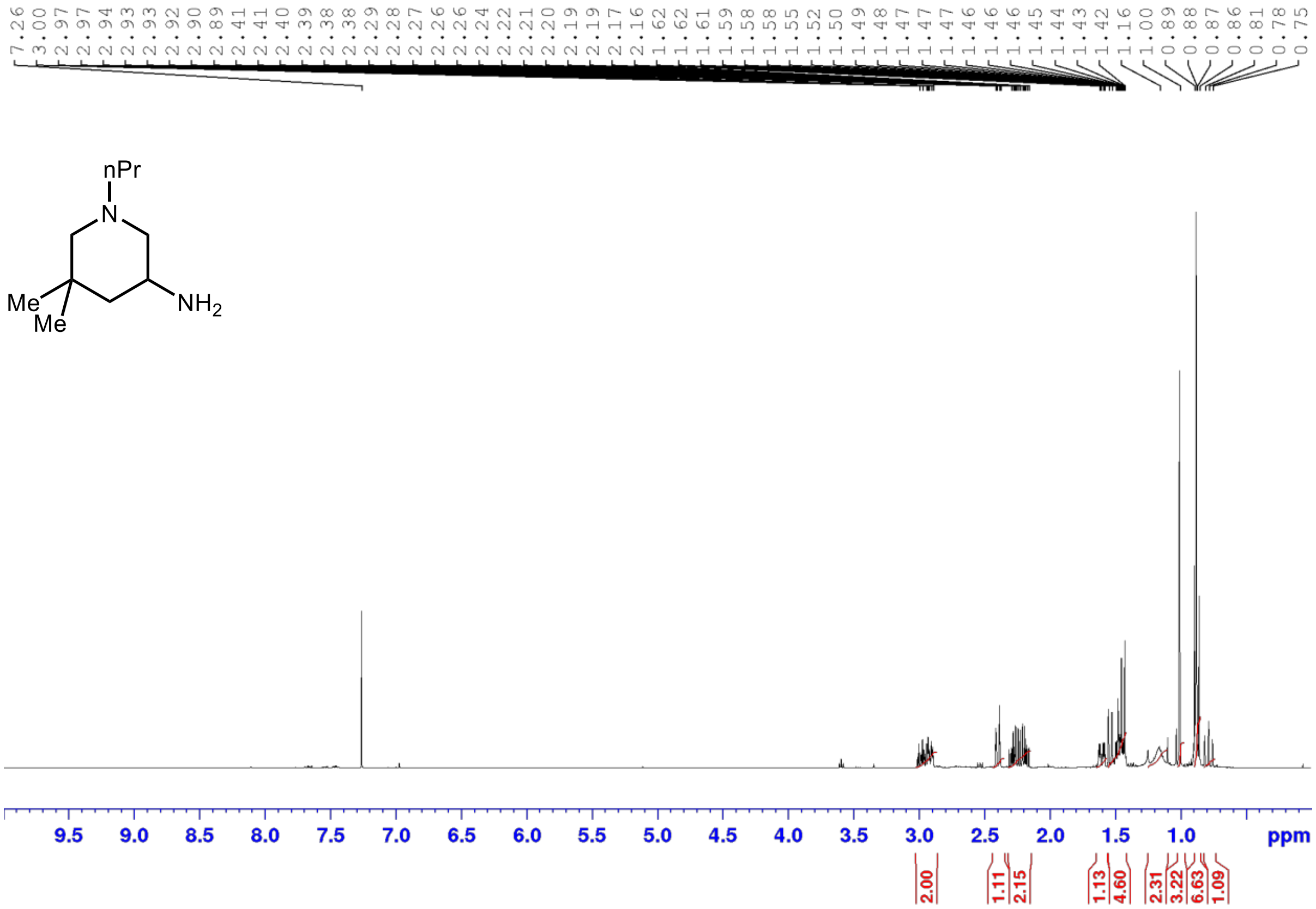

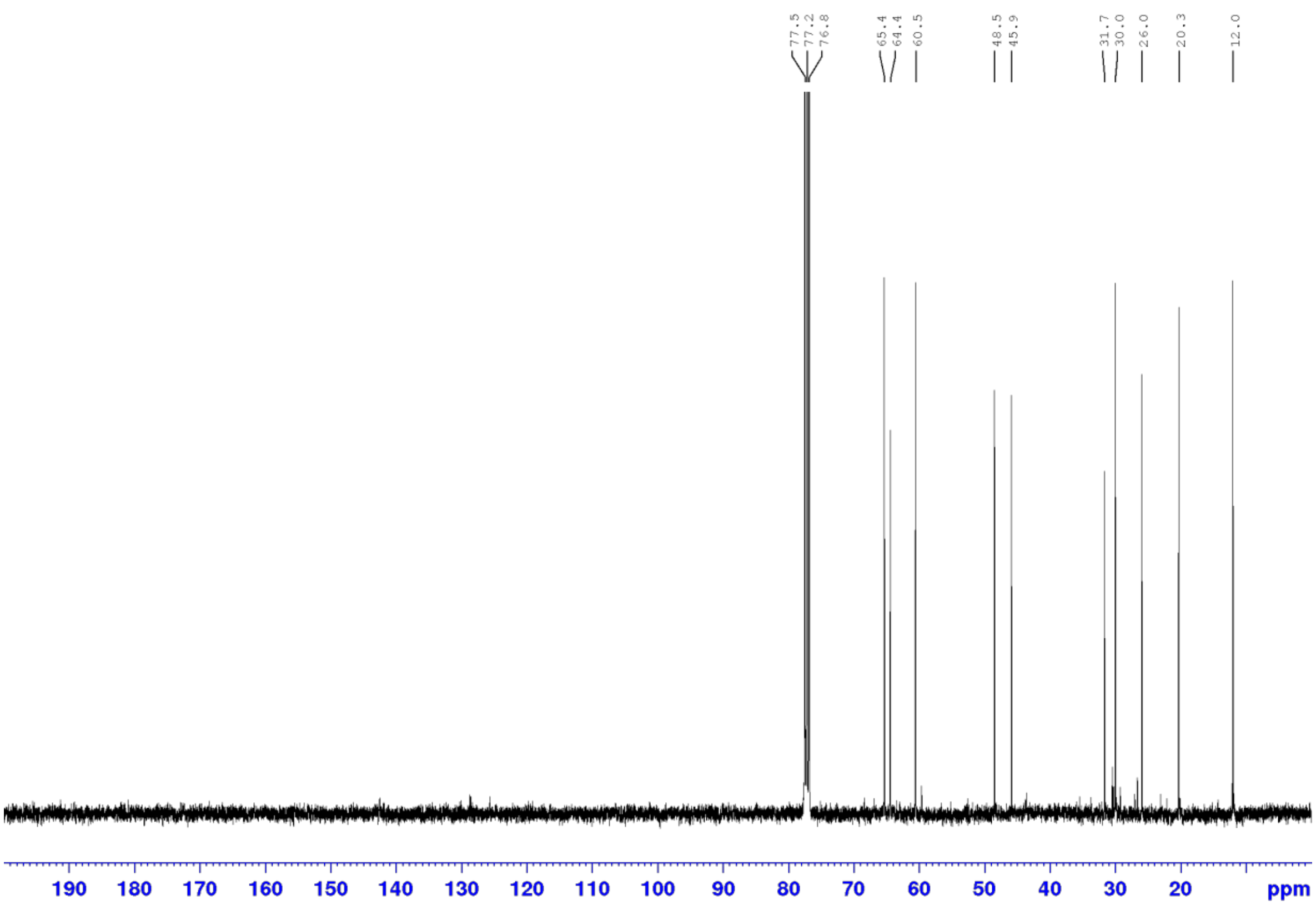

(E)-3,7-dimethyl-N-propyloct-4-en-1-aminium (25)

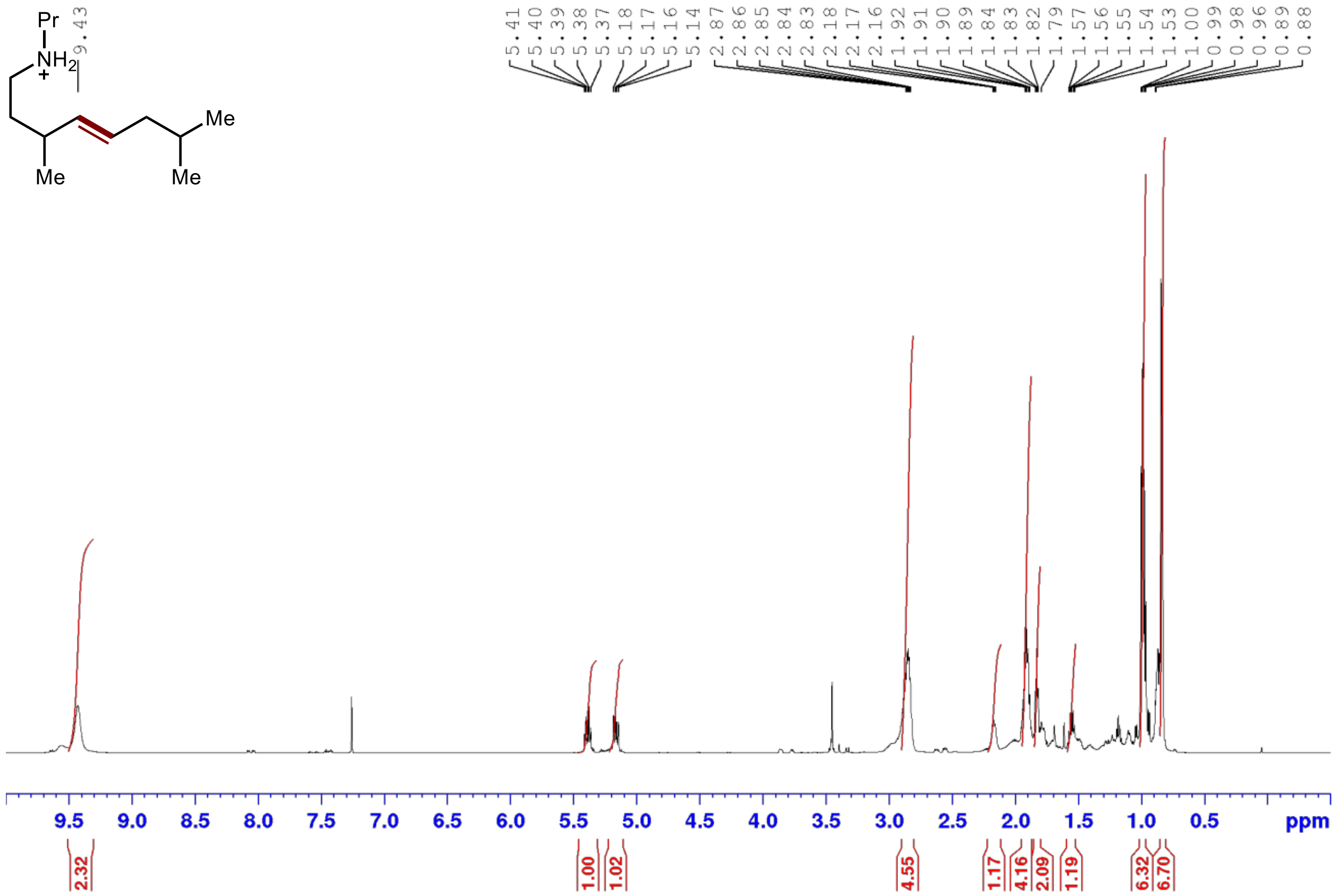

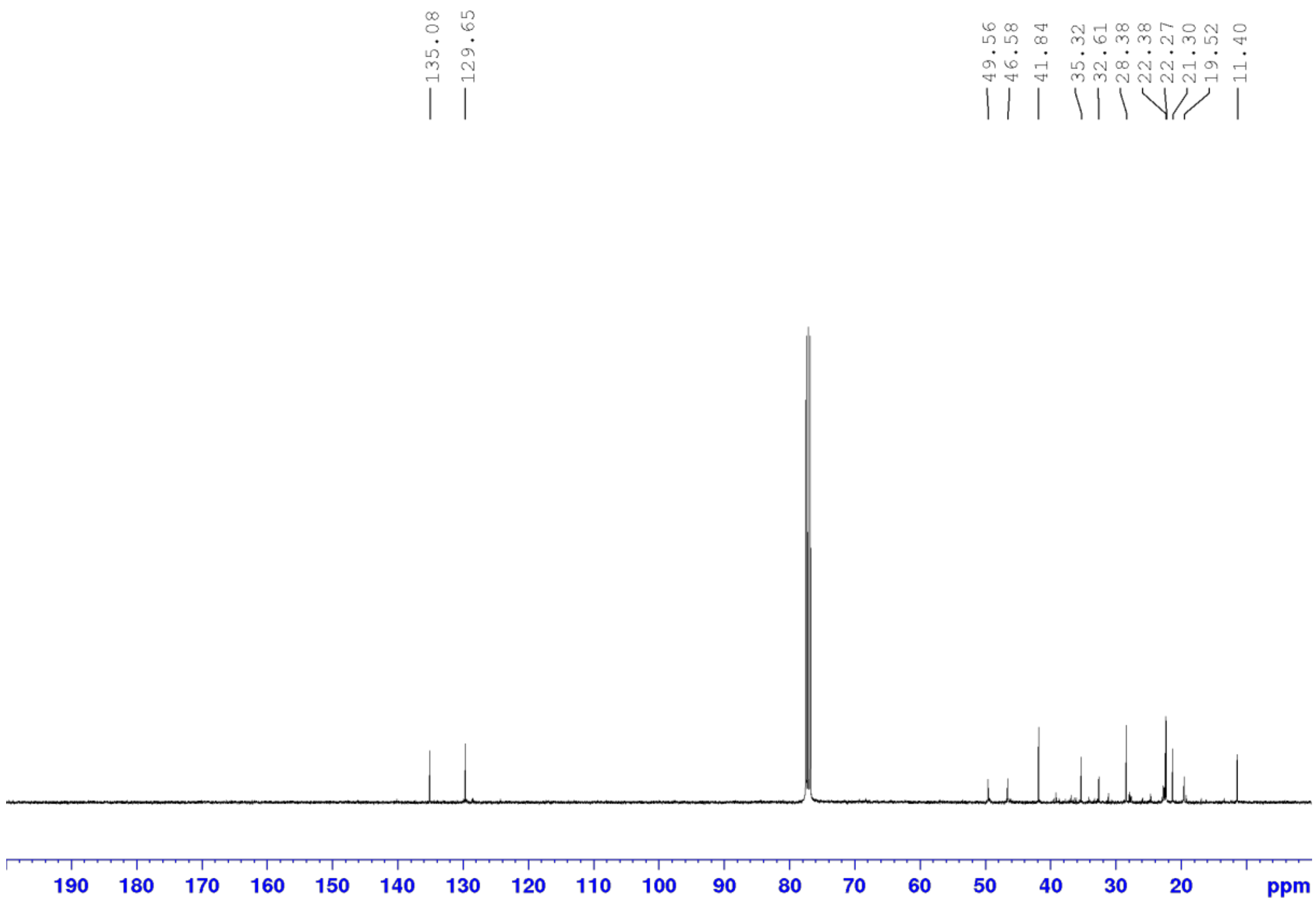

(*E*)-octa-1,6-dien-3-aminium (26)

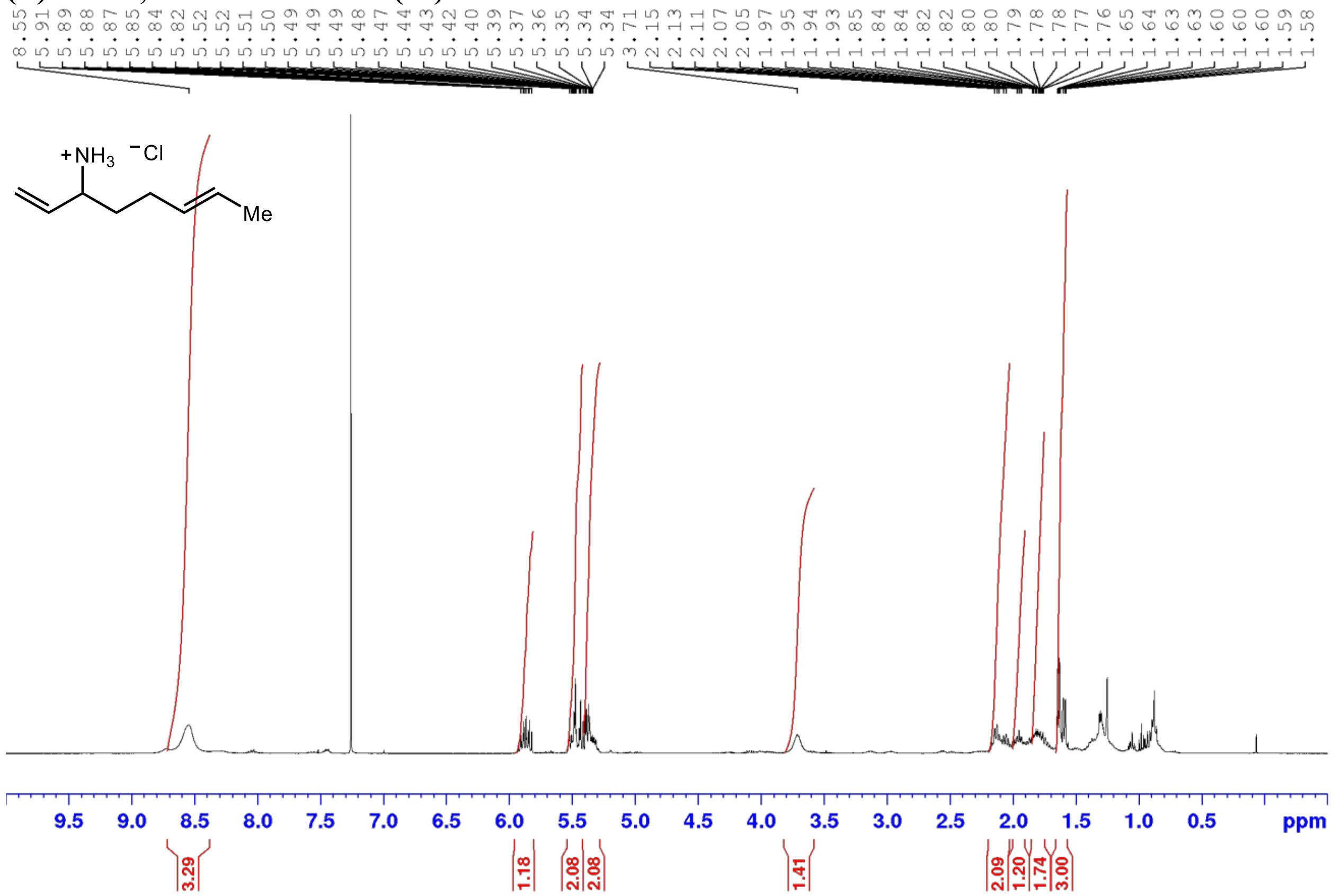

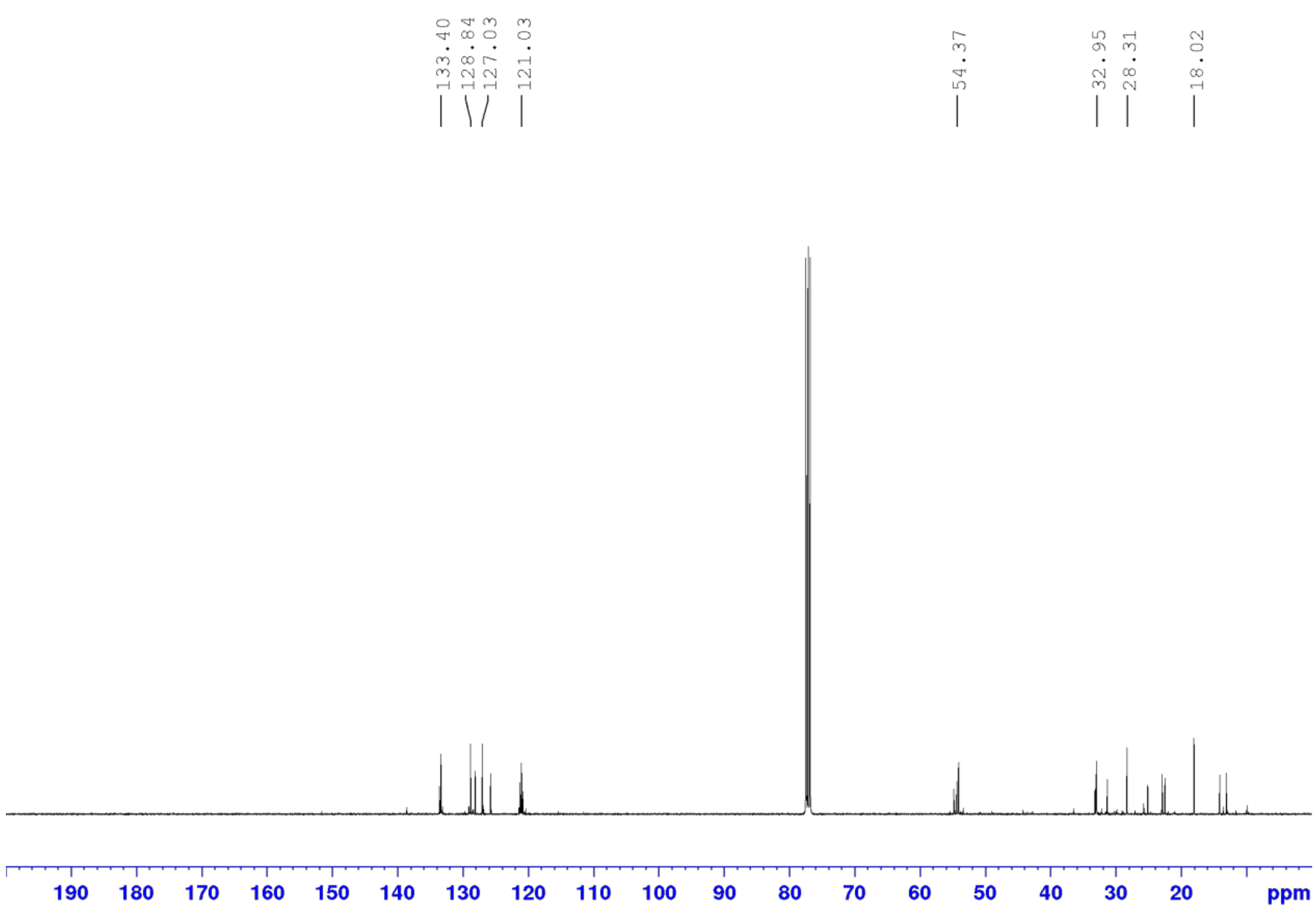

4-(Dipropylamino)-2-isopropyl-5-methylenecyclohexan-1-ol (29)

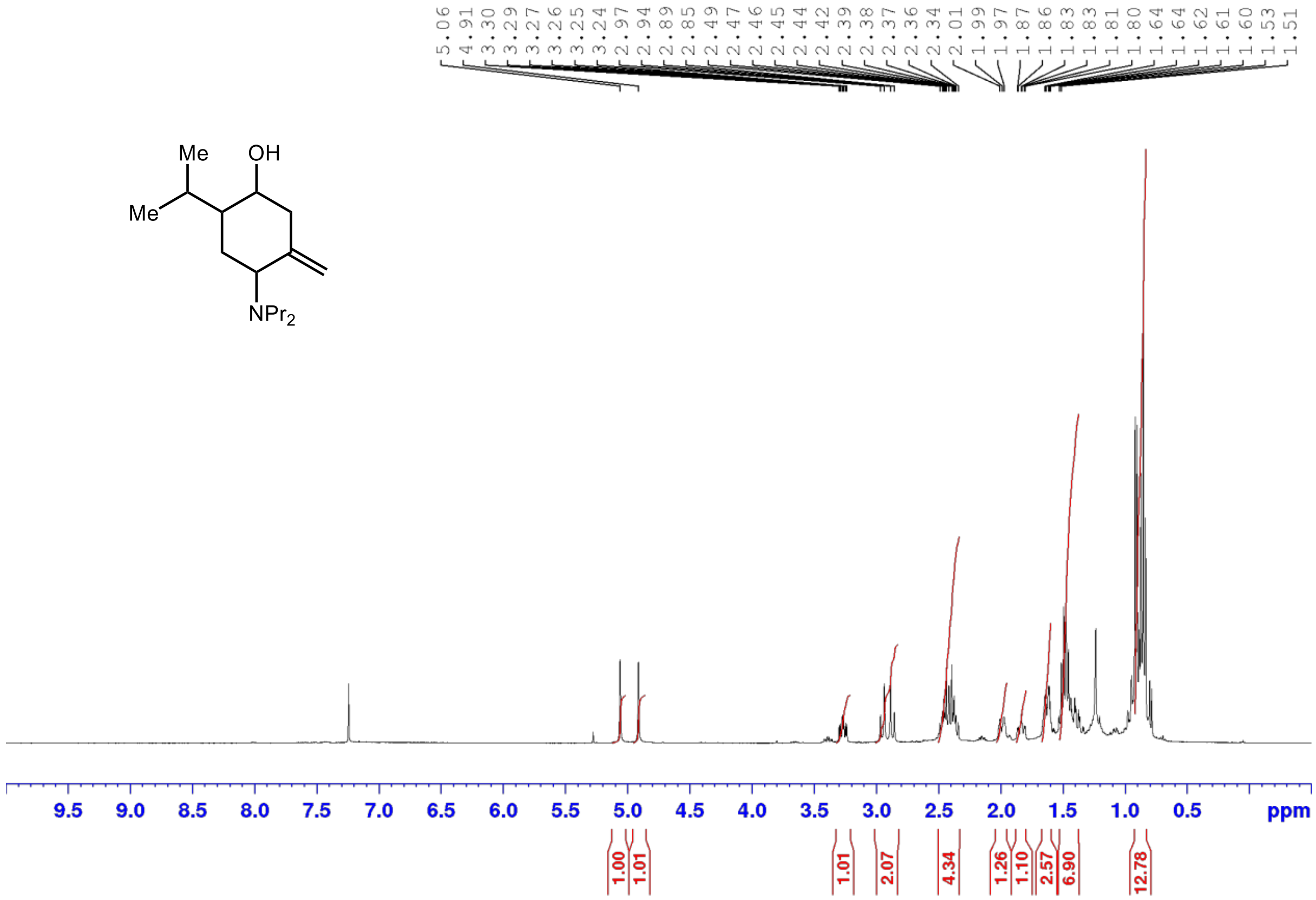

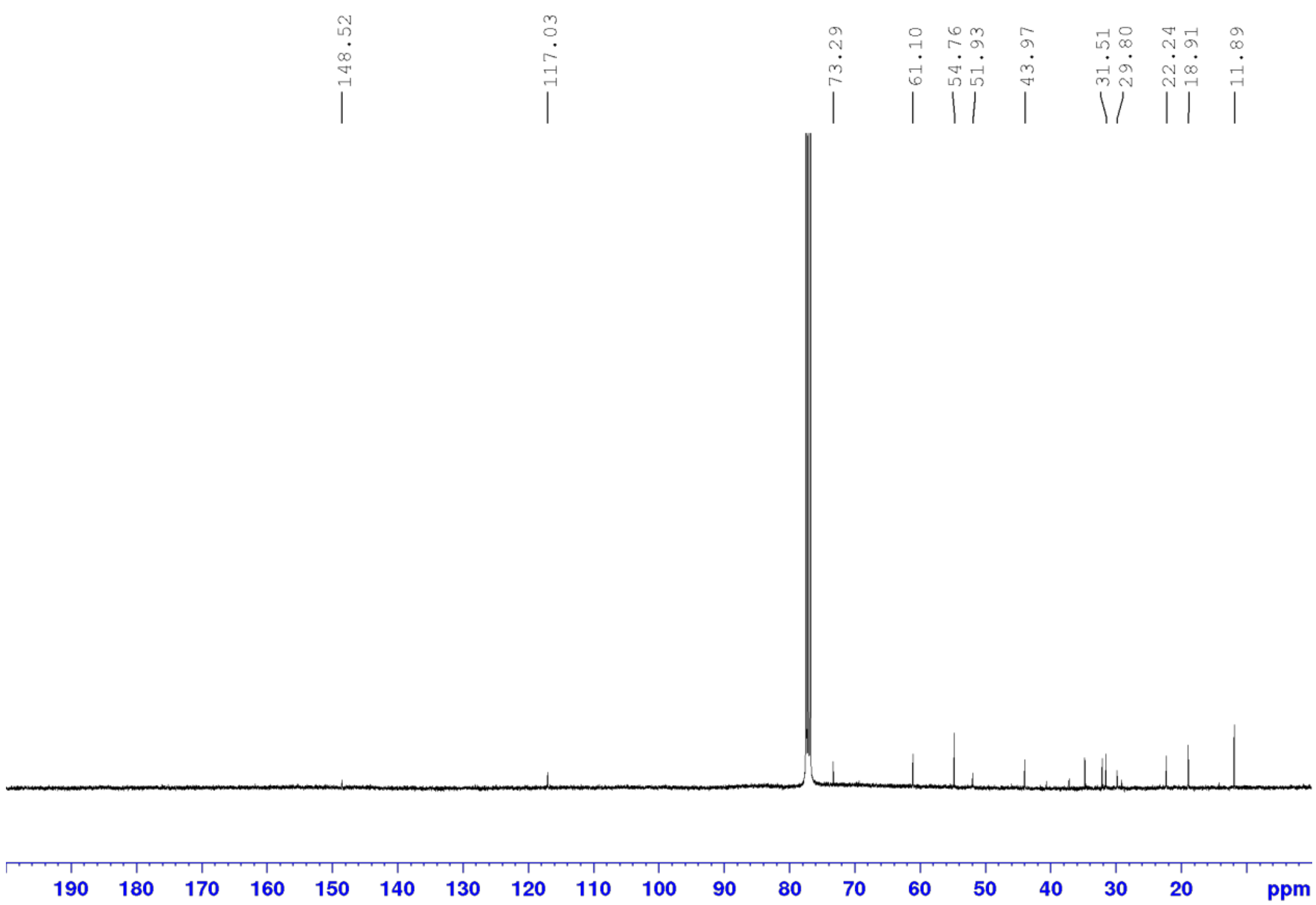

***N,N*-dipropylcyclohex-2-en-1-amine (30)**

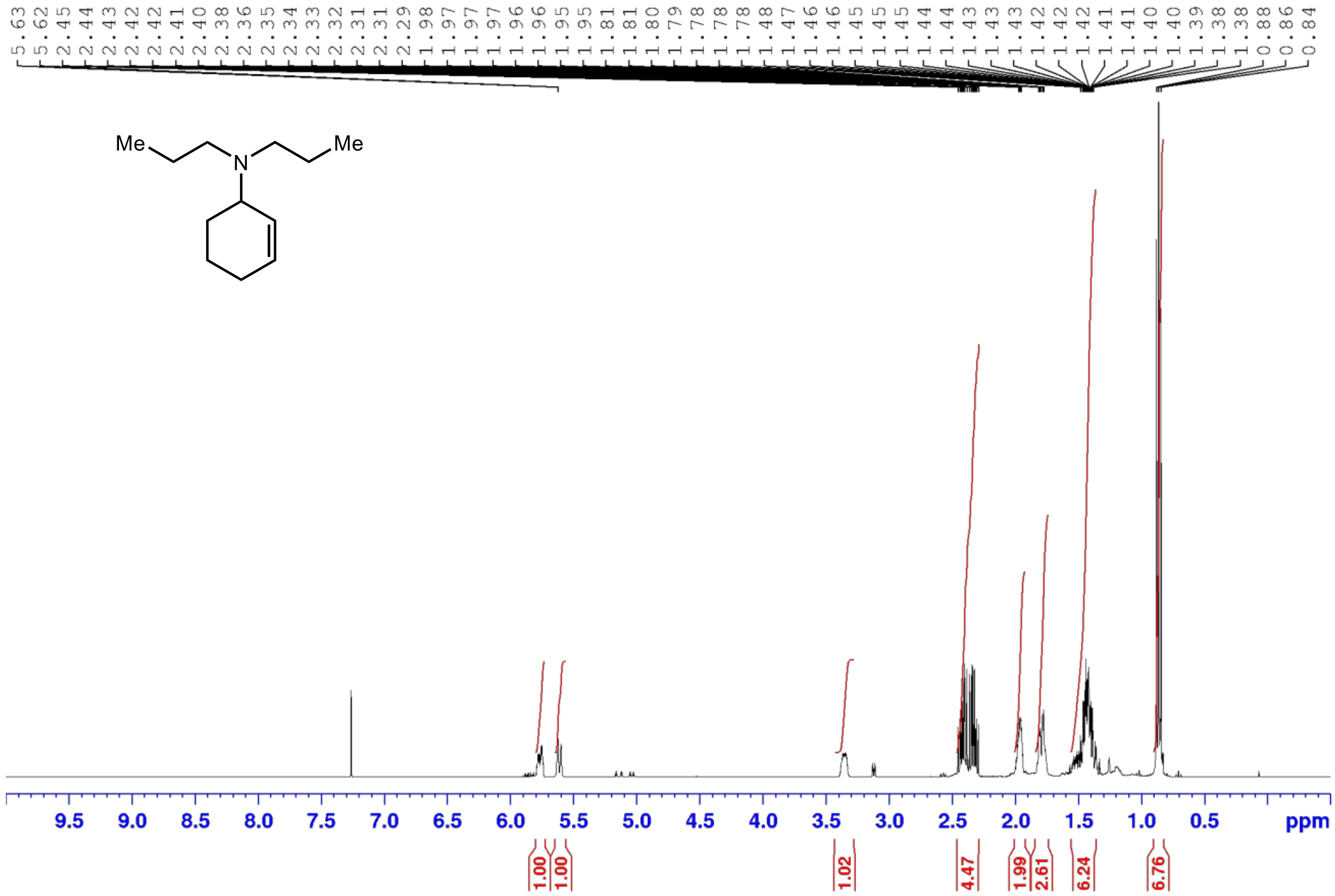

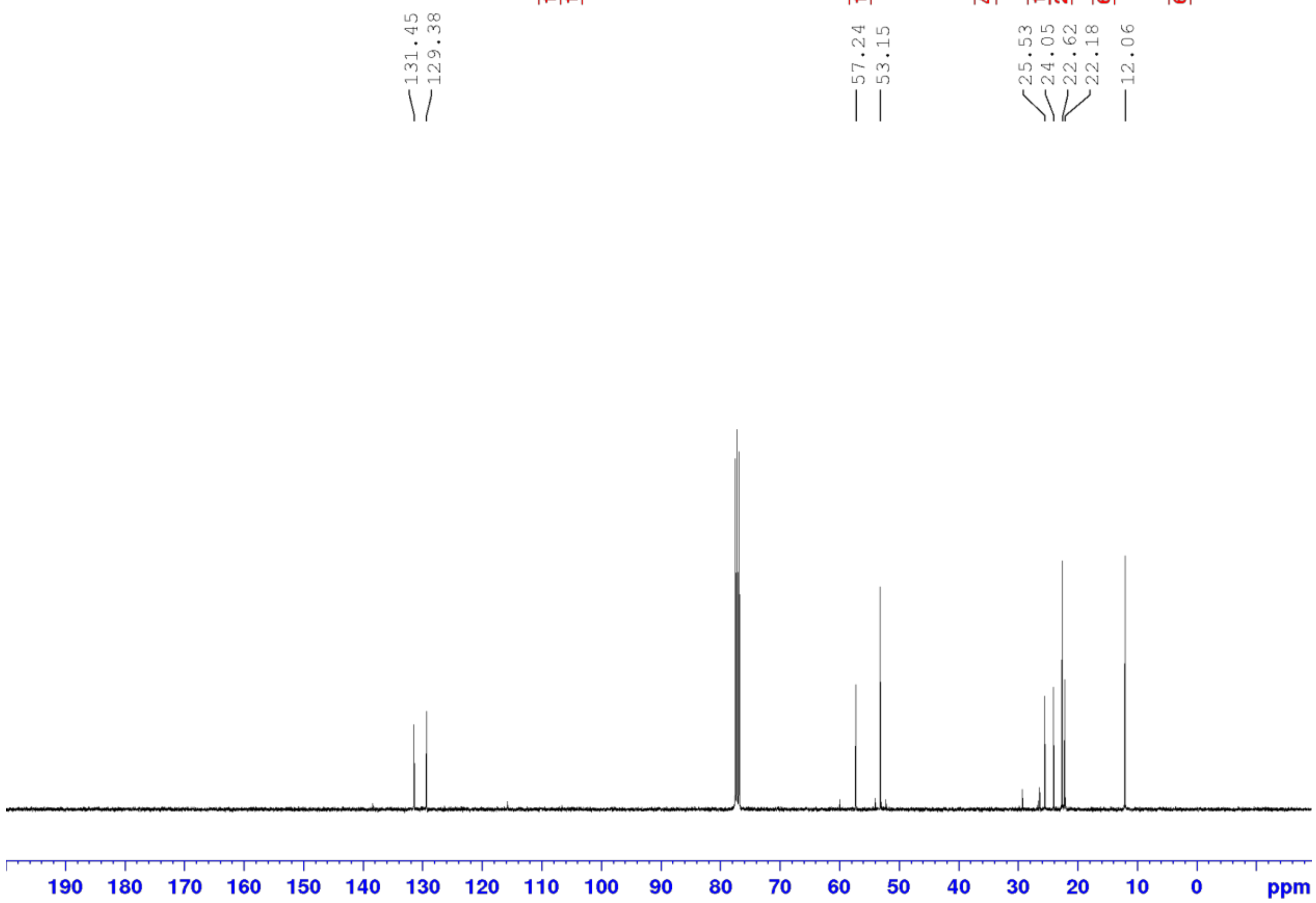

(E)-N-benzylhex-4-en-1-aminium (31)

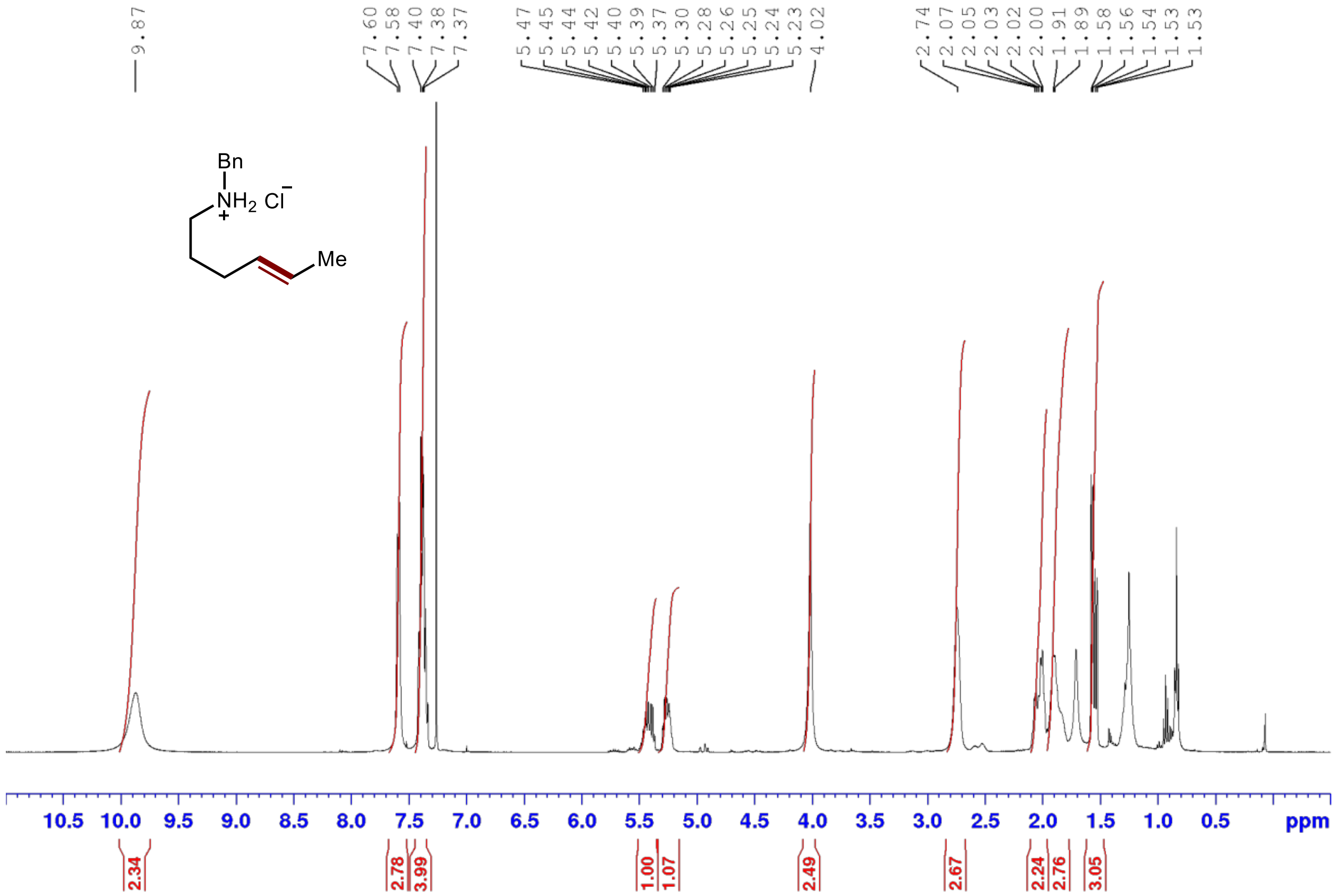

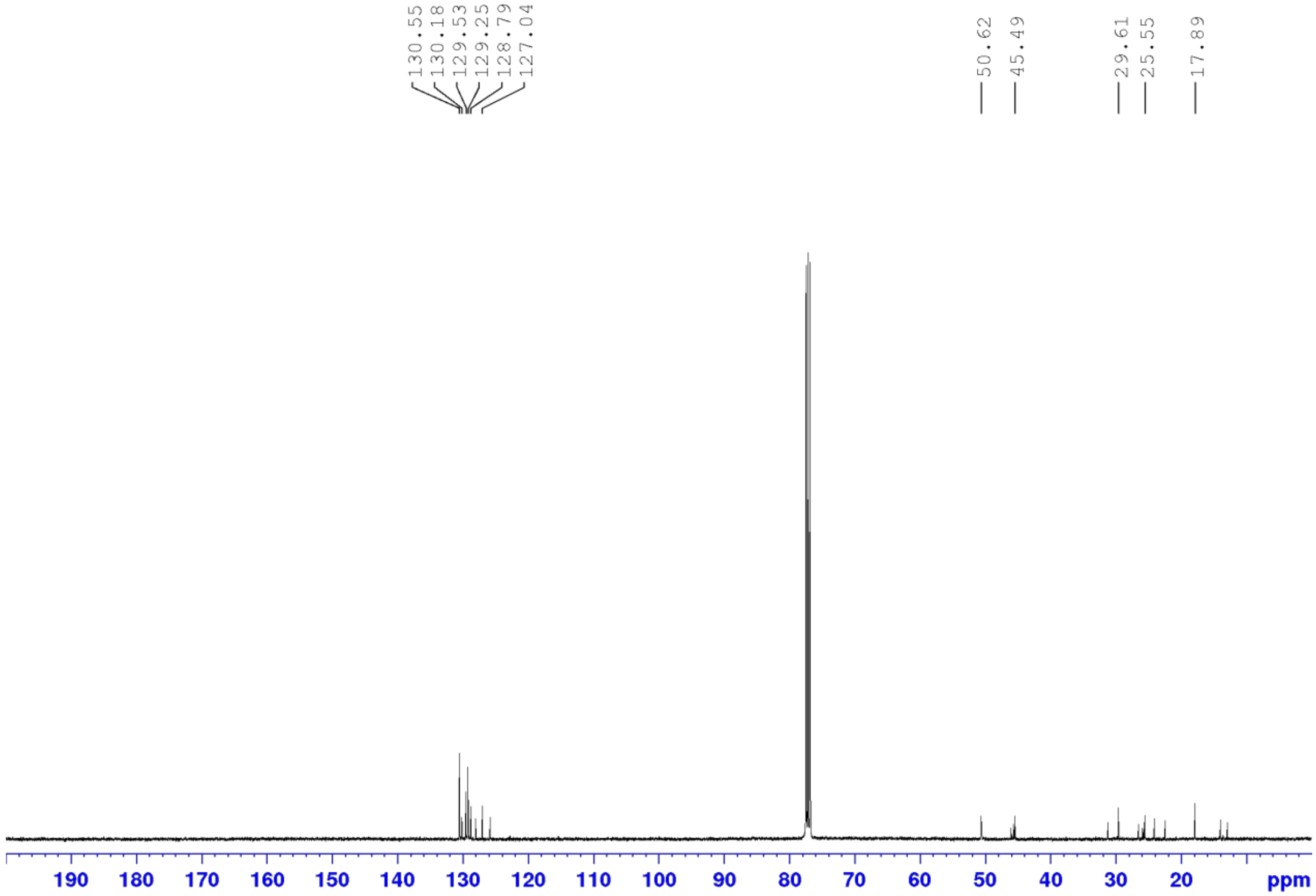

N-(2-cyanoethyl)pent-4-en-1-aminium (32)

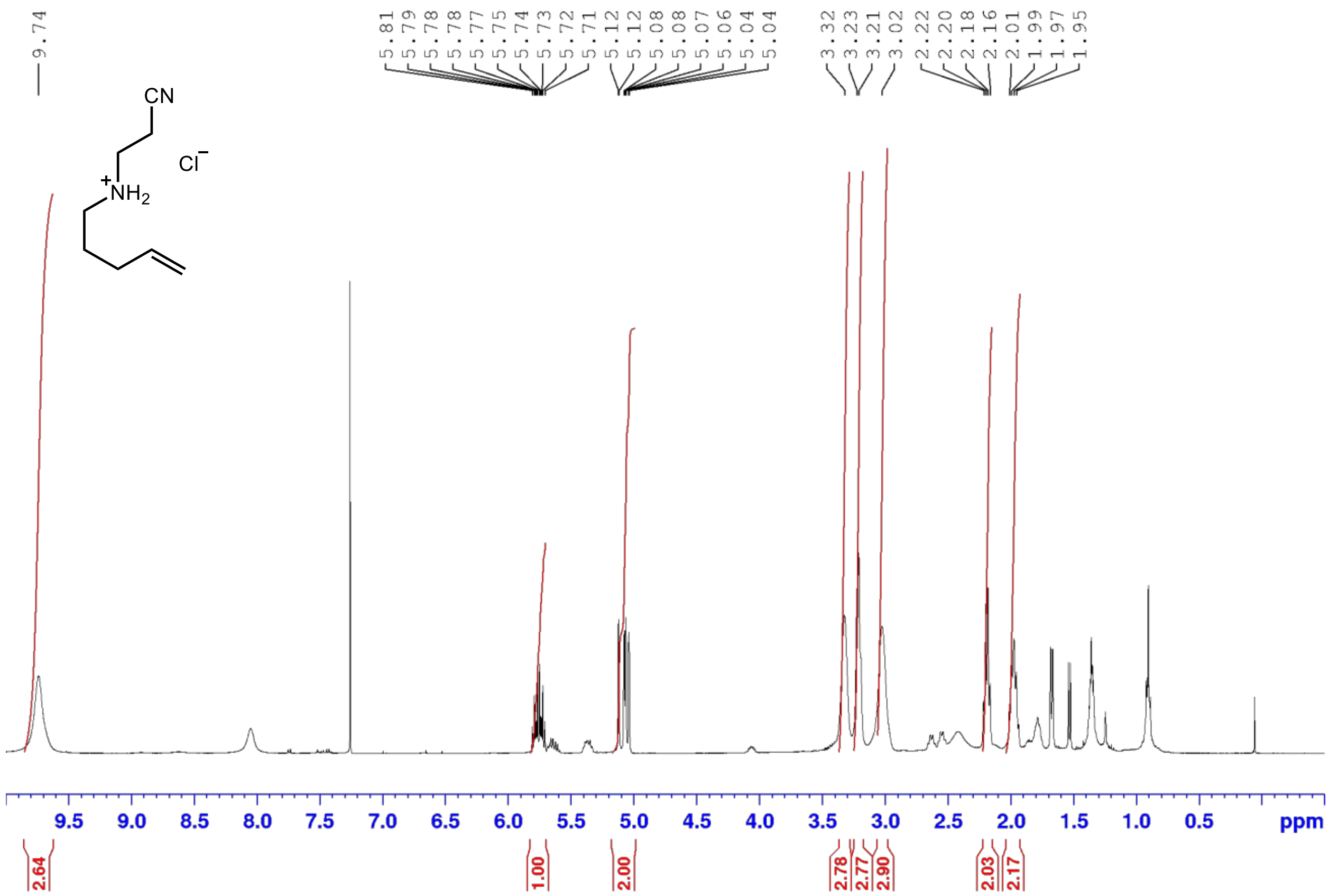

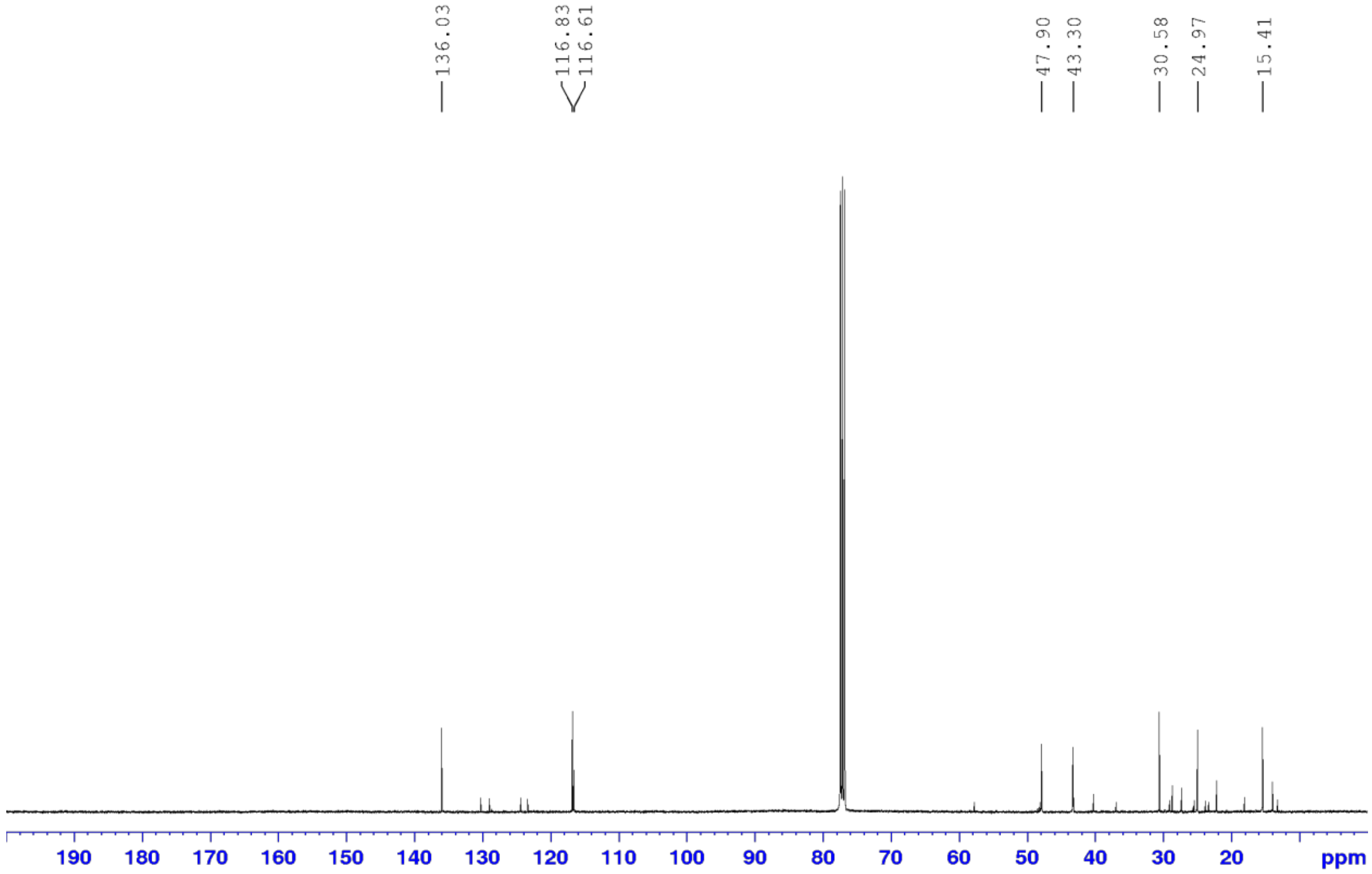

(E)-N-(3-hydroxypropyl)hex-4-en-1-aminium (33)

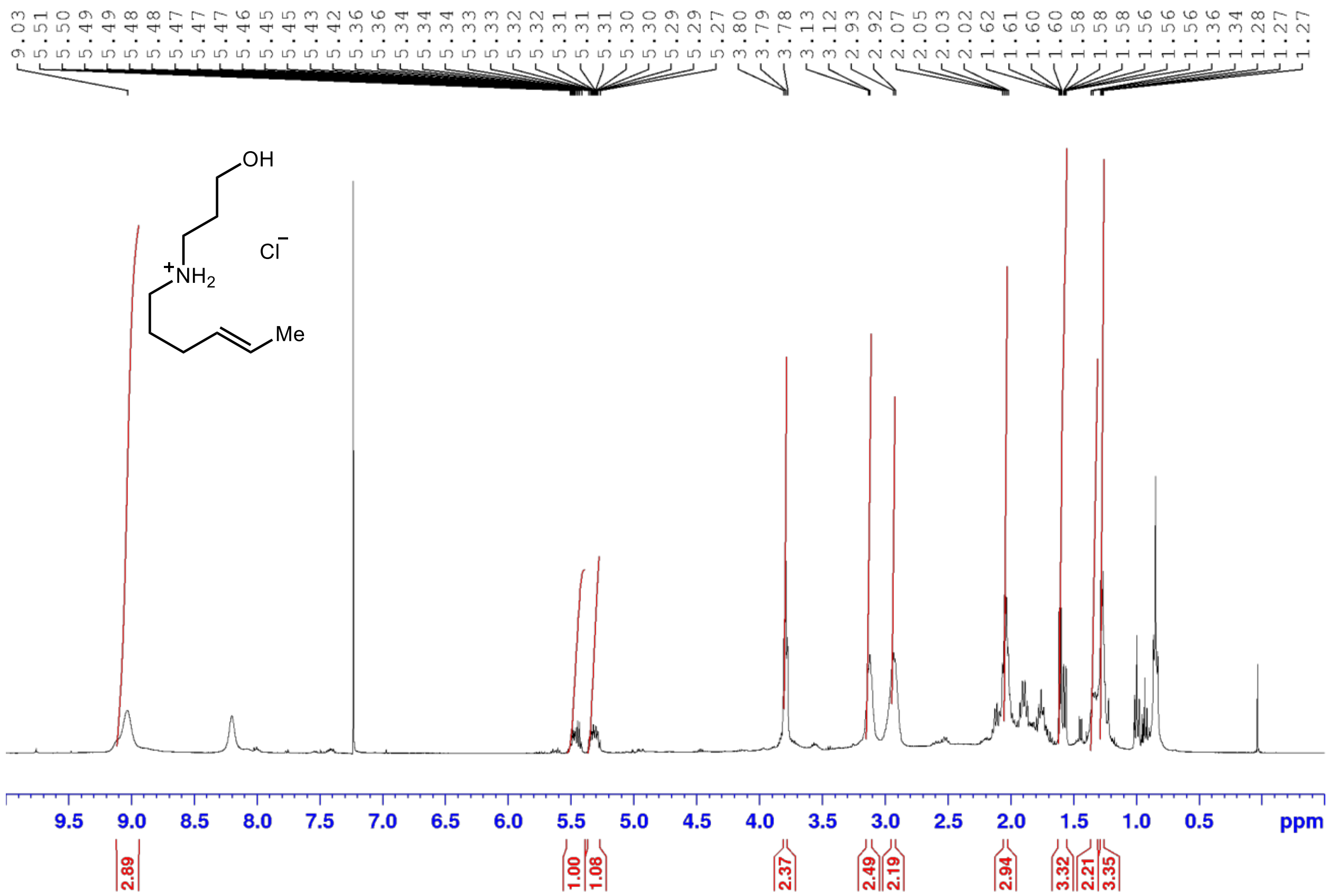

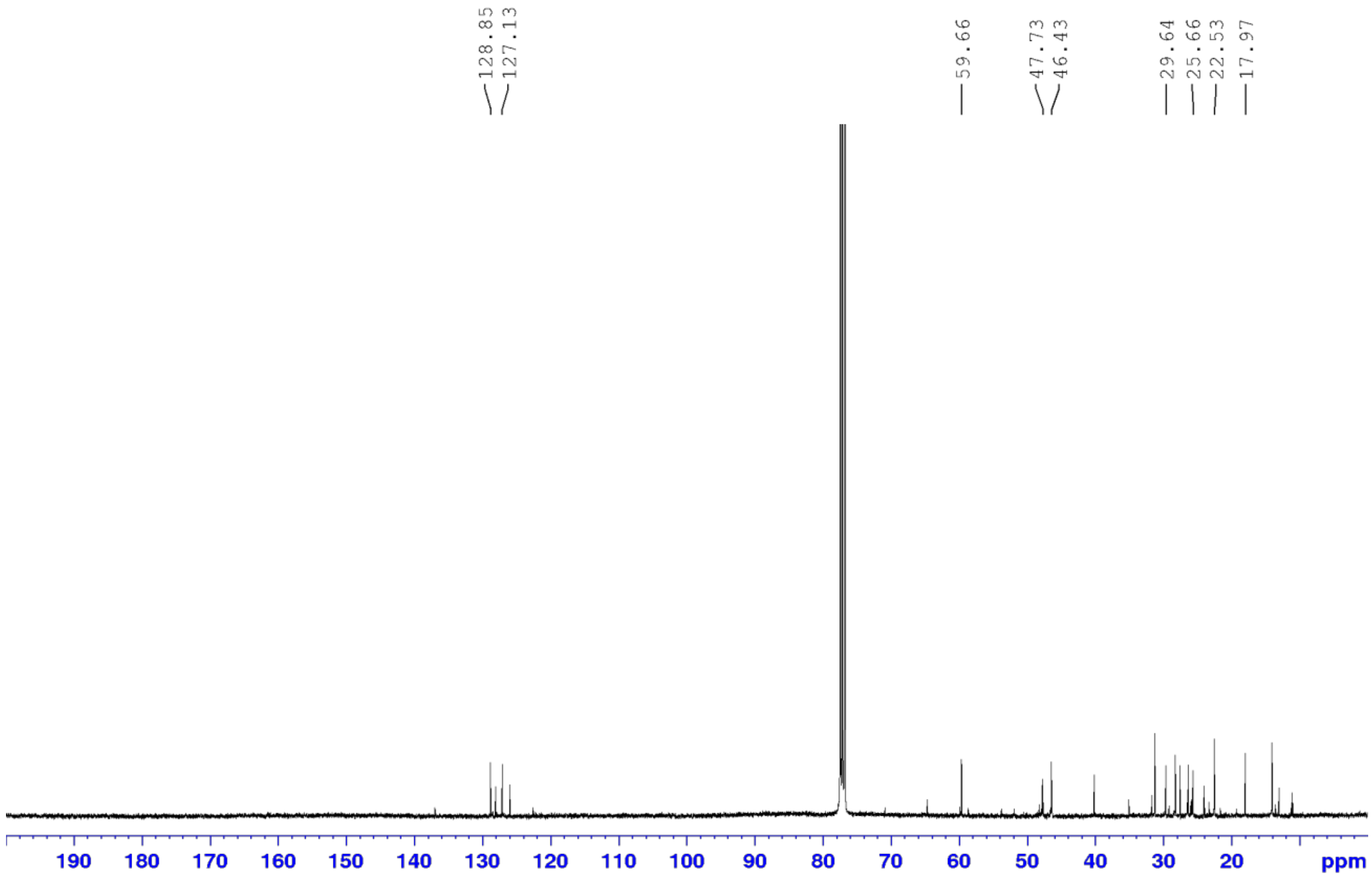

(E)-N-(3-(benzoyloxy)propyl)hex-4-en-1-aminium (34)

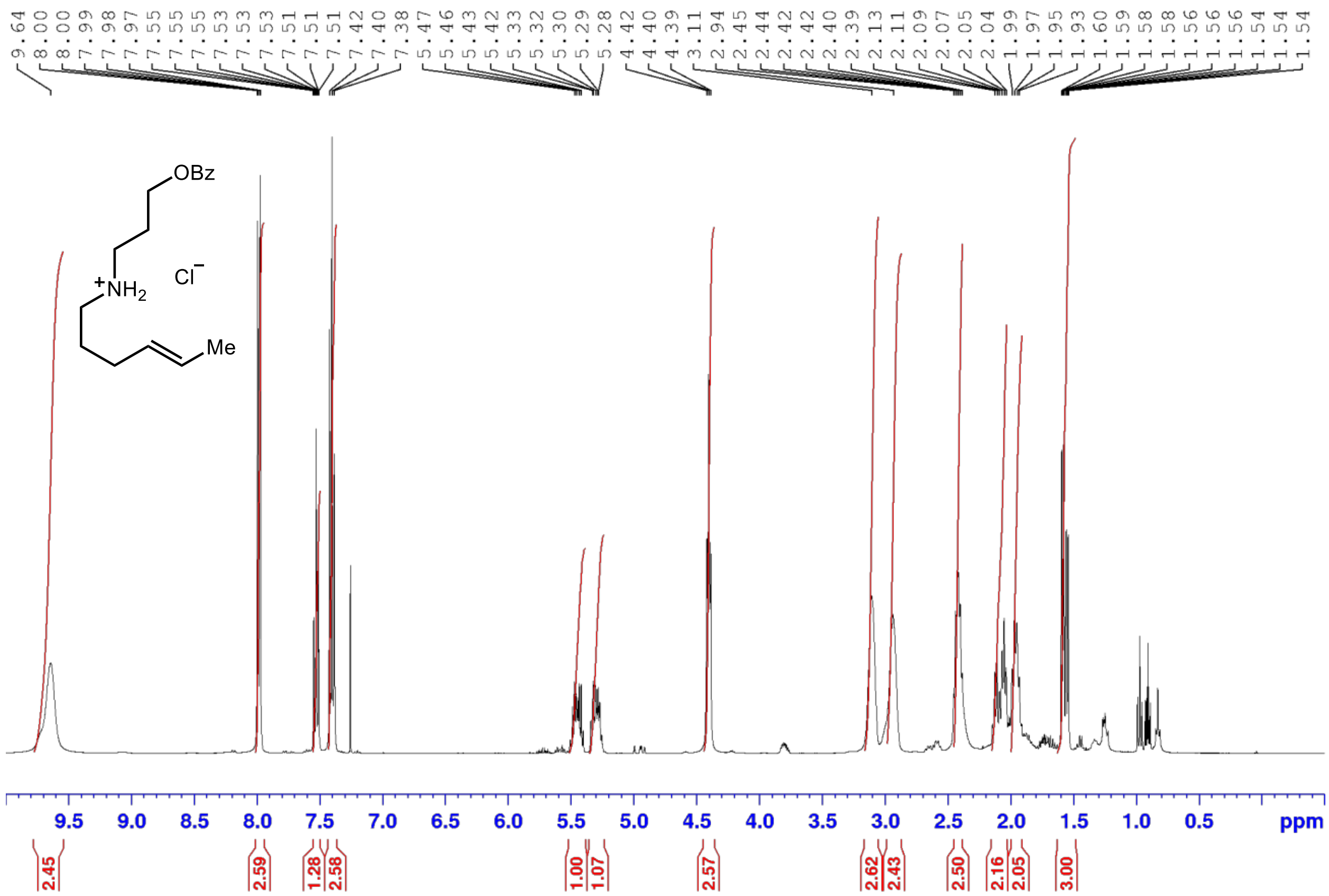

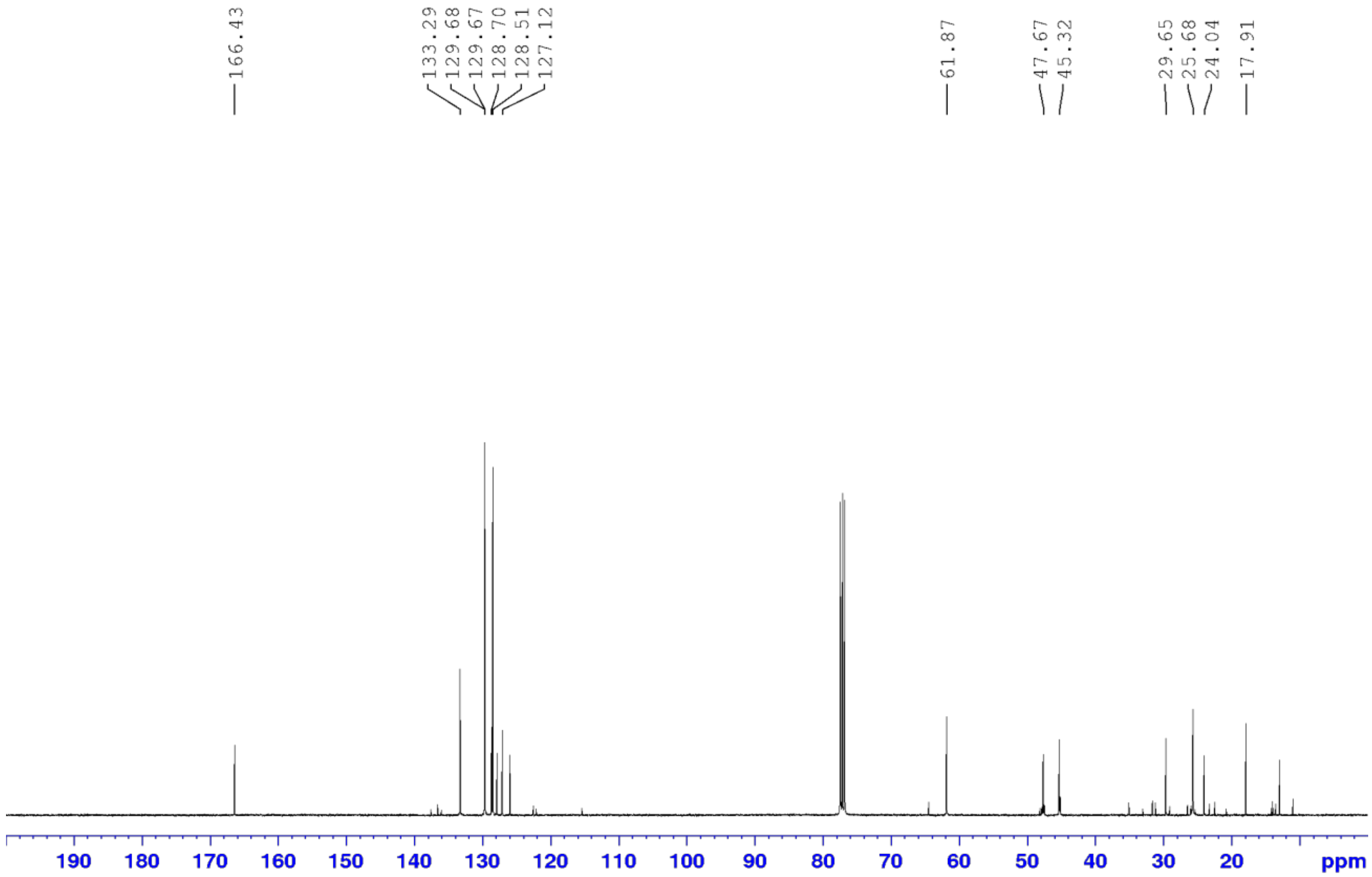

(E)-N-(3-((4-(trifluoromethyl)benzoyl)oxy)propyl)hex-4-en-1-aminium (35)

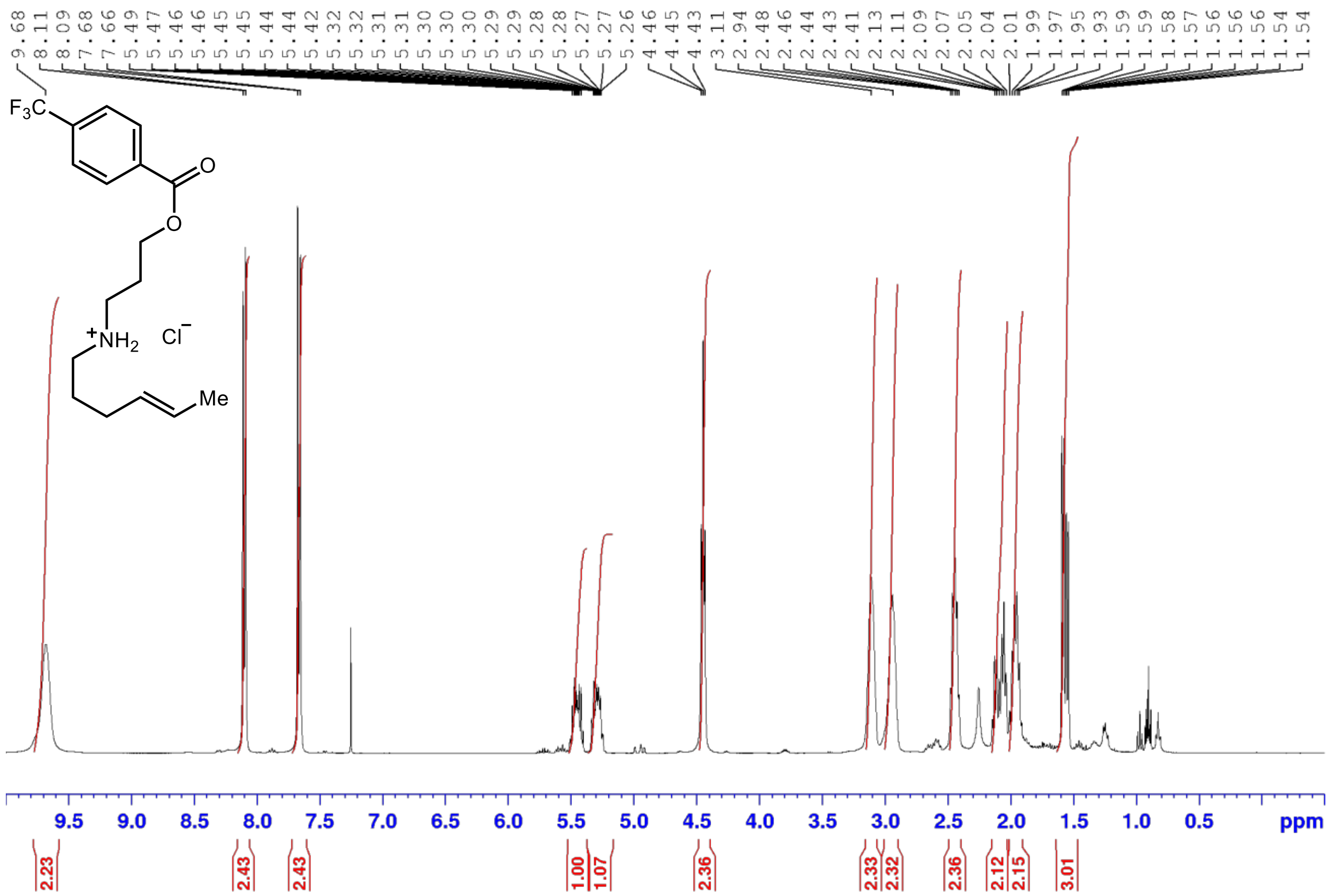

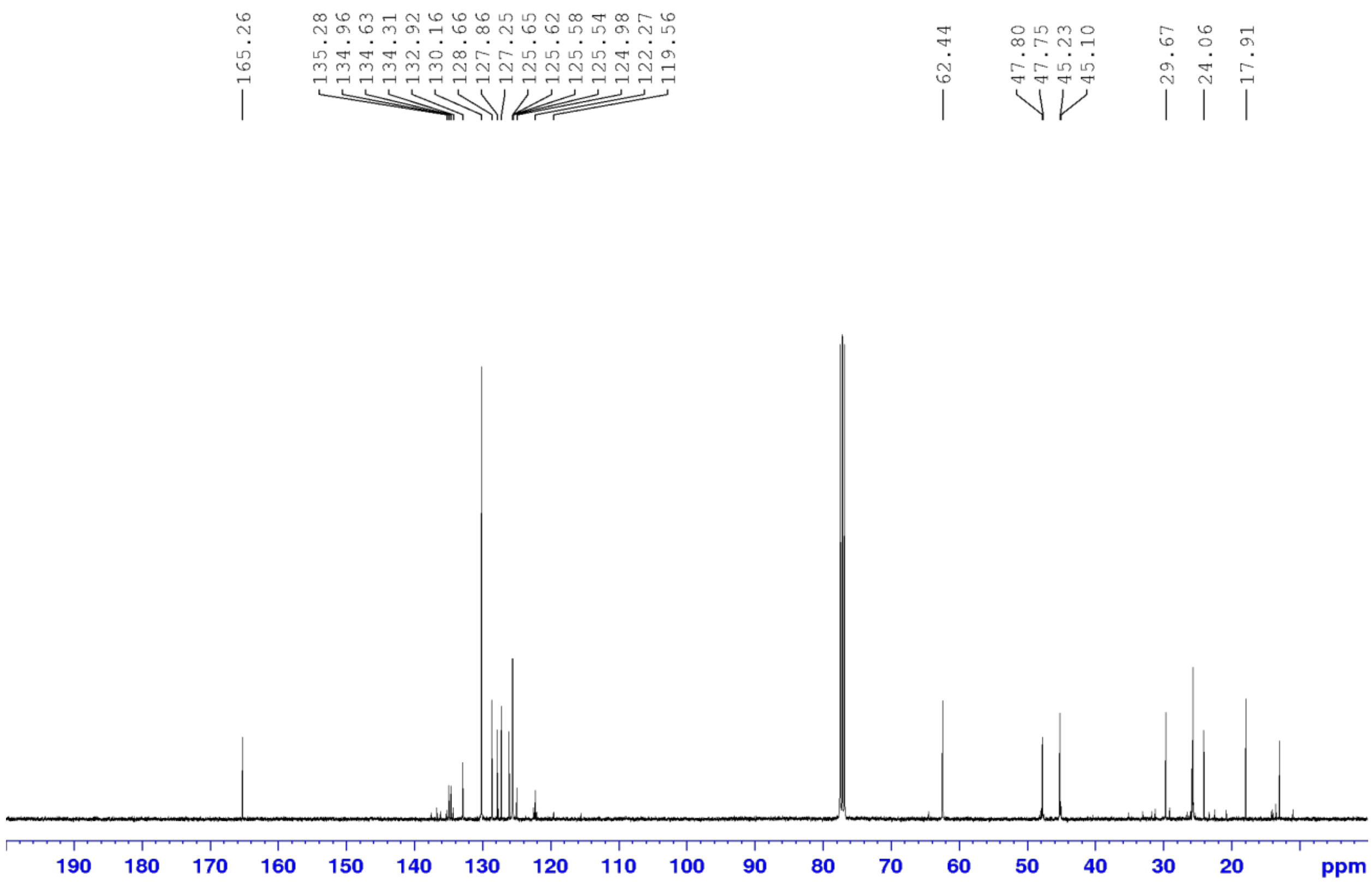

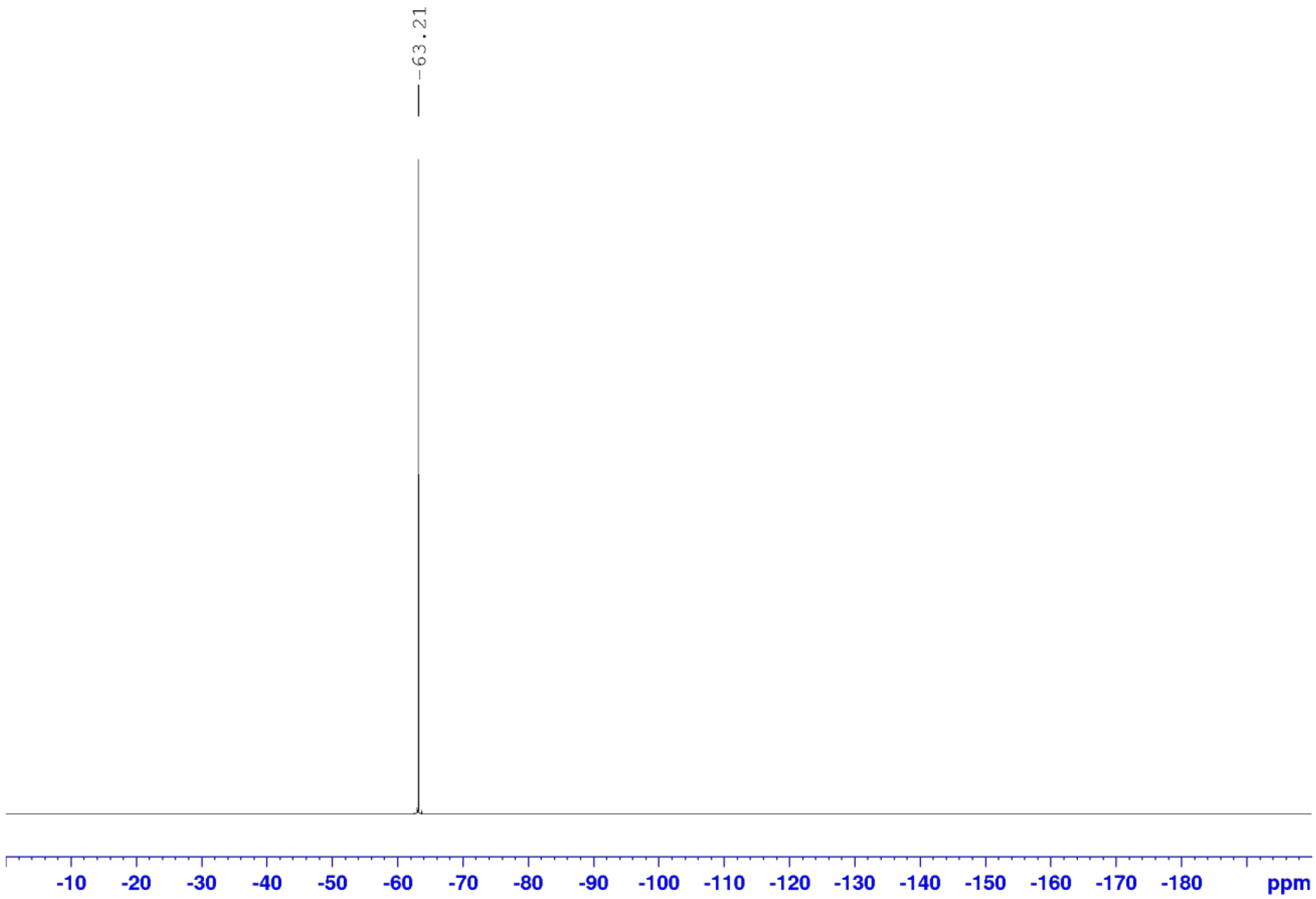
-63.21
-10
-20
-30
-40
-50
-60
-70
-80
-90
-100
-110
-120
-130
-140
-150
-160
-170
-180
ppm

(E)-N-(3-(2-(4-methoxyphenyl)acetoxy)propyl)hex-4-en-1-aminium (36)

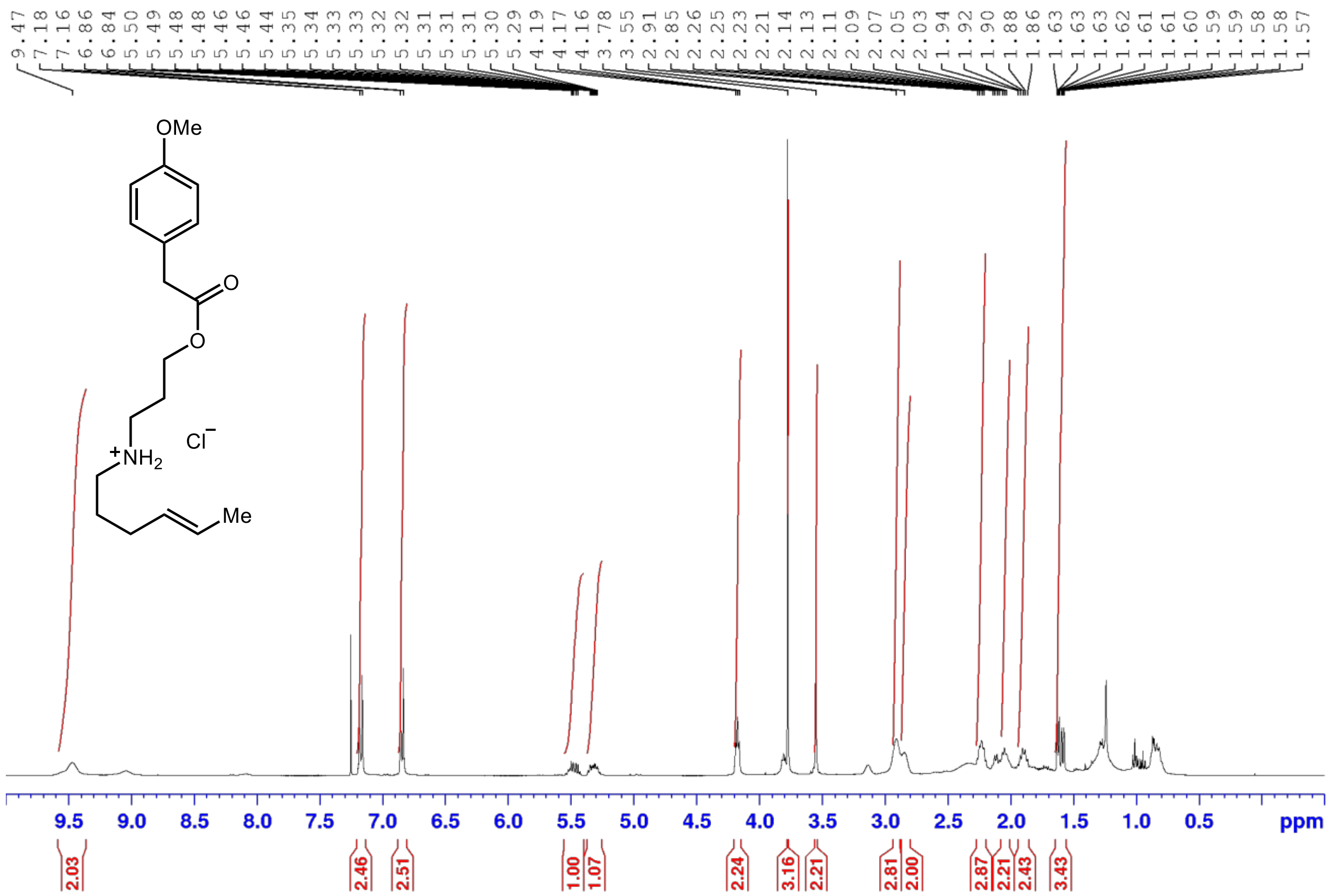

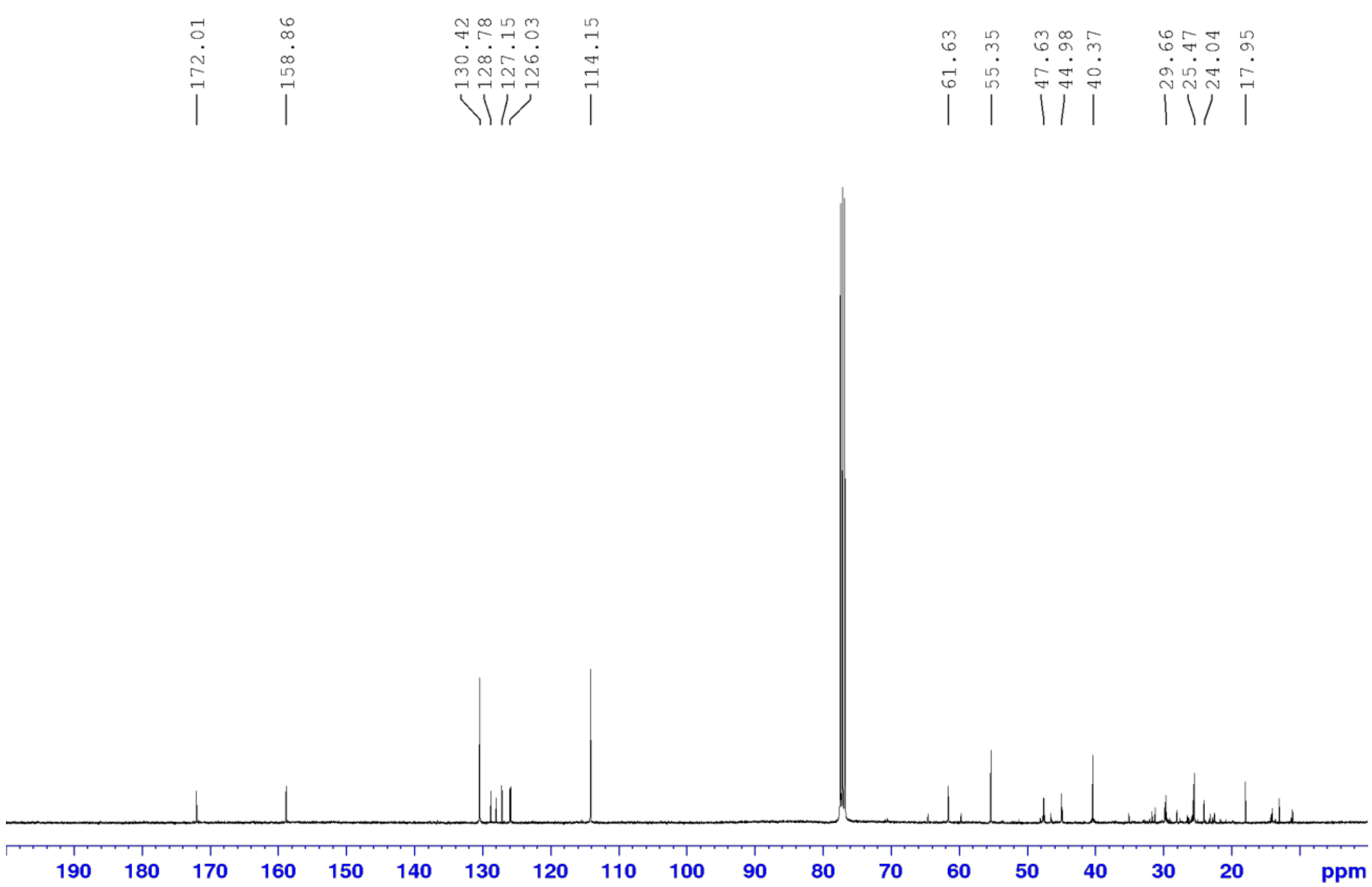

(E)-N-(3-((4-(5-(2-fluorophenyl)-1,2,4-oxadiazol-3-yl)benzoyl)oxy)propyl)hex-4-en-1-aminium (37)

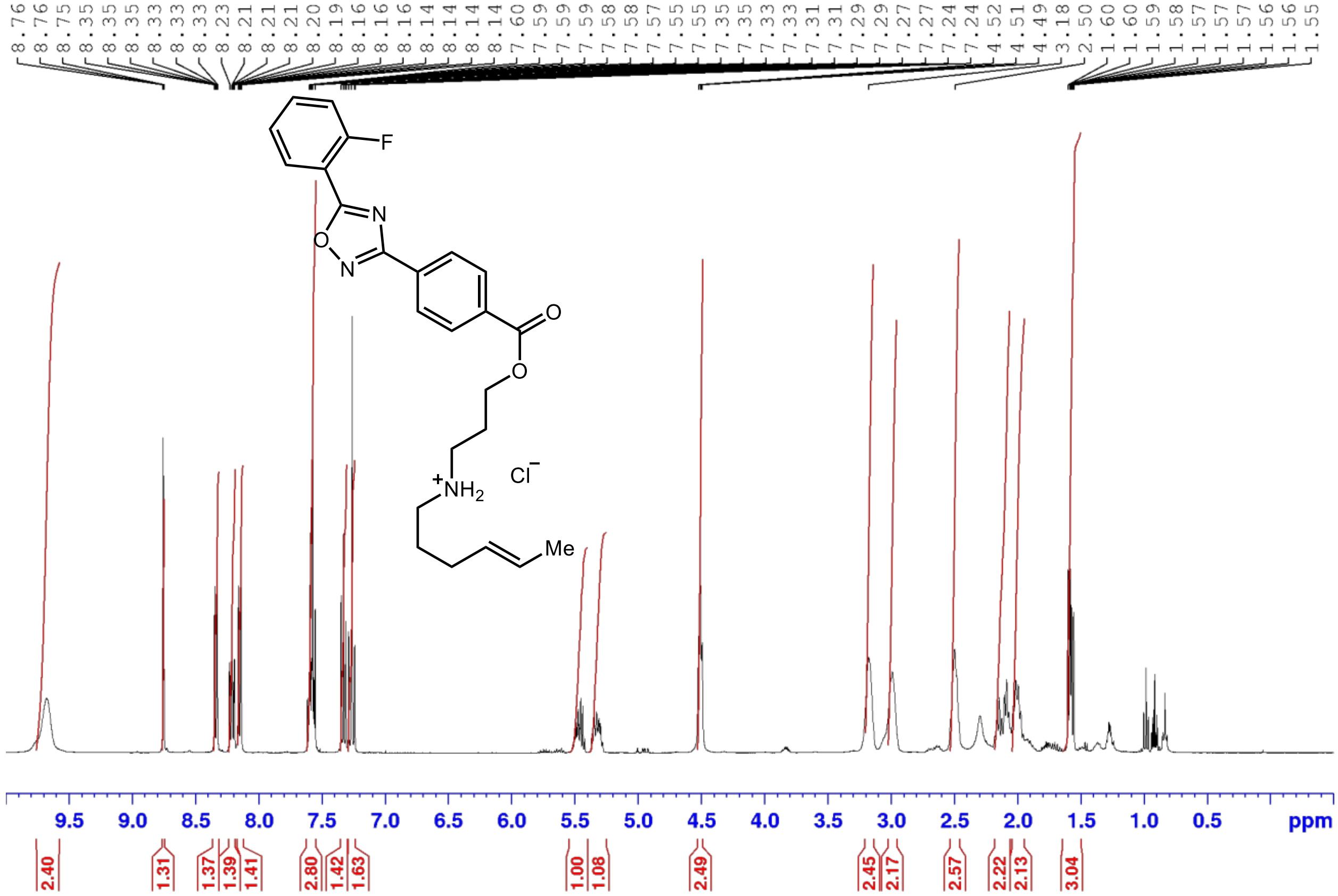

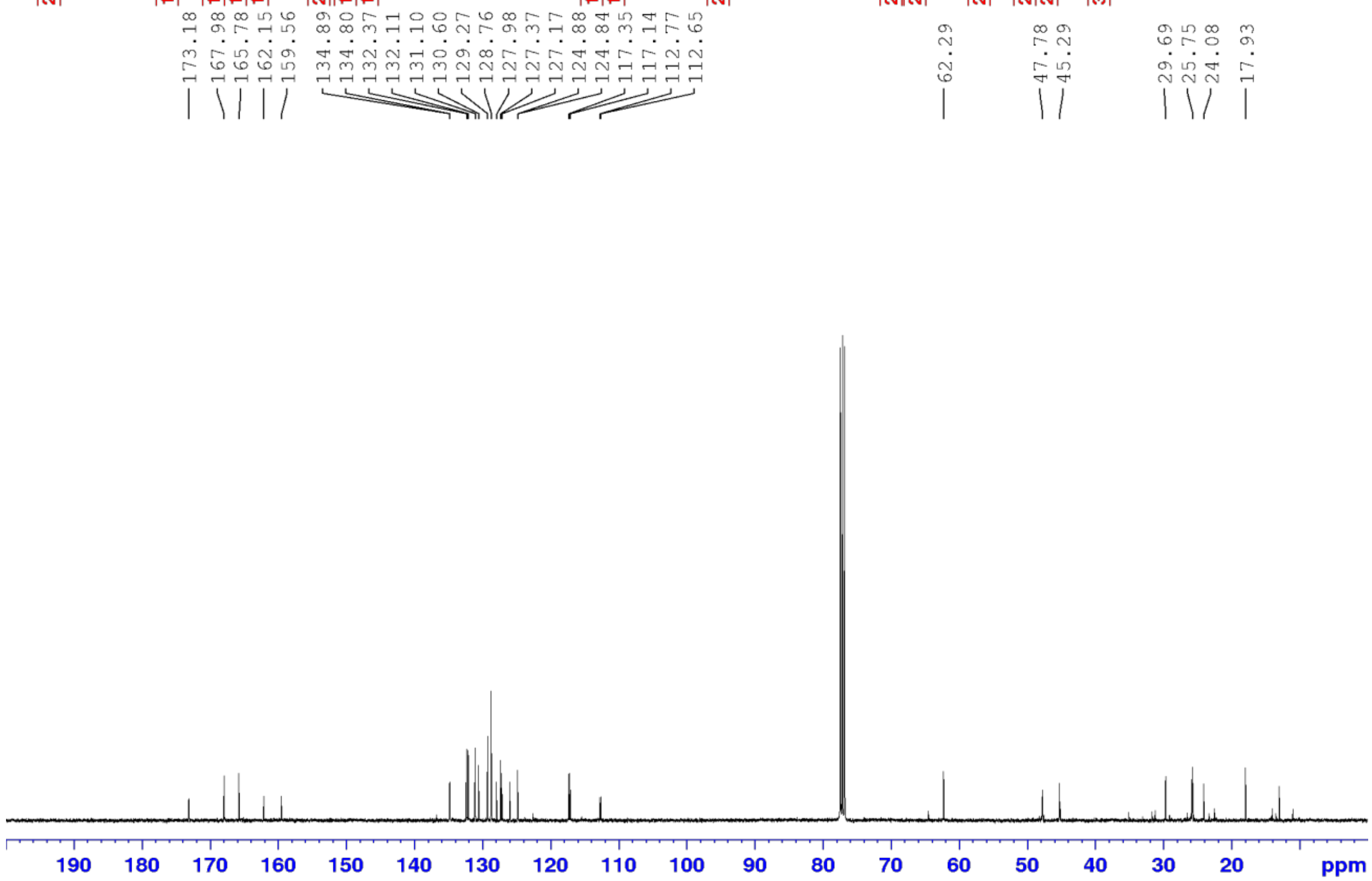

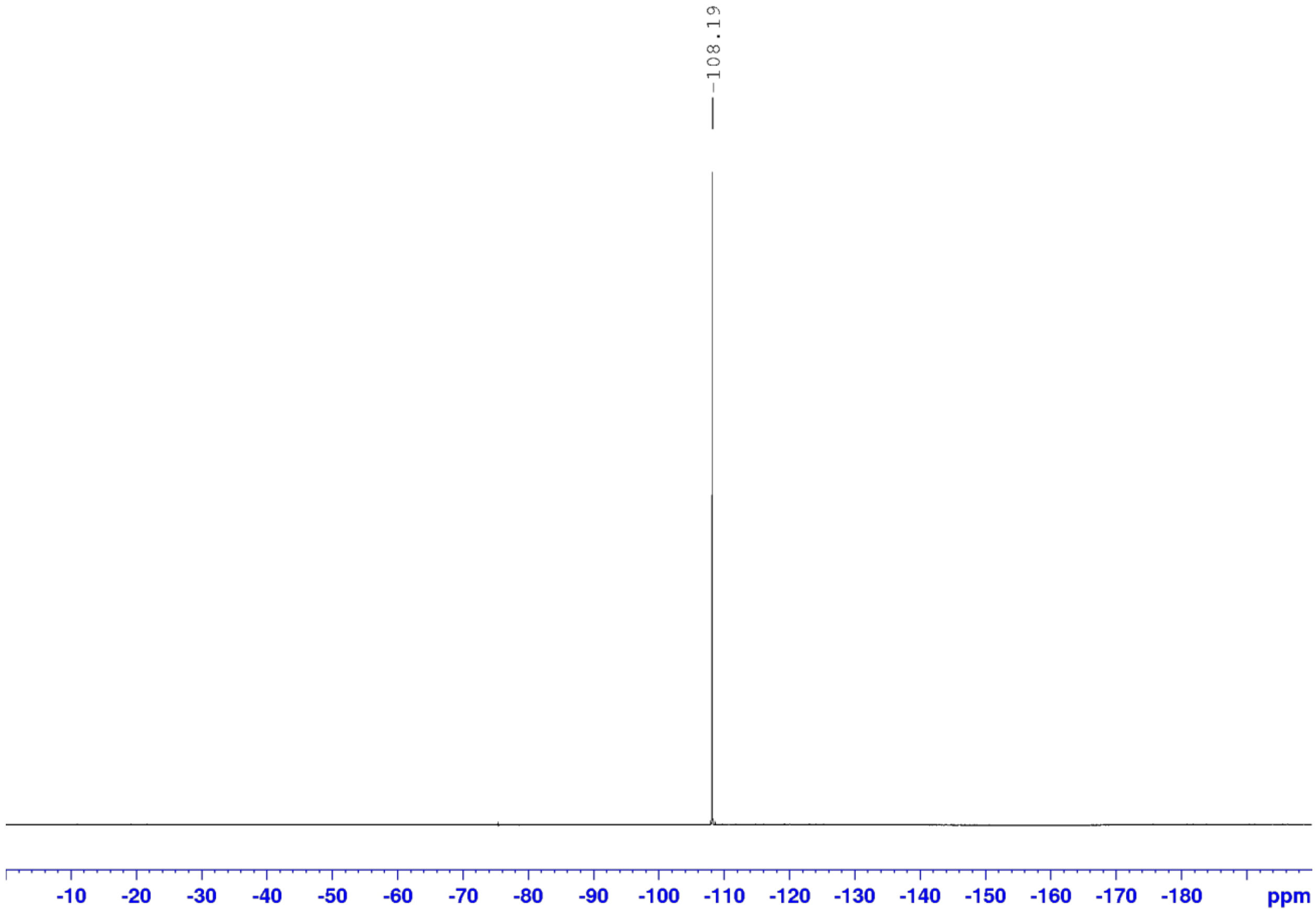
-108.19
-10
-20
-30
-40
-50
-60
-70
-80
-90
-100
-110
-120
-130
-140
-150
-160
-170
-180
ppm
